# Multi-trajectories of BMI, waist circumference, gut microbiota, and incident dyslipidemia: a 27-year prospective study

**DOI:** 10.1128/msystems.00243-25

**Published:** 2025-04-28

**Authors:** Xiaofan Zhang, Fangxu Guan, Wanglong Gou, Qi Wang, Shufa Du, Chang Su, Jiguo Zhang, Ju-Sheng Zheng, Huijun Wang, Bing Zhang

**Affiliations:** 1 National Institute for Nutrition and Health, Chinese Center for Disease Control and Prevention 337010, Beijing, China; 2 Key Laboratory of Public Nutrition and Health, National Health Commission of the People’s Republic of China 33132 , Beijing, China; 3 Zhejiang Key Laboratory of Multi-Omics in Infection and Immunity, Center for Infectious Disease Research, School of Medicine, Westlake University School of Medicine 681395, Hangzhou, Zhejiang, China; 4 Chaoyang District of Beijing Centre for Disease Control and Prevention 661283 , Beijing, China; 5 Department of Nutrition and Carolina Population Center, University of North Carolina at Chapel Hill 312055, Chapel Hill, North Carolina, USA; 6 Westlake Laboratory of Life Sciences and Biomedicine, Hangzhou, China; North Carolina Agricultural and Technical State University, Greensboro, North Carolina, USA

**Keywords:** body mass index, waist circumference, dyslipidemia, gut microbiota, prospective study

## Abstract

Evidence is insufficient to establish a longitudinal association between combined trajectories of body mass index (BMI) and waist circumference (WC) and dyslipidemia. This study investigated the associations between multi-trajectories of BMI and WC over 24 years and the subsequent risk of dyslipidemia in a large cohort of 10,678 Chinese adults from the China Health and Nutrition Survey. Utilizing a group-based trajectory model, we identified four distinct trajectories: normal, normal-increasing, overweight-increasing, and obesity-increasing. Our results indicated that ascending trajectories of BMI and WC are significantly associated with increased odds of dyslipidemia, particularly in males, with odds ratios (OR) of 2.10, 2.69, and 3.56 for the normal-increasing, overweight-increasing, and obesity-increasing groups, respectively. Among females, the normal-increasing group exhibited a significant increased risk (OR: 1.54). Furthermore, we explored the gut microbiota associated with these trajectories, identifying 3, 8, and 4 bacterial genera linked to increasing BMI and WC in males, alongside two genera in females with the normal-increasing trajectory. We identified a total of 23, 25, and 10 differential metabolites significantly associated with these genera, except for Group 2 in males. The inclusion of relevant microbiome and metabolite data improved the model’s predictive capacity for the risk of dyslipidemia, with ROC values increasing from 0.655 to 0.875. Our findings underscore the critical implications of continuous weight gain on metabolic health and suggest that gut microbiota may play a pivotal role in understanding these associations.

**IMPORTANCE:**

Emerging evidence suggests a close connection between the gut microbiome and both human obesity and dyslipidemia, suggesting that the gut microbiome may play an important role in the obesity-dyslipidemia relationship. In this study, we observed several characteristic genera, including *Clostridium_sensu_stricto_1*, *Turicibacter*, and CHKCI002 among males and *Parabacteroides* and *[Eubacterium]_brachy_group* among females, which were negatively associated with high-risk trajectories. They were also related to free fatty acids (FFAs) and oxidized lipid metabolites. These shared and unique gut microbial and metabolic signatures among combined trajectories of BMI and WC with a higher risk of dyslipidemia could provide important evidence for the omics mechanism pathway of long-term obesity trend leading to dyslipidemia.

## INTRODUCTION

Dyslipidemia is a major risk factor for cardiovascular disease and a leading cause of death globally, accounting for 46.7% and 44.3% of total deaths in rural and urban areas in China in 2019 (1). Given the increasing global burden and prevalence of dyslipidemia, it is imperative to uncover any novel risk factors and mechanisms to prevent the occurrence and development of dyslipidemia.

Obesity is a major driver of dyslipidemia. Numerous studies have explored the associations between individual obesity indicators and dyslipidemia. Individuals with higher body mass index (BMI) or waist circumference (WC) are more likely to develop dyslipidemia (2–4). Furthermore, BMI and WC can change throughout the lifespan, and several longitudinal studies have assessed the possible effects of long-term changes in anthropometric indices on the risk of dyslipidemia (5, 6). BMI, measured by combining weight and height, is widely used to assess obesity, but it cannot capture the distribution of abdominal adipose tissue as WC does. However, these longitudinal studies only focused on the single obesity index of BMI or WC, and no prospective study between longitudinal combined indices and dyslipidemia has been done. Moreover, traditional longitudinal research methods do not consider subgroups with different development trends. To our knowledge, there are no longitudinal studies reporting the relationship between multi-trajectories of BMI and WC from early adulthood and dyslipidemia among the Chinese population.

Emerging evidence suggests a close connection between the gut microbiome and both human obesity (7–9) and dyslipidemia (10, 11), suggesting that the gut microbiome may play an important role in the obesity-dyslipidemia relationship. However, most studies on the association between BMI/WC and gut microbiome still remain at the cross-sectional level. The longitudinal effects of BMI/WC on the gut microbiome and related metabolites are unclear. Specific gut microbial signatures that demonstrate patterns of BMI/WC changes over time could help explain mechanistic links between obesity and dyslipidemia.

Therefore, we hypothesized that longitudinal changes in BMI and WC are associated with dyslipidemia and may be related to differential changes in gut bacterial genera and metabolites. In this present prospective cohort study followed from 1991 to 2018, we established multi-trajectories based on 24 years of BMI and WC measurement data and revealed the gut bacterial genera and serum metabolites associated with those multi-trajectories.

## RESULTS

### Sample characteristics

Table 1 presents the baseline characteristics of the 10,678 enrolled participants (51.1% females). The mean (±SD) age of males and females was 39.6 ± 14.1 years and 41.0 ± 13.4 years, respectively. The mean follow-up time was 15.9 ± 6.1 years for males and 15.8 ± 6.3 years for females.

**TABLE 1:** Baseline characteristics of the population in the multi-trajectories analysis

Parameter	Male (*n* = 5,222 [48.9%])	Female (*n* = 5,456 [51.1%])
Follow-up time (years)^*a*^	15.9 ± 6.1	15.8 ± 6.3
Age (years)^*a*^	39.6 ± 14.1	41.0 ± 13.4
Urban (%)	29.7	31.8
Education (%)		
Primary and below	39.7	56.3
Junior high	35.7	26.6
Senior high and above	24.6	17.1
Household income (RMB)^*b*^	1,356.5 (629.7, 3,220.6)	1,378.1 (636.7, 3,393.6)
Smoker (%)	64.4	4.7
Alcohol drinker (%)	63.9	11.5
Energy intake (kcal/day)^*a*^	2,594.1 ± 749.2	2,259.4 ± 670.3
Physical activity (METs/week)^*b*^	292.0 (126.0, 528.0)	336.2 (144.1, 589.9)
Body mass index (kg/m^2^)^*a*^	22.1 ± 2.9	22.4 ± 3.2
Waist circumference (cm)^*a*^	78.7 ± 9.4	76.5 ± 9.2

^*a*^ Mean (SD) was reported.

^*b*^ Median (Q1, Q3) was reported.

### Multi-trajectories of BMI and WC

**Fig 1:**
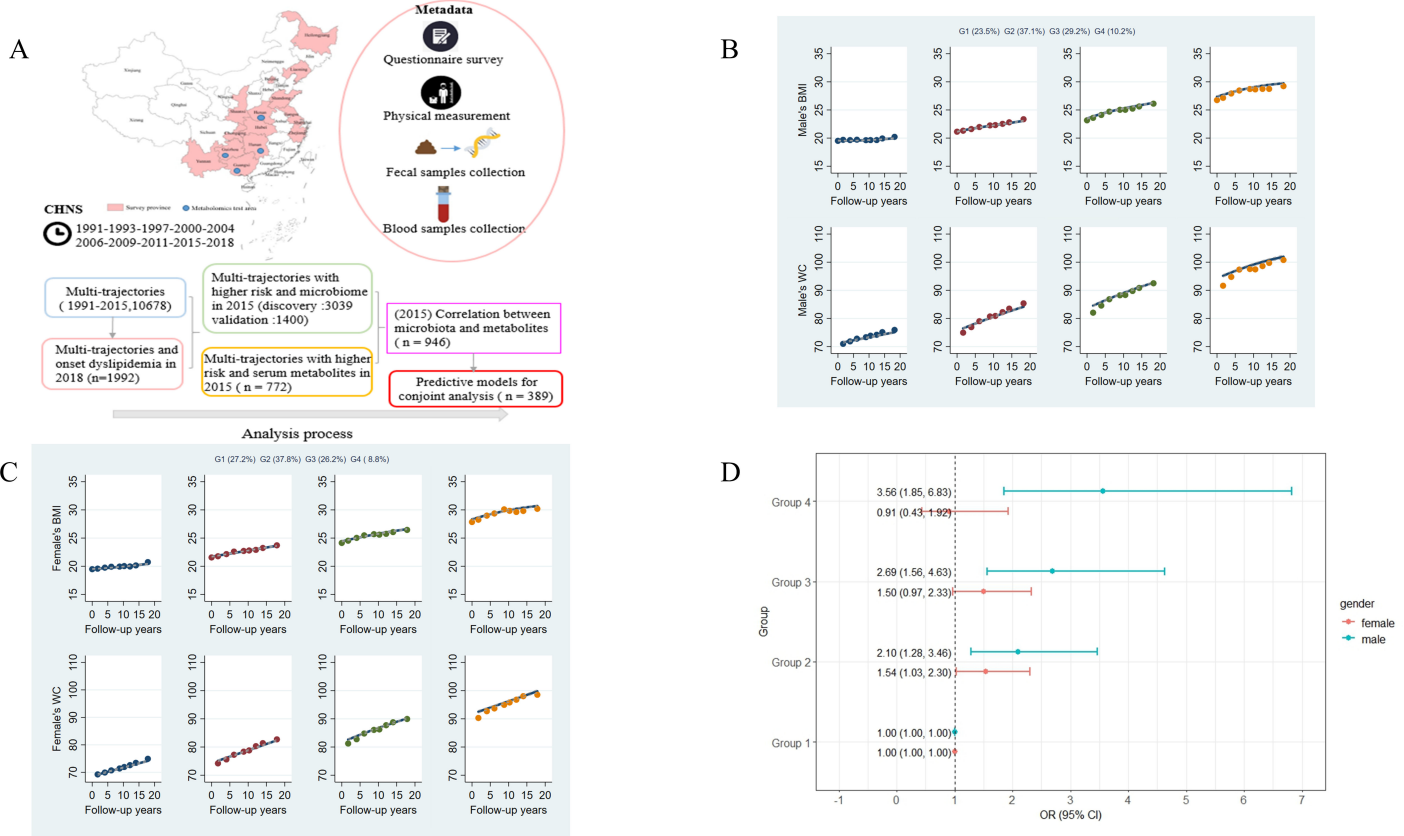
Multi-trajectories of BMI and WC and their associations with dyslipidemia. (**A**) Summary of the study population. This study utilized data from the China Health and Nutrition Survey (CHNS) spanning from 1991 to 2018. The analysis process can be summarized as follows: we first performed a multi-trajectory analysis using samples collected from 1991 to 2015 to examine the association between long-term trajectories of BMI and WC (1991–2015) and the incidence of new-onset dyslipidemia in 2018. Subsequently, we explored the relationships between these identified multi-trajectories and microbiota as well as metabolite profiles in the 2015 samples. This was followed by an in-depth investigation into the correlations between microbiota and metabolites. Finally, we conducted a conjoint analysis that integrated all key factors to provide a comprehensive understanding of the interactions among them. (**B and C**) Multi-trajectories of BMI and WC in the CHNS cohort (1991–2015) among males (**B**) and females (**C**) using GBTM (5,222 males and 5,456 females). The solid lines represent the average estimated BMI and WC over time. The dots represent the actual data, where we weighted each individual’s responses based on posterior probabilities of group membership. (**D**) Associations between multi-trajectories and dyslipidemia based on a binomial logistic regression model (841 males and 1,151 females). Both models were adjusted for age, location (urban/rural), geographical area (province), education level, smoking, drinking, household income, physical activity, and dietary energy intake.

Comparisons between the selected model and other models are shown in Table S1. We finally selected model 4 with a group ratio of more than 5% and the smallest absolute BIC. Four multi-trajectories of BMI and WC among males and females were identified by Group-based multi-trajectory modeling (GBTM) (Fig. 1B and C). We named trajectories based on baseline levels and trends. At baseline and during follow-up, 23.5% (1227) of males and 27.2% (1484) of females had BMI and WC within the normal range and were grouped into the normal trajectory (Group 1). 37.1% (1,937) of males and 37.8% (2,062) of females had normal BMI and WC at baseline and an upward trend during follow-up and were grouped into BMI and WC normal increasing trajectory (Group 2). 29.2% (1,525) of males and 26.2% (1,430) of females had a BMI in the overweight range and WC in the precentral obesity range at baseline and an upward trend during follow-up and were grouped into BMI and WC overweight increasing trajectory (Group 3). 10.2% (533) of males and 8.8% (480) of females had a BMI that was obesity and a WC that was central obesity at baseline, and an upward trend during follow-up was grouped into the obesity-increasing trajectory (Group 4).

### Multi-trajectories and dyslipidemia

Figure 1D shows the results of logistic regression analyses exploring associations between multi-trajectories and risk of dyslipidemia. Among male participants, compared with Group 1, Group 2 (OR: 2.10, 95% CI: 1.28–3.46), Group 3 (OR: 2.69, 95% CI: 1.56–4.63), and Group 4 (OR: 3.56, 95% CI: 1.85–6.83) were significantly associated with a higher risk of dyslipidemia after adjusting for potential confounders. However, among females, only Group 2 (OR: 1.54, 95% CI: 1.03–2.30) was associated with a higher risk of dyslipidemia.

### High-risk multi-trajectories and gut microbiota

**Fig 2:**
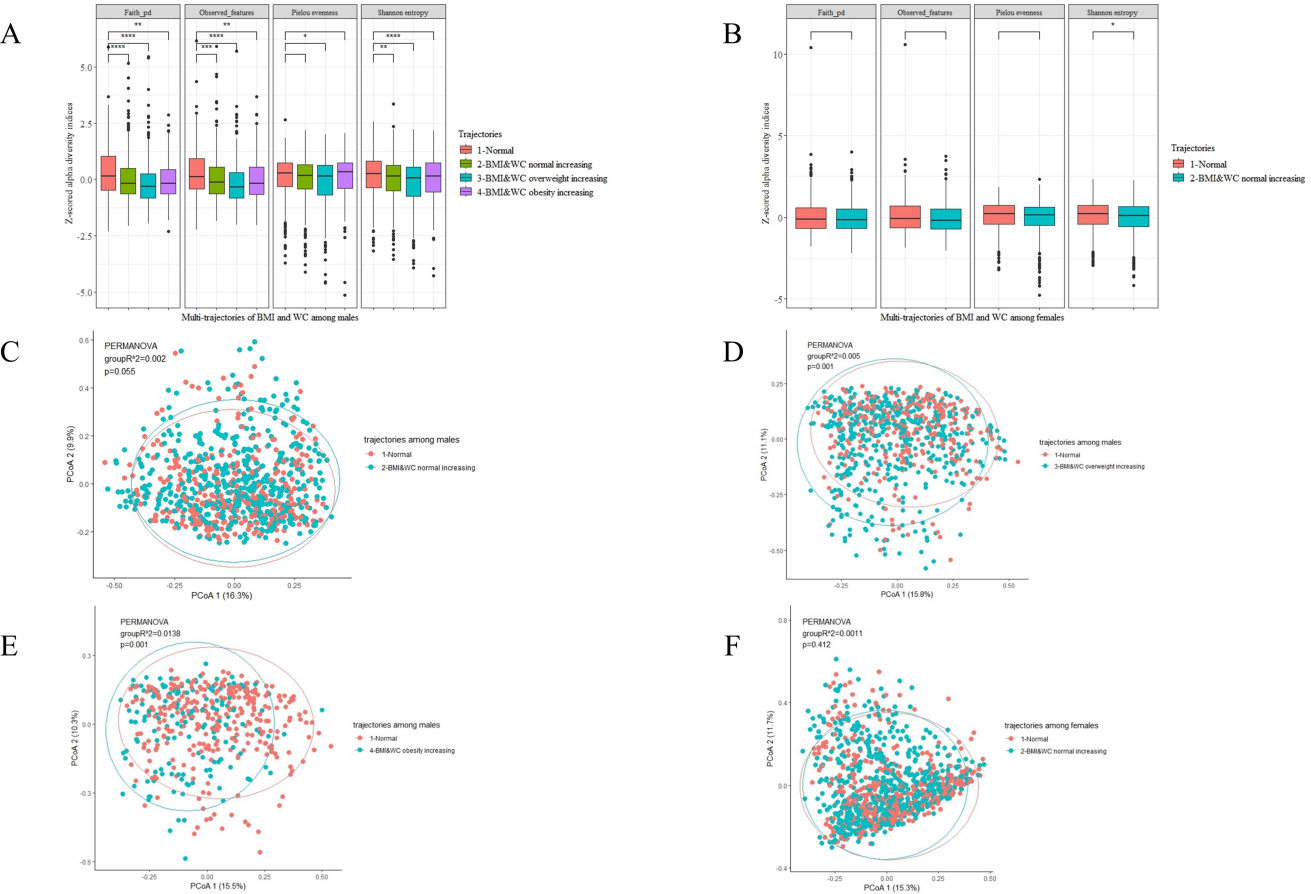
Comparison of the diversity of gut microbiota in multi-trajectories of different sexes (1,434 males and 1,605 females). (**A and B**) Alpha-diversity analysis between males (**A**) and females (**B**). Four alpha-diversity indices were scaled: Shannon’s diversity index, observed features, Pielou’s measure of species evenness, and Faith’s phylogenetic diversity. Comparison between each risk trajectory group and the normal group using the Kruskal-Wallis test. (**C–F**) β-Diversity analysis between males (**C–E**) and females (**F**). Pairwise comparisons were determined by PERMANOVA analyses based on the Bray-Curtis distance. *R*^2^ and *P* values were determined from 999 permutations.

First, we evaluated overall indicators of gut microbiota composition. The overview of microbial composition among the groups is shown in Fig. S1. Among males, we found that all four alpha-diversity indexes in Group 3 were lower than those in Group 1, while three and two alpha-diversity indexes in Group 2 and Group 4 were lower than Group 1, respectively (Fig. 2A). Among females, we only compared the alpha-diversity index between Group 2, which may increase the risk of dyslipidemia, and the reference group (Group 1). The Shannon’s index value was lower in Group 2 than in Group 1 (Fig. 2B). We also identified links between multi-trajectories and overall microbial structure (beta diversity). Among male participants, permutation multivariate ANOVA based on Bray-Curtis distance showed significant differences between Groups 3, 4, and 1, explaining 0.5% and 1.4% of the dissimilarities in the gut microbiota structure (PERMANOVA test, *P*＝0.001) (Fig. 2D and F). Results of the permutational dispersion test for distances within the group are seen in Table S2.

**Fig 3:**
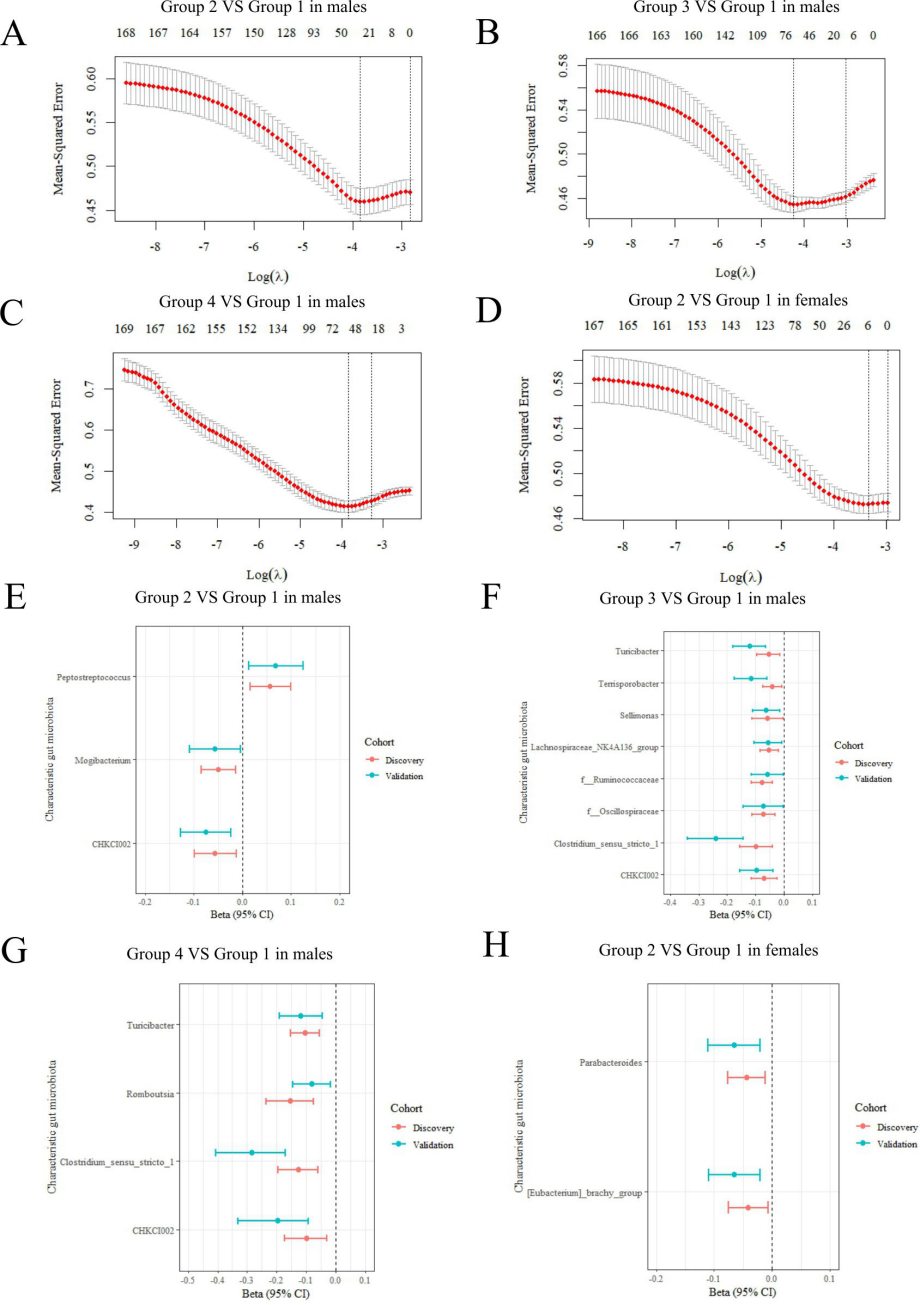
Characteristic genera for those multi-trajectories with higher risk of dyslipidemia. (A–D) Discovery cohort: 1,434 males and 1,605 females. Cross-validation curves of 169 bacteria genera for screening of characteristic genera by LASSO regression (discovery cohort). Characteristic genera selection for BMI and WC normal increasing trajectory (Group 2) in male (**A**), BMI and WC overweight increasing trajectory (Group 3) in male (**B**), BMI and WC obesity increasing trajectory (Group 4) in male (**C**) and BMIWC normal increasing trajectory (Group 2) in female (**D**). (E–H) Validation cohort: 650 males and 750 females. Characteristic genera with statistical significance after validation in both the discovery cohort and validation cohort by logistic regression. Association of selected characteristic genera with BMI and WC normal increasing trajectory (Group 2) in male (**E**), BMIWC overweight increasing trajectory (Group 3) in male (**F**), BMI and WC obesity increasing trajectory (Group 4) in male (**G**), and BMI and WC normal increasing trajectory (Group 2) in female (**H**).

We then explored those multi-trajectories characteristic genera with a higher risk of dyslipidemia. Among males, 30, 66, and 54 microbial genera were obtained for Groups 2, 3, and 4 by LASSO regression using minimum cross-validation error as a parameter in the validation data set (Fig. 3A through C). Among females, the model retained only six bacterial genera as characteristic genera for Group 2 (Fig. 3D). These selected bacterial genera were further validated by logistic regression. After dual verification, 3, 8, 4, and 2 characteristic bacteria genera were retained for different sexes (Fig. 3E through G). Among males, both Group 3 (*r* =−0.10 and −0.24 in discovery and validation cohort) and Group 4 (*r* ＝−0.13 and −0.28 in discovery and validation cohort) were negatively associated with genus *Clostridium_sensu_stricto_1*, with the validation cohort having a higher absolute coefficient value. The same is true for the genus *Turicibacter*. Meanwhile, the genus CHKCI002 should also be mentioned, as all Groups 2, 3, and 4 were negatively correlated with it, and correlations were growing (Tables S1 to S3). Among females, Group 2 was negatively associated with the genus *Parabacteroides* and *[Eubacterium]_brachy_group* (Table S4). The correlation was considered statistically significant when the FDR *P* value was less than 0.05. Detailed results are shown in Tables S1 to S4.

### Key serum metabolites associated with high-risk multi-trajectories and related gut microbiota

**Fig 4:**
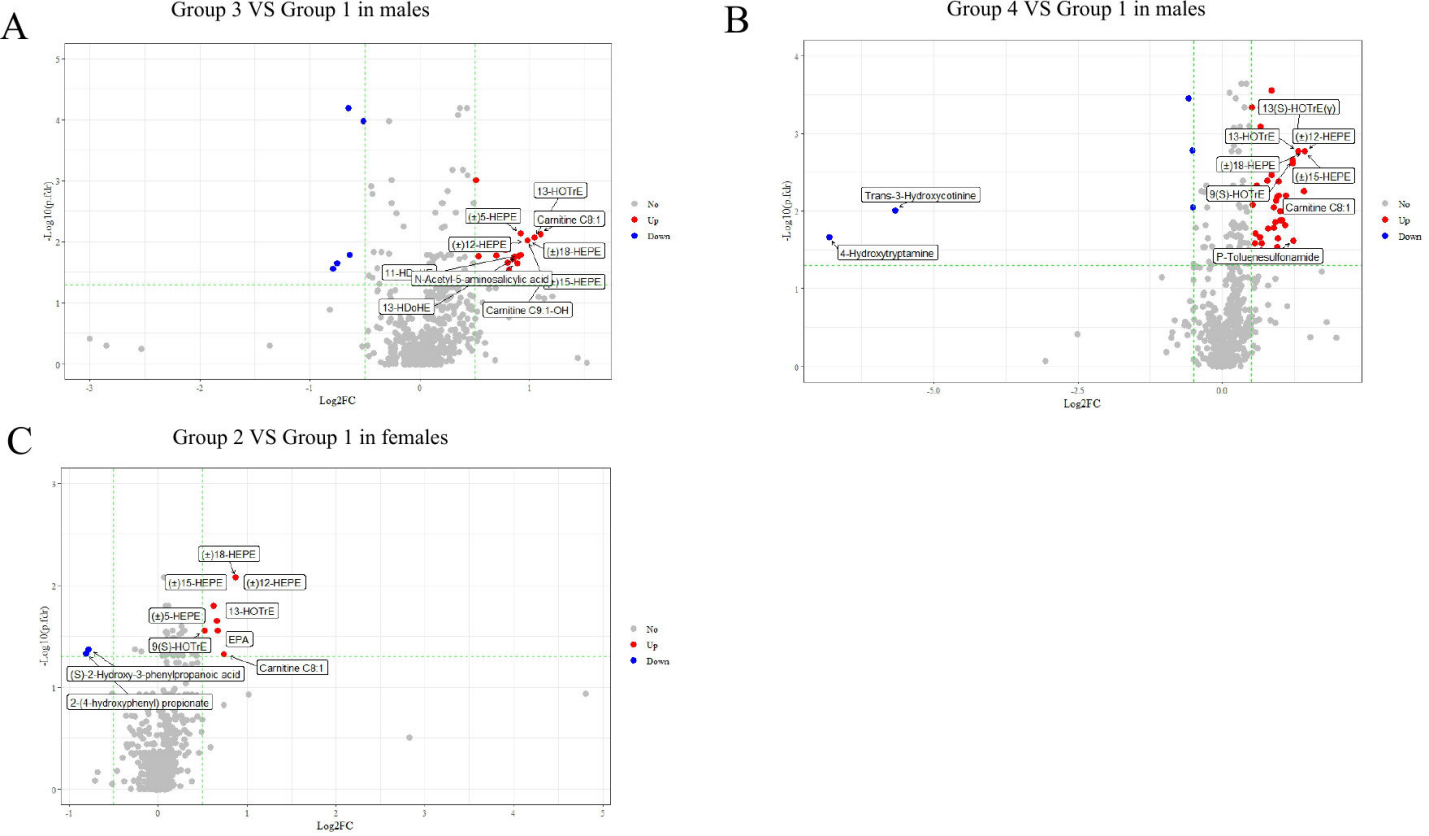
Volcanic map of differential metabolites (*P* < 0.05, |log2(FC) > 0.5 considered to be differential, 334 males and 438 females). Differential metabolites were found between the normal group (Group 1) and BMI and WC overweight increasing trajectory (Group 3) in male (**A**), BMI and WC obesity increasing trajectory (Group 4) in male (**B**), and BMI and WC normal increasing trajectory (Group 2) in female (**C**). Blue was the downregulated differential metabolite, red was the upregulated differential metabolite, and metabolites with no difference were marked as gray. The *P* value was further adjusted for multiple testing of pairwise comparison using the Benjamini-Hochberg method. Panels A and B show only the top 10 metabolites with significant differences, respectively, and the top 10 metabolites were sorted according to the absolute value of log2FC.

We initially identified several differential metabolites across multi-trajectories with a higher risk of dyslipidemia compared to the normal group, using the Wilcoxon test and fold change (FC) value. In males, no differences were observed in the metabolites of Group 2 (Table S5). In Group 3, we detected 36 differential metabolites, of which 30 were significantly upregulated. For Group 4, 52 out of 57 differential metabolites were also upregulated (Fig. 4A and B; Table S6 and S7). In females, we identified 10 differential metabolites in Group 2 (Fig. 4C; Table S8).

**Fig 5:**
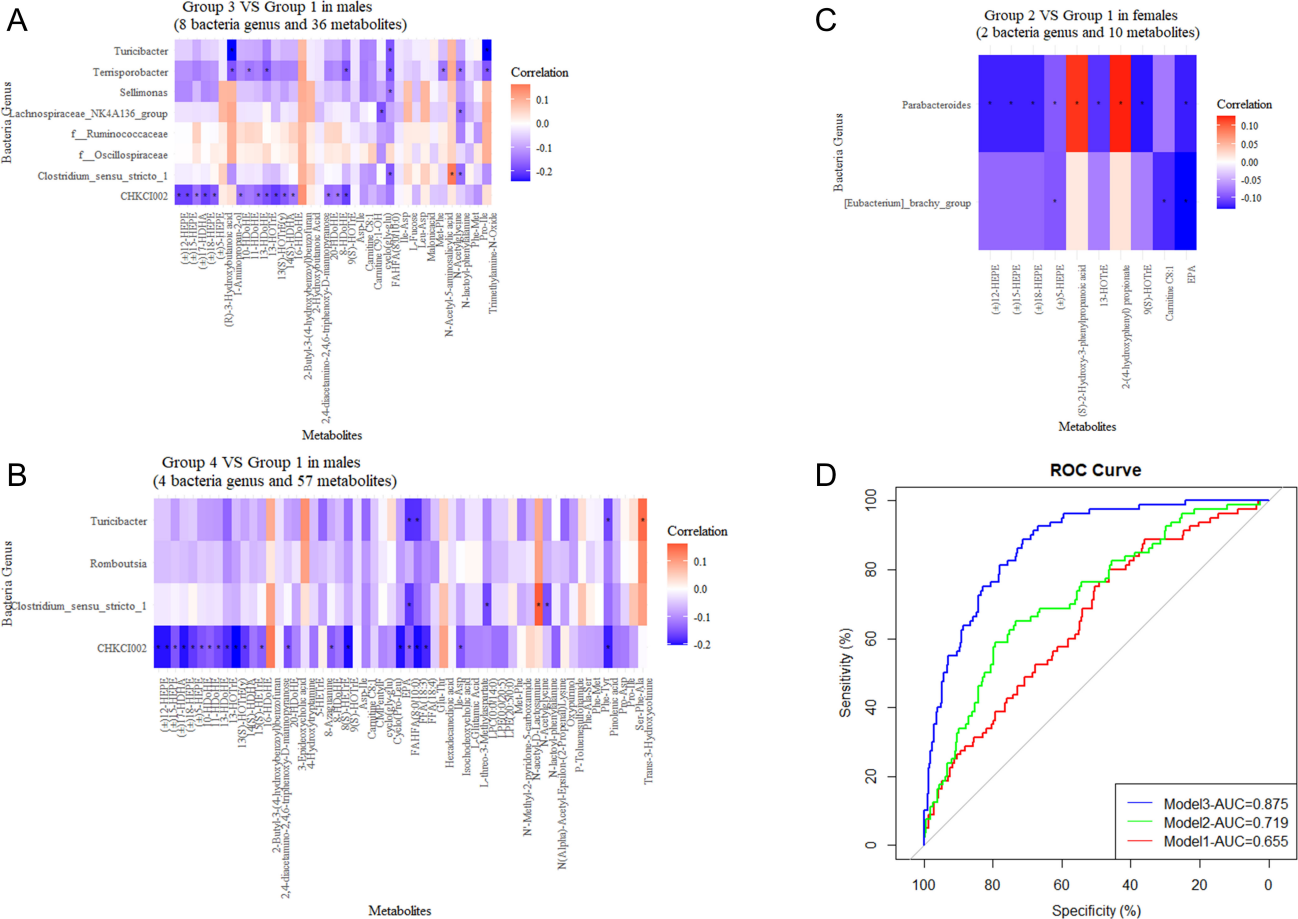
Spearman’s rank correlation between validated characteristic genera and differential metabolites (393 males and 553 females), and prediction models for the risk of dyslipidemia. (**A**) Association of validated eight characteristic genera for BMI and WC overweight increasing trajectory (Group 3) with 36 differential metabolites (between Group 3 and Group 1) in males. (**B**) Association of validated four characteristic genera for BMI and WC obesity increasing trajectory (Group 4) with 57 differential metabolites (between Group 4 and Group 1) in males. (**C**) Association of validated two characteristic genera for BMI and WC normal increasing trajectory (Group 2) with 10 differential metabolites (between Group 2 and Group 1) in females. (**D**) Logistic regression models to predict the risk of dyslipidemia. Model 1 included the trajectories and relevant covariates (age, gender, location [urban/rural], geographical area [province], education level, smoking, drinking, household income, physical activity, and dietary energy intake), model 2 further added the validated characteristic genera, and model 3 further added the differential metabolites.

We then examined the associations between characteristic microbiota (*n* = 8 in Group 3, = 4 in Group 4 in males, and = 2 in Group 2 in females) and differential metabolites (*n* = 36 in Group 3, = 57 in Group 4 in males, and = 10 in Group 2 in females) using Spearman correlation analysis (Fig. 5). The results showed that, in males, 23 metabolites were significantly associated with the characteristic genera of Group 3. Among them, FAHFA (8:0/10:0) was negatively associated with the genera *Clostridium_sensu_stricto_1* (r＝−0.17) and *Turicibacter* (r ＝−0.18). N-lactoyl-phenylalanine was negatively correlated not only with the genera *Clostridium_sensu_stricto_1* (r＝−0.15) but also with two other characteristic bacteria (*Lachnospiraceae_NK4A136_group* and *Terrisporobacter*). Negative associations were also found among trimethylamine-n-oxide, 1-aminopropan-2-ol, and genera *Turicibacter* and *Terrisporobacter* (Fig. 5A; Table S9). Twenty-five metabolites were significantly associated with characteristic genera of male Group 4. Among them, the result of FAHFA (8:0/10:0) was similar to those of Group 3. In addition, negative associations were also found among FFA (18:3), pinolenic acid, and genera *Turicibacter* and CHKCI002 (Fig. 5B; Table S9). In females, all 10 differential metabolites were significantly associated with characteristic genera in Group 2. Among them, negative associations were found among EPA, (±)5-HEPE, and genera *Parabacteroides* and *[Eubacterium]_brachy_group* (Fig. 5C; Table S10).

### Prediction models for the risk of dyslipidemia

For conjoint analysis, different models were constructed to evaluate the predictive performance of dyslipidemia. Model 1 included BMI and WC multi-trajectories and 10 related covariates, model 2 further added the characteristic bacteria genera obtained in the study, and model 3 added the differential metabolites on the basis of model 2. The inclusion of relevant microbiome and metabolite data improved the model’s predictive capacity for the risk of dyslipidemia, with ROC values increasing from 0.655 to 0.875 (Fig. 5D).

## DISCUSSION

The main results of our cohort study, based on anthropometric measurements, blood and fecal samples, and 27 years of data, are as follows. First, four multi-trajectories of BMI and WC among males and females were identified, with the same increasing trend from 1991 to 2015. Most of the population was in the BMI and WC normal growth group (Group 2). In addition, male participants with increasing trends in BMI and WC had a higher risk of dyslipidemia in 2018, with OR value increasing as baseline BMI and WC shifted from normal to obesity. However, in female participants, we only found an increased risk of dyslipidemia in Group 2. And second, we found several important characteristic genera negatively associated with groups that remained overweight/obesity or developed overweight/obesity, including *Clostridium_sensu_stricto_1*, *Turicibacter*, and CHKCI002 among males and *Parabacteroides* and *[Eubacterium]_brachy_group* among females. These important characteristic genera are also related to some differential metabolites, such as FAHFA (8:0/10:0), N-lactoyl-phenylalanine, trimethylamine-n-oxide, 1-aminopropan-2-ol, FFA (18:3), pinolenic acid, EPA, and (±)5-HEPE; most of them belong to free fatty acids (FFAs) and oxidized lipids.

### Multi-trajectories of BMI and WC are associated with the risk of dyslipidemia

Using 24 years of BMI and WC measurements and the GBTM method, we successfully established four distinct multi-trajectories of BMI and WC in the study sample. This grouping can not only reflect the status of obesity and central obesity but also show the baseline obesity level and long-term change trends simultaneously. To the best of our knowledge, only one study (12) reported a multi-trajectory of BMI and WC, and its results were similar to our study. In addition, the BMI trajectories (13, 14) and WC trajectories (15) derived from large samples of adults only also showed four increasing trend groups. This reminds us that the BMI and WC of Chinese adults seem to be on the rise simultaneously and need to be controlled urgently.

In our study, male Groups 2, 3, and 4 were associated with a higher risk of dyslipidemia. This is different from females. A cross-sectional study among Chinese primary school children also found sex differences in anthropometric indicators predicting dyslipidemia, which may be more true for boys than for girls (16). Another study of Chinese adults showed that males with obesity also had a higher risk of dyslipidemia than females (17). This sex disparity may be due to differences in lifestyle between males and females, which are risk factors for dyslipidemia (18). A possible explanation may also be related to sexual dimorphism in fat distribution and hormone levels. A study by Nedungadi and Clegg (19) showed that females have more subcutaneous fat and males have more visceral fat, suggesting a greater risk of dyslipidemia. In addition, studies have shown that females have higher leptin and adiponectin levels (considered cardiovascular-protective factors [20, 21]) than males (22, 23). Therefore, the association between BMI and WC and the risk of dyslipidemia is weaker in females than in males. To date, we are not aware of any longitudinal study evaluating the relationship between multi-trajectories of BMI and WC and dyslipidemia. Limited evidence from cohort studies suggests that the odds of developing dyslipidemia are associated with increased BMI (4) and WC (5), respectively. More effective attention and interventions should be taken to manage BMI and WC to reduce the prevalence of dyslipidemia, especially in males.

### Specific microbiota are associated with high-risk multi-trajectories

Little is known about the contribution of *Clostridium sensu stricto 1* to human gut health. Two mouse experiments in 2021 and 2022 suggest it may be a novel biomarker of obesity/obesity resistance (24, 25). Our longitudinal study in adult males also confirmed this new finding, suggesting that *Clostridium sensu stricto 1* might help prevent obesity. *Clostridium* is a known producer of butyrate (26), which contributes to the integrity of the intestinal barrier, attenuates chronic inflammation by promoting regulatory T cells, and prevents pathogen proliferation (27). Therefore, loss of butyrate-producing bacteria, such as *Clostridium*, induces chronic low-grade inflammation. However, conflicting results for *Clostridium sensu stricto 1* also existed. Evidence from the animal feeding experiment (28) and intervention trials in patients with obesity (29) both indicate that *Clostridium sensu stricto 1* is reduced after weight loss interventions. Similar to *Clostridium sensu stricto 1,* CHKCI002 also showed a positive correlation with butyrate in ducks (30). Farkas V first reported the negative correlation between the CHKCI002 genus and chicken body weight in 2022 (31). In this study, CHKCI002 was also negatively associated with all risk groups in males, suggesting a beneficial effect of CHKCI002 on obesity indicators. Many studies have linked *Turicibacter* to host lipid metabolism profile, but the results are inconsistent (32–34). This may be a result of phenotypic diversity among *Turicibacter*, where hosts may experience different lipid outcomes depending on their own specific *Turicibacter* strains. A recent study by Lynch JB (35) further identified genes capable of altering host bile acids and lipid metabolism in *Turicibacter* strains and positioned *Turicibacter* bacteria as modulators of host lipid biology. Our data show that *Turicibacter* is negatively associated with BMI and WC in the overweight or obesity-increasing trajectory groups (two groups with a higher risk of dyslipidemia), supporting these previous studies. This also highlights the advantage of long-term trajectory characteristic microbiota of obesity indicators in predicting dyslipidemia.

In addition to the above-mentioned beneficial bacteria found in males, we also found two dominant beneficial bacteria in females: the genus *Parabacteroides* and *Eubacterium brachy group*. Members of the genus *Parabacteroides* are saccharolytic bacteria that produce major end products of fermentation, such as acetic acid and succinic acid (36). According to numerous studies, the relative abundance of *Parabacteroides* is negatively associated with BMI (37, 38), which is consistent with our study. There are limited studies on the association of the *Eubacterium brachy group* with obesity or blood lipids. We found only one study on adult mice showing that a high-fat diet reduced the abundance of the *Eubacterium brachy group* at 18 weeks (39). Members of the genus *Eubacterium* can undergo bile acid and cholesterol transformations in the gut, thereby contributing to its homeostasis (40). Gut microbiologists agree that specific butyrate-producing microbial strains belonging to the genera *Eubacterium* may ultimately be considered as beneficial to human health as Lactobacillus and Bifidobacterium strains (41).

### Key serum metabolites are associated with high-risk multi-trajectories and related gut microbiota

N-lactoyl-phenylalanine (Lac-Phe) was proposed as an “exercise hormone” that suppresses appetite and adiposity in diet-induced mice with obesity; however, Li et al. demonstrated complete inactivity of Lac-Phe when administered orally (42). In this study, the amount of Lac-Phe in the overweight/obesity-increasing group was higher than that in the normal group, which is inconsistent with previous animal studies. Future work will help elucidate whether Lac-Phe can help outrun obesity. As a bioactive metabolite of the gut microbiota, trimethylamine-N-oxide (TMAO) plays a critical role in the progression of many diseases, including diabetes (43), obesity (44), atherosclerosis, and cardiovascular risks (45). In these disease states, elevated circulating TMAO concentrations are commonly observed. Meta-analyses also revealed a positive dose-dependent association between circulating TMAO concentrations and obesity (46). Mechanisms that may contribute to obesity include the role of FMO 3 (TMAO-producing enzyme) in obesity regulation and adipose tissue formation (44), as well as increased hepatic insulin resistance and consequent obesity through increased TMAO concentrations (47).

γ-Linolenic acid (GLA, 18:3 n-6), pinolenic acid, and EPA (the first two belong to omega-6 polyunsaturated fatty acids [PUFA], and the last one belongs to omega-3 PUFA) were significantly higher in Groups 3 and 4 and were negatively correlated with the beneficial bacteria *Turicibacter*, CHKCI002*, Parabacteroides*, and *[Eubacterium]_brachy_group* in this study. A review study showed that n-6 PUFA-derived eicosanoids have pro-inflammatory effects, whereas n-3 PUFA-derived eicosanoids have anti-inflammatory activities (48). Research by Sunhye Shin (49) further found that the high n-6:n-3 ratio of linoleic acid-rich oil increased lipogenesis and reduced lipid oxidation and thermogenesis. More importantly, adequate intake of n-3 PUFA can significantly influence the effects of n-6 PUFA on lipoprotein profiles (50). Therefore, the ratio of n-6 PUFA to n-3 PUFA is more important than the amount of a single n-6 PUFA or n-3 PUFA. This study found that groups with overweight or obesity have higher concentrations of n-3 and n-6 fatty acids and a higher risk of dyslipidemia, which may be related to the ratio of the two.

This study has several strengths. First, this is the first study to jointly use BMI and WC indicators to explore the association between long-term trajectories of comprehensive obesity indicators and the risk of dyslipidemia in a large 24-year natural population cohort. In addition, for the first time, we explored the key microflora and metabolites in different long-term obesity status trajectory groups with higher risk of dyslipidemia, providing important evidence for the omics mechanism pathway of obesity leading to dyslipidemia. This study also has limitations. First, gut microbiome and metabolome data were collected only in 2015, and longitudinal changes in omics and causal associations could not be observed. However, we conducted validation across different data sets and compared differential metabolites by both *P*-value and FC value. Second, although we adjusted for numerous covariates, we cannot completely rule out the possibility of residual confounding. Third, the associations of obesity with functional profiles of the gut microbiome are unclear due to the use of 16S rRNA data.

### Conclusion

In conclusion, our results suggest that obesity indicators influence dyslipidemia in males more than in females. We also identified some potential gut bacteria and differential metabolites associated with long-term BMI and WC trajectories, some of which were closely related to lipid levels, revealing the role of gut bacteria and related metabolites in obesity and lipid metabolism. Integrating microbiome and metabolite data could enhance dyslipidemia risk prediction.

## MATERIALS AND METHODS

### Study population

This study is based on the China Health and Nutrition Survey (CHNS), an ongoing population-based longitudinal study. The CHNS collects demographic information, lifestyle details, physical activity levels, dietary habits, anthropometric measurements, and biological samples (51). Across 11 survey rounds, approximately 15,000 participants were recruited in each round, representing 16 provinces and megacities across China. The most recent data available are from the 2018 survey.

According to the analysis process, our study included six sub-data sets (Fig. 1A). (i) After excluding participants <18 years of age, pregnant or breastfeeding, or patients with cancer, stroke, or disabled patients at the time of the survey, 10,678 individuals (5,222 males and 5,456 females) with at least three weight, height, and WC measurements from 1991 to 2015 were included in the multi-trajectories analysis. (ii) Among these participants, we further excluded those who had developed dyslipidemia before 2018 (*n* = 6,785) or those who had not followed up for 2018 (*n* = 1,901, no lipid data in 2018), leaving 1,992 individuals (841 males and 1,151 females) analyzed the association between the long-term trajectory of BMI and WC (1991–2015) and new onset dyslipidemia in 2018. (iii) When analyzing the relationship between multi-trajectories and gut microbiome, we included participants with 2015 stool samples collected in the trajectory population (1,593 males and 1,780 females in the discovery cohort, 704 males and 818 females in the validation cohort), and excluded those who had taken antibiotics within 3 months, used probiotics within the last 4 weeks, or had gastrointestinal disorders, diarrhea, and intestinal resection. 3,039 individuals (1,434 males and 1,605 females) were included in the discovery cohort, and 1,400 individuals (650 males and 750 females) were included in the validation cohort. (iv) From 997 participants with metabolic data in 2015, we included 772 samples (334 males and 438 females) with both multi-trajectories and metabolic data in 2015 to analyze the differences in metabolites between multi-trajectories. (v) We selected 946 individuals (393 males and 553 females) who had gut microbiome and metabolic data in 2015 to map the connection between metabolites and gut bacteria. (vi) We took the intersection of sample 2 and sample 5 to construct predictive models for conjoint analysis (*n* = 389).

### Data collection

#### Questionnaire survey

Sociodemographic characteristics, including location (urban or rural), geographical area (province), age, sex, education and household income, lifestyle factors (smoking status and alcohol consumption), dietary intake, physical activity, physiological and disease status (pregnancy, lactation, disability, stroke, cancer, gastrointestinal disorders, diarrhea, and intestinal resection), and medication data (the use of antibiotics and probiotics) were collected by face-to-face questionnaire interviews. Dietary energy intake was calculated by combining food intake data with the China food composition table, and the amount of physical activity was calculated by multiplying the amount of exercise time by activity intensity of various intensities.

#### Physical measurement

Anthropometric data, including height, weight, and waist circumference, were measured on-site by trained staff. Adhering to consistent measurement standards and utilizing specialized instruments, in each survey, our trained physicians and nurses measured height and weight without shoes to the nearest 0.1 cm and 0.1 kg. We then calculated BMI as weight in kg divided by height in meters (m) squared (kg/m^2^). We measured waist circumference using an inelastic soft ruler with a division value of 0.1 cm.

According to adult weight criteria WS/T 428-2013 in China, we defined a BMI between 18.5 kg/m^2^ and 23.9 kg/m^2^ as normal, a BMI between 24.0 kg/m^2^ and 27.9 kg/m^2^ as overweight, and a BMI of 28 kg/m^2^ or greater as obesity. We have sex-specific criteria for WC. For females, we defined a WC less than 80 cm as normal, a WC between 80 cm and 84.9 cm as precentral obesity, and a WC greater than or equal to 85 cm as central obesity. For males, we defined a WC less than 85 cm as normal, a WC between 85 cm and 89.9 cm as precentral obesity, and a WC greater than or equal to 90 cm as central obesity.

#### Biological sample collection

Fasting blood samples were collected, stored in dry ice, and sent to the laboratory for storage at −80°C within 3 hours. The plasma was centrifuged within 48 hours and stored at −80℃ for later use. Fecal samples were collected following standard procedures (52) and temporarily stored in a −20°C freezer for 20 minutes and then stored in a laboratory −80°C freezer.

### Assessment of dyslipidemia

Total cholesterol (TC), triglyceride (TG), high-density lipoprotein cholesterol (HDL-C), and low-density lipoprotein cholesterol (LDL-C) were measured using an automatic biochemical analyzer. The definition of dyslipidemia can be found in the Guidelines for the Prevention and Treatment of Dyslipidemia in Chinese Adults (2016 revised edition), including the following thresholds: TC ≥ 6.2 mmol/L, TG ≥ 2.3 mmol/L, LDL-C ≥ 4.1 mmol/L, or non-HDL-C ≥ 4.9 mmol/L.

### Bioinformatics analysis of gut microbiome

Methods of DNA extraction, amplification, and sequencing have been described previously (53). Taxonomic and functional profiles were generated using the Quantitative Insights Into Microbial Ecology 2 platform (QIIME2) (54). Pair-end reads were assembled using the QIIME tools import command. Low-quality regions of the sequences, marker gene Illumina sequences, and chimeric sequences (“consensus”) were filtered using the DADA2 pipeline (55). Reads were then summarized to amplicon sequence variants (ASV) in a feature table and annotated based on the naive Bayes classifier using the classify-sklearn package against the SILVA-132-99 reference sequences (56).

### Serum metabolome analysis

For metabolic analysis, 50 µL of the sample and 300 µL of the extraction solution (ACN:methanol = 1:4, vol/vol) containing internal standards were added to a 2 mL microcentrifuge tube. The sample was vortexed for 3 minutes and then centrifuged at 12,000 rpm for 10 minutes at 4°C. Subsequently, 200 µL of the supernatant was collected and placed at −20°C for 30 minutes, followed by another centrifugation at 12,000 rpm for 3 minutes at 4°C. An aliquot of 180 µL of the supernatant was transferred for LC-ESI-MS/MS analysis. The sample extracts were analyzed using an LC-ESI-MS/MS system (UPLC, ExionLC AD, https://sciex.com.cn/; MS, QTRAP System, https://sciex.com/) following standard protocols. The triple quadrupole-linear ion trap mass spectrometer (QTRAP) was used to perform LIT and triple quadrupole (QQQ) scans, operated and controlled by Analyst 1.6.3 software (Sciex) with standard parameters. The source temperature was 500°C; the ion spray voltage (IS) was 5,500 V (positive) and 4,500 V (negative); the ion source gas I (GSI), gas II (GSII), and curtain gas (CUR) were set at 55, 60, and 25.0 psi, respectively; the collision gas (CAD) was set to high. Instrument tuning and mass calibration were performed with 10 and 100 µmol/L polypropylene glycol solutions in QQQ and LIT modes, respectively. A specific set of MRM transitions was monitored for each period according to the metabolites eluted within this period.

### Statistical analysis

#### Multi-trajectories of BMI and WC

GBTM (57) was used to determine the multi-trajectories of BMI and WC. As the WC assessment criteria are different for different sexes, we performed multi-trajectory modeling with a STATA plug-in using continuous norming (cNORM) distribution for different sexes (58). We tested linear, quadratic, and cubic specifications for trajectory shape for participants in two, three, four, five, and six trajectory groups until we established the best-fitting model. We used statistically rigorous criteria to determine the best fit. (i) With the lowest Bayesian information criterion (BIC), we used the difference size (percentage change) of the BIC to choose between a more complex (with one additional specified trajectory group) and a simpler model; (ii) we included at least 5% of the sample population in each trajectory class; and (iii) we ascertained the average posterior probability value of membership within each group, where values greater than 0.7 indicate adequate internal reliability (59).

#### Association between multi-trajectories and dyslipidemia

We performed logistic regression models to explore the relationship between the sex-specific multi-trajectories (1991–2015) and onset dyslipidemia (2018). We built models for males and females and adjusted for baseline age, location (urban/rural) and geographical area (province), smoking, alcohol use, education, household income, dietary energy intake, and physical activity. Then, we used the “forestplot” and “ggplot2” functions in R to plot the forest plot and display the results of the model. We considered a two-sided *P* value < 0.05 to be statistically significant.

#### Gut microbiome analysis

We performed all gut microbiome analyses separately for different sexes. Four alpha-diversity indices were calculated at a sampling depth of 6000: Shannon’s diversity index, observed features, Pielou’s species evenness measure, and Faith’s phylogenetic diversity. To display the results of the four indicators on the same axis, we used the scale function in R to standardize them, then used the Wilcoxon test to compare these indicators between the dyslipidemia risk trajectory group and the normal group, and finally displayed the results in the form of box graphs. At the genus level, using the “vegdist” function from the R package “vegan,” we calculated Bray-Curtis distances between samples using genera abundance and visualized using PCoA. We then performed a permutational multivariate analysis of variance (PERMANOVA) based on the Bray-Curtis distance to determine whether there were differences between groups. In addition, we ran a permutational dispersion test to assess whether there are within-group distances.

Before identifying the characteristic genera, we first preprocessed the raw genera abundance data. Genus with a presence lower than 10% were excluded, and a centered log-ratio (CLR) transformation was applied. Two cohorts were used, including the discovery cohort (3,039 samples, 1,434 males, and 1,605 females) and the validation cohort (1,400 samples, 650 males, and 750 females). The selection of characteristic genera for those multi-trajectories with a higher risk of dyslipidemia was based on a discovery cohort derived from the least absolute selection and shrinkage operator (LASSO) regression. LASSO regression can simplify the model by adding a penalty function and continuously compressing the coefficients to avoid collinearity and overfitting. Important variables can be efficiently screened with a smaller sample size (60). Validations of these characteristic genera were based on both the discovery cohort and the validation cohort by logistic regression. To correct for the multiple testing issue, the *P* value was adjusted by the false discovery rate (FDR) method.

#### Serum metabolites analysis

Sex-specific metabolomics analyses were also performed. Wilcoxon test was used to identify differential metabolites between different multi-trajectory groups. The ratio of the median of the comparison group to the control group was used as the fold change (FC) value. The selection of differential metabolites was based on the following criteria: *P* value < 0.05 and |log2FC| > 0.5. Raw target metabolite data were log-transformed and standardized before analysis. Relationships between these differential metabolites and the characteristic microbiota of multi-trajectories were analyzed using Spearman correlation. All differential metabolites and correlation results were visualized as volcano maps and heat maps by the ggplot2 package in the R language, respectively. When the FDR-adjusted *P* value was <0.05, a statistical difference was considered.

#### Conjoint analysis

Finally, we combined all variables of interest, including multi-trajectories, gut microbiota, metabolite, and dyslipidemia, by building logistic regression models to predict the risk of dyslipidemia.

## Data Availability

The data sets generated and/or analyzed during the current study are not publicly available; please contact the corresponding authors for detailed information.

## SUPPLEMENTAL MATERIAL

Supplemental material msystems.00243-25-s0001.docx Fig. S1; Tables S1 to S12.

ASM does not own the copyrights to Supplemental Material that may be linked to, or accessed through, an article. The authors have granted ASM a non-exclusive, world-wide license to publish the Supplemental Material files. Please contact the corresponding author directly for reuse.
