# Supplementary material for: Multi-trajectories of BMI, waist circumference, gut microbiota, and incident dyslipidemia: a 27-year prospective study
Source: mSystems. 2025 Apr 28;10(5):e00243-25. doi: 10.1128/msystems.00243-25 (PMC12090771; doi:10.1128/msystems.00243-25)
Supplement: Supplemental material — Fig. S1; Tables S1 to S12. [file msystems.00243-25-s0001.docx]

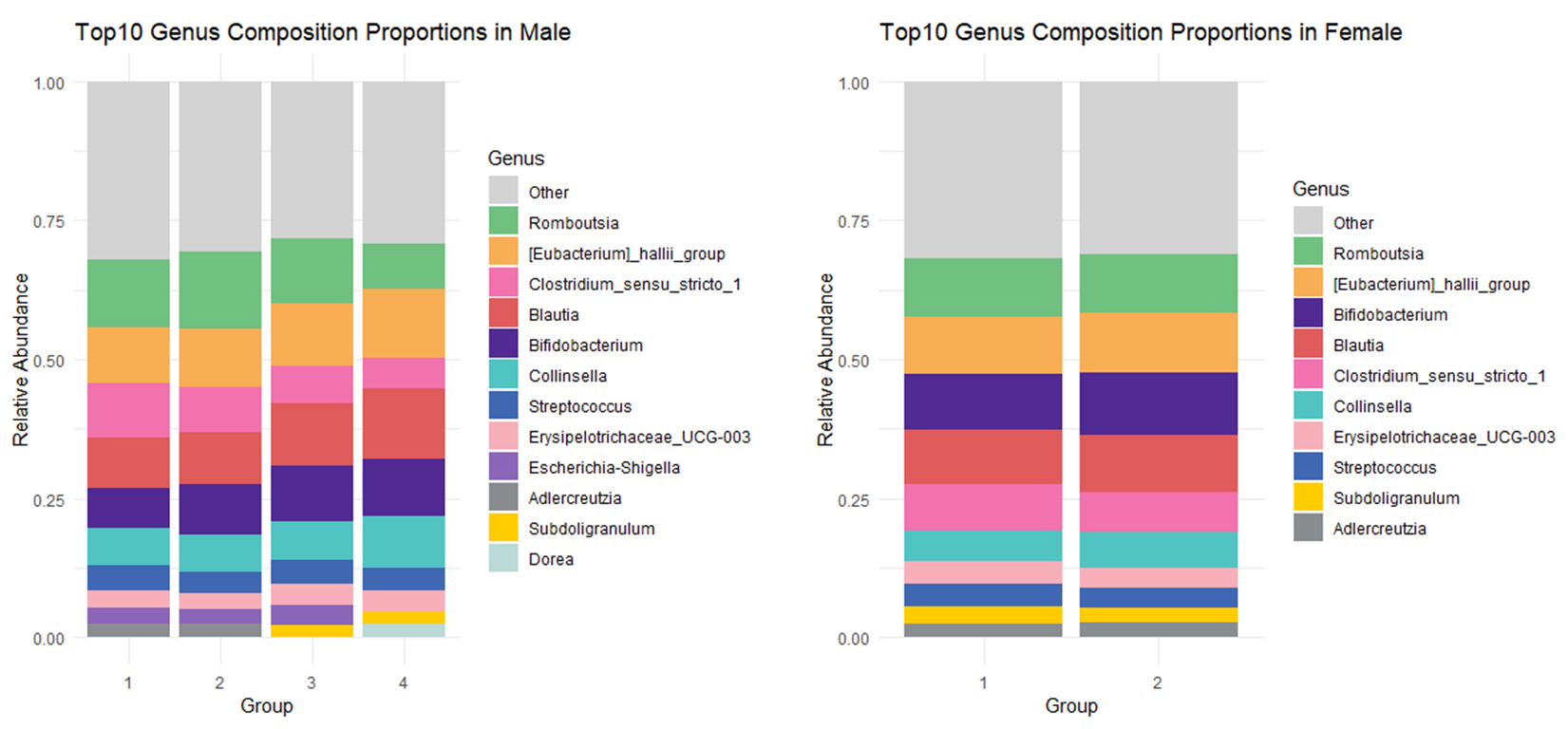


**Figure S1.** Top 10 gut microbiota genus composition proportions in the study population.

**Table S1.** Comparisons between the selected model and other models for defining groups.

| Gender | Model | Group quantity | Group proportion | BIC |
| --- | --- | --- | --- | --- |
| Male | 1 | 1 | G1(100%) | -64762.34 |
| Male | 2 | 2 | G1(60.8%),G2(39.2%) | -59622.64 |
| Male | 3 | 3 | G1(39.9%),G2(42.6%),G3(17.5%) | -57564.16 |
| **Male** | **4** | **4** | **G1(23.5%),G2(37.1%),G3(29.2%),G4(10.2%)** | **-56556.93** |
| Male | 5 | 5 | G1(19.6%),G2(33.2%),G3(27.8%),G4(14.6%),G5(4.9%) | -56113.91 |
| Male | 6 | 6 | G1(13.2%),G2(25.4%),G3(25.2%),G4(20.2%),G5(12.2%),G6(3.9%) | -55807.31 |
| Female | 1 | 1 | G1(100%) | -85499.04 |
| Female | 2 | 2 | G1(60.2%),G2(39.8%) | -78911.62 |
| Female | 3 | 3 | G1(38.0%),G2(44.2%),G3(17.8%) | -76353.03 |
| **Female** | **4** | **4** | **G1(27.2%),G2(37.8%),G3(26.2%),G4(8.8%)** | **-75141.53** |
| Female | 5 | 5 | G1(21.8%),G2(33.7%),G3(26.2%),G4(14.4%),G5(3.9%) | -74577.11 |
| Female | 6 | 6 | G1(9.9%),G2(23.4%),G3(27.6%),G4(22.4%),G5(13.2%),G6(3.5%) | -74209.21 |

**Table S2.** Permutational dispersion test within different multi-trajectory groups.

| Gender | Group | Average distance | F value | P value |
| --- | --- | --- | --- | --- |
| Male | Group 1 | 34552 | 2.355 | 0.125 |
|  | Group 2 | 36525 |  |  |
| Male | Group 1 | 34552 | 9.296 | 0.002 |
|  | Group 3 | 38694 |  |  |
| Male | Group 1 | 34552 | 0.311 | 0.577 |
|  | Group 4 | 33659 |  |  |
| Female | Group 1 | 33759 | 2.958 | 0.085 |
|  | Group 2 | 35615 |  |  |

**Table S3.** Characteristic genera validated by logistic regression for group 2 trajectory in males in both discovery dataset and validation dataset.

| Variable | Coefficient | Ci_lower | Ci_upper | Fdr_p | Data |
| --- | --- | --- | --- | --- | --- |
| CHKCI002 | -0.056 | -0.099 | -0.013 | 0.011 | discovery |
| Mogibacterium | -0.049 | -0.085 | -0.014 | 0.006 | discovery |
| Peptostreptococcus | 0.057 | 0.015 | 0.100 | 0.008 | discovery |
| CHKCI002 | -0.075 | -0.128 | -0.024 | 0.005 | validation |
| Mogibacterium | -0.056 | -0.109 | -0.005 | 0.034 | validation |
| Peptostreptococcus | 0.0689 | 0.013 | 0.125 | 0.016 | validation |

**Table S4.** Characteristic genera validated by logistic regression for group 3 trajectory in males in both discovery dataset and validation dataset.

| Variable | Coefficient | Ci_lower | Ci_upper | Fdr_p | Data |
| --- | --- | --- | --- | --- | --- |
| CHKCI002 | -0.069 | -0.115 | -0.024 | 0.003 | discovery |
| Turicibacter | -0.053 | -0.095 | -0.014 | 0.010 | discovery |
| Clostridium_sensu_stricto_1 | -0.097 | -0.156 | -0.041 | 0.001 | discovery |
| Lachnospiraceae_NK4A136_group | -0.051 | -0.084 | -0.019 | 0.002 | discovery |
| Sellimonas | -0.057 | -0.112 | -0.001 | 0.046 | discovery |
| f__Oscillospiraceae | -0.071 | -0.112 | -0.030 | 0.001 | discovery |
| f__Ruminococcaceae | -0.077 | -0.115 | -0.040 | 0.000 | discovery |
| Terrisporobacter | -0.040 | -0.075 | -0.006 | 0.023 | discovery |
| CHKCI002 | -0.095 | -0.155 | -0.038 | 0.001 | validation |
| Turicibacter | -0.120 | -0.181 | -0.064 | 0.000 | validation |
| Clostridium_sensu_stricto_1 | -0.238 | -0.339 | -0.144 | 0.000 | validation |
| Lachnospiraceae_NK4A136_group | -0.055 | -0.105 | -0.006 | 0.027 | validation |
| Sellimonas | -0.061 | -0.110 | -0.013 | 0.014 | validation |
| f__Oscillospiraceae | -0.072 | -0.144 | -0.002 | 0.046 | validation |
| f__Ruminococcaceae | -0.057 | -0.114 | -0.002 | 0.044 | validation |
| Terrisporobacter | -0.116 | -0.175 | -0.060 | 0.000 | validation |

**Table S5.** Characteristic genera validated by logistic regression for group 4 trajectory in males in both discovery dataset and validation dataset.

| Variable | Coefficient | Ci_lower | Ci_upper | Fdr_p | Data |
| --- | --- | --- | --- | --- | --- |
| CHKCI002 | -0.097 | -0.172 | -0.031 | 0.007 | discovery |
| Turicibacter | -0.103 | -0.152 | -0.054 | 0.000 | discovery |
| Clostridium_sensu_stricto_1 | -0.127 | -0.196 | -0.060 | 0.000 | discovery |
| Romboutsia | -0.153 | -0.237 | -0.074 | 0.000 | discovery |
| CHKCI002 | -0.196 | -0.331 | -0.094 | 0.001 | validation |
| Turicibacter | -0.117 | -0.191 | -0.046 | 0.001 | validation |
| Clostridium_sensu_stricto_1 | -0.283 | -0.407 | -0.170 | 0.000 | validation |
| Romboutsia | -0.080 | -0.145 | -0.018 | 0.013 | validation |

**Table S6.** Characteristic genera validated by logistic regression for group 2 trajectory in females in both discovery dataset and validation dataset.

| Variable | Coefficient | Ci_lower | Ci_upper | Fdr_p | Data |
| --- | --- | --- | --- | --- | --- |
| Parabacteroides | -0.044 | -0.077 | -0.011 | 0.009 | discovery |
| [Eubacterium]_brachy_group | -0.041 | -0.075 | -0.006 | 0.021 | discovery |
| Parabacteroides | -0.065 | -0.111 | -0.020 | 0.005 | validation |
| [Eubacterium]_brachy_group | -0.065 | -0.110 | -0.020 | 0.005 | validation |

**Table S7.** 839 Metabolites comparisions between group 2 trajectory and group 1 trajectory in males.

| Compounds | P_value | P_adjusted | FC | log2FC |
| --- | --- | --- | --- | --- |
| 3-carboxy-4-methyl-5-propyl-2-furanpropionic acid | 0.398 | 0.701 | 1.111 | 0.152 |
| 2,4-diacetamino-2,4,6-triphenoxy-D-mannopyranose | 0.022 | 0.230 | 1.365 | 0.449 |
| L-Threonine | 0.944 | 0.984 | 1.001 | 0.001 |
| L-Arginine | 0.125 | 0.441 | 1.051 | 0.072 |
| L-Aspartic Acid | 0.729 | 0.929 | 1.027 | 0.038 |
| L-Citrulline | 0.016 | 0.215 | 0.884 | -0.177 |
| L-Glutamic Acid | 0.000 | 0.101 | 1.251 | 0.323 |
| L-Phenylalanine | 0.123 | 0.440 | 1.031 | 0.044 |
| L-Serine | 0.131 | 0.447 | 0.937 | -0.094 |
| L-Tryptophan | 0.785 | 0.933 | 1.018 | 0.026 |
| (5-L-Glutamyl)-L-Amino Acid | 0.100 | 0.403 | 1.172 | 0.229 |
| Allantoin | 0.186 | 0.523 | 1.377 | 0.462 |
| Asp-Phe | 0.546 | 0.819 | 1.162 | 0.216 |
| Glutathione Oxidized | 0.745 | 0.930 | 1.020 | 0.029 |
| Hexanoyl Glycine | 0.179 | 0.510 | 0.901 | -0.150 |
| L-Asparagine Anhydrous | 0.948 | 0.984 | 1.005 | 0.007 |
| L-Glutamine | 0.002 | 0.132 | 0.859 | -0.219 |
| L-Homocitrulline | 0.557 | 0.827 | 1.072 | 0.101 |
| L-Theanine | 0.604 | 0.858 | 0.989 | -0.016 |
| N-Acetylaspartate | 0.335 | 0.660 | 0.971 | -0.042 |
| N-Acetyl-L-Leucine | 0.013 | 0.210 | 1.224 | 0.291 |
| N-Acetyl-L-Tyrosine | 0.787 | 0.933 | 1.000 | 0.000 |
| N-Acetylneuraminic Acid(SA) | 0.021 | 0.230 | 0.846 | -0.241 |
| Gly-Leu | 0.178 | 0.509 | 1.263 | 0.337 |
| N-Isovaleroylglycine | 0.253 | 0.595 | 1.237 | 0.307 |
| N-Propionylglycine | 0.022 | 0.230 | 1.069 | 0.096 |
| Nα-Acetyl-L-Arginine | 0.155 | 0.488 | 1.182 | 0.241 |
| O-Phospho-L-Serine | 0.752 | 0.931 | 0.971 | -0.043 |
| Phenylacetyl-L-Glutamine | 0.991 | 0.995 | 1.051 | 0.071 |
| Phe-Phe | 0.021 | 0.230 | 1.135 | 0.182 |
| S-(5-Adenosy)-L-Homocysteine | 0.340 | 0.663 | 0.936 | -0.096 |
| S-Sulfo-L-Cysteine | 0.291 | 0.635 | 0.775 | -0.367 |
| Trans-4-Hydroxy-L-Proline | 0.022 | 0.230 | 1.069 | 0.096 |
| γ-L-Glutamate-Cysteine | 0.400 | 0.701 | 0.988 | -0.018 |
| N-Acetyl-L-phenylalanine | 0.047 | 0.305 | 1.088 | 0.122 |
| Benzoylformic Acid | 0.099 | 0.401 | 0.910 | -0.136 |
| 3-Hydroxyanthranilic Acid | 0.208 | 0.541 | 1.031 | 0.043 |
| P–Hydroxyphenyl Acetic Acid | 0.957 | 0.984 | 1.047 | 0.067 |
| 2-Picolinic Acid | 0.499 | 0.787 | 0.989 | -0.016 |
| 4-Pyridoxic Acid | 0.138 | 0.459 | 1.083 | 0.115 |
| 6-Hydroxynicotinic Acid | 0.028 | 0.249 | 1.302 | 0.381 |
| Taurocholic acid | 0.855 | 0.971 | 1.070 | 0.097 |
| Taurochenodesoxycholic Acid | 0.546 | 0.819 | 0.864 | -0.211 |
| Glycolithocholic acid | 0.351 | 0.669 | 0.987 | -0.019 |
| Hyodeoxycholic acid | 0.734 | 0.930 | 0.882 | -0.181 |
| Glycoursodeoxycholic Acid | 0.955 | 0.984 | 0.988 | -0.017 |
| Glycochenodeoxycholic Acid | 0.778 | 0.933 | 0.862 | -0.214 |
| Chenodeoxycholic Acid | 0.562 | 0.827 | 1.274 | 0.350 |
| 4-Methylcatechol | 0.750 | 0.930 | 0.903 | -0.147 |
| 4-Hydroxy-3-methoxybenzaldehyde | 0.583 | 0.849 | 0.967 | -0.048 |
| 1,7-Dimethylxanthine | 0.944 | 0.984 | 0.512 | -0.966 |
| 1-Methylxanthine | 0.565 | 0.827 | 0.838 | -0.255 |
| Xanthine | 0.148 | 0.483 | 1.129 | 0.175 |
| 3-Methylxanthine | 0.565 | 0.827 | 0.838 | -0.255 |
| 5-Methylcytosine | 0.386 | 0.694 | 0.929 | -0.107 |
| 7-Methylxanthine | 0.565 | 0.827 | 0.838 | -0.255 |
| Guanosine | 0.001 | 0.109 | 2.472 | 1.305 |
| Hypoxanthine | 0.740 | 0.930 | 0.934 | -0.098 |
| Uridine | 0.427 | 0.725 | 0.954 | -0.068 |
| L-Thyroxine | 0.162 | 0.495 | 0.932 | -0.102 |
| Norepinephrine | 0.373 | 0.690 | 1.048 | 0.067 |
| Succinic Acid | 0.346 | 0.666 | 1.029 | 0.041 |
| Cis-Aconitic Acid | 0.394 | 0.698 | 1.055 | 0.078 |
| Melatonin | 0.330 | 0.657 | 1.012 | 0.017 |
| Tryptamine | 0.629 | 0.876 | 1.006 | 0.009 |
| D-Glucose | 0.299 | 0.638 | 1.038 | 0.054 |
| D-Trehalose | 0.522 | 0.804 | 0.979 | -0.031 |
| D-Glucose 6-Phosphate | 0.129 | 0.441 | 1.088 | 0.122 |
| Lactose | 0.522 | 0.804 | 0.979 | -0.031 |
| Lactulose | 0.522 | 0.804 | 0.979 | -0.031 |
| L-Fucose | 0.009 | 0.210 | 0.719 | -0.476 |
| Maltose | 0.522 | 0.804 | 0.979 | -0.031 |
| D-Glucoronic Acid | 0.191 | 0.529 | 1.078 | 0.109 |
| Pantothenate | 0.299 | 0.638 | 1.046 | 0.064 |
| 3-Indolepropionic Acid | 0.844 | 0.966 | 1.054 | 0.076 |
| Indole-3-Carboxaldehyde | 0.706 | 0.924 | 0.948 | -0.077 |
| Methyl Indole-3-Acetate | 0.085 | 0.384 | 1.137 | 0.185 |
| 2-Hydroxybutanoic Acid | 0.008 | 0.210 | 0.643 | -0.637 |
| 2-Hydroxyisocaproic Acid | 0.967 | 0.984 | 1.004 | 0.005 |
| 2-Methylsuccinic Acid | 0.224 | 0.554 | 1.144 | 0.194 |
| 3-Hydroxy-3-Methyl Butyric Acid | 0.376 | 0.693 | 0.948 | -0.078 |
| 3-Methylcrotonyl Glycine | 0.904 | 0.981 | 1.002 | 0.002 |
| 4-Hydroxy-2-Oxoglutaric Acid | 0.078 | 0.374 | 1.144 | 0.194 |
| Adipic Acid | 0.034 | 0.278 | 0.905 | -0.144 |
| Azelaic Acid | 0.967 | 0.984 | 0.971 | -0.043 |
| Caffeic Acid | 0.993 | 0.996 | 0.873 | -0.196 |
| Creatine | 0.365 | 0.681 | 1.137 | 0.185 |
| Dodecanedioic Aicd | 0.004 | 0.168 | 0.889 | -0.170 |
| Glutaric Acid | 0.224 | 0.554 | 1.144 | 0.194 |
| Guanidinoethyl Sulfonate | 0.535 | 0.810 | 0.984 | -0.023 |
| Hippuric Acid | 0.944 | 0.984 | 0.849 | -0.236 |
| Hydrocinnamic Acid | 0.307 | 0.640 | 0.906 | -0.143 |
| L-kynurenine | 0.435 | 0.736 | 0.981 | -0.027 |
| Kynurenic Acid | 0.061 | 0.336 | 1.085 | 0.118 |
| L-Lactic Acid | 0.252 | 0.595 | 0.984 | -0.023 |
| Malonicacid | 0.008 | 0.210 | 0.658 | -0.604 |
| Mandelic Acid | 0.957 | 0.984 | 1.047 | 0.067 |
| Methylmalonic Acid | 0.346 | 0.666 | 1.029 | 0.041 |
| Phenyllactate(Pla) | 0.563 | 0.827 | 0.940 | -0.090 |
| Pyrrole-2-Carboxylic Acid | 0.594 | 0.855 | 0.973 | -0.039 |
| Sebacate | 0.045 | 0.298 | 0.893 | -0.164 |
| Shikimic Acid | 0.277 | 0.619 | 1.139 | 0.187 |
| Subericacid | 0.327 | 0.657 | 0.953 | -0.069 |
| TXB2 | 0.978 | 0.986 | 1.452 | 0.538 |
| (±)15-HETE | 0.096 | 0.399 | 1.280 | 0.357 |
| LPG(18:1/0:0) | 0.461 | 0.761 | 0.966 | -0.050 |
| LPE(18:1/0:0) | 0.863 | 0.972 | 0.992 | -0.012 |
| LPE(18:0/0:0) | 0.157 | 0.488 | 1.055 | 0.078 |
| LPE(16:0/0:0) | 0.045 | 0.298 | 1.127 | 0.172 |
| LPE(14:0/0:0) | 0.002 | 0.132 | 1.184 | 0.244 |
| LPA(0:0/18:0) | 0.959 | 0.984 | 0.996 | -0.006 |
| LPA(0:0/16:0) | 0.227 | 0.555 | 1.007 | 0.010 |
| LipoxinA4 | 0.085 | 0.384 | 1.113 | 0.154 |
| 13-HOTrE | 0.010 | 0.210 | 1.520 | 0.604 |
| 9,10-DiHOME | 0.015 | 0.210 | 0.808 | -0.307 |
| FFA(18:3) | 0.324 | 0.657 | 1.064 | 0.089 |
| FFA(16:0) | 0.201 | 0.541 | 0.976 | -0.034 |
| FFA(18:2) | 0.010 | 0.210 | 0.921 | -0.119 |
| FFA(12:0) | 0.147 | 0.482 | 0.934 | -0.098 |
| FFA(18:1) | 0.023 | 0.241 | 0.887 | -0.174 |
| EPA | 0.307 | 0.640 | 1.057 | 0.080 |
| FFA(20:2) | 0.021 | 0.230 | 0.856 | -0.224 |
| FFA(10:0) | 0.154 | 0.488 | 0.208 | -2.268 |
| AA | 0.204 | 0.541 | 0.932 | -0.102 |
| Urocanic Acid | 0.688 | 0.912 | 0.989 | -0.016 |
| 4-Hydroxybenzaldehyde | 0.498 | 0.787 | 1.024 | 0.035 |
| Neopterin | 0.502 | 0.788 | 0.999 | -0.002 |
| Ethylmalonate | 0.624 | 0.875 | 1.088 | 0.122 |
| 2-(Formylamino)Benzoic Acid | 0.207 | 0.541 | 1.099 | 0.136 |
| Ureidoisobutyric Acid | 0.982 | 0.989 | 0.965 | -0.052 |
| Uridine 5-Monophosphate | 0.136 | 0.455 | 1.117 | 0.160 |
| N-Acetylglycine | 0.041 | 0.296 | 0.845 | -0.243 |
| 3-Hydroxyhippuric Acid | 0.050 | 0.305 | 1.751 | 0.808 |
| 2-(Dimethylamino)Guanosine | 0.816 | 0.943 | 1.003 | 0.004 |
| 5-Hydroxyhexanoic Acid | 0.967 | 0.984 | 1.004 | 0.005 |
| Β-Pseudouridine | 0.573 | 0.836 | 1.068 | 0.095 |
| N-Acetylthreonine | 0.342 | 0.666 | 0.976 | -0.035 |
| 3,4,5-Trimethoxybenzoic Acid | 0.718 | 0.926 | 0.869 | -0.202 |
| Hypoxanthine-9-β-D-Arabinofuranoside | 0.006 | 0.192 | 2.194 | 1.134 |
| D-Sedoheptuiose 7-Phosphate | 0.570 | 0.833 | 0.978 | -0.032 |
| D-Fructose 6-Phosphate-Disodium Salt | 0.129 | 0.441 | 1.088 | 0.122 |
| Aminomalonic Acid | 0.672 | 0.900 | 1.001 | 0.002 |
| 8,15-Dihete | 0.381 | 0.694 | 0.872 | -0.197 |
| N-Acetyl-L-methionine | 0.352 | 0.670 | 0.961 | -0.058 |
| Argininosuccinic acid | 0.002 | 0.132 | 1.233 | 0.302 |
| 2-Deoxyribose 1-Phosphate | 0.555 | 0.826 | 0.933 | -0.100 |
| N-Acetylglucosamine 1-Phosphate | 0.330 | 0.657 | 1.036 | 0.050 |
| Jasmonic acid | 0.803 | 0.936 | 0.990 | -0.014 |
| Indole-3-lactic acid | 0.972 | 0.984 | 1.027 | 0.038 |
| (3-Methoxy-4-hydroxyphenyl)ethylene glycol sulfate | 0.037 | 0.288 | 0.872 | -0.198 |
| Xanthosine | 0.110 | 0.410 | 0.942 | -0.086 |
| estrone 3-sulfate | 0.861 | 0.971 | 0.990 | -0.014 |
| 1-Methylguanine | 0.489 | 0.780 | 1.056 | 0.079 |
| DL-3,4-Dihydroxyphenyl glycol | 0.522 | 0.804 | 1.166 | 0.222 |
| dihydrotachysterol | 0.722 | 0.927 | 0.965 | -0.052 |
| Indoleacrylic acid | 0.868 | 0.974 | 1.082 | 0.114 |
| 2-(4-Hydroxyphenyl)ethanol | 0.688 | 0.912 | 1.008 | 0.012 |
| Hydroxyphenyllactic acid | 0.444 | 0.743 | 0.976 | -0.035 |
| Indole 3-carbinol | 0.635 | 0.876 | 1.007 | 0.010 |
| 2-Methylguanosine | 0.328 | 0.657 | 0.949 | -0.076 |
| 1,2,3-Trihydroxybenzene | 0.740 | 0.930 | 1.078 | 0.109 |
| N-lactoyl-phenylalanine | 0.045 | 0.298 | 1.152 | 0.204 |
| N-Acetyl-L-alanine | 0.209 | 0.541 | 0.966 | -0.050 |
| Cyclamic acid | 0.969 | 0.984 | 1.165 | 0.220 |
| D-Malic acid | 0.604 | 0.858 | 1.024 | 0.034 |
| Tetradecanedioic acid | 0.070 | 0.353 | 0.878 | -0.188 |
| Uridine triphosphate(UTP) | 0.743 | 0.930 | 1.106 | 0.145 |
| 6β-hydroxytestosterone | 0.190 | 0.529 | 0.974 | -0.037 |
| O-Acetyl-L-serine | 0.899 | 0.980 | 0.990 | -0.014 |
| Indoxylsulfuric acid | 0.410 | 0.707 | 1.074 | 0.102 |
| Porphobilinogen | 0.725 | 0.928 | 1.069 | 0.096 |
| Hydroquinone | 0.385 | 0.694 | 1.101 | 0.139 |
| Anthranilic acid | 0.165 | 0.497 | 1.164 | 0.219 |
| Indoleacetaldehyde | 0.315 | 0.647 | 1.004 | 0.006 |
| Hexadecanedioic acid | 0.013 | 0.210 | 0.877 | -0.190 |
| Pyrophosphate | 0.890 | 0.978 | 1.015 | 0.021 |
| 2-hydroxy-2-(4-hydroxy-3-methoxyphenyl)acetic acid | 0.853 | 0.971 | 1.032 | 0.045 |
| Glu-Leu | 0.006 | 0.192 | 1.114 | 0.156 |
| 5-oxoETE | 0.092 | 0.399 | 1.196 | 0.259 |
| Ethylsalicylate | 0.770 | 0.933 | 1.182 | 0.242 |
| Octadecanamide | 0.720 | 0.926 | 1.077 | 0.107 |
| Undecanedioic acid | 0.660 | 0.889 | 0.907 | -0.140 |
| Oxaloacetic acid | 0.933 | 0.984 | 0.970 | -0.045 |
| Phenoxyacetic acid | 0.486 | 0.780 | 0.779 | -0.360 |
| 4-Hydroxybenzyl alcohol | 0.704 | 0.924 | 0.940 | -0.089 |
| Methanesulfonic acid | 0.518 | 0.804 | 0.998 | -0.003 |
| Propylparaben | 0.222 | 0.554 | 0.942 | -0.087 |
| Butylparaben | 0.559 | 0.827 | 1.145 | 0.196 |
| Methylparaben | 0.255 | 0.595 | 0.951 | -0.072 |
| 44986 | 0.107 | 0.410 | 1.250 | 0.321 |
| (±)12-HEPE | 0.044 | 0.298 | 1.665 | 0.736 |
| (±)12-HETE | 0.306 | 0.640 | 1.163 | 0.218 |
| (±)15-HEPE | 0.044 | 0.298 | 1.665 | 0.736 |
| (±)17-HDHA | 0.044 | 0.298 | 1.248 | 0.320 |
| (±)18-HEPE | 0.044 | 0.298 | 1.665 | 0.736 |
| (±)4-HDHA | 0.027 | 0.249 | 1.157 | 0.211 |
| (±)5-HEPE | 0.016 | 0.215 | 1.518 | 0.602 |
| (±)5-HETE | 0.071 | 0.353 | 1.217 | 0.283 |
| (±)9-HETE | 0.071 | 0.353 | 1.217 | 0.283 |
| 11,12-EET | 0.051 | 0.305 | 1.294 | 0.372 |
| 13-oxoODE | 0.055 | 0.316 | 1.680 | 0.748 |
| 14(S)-HDHA | 0.155 | 0.488 | 1.150 | 0.201 |
| 15-oxoETE | 0.011 | 0.210 | 1.568 | 0.649 |
| 5(S),15(S)-DiHETE | 0.065 | 0.343 | 1.445 | 0.531 |
| 5,6-DiHETrE | 0.076 | 0.371 | 1.163 | 0.218 |
| 5-HETrE | 0.015 | 0.210 | 1.556 | 0.638 |
| 9-oxoODE | 0.055 | 0.316 | 1.680 | 0.748 |
| LTB4 | 0.039 | 0.293 | 1.506 | 0.591 |
| PDX | 0.107 | 0.410 | 1.250 | 0.321 |
| Prostaglandin E2 | 0.050 | 0.305 | 1.349 | 0.431 |
| RvD5 | 0.107 | 0.410 | 1.250 | 0.321 |
| Nα-Acetyl-L-glutamine | 0.412 | 0.707 | 0.990 | -0.015 |
| FFA(14:0) | 0.734 | 0.930 | 0.951 | -0.072 |
| 4-Hydroxyhippurate | 0.222 | 0.554 | 1.230 | 0.299 |
| 3-Hydroxyglutaric acid | 0.822 | 0.948 | 0.956 | -0.064 |
| 3-(3-Hydroxyphenyl)-3-hydroxypropanoic acid | 0.025 | 0.244 | 1.312 | 0.392 |
| N-acetylornithine | 0.037 | 0.288 | 1.060 | 0.084 |
| N-Alpha-Acetyl-L-Asparagine | 0.267 | 0.614 | 0.953 | -0.070 |
| N-Amidino-L-Aspartate | 0.219 | 0.554 | 1.063 | 0.088 |
| Lumichrome | 0.084 | 0.384 | 0.938 | -0.093 |
| 3-Amino-4-Hydroxybenzoic Acid | 0.208 | 0.541 | 1.031 | 0.043 |
| 2',4'-Dihydroxyacetophenone | 0.255 | 0.595 | 0.951 | -0.072 |
| Inosine | 0.005 | 0.192 | 2.117 | 1.082 |
| L-Sepiapterin | 0.006 | 0.198 | 1.145 | 0.195 |
| Phosphoenolpyruvate | 0.953 | 0.984 | 1.030 | 0.043 |
| Uric acid | 0.740 | 0.930 | 1.093 | 0.128 |
| 3-hydroxyphenylacetic acid | 0.974 | 0.985 | 1.075 | 0.104 |
| 7-Methylguanine | 0.489 | 0.780 | 1.056 | 0.079 |
| Nicotinic Acid | 0.749 | 0.930 | 1.007 | 0.010 |
| 3-(4-Hydroxyphenyl)-Propionic Acid | 0.455 | 0.754 | 0.986 | -0.021 |
| 5,6-Dimethylbenzimidazole | 0.187 | 0.525 | 1.012 | 0.017 |
| Gly-Phe | 0.798 | 0.936 | 1.029 | 0.041 |
| 13(R)-HODE | 0.159 | 0.489 | 1.353 | 0.437 |
| p-Cresol | 0.878 | 0.977 | 1.073 | 0.102 |
| Indole-4-carboxaldehyde | 0.706 | 0.924 | 0.948 | -0.077 |
| 9(S)-HpOTrE | 0.002 | 0.132 | 0.739 | -0.436 |
| Iminodiacetic acid | 0.495 | 0.783 | 1.052 | 0.073 |
| 2-Methyl-d-erythritol 2,4-cyclodiphosphate | 0.774 | 0.933 | 1.019 | 0.028 |
| Tauroursodeoxycholic acid | 0.373 | 0.690 | 1.034 | 0.048 |
| Gly-Val | 0.745 | 0.930 | 0.955 | -0.067 |
| FFA(18:4) | 0.228 | 0.555 | 1.190 | 0.250 |
| alpha-Muricholic acid | 0.967 | 0.984 | 1.050 | 0.070 |
| 12,13-DiHOME | 0.018 | 0.223 | 0.790 | -0.340 |
| Sphingosine 1-phosphate | 0.409 | 0.707 | 0.877 | -0.189 |
| 4-acetoxyphenol | 0.551 | 0.822 | 1.016 | 0.023 |
| 2-ethyl-2-hydroxybutyric acid | 0.758 | 0.933 | 1.021 | 0.030 |
| 7-ketolithocholic acid | 0.483 | 0.780 | 1.155 | 0.208 |
| 1,6-anhydro-β-D-glucose | 0.936 | 0.984 | 1.014 | 0.019 |
| 16-Hydroxyhexadecanoic acid | 0.014 | 0.210 | 0.912 | -0.133 |
| 12-ketolithocholic acid | 0.483 | 0.780 | 1.155 | 0.208 |
| N,N′-dicyclohexylcarbodiimide | 0.085 | 0.384 | 0.940 | -0.090 |
| Gamma-Mercholic Acid | 0.967 | 0.984 | 1.050 | 0.070 |
| Apocholic acid | 0.483 | 0.780 | 1.155 | 0.208 |
| 2-hydroxyhexadecanoic acid | 0.014 | 0.210 | 0.912 | -0.133 |
| Indole-3-carboxylic acid | 0.840 | 0.965 | 1.004 | 0.005 |
| 4-Methyl-2-oxovaleric acid | 0.227 | 0.555 | 0.944 | -0.083 |
| FFA(20:4) | 0.365 | 0.681 | 0.971 | -0.042 |
| Quinolinic acid | 0.885 | 0.978 | 0.977 | -0.033 |
| p-Tolyl Sulfate | 0.543 | 0.817 | 0.926 | -0.111 |
| 5-nitrobenzimidazole | 0.929 | 0.984 | 0.998 | -0.004 |
| Lythramine | 0.438 | 0.739 | 1.087 | 0.121 |
| Acetaminophen | 0.302 | 0.640 | 1.051 | 0.072 |
| 3-Sulfocatechol | 0.314 | 0.647 | 1.106 | 0.146 |
| 6-hydroxy-3-succinylpyridine | 0.222 | 0.554 | 1.230 | 0.299 |
| 2-(4-hydroxyphenyl) propionate | 0.394 | 0.698 | 1.243 | 0.314 |
| D-Mannose 6-phosphate | 0.129 | 0.441 | 1.088 | 0.122 |
| 2-amino-4-oxovaleric acid | 0.209 | 0.541 | 0.966 | -0.050 |
| 1-O-vanillyl-β-D-glucose | 0.767 | 0.933 | 1.012 | 0.017 |
| L-2-amino-6-oximelic acid | 0.149 | 0.483 | 1.106 | 0.146 |
| 3-(pyrazol-1-yl)-L-alanine | 0.192 | 0.530 | 1.031 | 0.044 |
| 1-pyrroline-4-hydroxy-2-carboxylate | 0.184 | 0.520 | 0.938 | -0.093 |
| LPE(0:0/22:4) | 0.604 | 0.858 | 1.091 | 0.126 |
| LPE(22:4/0:0) | 0.604 | 0.858 | 1.091 | 0.126 |
| LPE(0:0/22:5) | 0.813 | 0.940 | 0.990 | -0.014 |
| LPE(22:5/0:0) | 0.813 | 0.940 | 0.990 | -0.014 |
| LPE(0:0/22:6) | 0.074 | 0.366 | 1.134 | 0.182 |
| LPE(22:6/0:0) | 0.074 | 0.366 | 1.134 | 0.182 |
| LPE(0:0/20:2) | 0.501 | 0.787 | 0.894 | -0.162 |
| LPE(20:2/0:0) | 0.501 | 0.787 | 0.894 | -0.162 |
| LPE(0:0/20:3) | 0.051 | 0.305 | 1.055 | 0.077 |
| LPE(20:3/0:0) | 0.051 | 0.305 | 1.055 | 0.077 |
| LPE(20:4/0:0) | 0.658 | 0.889 | 1.005 | 0.008 |
| LPE(0:0/20:5) | 0.063 | 0.336 | 1.159 | 0.213 |
| LPE(20:5/0:0) | 0.063 | 0.336 | 1.159 | 0.213 |
| LPE(0:0/18:0) | 0.157 | 0.488 | 1.055 | 0.078 |
| LPE(0:0/18:2) | 0.489 | 0.780 | 1.057 | 0.079 |
| LPE(0:0/16:0) | 0.045 | 0.298 | 1.127 | 0.172 |
| LPE(0:0/16:1) | 0.121 | 0.434 | 1.131 | 0.178 |
| 1-Aminocyclohexanoic acid | 0.718 | 0.926 | 0.972 | -0.042 |
| Ureidosuccinic acid | 0.912 | 0.984 | 0.970 | -0.043 |
| 2-Hydroxycaprylic acid | 0.086 | 0.384 | 0.870 | -0.201 |
| 2-hydroxyphenylacetic acid | 0.525 | 0.804 | 1.025 | 0.036 |
| 4-Hydroxy-3-methylbenzoic acid | 0.525 | 0.804 | 1.025 | 0.036 |
| 2-Hydroxy-2-Methyl Butyric acid | 0.770 | 0.933 | 1.038 | 0.054 |
| 2-Octenoic acid | 0.128 | 0.441 | 1.065 | 0.091 |
| 2-Methylglutaric Acid | 0.042 | 0.298 | 0.919 | -0.121 |
| (S)-Leucic acid | 0.967 | 0.984 | 1.004 | 0.005 |
| Glycohyodeoxycholic acid | 0.990 | 0.995 | 0.963 | -0.054 |
| N-Cinnamylglycine | 0.058 | 0.323 | 0.623 | -0.682 |
| (R)-(-)-2-Phenylpropionic Acid | 0.307 | 0.640 | 0.906 | -0.143 |
| 8-Aminooctanoic Acid | 0.593 | 0.855 | 0.999 | -0.001 |
| 4-Methoxysalicylic Acid | 0.176 | 0.509 | 1.576 | 0.657 |
| Tridecanedioic acid | 0.978 | 0.986 | 1.006 | 0.008 |
| 12-Hydroxyoctadecanoic acid | 0.995 | 0.996 | 0.997 | -0.004 |
| N-Palmitoylglycine | 0.013 | 0.210 | 0.956 | -0.065 |
| Taurolithocholic acid | 0.779 | 0.933 | 0.957 | -0.063 |
| 13(S)-HOTrE(γ) | 0.015 | 0.210 | 1.504 | 0.589 |
| 9(S)-HOTrE | 0.012 | 0.210 | 1.367 | 0.451 |
| (±)8-HETE | 0.061 | 0.336 | 1.193 | 0.254 |
| 8(S)-HETrE | 0.015 | 0.210 | 1.556 | 0.638 |
| 15(S)-HETrE | 0.015 | 0.210 | 1.556 | 0.638 |
| 9(S),12(S),13(S)-TriHOME | 0.240 | 0.571 | 1.113 | 0.154 |
| Bicyclo Prostaglandin E2 | 0.124 | 0.441 | 1.300 | 0.379 |
| 20-COOH-AA | 0.690 | 0.913 | 1.146 | 0.197 |
| Prostaglandin B2 | 0.112 | 0.413 | 1.286 | 0.363 |
| 6-trans-12-epi Leukotriene B4 | 0.039 | 0.293 | 1.506 | 0.591 |
| 6-trans Leukotriene B4 | 0.039 | 0.293 | 1.506 | 0.591 |
| 13-HDoHE | 0.027 | 0.249 | 1.276 | 0.352 |
| 10-HDoHE | 0.045 | 0.298 | 1.225 | 0.293 |
| 8-HDoHE | 0.108 | 0.410 | 1.227 | 0.295 |
| 11-HDoHE | 0.048 | 0.305 | 1.354 | 0.437 |
| 16-HDoHE | 0.044 | 0.298 | 1.262 | 0.336 |
| 20-HDoHE | 0.077 | 0.374 | 1.308 | 0.387 |
| 11-HEDE | 0.170 | 0.503 | 1.243 | 0.314 |
| 15-HEDE | 0.170 | 0.503 | 1.243 | 0.314 |
| (±)19(20)-EpDPE(A) | 0.034 | 0.276 | 1.180 | 0.238 |
| 11β-Prostaglandin E2 | 0.050 | 0.305 | 1.349 | 0.431 |
| Glu-Gln | 0.206 | 0.541 | 0.800 | -0.322 |
| Ethionamide | 0.833 | 0.957 | 1.154 | 0.206 |
| Glycerophospho-N-Arachidonoyl Ethanolamine | 0.658 | 0.889 | 1.005 | 0.008 |
| Testosterone sulfate | 0.608 | 0.860 | 1.024 | 0.035 |
| FFA(16:2) | 0.236 | 0.564 | 1.310 | 0.389 |
| (R)-(-)-Mandelic acid | 0.711 | 0.926 | 1.133 | 0.180 |
| (R)-3-Hydroxybutanoic acid | 0.008 | 0.210 | 0.643 | -0.637 |
| 2-Hydroxyhexanoic acid | 0.967 | 0.984 | 1.004 | 0.005 |
| 2'-O-methyluridine | 0.333 | 0.657 | 0.940 | -0.089 |
| 2-Phenylbutyric acid | 0.723 | 0.927 | 0.998 | -0.003 |
| 3-Hydroxycinnamic acid | 0.813 | 0.940 | 0.996 | -0.005 |
| 3-Methyluridine | 0.790 | 0.933 | 1.024 | 0.035 |
| 3-Phenoxybenzoic acid | 0.594 | 0.855 | 0.950 | -0.073 |
| 4-Ethyloctanoic acid | 0.961 | 0.984 | 0.998 | -0.003 |
| 4-Methoxyphenol | 0.750 | 0.930 | 0.903 | -0.147 |
| 5-Hydroxy-2'-deoxyuridine | 0.591 | 0.855 | 1.018 | 0.026 |
| 6-Hydroxyflavone (6-HF) | 0.241 | 0.572 | 1.085 | 0.118 |
| Acetylvaline | 0.089 | 0.387 | 1.111 | 0.152 |
| D-Galacturonic Acid | 0.027 | 0.249 | 1.120 | 0.163 |
| Dimethylmalonic acid | 0.224 | 0.554 | 1.144 | 0.194 |
| D-Tagatose | 0.299 | 0.638 | 1.038 | 0.054 |
| Val-Ala | 0.927 | 0.984 | 0.981 | -0.028 |
| Isethionic acid | 0.109 | 0.410 | 0.949 | -0.075 |
| Octadecanedioic acid | 0.007 | 0.198 | 0.831 | -0.266 |
| Sucrose 6′-monophosphate | 0.155 | 0.488 | 1.094 | 0.130 |
| Traumatic acid | 0.006 | 0.198 | 0.851 | -0.233 |
| Phosphatidylethanolamine lyso alkenyl 16:0 | 0.732 | 0.930 | 1.015 | 0.022 |
| LPA(16:0/0:0) | 0.333 | 0.657 | 1.024 | 0.034 |
| 3-Hydroxy-tetradecanoic acid | 0.056 | 0.316 | 0.867 | -0.206 |
| Acetanilide | 0.803 | 0.936 | 1.005 | 0.007 |
| FFA(22:4) | 0.041 | 0.296 | 0.815 | -0.295 |
| Cytochalasin H | 0.217 | 0.554 | 0.971 | -0.042 |
| Glu-Val | 0.011 | 0.210 | 1.087 | 0.121 |
| Isocitric acid | 0.025 | 0.244 | 1.084 | 0.116 |
| N-Acetyl-5-aminosalicylic acid | 0.088 | 0.387 | 1.397 | 0.482 |
| 2,4-Quinolinediol | 0.480 | 0.780 | 1.003 | 0.005 |
| Glu-Thr | 0.370 | 0.688 | 1.033 | 0.047 |
| Glu-Tyr | 0.293 | 0.638 | 1.075 | 0.105 |
| LPA(18:1/0:0) | 0.657 | 0.889 | 0.991 | -0.013 |
| CMPentylF | 0.533 | 0.810 | 0.897 | -0.157 |
| Barbital | 0.925 | 0.984 | 1.005 | 0.007 |
| Androsterone sulfate | 0.781 | 0.933 | 1.018 | 0.025 |
| Hydroxypiperazic acid | 0.005 | 0.175 | 0.846 | -0.241 |
| 2-Naphthalenesulfonic acid | 0.591 | 0.855 | 0.972 | -0.041 |
| 2-Deoxyribose 5'-phosphate | 0.358 | 0.679 | 0.944 | -0.083 |
| 3-(2-Naphthyl)-L-alanine | 0.234 | 0.562 | 1.008 | 0.012 |
| L-threo-3-Methylaspartate | 0.000 | 0.101 | 1.251 | 0.323 |
| FAHFA(8:0/10:0) | 0.347 | 0.667 | 0.953 | -0.069 |
| Leu-Ile | 0.285 | 0.628 | 1.243 | 0.314 |
| Docodiendioicacid | 0.646 | 0.882 | 0.975 | -0.037 |
| His-Ser | 0.017 | 0.215 | 0.632 | -0.662 |
| Phosphatidylethanolamine lyso alkenyl 18:2 | 0.522 | 0.804 | 1.043 | 0.061 |
| 2-Methylhexanoic acid | 0.284 | 0.628 | 1.043 | 0.060 |
| 3-(2-Hydroxyphenyl)propanoic acid | 0.455 | 0.754 | 0.986 | -0.021 |
| Salicyluric acid | 0.358 | 0.679 | 2.860 | 1.516 |
| 2-Octanamidoacetic acid | 0.053 | 0.314 | 0.838 | -0.255 |
| Palatinose | 0.308 | 0.640 | 1.053 | 0.074 |
| Tropine | 0.563 | 0.827 | 0.940 | -0.090 |
| M-toluene acetic acid | 0.306 | 0.640 | 0.911 | -0.134 |
| Piperic acid | 0.831 | 0.957 | 1.190 | 0.251 |
| Cholic acid | 0.967 | 0.984 | 1.050 | 0.070 |
| 7-Nitroindazole | 0.101 | 0.405 | 0.878 | -0.188 |
| 4-Methylhexanoic acid | 0.284 | 0.628 | 1.043 | 0.060 |
| 3-Methoxycatechol | 0.769 | 0.933 | 1.007 | 0.010 |
| 3,4-Dimethylbenzoic acid | 0.296 | 0.638 | 0.954 | -0.068 |
| 2,2-Dimethylpentanoic acid | 0.284 | 0.628 | 1.043 | 0.060 |
| D-Talose | 0.299 | 0.638 | 1.038 | 0.054 |
| D-Allose | 0.299 | 0.638 | 1.038 | 0.054 |
| 3,4-Dimethoxycinnamic acid | 0.876 | 0.977 | 0.970 | -0.044 |
| Naphthofluorescein | 0.177 | 0.509 | 0.890 | -0.169 |
| Zereno | 0.472 | 0.774 | 0.968 | -0.047 |
| FFA(16:1) | 0.155 | 0.488 | 0.970 | -0.044 |
| Dihydrodaidzein | 0.098 | 0.401 | 0.804 | -0.315 |
| 20-Hydroxy Prostaglandin F2α | 0.772 | 0.933 | 0.999 | -0.002 |
| Pinolenic acid | 0.667 | 0.894 | 1.021 | 0.030 |
| 15(R)-17-phenyl trinor prostaglandin F2α | 0.595 | 0.855 | 0.985 | -0.022 |
| O-1821 | 0.803 | 0.936 | 1.062 | 0.087 |
| 9,10-dihydroxystearic acid | 0.444 | 0.743 | 0.974 | -0.038 |
| (R)-3-Hydroxymyristic acid | 0.056 | 0.316 | 0.867 | -0.206 |
| 6,6'-Dihydroxy-5,5'-dimethoxybiphenyl-3,3'-dicarboxylic acid | 0.441 | 0.742 | 1.030 | 0.043 |
| 4-Oxoretinoic acid | 0.380 | 0.694 | 1.015 | 0.022 |
| N-Myristoylglycine | 0.093 | 0.399 | 0.935 | -0.098 |
| Isochodeoxycholic acid | 0.082 | 0.384 | 1.203 | 0.267 |
| Carbocyclic thromboxane A2 | 0.153 | 0.488 | 1.211 | 0.276 |
| Hydroferulic acid | 0.891 | 0.978 | 0.858 | -0.221 |
| (S)-2-Hydroxy-3-phenylpropanoic acid | 0.361 | 0.681 | 1.069 | 0.097 |
| Ethyl hydrogen malonate | 0.224 | 0.554 | 1.144 | 0.194 |
| L-Gulose | 0.327 | 0.657 | 1.028 | 0.039 |
| Deoxycholic acid | 0.633 | 0.876 | 1.158 | 0.212 |
| beta-Muricholic acid | 0.967 | 0.984 | 1.050 | 0.070 |
| Glycine deoxycholic acid | 0.859 | 0.971 | 0.866 | -0.208 |
| 3-Epideoxycholic acid | 0.085 | 0.384 | 1.228 | 0.296 |
| 5-Carboxyvanillic Acid | 0.969 | 0.984 | 1.017 | 0.024 |
| 4-Hydroxybenzoic Acid | 0.655 | 0.889 | 1.004 | 0.006 |
| Cys-Pro | 0.097 | 0.401 | 1.085 | 0.118 |
| Ser-Ala | 0.267 | 0.614 | 1.138 | 0.186 |
| Ala-Glu | 0.104 | 0.409 | 1.222 | 0.289 |
| S-Methyl-L-Cysteine-S-oxide | 0.547 | 0.819 | 0.939 | -0.091 |
| L-lyxose | 0.031 | 0.258 | 1.051 | 0.072 |
| Val-Asn | 0.099 | 0.401 | 0.768 | -0.381 |
| D-ribonate lithium salt | 0.255 | 0.595 | 1.023 | 0.033 |
| γ-Glu-Gln | 0.206 | 0.541 | 0.800 | -0.322 |
| Val-Thr | 0.052 | 0.308 | 1.075 | 0.105 |
| 2-keto-D-gluconic acid | 0.027 | 0.249 | 1.120 | 0.163 |
| Met-Asp | 0.259 | 0.601 | 1.044 | 0.062 |
| Val-Gly | 0.796 | 0.936 | 1.055 | 0.077 |
| Pyroglutamic acid | 0.172 | 0.508 | 0.932 | -0.101 |
| cyclo(gly-glu) | 0.014 | 0.210 | 1.317 | 0.397 |
| α-Hydroxyglutaric Acid (sodium salt) | 0.001 | 0.108 | 1.199 | 0.262 |
| cyclo(glu-glu) | 0.213 | 0.549 | 1.029 | 0.041 |
| Ile-Gly | 0.011 | 0.210 | 1.296 | 0.374 |
| Ile-Val | 0.011 | 0.210 | 1.230 | 0.299 |
| Asp-Leu | 0.078 | 0.376 | 1.174 | 0.232 |
| N-Acetyl-L-Glutamic Acid | 0.633 | 0.876 | 1.012 | 0.017 |
| Trp-Gly | 0.012 | 0.210 | 1.081 | 0.113 |
| 2-Hydroxy-3-Methyl Butanoic Acid | 0.776 | 0.933 | 1.011 | 0.015 |
| Salicylic acid β-D-O-glucuronic acid | 0.365 | 0.681 | 1.104 | 0.143 |
| Homovanillic Acid sulfate (sodium salt) | 0.933 | 0.984 | 1.100 | 0.138 |
| 4-Acetylaminobenzoic acid | 0.944 | 0.984 | 0.849 | -0.236 |
| Trp-Leu | 0.013 | 0.210 | 1.186 | 0.246 |
| 4-toluenesulfonic acid | 0.484 | 0.780 | 0.963 | -0.054 |
| 2,4-Dihydroxy-6-pentylbenzoic acid | 0.158 | 0.489 | 0.890 | -0.169 |
| Daidzein | 0.790 | 0.933 | 1.050 | 0.070 |
| D-Gulonic acid γ-lactone | 0.940 | 0.984 | 0.979 | -0.031 |
| L-Iditol | 0.664 | 0.891 | 1.020 | 0.028 |
| 3-Amino-5-hydroxybenzoic acid | 0.208 | 0.541 | 1.031 | 0.043 |
| 2-Methyllactic acid | 0.686 | 0.912 | 1.014 | 0.020 |
| Imidazole-4-methanol | 0.319 | 0.650 | 1.009 | 0.013 |
| (R)-2-Hydroxybutyric acid | 0.686 | 0.912 | 1.014 | 0.020 |
| 2-Methyl-3-hydroxybutyric acid | 0.787 | 0.933 | 0.995 | -0.007 |
| Pyrazine-2-carboxylic acid | 0.365 | 0.681 | 1.006 | 0.008 |
| (2s)-2-Amino-4-sulfinobutanoic acid | 0.895 | 0.979 | 1.013 | 0.018 |
| Ala-Ser | 0.927 | 0.984 | 0.980 | -0.029 |
| （2S，3R，4R，5R）-2,3,4,5,6-五羟基己醛 | 0.299 | 0.638 | 1.038 | 0.054 |
| L-rhamnonic acid | 0.889 | 0.978 | 0.990 | -0.014 |
| 3-Hydroxy-L-phenylalanine | 0.619 | 0.872 | 0.952 | -0.070 |
| Lys-Gly | 0.606 | 0.859 | 1.005 | 0.007 |
| 2-(Acetylamino)-2-deoxy-A-D-glucopyranose | 0.097 | 0.401 | 1.185 | 0.245 |
| Hyp-Thr | 0.409 | 0.707 | 1.018 | 0.026 |
| γ-Glu-Met | 0.337 | 0.660 | 1.141 | 0.190 |
| Lys-Phe | 0.313 | 0.647 | 1.014 | 0.020 |
| Met-Phe | 0.003 | 0.152 | 1.424 | 0.510 |
| Dipyrocetyl | 0.279 | 0.622 | 1.222 | 0.289 |
| 4-(Hydroxyamino)quinoline 1-oxide | 0.789 | 0.933 | 1.000 | 0.000 |
| P-Toluenesulfonamide | 0.180 | 0.512 | 1.329 | 0.411 |
| 4-Hydroxyquinoline | 0.304 | 0.640 | 1.013 | 0.018 |
| 2-Phenyl-5-benzimidazole sulfonic acid | 0.686 | 0.912 | 1.048 | 0.067 |
| 10-Hydroxystearic Acid | 0.223 | 0.554 | 0.951 | -0.073 |
| L-Glycine | 0.465 | 0.765 | 1.015 | 0.021 |
| L-Cystine | 0.876 | 0.977 | 0.919 | -0.122 |
| L-Tyrosine | 0.397 | 0.701 | 1.049 | 0.069 |
| L-Ornithine | 0.348 | 0.667 | 0.954 | -0.068 |
| L-Alanine | 0.037 | 0.286 | 1.113 | 0.155 |
| L-Histidine | 0.075 | 0.367 | 1.060 | 0.083 |
| L-Methionine | 0.177 | 0.509 | 0.900 | -0.152 |
| L-Proline | 0.393 | 0.698 | 1.083 | 0.115 |
| L-Valine | 0.056 | 0.316 | 1.138 | 0.186 |
| 5-Oxoproline | 0.119 | 0.432 | 0.898 | -0.155 |
| Betaine | 0.702 | 0.923 | 0.949 | -0.075 |
| Glyc-Pro | 0.662 | 0.890 | 1.015 | 0.021 |
| L-Cysteine | 0.014 | 0.210 | 0.929 | -0.106 |
| N6-Acetyl-L-Lysine | 0.098 | 0.401 | 1.054 | 0.076 |
| N-Acetylcysteine | 0.051 | 0.305 | 0.936 | -0.095 |
| N-Acetylputrescine | 0.948 | 0.984 | 1.005 | 0.007 |
| Serotonin | 0.311 | 0.643 | 1.049 | 0.069 |
| Trimethylamine-N-Oxide | 0.029 | 0.253 | 1.320 | 0.400 |
| Ala-Lys | 0.214 | 0.549 | 0.942 | -0.086 |
| N-Acetylhistamine | 0.160 | 0.492 | 0.838 | -0.255 |
| P-Coumaric Acid | 0.029 | 0.253 | 1.093 | 0.128 |
| 1,4-Dihydro-1-Methyl-4-Oxo-3-Pyridinecarboxamide | 0.303 | 0.640 | 1.130 | 0.177 |
| Theobromine | 0.538 | 0.812 | 0.294 | -1.765 |
| Choline | 0.017 | 0.215 | 0.924 | -0.113 |
| 1,5-Diaminopentane | 0.547 | 0.819 | 1.002 | 0.003 |
| Diethanolamine | 0.031 | 0.259 | 0.837 | -0.256 |
| Myoinositol | 0.524 | 0.804 | 0.975 | -0.036 |
| 1-Methylhistidine | 0.462 | 0.762 | 0.988 | -0.017 |
| 5,6-Dihydro-5-Methyluracil | 0.761 | 0.933 | 0.995 | -0.007 |
| 5-Methyluridine | 0.177 | 0.509 | 0.948 | -0.078 |
| Adenine | 0.779 | 0.933 | 0.993 | -0.010 |
| Cytosine | 0.337 | 0.660 | 0.895 | -0.160 |
| Purine | 0.080 | 0.384 | 1.041 | 0.058 |
| Uracil | 0.228 | 0.555 | 0.925 | -0.112 |
| 3,3',5-Triiodo-L-Thyronine | 0.880 | 0.978 | 0.962 | -0.056 |
| N-Methyltryptamine | 0.972 | 0.984 | 1.127 | 0.172 |
| D-Fructose | 0.495 | 0.783 | 1.106 | 0.145 |
| D-Mannose | 0.495 | 0.783 | 1.106 | 0.145 |
| D-Gluconic Acid | 0.942 | 0.984 | 0.973 | -0.040 |
| Orotic Acid | 0.410 | 0.707 | 1.006 | 0.009 |
| Nicotinamide | 0.861 | 0.971 | 0.972 | -0.041 |
| Riboflavin | 0.094 | 0.399 | 0.788 | -0.344 |
| Trigonelline | 0.028 | 0.249 | 1.302 | 0.381 |
| 3-Indolebutyric Acid | 0.382 | 0.694 | 1.022 | 0.032 |
| 2-Aminoethanesulfonic Acid | 0.152 | 0.488 | 0.866 | -0.207 |
| 4-Guanidinobutyric Acid | 0.233 | 0.562 | 1.070 | 0.098 |
| 5-Aminovaleric Acid | 0.209 | 0.541 | 0.916 | -0.127 |
| 6-Aminocaproic-Acid | 0.346 | 0.666 | 1.031 | 0.044 |
| 7-Methyluric Acid | 0.889 | 0.978 | 0.986 | -0.020 |
| Creatinine | 0.559 | 0.827 | 1.027 | 0.038 |
| Dl-2-Aminooctanoic Acid | 0.017 | 0.215 | 0.801 | -0.321 |
| Guanidineacetic Acid | 0.861 | 0.971 | 0.923 | -0.116 |
| L-Dihydroorotic Acid | 0.761 | 0.933 | 1.024 | 0.034 |
| L-Homoserine | 0.166 | 0.497 | 0.959 | -0.060 |
| Maleic Acid | 0.388 | 0.694 | 1.024 | 0.035 |
| LPC(0:0/14:0) | 0.000 | 0.096 | 1.326 | 0.407 |
| LPC(16:0/0:0) | 0.275 | 0.619 | 1.018 | 0.025 |
| Trans-3-Hydroxycotinine | 0.229 | 0.556 | 0.174 | -2.523 |
| L-Homoarginine | 0.390 | 0.697 | 1.063 | 0.088 |
| Pantetheine | 0.633 | 0.876 | 1.015 | 0.022 |
| D-piperidine acid | 0.119 | 0.432 | 0.898 | -0.155 |
| 5'-Deoxy-5'-(Methylthio) Adenosine | 0.088 | 0.387 | 1.042 | 0.059 |
| Sarcosine | 0.917 | 0.984 | 1.009 | 0.013 |
| Imidazoleacetic acid | 0.758 | 0.933 | 1.023 | 0.032 |
| 2-Aminoadipic Acid | 0.070 | 0.353 | 1.212 | 0.277 |
| LPC(17:0/0:0) | 0.720 | 0.926 | 0.997 | -0.004 |
| Sn-Glycero-3-Phosphocholine | 0.594 | 0.855 | 1.105 | 0.144 |
| Indole | 0.094 | 0.399 | 1.067 | 0.094 |
| LPC(15:0/0:0) | 0.128 | 0.441 | 1.070 | 0.097 |
| LPC(0:0/18:2) | 0.227 | 0.555 | 0.975 | -0.037 |
| 2-Hydroxycinnamic acid | 0.902 | 0.981 | 1.040 | 0.056 |
| Carnitine C2:0 | 0.776 | 0.933 | 0.982 | -0.026 |
| DL-Stachydrine | 0.963 | 0.984 | 1.035 | 0.050 |
| L-Norleucine | 0.107 | 0.410 | 1.027 | 0.038 |
| DL-Carnitine | 0.194 | 0.533 | 1.079 | 0.110 |
| 6-Dimethylaminopurine | 0.275 | 0.619 | 1.011 | 0.016 |
| Triethyl-phosphate | 0.787 | 0.933 | 1.048 | 0.067 |
| LPC(O-16:0/2:0) | 0.778 | 0.933 | 0.963 | -0.055 |
| 18-Hydroxycorticosterone | 0.633 | 0.876 | 0.997 | -0.004 |
| Oleamide | 0.086 | 0.384 | 0.800 | -0.322 |
| Carnitine isoC4:0 | 0.938 | 0.984 | 1.025 | 0.035 |
| 2'-Hydroxy-5'-methylacetophenone | 0.749 | 0.930 | 1.057 | 0.079 |
| Spermidine | 0.105 | 0.410 | 1.079 | 0.110 |
| N-Acetyl-L-Histidine | 0.399 | 0.701 | 0.995 | -0.007 |
| 2-Furoylglycine | 0.456 | 0.755 | 1.204 | 0.267 |
| Carnitine-2-methyl-C4 | 0.136 | 0.455 | 1.083 | 0.115 |
| Isonicotinic acid | 0.508 | 0.793 | 0.973 | -0.040 |
| Phe-Pro | 0.190 | 0.529 | 1.050 | 0.071 |
| LPE(16:1/0:0) | 0.050 | 0.305 | 1.177 | 0.235 |
| 3-Chloroaniline | 0.855 | 0.971 | 1.002 | 0.003 |
| Methylcysteine | 0.991 | 0.995 | 0.971 | -0.043 |
| DL-Leucine | 0.346 | 0.666 | 1.031 | 0.044 |
| 6-Methylnicotinamide | 0.927 | 0.984 | 0.956 | -0.065 |
| (R)-2-Hydroxy-3-phenylpropionic-acid | 0.936 | 0.984 | 1.011 | 0.016 |
| 1-Aminopropan-2-ol | 0.029 | 0.253 | 1.320 | 0.400 |
| N-Methylalanine | 0.066 | 0.344 | 0.953 | -0.070 |
| Dihydro-D-sphingosine | 0.660 | 0.889 | 0.995 | -0.007 |
| Hypaphorine | 0.168 | 0.500 | 1.151 | 0.203 |
| N-Methyl-L-Glutamate | 0.070 | 0.353 | 1.212 | 0.277 |
| Phosphocholine | 0.618 | 0.872 | 1.016 | 0.023 |
| 2,4-Dihydroxypteridine | 0.264 | 0.611 | 1.020 | 0.028 |
| Cortisol | 0.536 | 0.811 | 0.996 | -0.005 |
| L-Tryptophanamide | 0.308 | 0.640 | 1.045 | 0.063 |
| Catechol | 0.254 | 0.595 | 1.011 | 0.016 |
| Acetylcholine | 0.902 | 0.981 | 1.018 | 0.025 |
| 1-Hydroxylamino-2-phenylethane | 0.386 | 0.694 | 1.046 | 0.065 |
| β-Alanine | 0.037 | 0.286 | 1.113 | 0.155 |
| D-(+)-sucrose | 0.527 | 0.804 | 0.946 | -0.081 |
| Thr-Phe | 0.141 | 0.464 | 1.104 | 0.143 |
| Salicylaldehyde | 0.732 | 0.930 | 0.925 | -0.112 |
| Benzaldehyde | 0.036 | 0.285 | 1.054 | 0.076 |
| Biliverdin | 0.707 | 0.925 | 1.069 | 0.097 |
| N,-N-diacetyl-O-methylhydroxylamine | 0.142 | 0.469 | 0.959 | -0.060 |
| Hydroxyquinoline | 0.064 | 0.338 | 1.058 | 0.081 |
| DL-O-tyrosine | 0.098 | 0.401 | 1.021 | 0.030 |
| 7-Methylguanosine | 0.716 | 0.926 | 1.001 | 0.002 |
| 6-O-methylguanine | 0.288 | 0.633 | 0.951 | -0.073 |
| Oxypurinol | 0.413 | 0.707 | 1.217 | 0.283 |
| Carnitine C12:0 | 0.041 | 0.296 | 0.837 | -0.258 |
| Allopurinol | 0.002 | 0.132 | 2.199 | 1.137 |
| PC(12:0/12:0) | 0.291 | 0.635 | 1.037 | 0.052 |
| (R)-(-)-2-phenylglycine | 0.386 | 0.694 | 1.046 | 0.065 |
| 1-acetylindole | 0.272 | 0.619 | 1.037 | 0.053 |
| 3-Carboxypropyltrimethylammonium | 0.723 | 0.927 | 1.066 | 0.092 |
| L-Isoleucine | 0.414 | 0.707 | 1.025 | 0.035 |
| LPC(O-18:0/0:0) | 0.404 | 0.704 | 0.895 | -0.160 |
| Creatine phosphate | 0.471 | 0.773 | 0.950 | -0.074 |
| PC(O-16:0/O-2:0) | 0.404 | 0.704 | 0.895 | -0.160 |
| Butenoyl-PAF | 0.110 | 0.410 | 0.944 | -0.082 |
| N-Methyl-α-aminoisobutyric acid | 0.891 | 0.978 | 1.046 | 0.065 |
| Biotinamide | 0.853 | 0.971 | 0.974 | -0.038 |
| 8,8a-deoxy-oleane | 0.916 | 0.984 | 1.111 | 0.152 |
| N-acetylpyrrolidine | 0.653 | 0.889 | 0.940 | -0.089 |
| 1,3-Dicyclohexylurea | 0.631 | 0.876 | 0.963 | -0.054 |
| PC(O-16:0/O-1:0) | 0.758 | 0.933 | 1.000 | 0.000 |
| 4-tert-butylbenzoic-acid | 0.715 | 0.926 | 1.011 | 0.016 |
| 4-Hydroxytryptamine | 0.439 | 0.740 | 0.287 | -1.799 |
| 1-Deoxyvaleric-acid | 0.271 | 0.619 | 0.987 | -0.018 |
| 2-Mercaptobenzothiazole | 0.063 | 0.336 | 0.880 | -0.184 |
| Urobilin | 0.844 | 0.966 | 0.914 | -0.129 |
| 20,26-dihydroxyecdysone | 0.384 | 0.694 | 1.012 | 0.017 |
| Carnitine C6:0 | 0.206 | 0.541 | 0.823 | -0.281 |
| Leu-Gly | 0.008 | 0.210 | 1.213 | 0.278 |
| LPC(0:0/22:4) | 0.277 | 0.619 | 0.934 | -0.099 |
| LPC(22:4/0:0) | 0.277 | 0.619 | 0.934 | -0.099 |
| LPC(0:0/22:5) | 0.425 | 0.724 | 0.957 | -0.063 |
| LPC(22:5/0:0) | 0.386 | 0.694 | 1.089 | 0.124 |
| LPC(20:1/0:0) | 0.110 | 0.410 | 0.944 | -0.082 |
| LPC(20:2/0:0) | 0.316 | 0.647 | 0.960 | -0.058 |
| LPC(0:0/20:2) | 0.316 | 0.647 | 0.960 | -0.058 |
| LPC(0:0/20:3) | 0.203 | 0.541 | 1.107 | 0.147 |
| LPC(20:3/0:0) | 0.203 | 0.541 | 1.107 | 0.147 |
| LPC(0:0/20:4) | 0.638 | 0.876 | 0.956 | -0.064 |
| LPC(20:4/0:0) | 0.638 | 0.876 | 0.956 | -0.064 |
| LPC(18:0/0:0) | 0.778 | 0.933 | 0.963 | -0.055 |
| LPC(18:1/0:0) | 0.329 | 0.657 | 0.957 | -0.063 |
| LPC(18:2/0:0) | 0.227 | 0.555 | 0.975 | -0.037 |
| LPC(0:0/16:0) | 0.275 | 0.619 | 1.018 | 0.025 |
| LPC(16:1/0:0) | 0.128 | 0.441 | 1.077 | 0.108 |
| Mycosporine-glycine | 0.373 | 0.690 | 0.947 | -0.079 |
| Carnitine C18:0 | 0.599 | 0.858 | 0.948 | -0.076 |
| Carnitine C18:2 | 0.914 | 0.984 | 1.021 | 0.030 |
| Carnitine C16:0 | 0.720 | 0.926 | 0.973 | -0.040 |
| Carnitine C16:1 | 0.022 | 0.230 | 0.818 | -0.289 |
| Carnitine C16:2 | 0.048 | 0.305 | 0.886 | -0.174 |
| Carnitine C14-OH | 0.206 | 0.541 | 0.891 | -0.166 |
| Carnitine C14:2-OH | 0.155 | 0.488 | 0.799 | -0.323 |
| Carnitine C14:2 | 0.026 | 0.249 | 0.820 | -0.286 |
| Carnitine C12-OH | 0.113 | 0.415 | 0.914 | -0.130 |
| Carnitine C11:DC | 0.081 | 0.384 | 0.882 | -0.181 |
| Carnitine C13:1 | 0.803 | 0.936 | 0.909 | -0.138 |
| Carnitine C11:0 | 0.961 | 0.984 | 1.011 | 0.016 |
| Carnitine C11:1 | 0.740 | 0.930 | 1.007 | 0.010 |
| Carnitine C10:0 | 0.084 | 0.384 | 0.868 | -0.204 |
| Carnitine C8-OH | 0.619 | 0.872 | 0.884 | -0.178 |
| Carnitine C9:0 | 0.638 | 0.876 | 0.979 | -0.030 |
| Carnitine C8:0 | 0.111 | 0.411 | 0.818 | -0.290 |
| Carnitine C8:1 | 0.379 | 0.694 | 1.078 | 0.108 |
| Carnitine ph-C1 | 0.865 | 0.973 | 0.846 | -0.241 |
| Carnitine C4:DC | 0.489 | 0.780 | 1.055 | 0.077 |
| Carnitine C5:0 | 0.136 | 0.455 | 1.083 | 0.115 |
| Carnitine C5:1 | 0.770 | 0.933 | 1.038 | 0.054 |
| Carnitine C4:0 | 0.938 | 0.984 | 1.025 | 0.035 |
| 1,3-Diphenylguanidine | 0.827 | 0.954 | 0.969 | -0.046 |
| N'-Methyl-2-pyridone-5-carboxamide | 0.148 | 0.483 | 1.090 | 0.125 |
| 5-Methoxytryptamine | 0.962 | 0.984 | 0.990 | -0.015 |
| L-Phenylephrine | 0.655 | 0.889 | 1.013 | 0.018 |
| Theophylline | 0.900 | 0.981 | 0.492 | -1.024 |
| SDMA | 0.965 | 0.984 | 1.012 | 0.017 |
| Methylguanidine | 0.384 | 0.694 | 0.990 | -0.015 |
| N6-methyladenosine | 0.508 | 0.793 | 0.970 | -0.044 |
| 8-Azaguanine | 0.413 | 0.707 | 1.217 | 0.283 |
| Isocytosine | 0.337 | 0.660 | 0.895 | -0.160 |
| Leu-Val | 0.016 | 0.215 | 1.279 | 0.355 |
| Phe-Asn | 0.052 | 0.309 | 1.140 | 0.188 |
| Phe-Met | 0.001 | 0.132 | 1.318 | 0.398 |
| Glu-Met | 0.480 | 0.780 | 1.041 | 0.058 |
| Met-Glu | 0.329 | 0.657 | 1.044 | 0.062 |
| Phe-Val | 0.031 | 0.258 | 1.078 | 0.108 |
| Carnitine C10:1 | 0.157 | 0.489 | 0.815 | -0.295 |
| Carnitine C14:2:DC | 0.012 | 0.210 | 0.833 | -0.263 |
| Caldine | 0.346 | 0.666 | 0.945 | -0.082 |
| 1-Methyladenosine | 0.508 | 0.793 | 0.970 | -0.044 |
| PC(O-16:0/0:0) | 0.893 | 0.979 | 0.995 | -0.007 |
| LPC(O-18:1/0:0) | 0.876 | 0.977 | 0.995 | -0.007 |
| LPC(O-0:0/18:0) | 0.404 | 0.704 | 0.895 | -0.160 |
| NE,NE,NE-TRIMETHYLLYSINE | 0.012 | 0.210 | 1.073 | 0.102 |
| N,N-Dimethylarginine | 0.382 | 0.694 | 1.037 | 0.053 |
| Carnitine C3:0 | 0.368 | 0.687 | 1.109 | 0.149 |
| Phe-Glu | 0.165 | 0.497 | 1.156 | 0.209 |
| Gly-Gly-Phe | 0.967 | 0.984 | 1.017 | 0.024 |
| LPC(0:0/17:0) | 0.870 | 0.975 | 1.005 | 0.008 |
| LPC(0:0/15:0) | 0.128 | 0.441 | 1.070 | 0.097 |
| LPC(12:0/0:0) | 0.062 | 0.336 | 1.206 | 0.271 |
| LPC(O-16:1/0:0) | 0.111 | 0.411 | 0.917 | -0.125 |
| Sphingosyl-phosphocholine | 0.365 | 0.681 | 1.060 | 0.084 |
| LPC(0:0/16:1) | 0.128 | 0.441 | 1.077 | 0.108 |
| LPC(0:0/18:0) | 0.778 | 0.933 | 0.963 | -0.055 |
| LPC(0:0/18:1) | 0.329 | 0.657 | 0.957 | -0.063 |
| LPC(0:0/20:1) | 0.110 | 0.410 | 0.944 | -0.082 |
| Methyldopa | 0.233 | 0.562 | 0.925 | -0.113 |
| (E)-Guggulsterone | 0.003 | 0.132 | 1.114 | 0.156 |
| (Z)-Guggulsterone | 0.003 | 0.132 | 1.114 | 0.156 |
| 1-Methylinosine | 0.584 | 0.850 | 1.001 | 0.001 |
| 2'-O-methylcytidine | 0.807 | 0.939 | 1.009 | 0.013 |
| Acrylamide | 0.024 | 0.244 | 1.059 | 0.083 |
| Agmatine | 0.749 | 0.930 | 0.984 | -0.023 |
| Ala-Phe | 0.136 | 0.455 | 1.032 | 0.045 |
| Cyromazine | 0.889 | 0.978 | 1.029 | 0.042 |
| Cytidine 5'-diphosphate | 0.699 | 0.920 | 1.006 | 0.009 |
| L-Allothreonine | 0.166 | 0.497 | 0.959 | -0.060 |
| D-Glucosaminic acid | 0.221 | 0.554 | 0.887 | -0.173 |
| Guanidine | 0.493 | 0.783 | 0.974 | -0.038 |
| Leu-Ala | 0.199 | 0.541 | 1.141 | 0.190 |
| L-Norvaline | 0.056 | 0.316 | 1.138 | 0.186 |
| Leu-Phe | 0.013 | 0.210 | 1.316 | 0.396 |
| N4-Acetylcytidine | 0.727 | 0.928 | 1.093 | 0.129 |
| Gly-Ile | 0.201 | 0.541 | 1.186 | 0.246 |
| Palmitoylethanolamide（(PEA） | 0.967 | 0.984 | 1.013 | 0.019 |
| Sanguinarine | 0.445 | 0.744 | 1.123 | 0.167 |
| Stachydrine | 0.809 | 0.940 | 1.125 | 0.170 |
| Synephrine | 0.095 | 0.399 | 0.933 | -0.100 |
| Bicine | 0.452 | 0.753 | 0.933 | -0.101 |
| Eicosanoyl-EA | 0.674 | 0.901 | 0.989 | -0.017 |
| Gln-Phe | 0.934 | 0.984 | 1.007 | 0.009 |
| Ile-Met | 0.004 | 0.168 | 1.233 | 0.302 |
| LPE(17:1/0:0) | 0.275 | 0.619 | 1.005 | 0.007 |
| Pro-Ile | 0.104 | 0.409 | 1.207 | 0.271 |
| Ser-Leu | 0.041 | 0.296 | 1.283 | 0.359 |
| Ser-Phe | 0.067 | 0.349 | 1.101 | 0.139 |
| Tyr-Leu | 0.003 | 0.150 | 1.543 | 0.626 |
| 3-Hydroxyphenylurea | 0.789 | 0.933 | 1.011 | 0.015 |
| Caffeine | 0.885 | 0.978 | 1.947 | 0.961 |
| Carnitine C9:1-OH | 0.972 | 0.984 | 1.127 | 0.172 |
| LPE(18:2/0:0) | 0.486 | 0.780 | 1.056 | 0.079 |
| (E,Z)-2-Amino-3,14-octadecadien-1-ol | 0.086 | 0.384 | 0.800 | -0.322 |
| Phe-Trp | 0.740 | 0.930 | 1.031 | 0.044 |
| N(Alpha)-Acetyl-Epsilon-(2-Propenal)Lysine | 0.167 | 0.500 | 1.358 | 0.441 |
| Cyclo(Pro-Leu) | 0.117 | 0.425 | 1.092 | 0.127 |
| Thr-Gln | 0.868 | 0.974 | 1.212 | 0.277 |
| Cyclo(Phe-Glu) | 0.530 | 0.807 | 1.023 | 0.033 |
| Cyclo(Pro-Val) | 0.132 | 0.450 | 1.090 | 0.125 |
| Ile-Asp | 0.022 | 0.230 | 1.365 | 0.449 |
| N-MethyTrans-4-Hydroxy-Proline | 0.796 | 0.936 | 0.944 | -0.082 |
| Ser-Ile | 0.041 | 0.296 | 1.283 | 0.359 |
| 5-Aminoimidazole ribonucleotide | 0.195 | 0.535 | 1.125 | 0.170 |
| N6-(2-Hydroxyethyl)adenosine | 0.885 | 0.978 | 0.967 | -0.049 |
| 7-(alpha-D-glucosyl)-N(6)-isopentenyladenine | 0.091 | 0.394 | 1.326 | 0.407 |
| D-Proline-betaine | 0.936 | 0.984 | 1.027 | 0.038 |
| 2-Methyl-1-Pyrroline | 0.555 | 0.826 | 1.019 | 0.028 |
| Melibiose | 0.750 | 0.930 | 0.939 | -0.091 |
| N-Formylglycine | 0.448 | 0.747 | 0.977 | -0.033 |
| D-Ornithine | 0.275 | 0.619 | 0.923 | -0.115 |
| Dehydroascorbic-acid | 0.095 | 0.399 | 0.952 | -0.070 |
| Androstenediol | 0.852 | 0.971 | 0.992 | -0.011 |
| Triethylenetetramine | 0.024 | 0.244 | 0.911 | -0.135 |
| FFA(10:1) | 0.070 | 0.353 | 0.799 | -0.324 |
| 3-Aminoquinoline | 0.029 | 0.253 | 1.062 | 0.087 |
| L-Isserine | 0.198 | 0.540 | 0.951 | -0.072 |
| 1-Amino-1-cyclobutane-carboxylic-acid | 0.393 | 0.698 | 1.083 | 0.115 |
| N,N-Bis(2-hydroxyethyl)dodecanamide | 0.878 | 0.977 | 0.983 | -0.025 |
| (E)-8-Methyl-6-nonenoic-acid | 0.115 | 0.421 | 0.888 | -0.171 |
| Cork-oximate | 0.933 | 0.984 | 1.079 | 0.110 |
| 8-iso-15-keto-Prostaglandin-F2α | 0.527 | 0.804 | 0.986 | -0.020 |
| 2-Amino-3-phosphonopropionic-acid | 0.176 | 0.509 | 0.889 | -0.170 |
| Inositol 1,3,4-trisphosphate | 0.474 | 0.775 | 1.005 | 0.007 |
| S-methyl-L-thiocitrulline | 0.603 | 0.858 | 0.910 | -0.136 |
| ST-638 | 0.601 | 0.858 | 1.120 | 0.164 |
| Leu-Leu | 0.641 | 0.876 | 1.227 | 0.295 |
| Gly-Lys | 0.910 | 0.984 | 1.026 | 0.037 |
| Gly-Gln | 0.333 | 0.657 | 1.055 | 0.078 |
| Lys-Ser | 0.388 | 0.694 | 0.928 | -0.108 |
| Arg-Glu | 0.033 | 0.269 | 1.226 | 0.294 |
| Glu-His | 0.000 | 0.096 | 1.348 | 0.431 |
| Cyclocreatine | 0.895 | 0.979 | 1.007 | 0.010 |
| γ-Glu-Lys | 0.176 | 0.509 | 0.789 | -0.341 |
| (R)-(-)-1-Amino-2-propanol | 0.014 | 0.210 | 1.180 | 0.238 |
| Gly-Thr | 0.236 | 0.564 | 1.051 | 0.071 |
| Pro-Ser | 0.412 | 0.707 | 0.956 | -0.065 |
| N-Ethylglycine | 0.066 | 0.344 | 0.953 | -0.070 |
| 3-Guanidinopropionic acid | 0.850 | 0.971 | 0.988 | -0.018 |
| Glu-Ser | 0.085 | 0.384 | 1.139 | 0.188 |
| Ammeline | 0.244 | 0.579 | 0.949 | -0.075 |
| Thr-Glu | 0.124 | 0.441 | 1.137 | 0.186 |
| Glu-Cit | 0.635 | 0.876 | 0.932 | -0.101 |
| Glu-Gly | 0.330 | 0.657 | 0.880 | -0.184 |
| N-acetyl-D-Lactosamine | 0.176 | 0.509 | 1.118 | 0.160 |
| (+/-)-High-Proline | 0.048 | 0.305 | 1.151 | 0.203 |
| 3-(imidazol-4-yl)propionic-acid | 0.857 | 0.971 | 0.981 | -0.028 |
| Ser-Pro | 0.409 | 0.707 | 1.076 | 0.105 |
| N1-Acetylspermidine | 0.961 | 0.984 | 1.016 | 0.024 |
| Ser-Val | 0.640 | 0.876 | 0.994 | -0.008 |
| Ile-Gln | 0.012 | 0.210 | 1.401 | 0.486 |
| Ile-Ser | 0.162 | 0.495 | 1.098 | 0.134 |
| Tyr-Glu | 0.086 | 0.384 | 0.830 | -0.269 |
| Ile-Glu | 0.626 | 0.875 | 1.071 | 0.099 |
| Leu-Glu | 0.626 | 0.875 | 1.071 | 0.099 |
| Glu-Ile | 0.626 | 0.875 | 1.071 | 0.099 |
| Ile-Thr | 0.021 | 0.230 | 1.336 | 0.418 |
| Leu-Thr | 0.021 | 0.230 | 1.336 | 0.418 |
| 2,2'-Cyclouridine | 0.999 | 0.999 | 0.996 | -0.006 |
| 3-aminobenzamide | 0.002 | 0.132 | 1.802 | 0.850 |
| N-(2-hydroxyethyl)-3-pyridinecarboxamide | 0.431 | 0.730 | 1.007 | 0.010 |
| Phe-Ala-Ser | 0.027 | 0.249 | 1.329 | 0.410 |
| Ser-Phe-Ala | 0.027 | 0.249 | 1.329 | 0.410 |
| 1-Methylguanosine | 0.716 | 0.926 | 1.001 | 0.002 |
| Cyclo(Ala-Pro) | 0.191 | 0.529 | 1.052 | 0.073 |
| Phe-Gly | 0.789 | 0.933 | 1.020 | 0.029 |
| Val-Ile | 0.021 | 0.230 | 1.242 | 0.312 |
| Val-Leu | 0.021 | 0.230 | 1.242 | 0.312 |
| Asp-Ile | 0.022 | 0.230 | 1.365 | 0.449 |
| Leu-Met | 0.004 | 0.168 | 1.233 | 0.302 |
| cyclo(pro-pro) | 0.350 | 0.668 | 1.066 | 0.092 |
| Phe-Tyr | 0.095 | 0.399 | 1.269 | 0.344 |
| Ile-Leu | 0.641 | 0.876 | 1.227 | 0.295 |
| Phe-Ala-Leu | 0.897 | 0.980 | 0.997 | -0.004 |
| Securinine | 0.692 | 0.914 | 0.944 | -0.082 |
| Thr-Arg | 0.181 | 0.513 | 1.069 | 0.097 |
| Arg-Thr | 0.480 | 0.780 | 0.948 | -0.077 |
| Leu-Asn | 0.295 | 0.638 | 1.032 | 0.045 |
| Glu-Phe-Ala | 0.256 | 0.595 | 1.052 | 0.073 |
| 4-Amino-3-hydroxybutyric acid | 0.699 | 0.920 | 1.083 | 0.115 |
| D-Allo-Isoleucine | 0.414 | 0.707 | 1.025 | 0.035 |
| 5-Acetylamino-6-amino-3-methyluracil | 0.535 | 0.810 | 0.957 | -0.064 |
| Gln-Gly | 0.286 | 0.630 | 1.061 | 0.085 |
| Gly-Glu | 0.330 | 0.657 | 0.880 | -0.184 |
| Lys-Ala | 0.214 | 0.549 | 0.942 | -0.086 |
| Pro-Asn | 0.763 | 0.933 | 1.017 | 0.025 |
| Pro-Asp | 0.218 | 0.554 | 1.100 | 0.138 |
| Arg-Gly | 0.940 | 0.984 | 1.015 | 0.022 |
| Gly-Arg | 0.940 | 0.984 | 1.015 | 0.022 |
| Ser-Lys | 0.761 | 0.933 | 0.986 | -0.021 |
| Ser-Glu | 0.802 | 0.936 | 0.975 | -0.037 |
| Phe-Ala | 0.774 | 0.933 | 1.041 | 0.058 |
| Ile-Asn | 0.295 | 0.638 | 1.032 | 0.045 |
| Leu-Asp | 0.059 | 0.329 | 1.218 | 0.285 |
| Phe-Thr | 0.141 | 0.464 | 1.104 | 0.143 |
| Phe-Hyp | 0.102 | 0.407 | 0.948 | -0.077 |
| Glu-Phe | 0.165 | 0.497 | 1.156 | 0.209 |
| Glu-Arg | 0.013 | 0.210 | 1.290 | 0.367 |
| 2-Butyl-3-(4-hydroxybenzoyl)benzofuran | 0.002 | 0.132 | 0.816 | -0.294 |
| Quinmerac | 0.716 | 0.926 | 1.001 | 0.001 |
| Chaps | 0.803 | 0.936 | 0.886 | -0.175 |
| trans-resveratrol-3-O-sulfate | 0.741 | 0.930 | 0.970 | -0.044 |

**Table S8.** 839 Metabolites comparisions between group 3 trajectory and group 1 trajectory in males.

| Compounds | P_value | P_adjusted | FC | log2FC |
| --- | --- | --- | --- | --- |
| 3-carboxy-4-methyl-5-propyl-2-furanpropionic acid | 0.050 | 0.182 | 1.419 | 0.505 |
| 2,4-diacetamino-2,4,6-triphenoxy-D-mannopyranose | 0.005 | 0.039 | 1.589 | 0.668 |
| L-Threonine | 0.876 | 0.963 | 0.964 | -0.053 |
| L-Arginine | 0.027 | 0.119 | 1.076 | 0.106 |
| L-Aspartic Acid | 0.179 | 0.414 | 1.066 | 0.092 |
| L-Citrulline | 0.154 | 0.379 | 0.879 | -0.185 |
| L-Glutamic Acid | 0.000 | 0.001 | 1.348 | 0.431 |
| L-Phenylalanine | 0.001 | 0.017 | 1.062 | 0.087 |
| L-Serine | 0.536 | 0.752 | 1.013 | 0.018 |
| L-Tryptophan | 0.090 | 0.282 | 1.060 | 0.085 |
| (5-L-Glutamyl)-L-Amino Acid | 0.094 | 0.289 | 1.131 | 0.177 |
| Allantoin | 0.035 | 0.142 | 1.625 | 0.701 |
| Asp-Phe | 0.351 | 0.637 | 1.216 | 0.282 |
| Glutathione Oxidized | 0.697 | 0.871 | 0.977 | -0.033 |
| Hexanoyl Glycine | 0.004 | 0.035 | 0.724 | -0.466 |
| L-Asparagine Anhydrous | 0.400 | 0.666 | 1.016 | 0.022 |
| L-Glutamine | 0.005 | 0.039 | 0.860 | -0.218 |
| L-Homocitrulline | 0.944 | 0.983 | 1.036 | 0.051 |
| L-Theanine | 0.292 | 0.568 | 0.930 | -0.105 |
| N-Acetylaspartate | 0.768 | 0.922 | 1.006 | 0.008 |
| N-Acetyl-L-Leucine | 0.033 | 0.133 | 1.150 | 0.202 |
| N-Acetyl-L-Tyrosine | 0.427 | 0.695 | 1.008 | 0.011 |
| N-Acetylneuraminic Acid(SA) | 0.702 | 0.871 | 1.149 | 0.200 |
| Gly-Leu | 0.051 | 0.182 | 1.350 | 0.433 |
| N-Isovaleroylglycine | 0.297 | 0.572 | 1.182 | 0.241 |
| N-Propionylglycine | 0.000 | 0.003 | 1.103 | 0.141 |
| Nα-Acetyl-L-Arginine | 0.342 | 0.631 | 1.181 | 0.240 |
| O-Phospho-L-Serine | 0.330 | 0.615 | 0.906 | -0.143 |
| Phenylacetyl-L-Glutamine | 0.386 | 0.660 | 1.162 | 0.217 |
| Phe-Phe | 0.428 | 0.697 | 1.049 | 0.069 |
| S-(5-Adenosy)-L-Homocysteine | 0.115 | 0.328 | 1.104 | 0.142 |
| S-Sulfo-L-Cysteine | 0.896 | 0.970 | 0.907 | -0.140 |
| Trans-4-Hydroxy-L-Proline | 0.000 | 0.003 | 1.103 | 0.141 |
| γ-L-Glutamate-Cysteine | 0.113 | 0.325 | 0.945 | -0.081 |
| N-Acetyl-L-phenylalanine | 0.208 | 0.457 | 0.980 | -0.030 |
| Benzoylformic Acid | 0.085 | 0.271 | 0.924 | -0.114 |
| 3-Hydroxyanthranilic Acid | 0.122 | 0.333 | 1.075 | 0.104 |
| P–Hydroxyphenyl Acetic Acid | 0.979 | 1.000 | 1.085 | 0.117 |
| 2-Picolinic Acid | 0.812 | 0.937 | 0.995 | -0.007 |
| 4-Pyridoxic Acid | 0.018 | 0.089 | 1.182 | 0.241 |
| 6-Hydroxynicotinic Acid | 0.029 | 0.122 | 1.531 | 0.614 |
| Taurocholic acid | 0.844 | 0.949 | 1.048 | 0.067 |
| Taurochenodesoxycholic Acid | 0.783 | 0.926 | 0.780 | -0.358 |
| Glycolithocholic acid | 0.171 | 0.404 | 1.034 | 0.048 |
| Hyodeoxycholic acid | 0.516 | 0.748 | 0.921 | -0.119 |
| Glycoursodeoxycholic Acid | 0.236 | 0.495 | 0.720 | -0.474 |
| Glycochenodeoxycholic Acid | 0.482 | 0.736 | 0.725 | -0.463 |
| Chenodeoxycholic Acid | 0.441 | 0.709 | 1.514 | 0.599 |
| 4-Methylcatechol | 0.478 | 0.736 | 1.061 | 0.086 |
| 4-Hydroxy-3-methoxybenzaldehyde | 0.156 | 0.381 | 0.925 | -0.113 |
| 1,7-Dimethylxanthine | 0.683 | 0.860 | 1.604 | 0.682 |
| 1-Methylxanthine | 0.934 | 0.980 | 0.930 | -0.105 |
| Xanthine | 0.118 | 0.328 | 1.116 | 0.159 |
| 3-Methylxanthine | 0.934 | 0.980 | 0.930 | -0.105 |
| 5-Methylcytosine | 0.440 | 0.708 | 1.071 | 0.099 |
| 7-Methylxanthine | 0.934 | 0.980 | 0.930 | -0.105 |
| Guanosine | 0.045 | 0.171 | 1.751 | 0.808 |
| Hypoxanthine | 0.574 | 0.784 | 1.115 | 0.158 |
| Uridine | 0.176 | 0.412 | 1.021 | 0.030 |
| L-Thyroxine | 0.844 | 0.949 | 0.988 | -0.017 |
| Norepinephrine | 0.966 | 0.995 | 1.025 | 0.036 |
| Succinic Acid | 0.009 | 0.060 | 1.129 | 0.175 |
| Cis-Aconitic Acid | 0.260 | 0.529 | 0.943 | -0.085 |
| Melatonin | 0.670 | 0.849 | 0.988 | -0.017 |
| Tryptamine | 0.019 | 0.091 | 1.041 | 0.059 |
| D-Glucose | 0.499 | 0.736 | 1.013 | 0.018 |
| D-Trehalose | 0.859 | 0.952 | 1.014 | 0.020 |
| D-Glucose 6-Phosphate | 0.118 | 0.328 | 1.076 | 0.106 |
| Lactose | 0.859 | 0.952 | 1.014 | 0.020 |
| Lactulose | 0.859 | 0.952 | 1.014 | 0.020 |
| L-Fucose | 0.001 | 0.017 | 0.643 | -0.637 |
| Maltose | 0.859 | 0.952 | 1.014 | 0.020 |
| D-Glucoronic Acid | 0.013 | 0.074 | 1.119 | 0.163 |
| Pantothenate | 0.000 | 0.006 | 1.172 | 0.229 |
| 3-Indolepropionic Acid | 0.206 | 0.454 | 1.502 | 0.587 |
| Indole-3-Carboxaldehyde | 0.994 | 1.000 | 0.942 | -0.087 |
| Methyl Indole-3-Acetate | 0.043 | 0.169 | 1.182 | 0.241 |
| 2-Hydroxybutanoic Acid | 0.002 | 0.028 | 0.580 | -0.787 |
| 2-Hydroxyisocaproic Acid | 0.140 | 0.359 | 1.028 | 0.040 |
| 2-Methylsuccinic Acid | 0.559 | 0.770 | 1.125 | 0.169 |
| 3-Hydroxy-3-Methyl Butyric Acid | 0.123 | 0.334 | 0.908 | -0.140 |
| 3-Methylcrotonyl Glycine | 0.145 | 0.364 | 1.138 | 0.187 |
| 4-Hydroxy-2-Oxoglutaric Acid | 0.016 | 0.085 | 1.175 | 0.233 |
| Adipic Acid | 0.205 | 0.454 | 0.960 | -0.060 |
| Azelaic Acid | 0.344 | 0.632 | 0.959 | -0.061 |
| Caffeic Acid | 0.507 | 0.744 | 1.013 | 0.018 |
| Creatine | 0.409 | 0.672 | 1.167 | 0.223 |
| Dodecanedioic Aicd | 0.000 | 0.003 | 0.862 | -0.214 |
| Glutaric Acid | 0.559 | 0.770 | 1.125 | 0.169 |
| Guanidinoethyl Sulfonate | 0.913 | 0.979 | 0.999 | -0.002 |
| Hippuric Acid | 0.596 | 0.795 | 1.013 | 0.019 |
| Hydrocinnamic Acid | 0.351 | 0.637 | 0.979 | -0.031 |
| L-kynurenine | 0.001 | 0.017 | 1.116 | 0.159 |
| Kynurenic Acid | 0.000 | 0.003 | 1.252 | 0.324 |
| L-Lactic Acid | 0.407 | 0.670 | 1.076 | 0.106 |
| Malonicacid | 0.002 | 0.022 | 0.594 | -0.751 |
| Mandelic Acid | 0.979 | 1.000 | 1.085 | 0.117 |
| Methylmalonic Acid | 0.009 | 0.060 | 1.129 | 0.175 |
| Phenyllactate(Pla) | 0.961 | 0.992 | 0.991 | -0.013 |
| Pyrrole-2-Carboxylic Acid | 0.062 | 0.215 | 1.152 | 0.204 |
| Sebacate | 0.059 | 0.206 | 0.877 | -0.189 |
| Shikimic Acid | 0.021 | 0.101 | 1.346 | 0.429 |
| Subericacid | 0.224 | 0.486 | 0.935 | -0.097 |
| TXB2 | 0.273 | 0.542 | 0.937 | -0.094 |
| (±)15-HETE | 0.058 | 0.205 | 1.122 | 0.167 |
| LPG(18:1/0:0) | 0.460 | 0.731 | 0.957 | -0.063 |
| LPE(18:1/0:0) | 0.001 | 0.015 | 0.748 | -0.419 |
| LPE(18:0/0:0) | 0.499 | 0.736 | 1.081 | 0.112 |
| LPE(16:0/0:0) | 0.532 | 0.752 | 1.039 | 0.056 |
| LPE(14:0/0:0) | 0.027 | 0.117 | 1.159 | 0.213 |
| LPA(0:0/18:0) | 0.589 | 0.793 | 0.974 | -0.038 |
| LPA(0:0/16:0) | 0.939 | 0.982 | 1.030 | 0.043 |
| LipoxinA4 | 0.495 | 0.736 | 0.964 | -0.053 |
| 13-HOTrE | 0.000 | 0.009 | 2.054 | 1.038 |
| 9,10-DiHOME | 0.080 | 0.261 | 0.779 | -0.360 |
| FFA(18:3) | 0.015 | 0.082 | 1.294 | 0.372 |
| FFA(16:0) | 0.854 | 0.951 | 1.034 | 0.048 |
| FFA(18:2) | 0.827 | 0.942 | 0.983 | -0.024 |
| FFA(12:0) | 0.033 | 0.133 | 0.918 | -0.124 |
| FFA(18:1) | 0.038 | 0.152 | 0.884 | -0.178 |
| EPA | 0.001 | 0.018 | 1.329 | 0.410 |
| FFA(20:2) | 0.140 | 0.359 | 0.940 | -0.089 |
| FFA(10:0) | 0.291 | 0.567 | 0.173 | -2.530 |
| AA | 0.871 | 0.959 | 1.043 | 0.060 |
| Urocanic Acid | 0.074 | 0.246 | 1.052 | 0.073 |
| 4-Hydroxybenzaldehyde | 0.147 | 0.366 | 0.917 | -0.126 |
| Neopterin | 0.520 | 0.749 | 1.005 | 0.007 |
| Ethylmalonate | 0.931 | 0.980 | 1.119 | 0.162 |
| 2-(Formylamino)Benzoic Acid | 0.156 | 0.381 | 1.117 | 0.160 |
| Ureidoisobutyric Acid | 0.123 | 0.334 | 1.042 | 0.059 |
| Uridine 5-Monophosphate | 0.559 | 0.770 | 1.023 | 0.033 |
| N-Acetylglycine | 0.000 | 0.000 | 0.700 | -0.514 |
| 3-Hydroxyhippuric Acid | 0.126 | 0.338 | 1.281 | 0.357 |
| 2-(Dimethylamino)Guanosine | 0.003 | 0.032 | 1.108 | 0.148 |
| 5-Hydroxyhexanoic Acid | 0.140 | 0.359 | 1.028 | 0.040 |
| Β-Pseudouridine | 0.152 | 0.377 | 1.073 | 0.102 |
| N-Acetylthreonine | 0.622 | 0.814 | 1.004 | 0.006 |
| 3,4,5-Trimethoxybenzoic Acid | 0.934 | 0.980 | 0.846 | -0.241 |
| Hypoxanthine-9-β-D-Arabinofuranoside | 0.017 | 0.085 | 2.187 | 1.129 |
| D-Sedoheptuiose 7-Phosphate | 0.844 | 0.949 | 0.970 | -0.045 |
| D-Fructose 6-Phosphate-Disodium Salt | 0.118 | 0.328 | 1.076 | 0.106 |
| Aminomalonic Acid | 0.014 | 0.078 | 1.056 | 0.079 |
| 8,15-Dihete | 0.089 | 0.279 | 0.919 | -0.122 |
| N-Acetyl-L-methionine | 0.951 | 0.986 | 1.006 | 0.009 |
| Argininosuccinic acid | 0.013 | 0.073 | 1.138 | 0.186 |
| 2-Deoxyribose 1-Phosphate | 0.616 | 0.809 | 0.917 | -0.124 |
| N-Acetylglucosamine 1-Phosphate | 0.008 | 0.057 | 1.108 | 0.148 |
| Jasmonic acid | 0.391 | 0.661 | 1.198 | 0.261 |
| Indole-3-lactic acid | 0.773 | 0.924 | 1.006 | 0.008 |
| (3-Methoxy-4-hydroxyphenyl)ethylene glycol sulfate | 0.196 | 0.440 | 0.896 | -0.159 |
| Xanthosine | 0.050 | 0.182 | 1.155 | 0.208 |
| estrone 3-sulfate | 0.986 | 1.000 | 0.967 | -0.048 |
| 1-Methylguanine | 0.854 | 0.951 | 1.125 | 0.170 |
| DL-3,4-Dihydroxyphenyl glycol | 0.627 | 0.818 | 1.036 | 0.052 |
| dihydrotachysterol | 0.240 | 0.498 | 0.926 | -0.111 |
| Indoleacrylic acid | 0.182 | 0.415 | 1.438 | 0.524 |
| 2-(4-Hydroxyphenyl)ethanol | 0.861 | 0.953 | 1.008 | 0.011 |
| Hydroxyphenyllactic acid | 0.670 | 0.849 | 1.062 | 0.087 |
| Indole 3-carbinol | 0.544 | 0.761 | 0.999 | -0.001 |
| 2-Methylguanosine | 0.737 | 0.899 | 1.011 | 0.015 |
| 1,2,3-Trihydroxybenzene | 0.984 | 1.000 | 1.102 | 0.140 |
| N-lactoyl-phenylalanine | 0.000 | 0.001 | 1.423 | 0.509 |
| N-Acetyl-L-alanine | 0.195 | 0.439 | 1.041 | 0.058 |
| Cyclamic acid | 0.405 | 0.669 | 0.908 | -0.139 |
| D-Malic acid | 0.476 | 0.736 | 1.015 | 0.021 |
| Tetradecanedioic acid | 0.001 | 0.015 | 0.783 | -0.353 |
| Uridine triphosphate(UTP) | 0.237 | 0.495 | 1.214 | 0.280 |
| 6β-hydroxytestosterone | 0.589 | 0.793 | 0.991 | -0.013 |
| O-Acetyl-L-serine | 0.328 | 0.613 | 1.008 | 0.012 |
| Indoxylsulfuric acid | 0.031 | 0.128 | 1.384 | 0.469 |
| Porphobilinogen | 0.778 | 0.926 | 0.842 | -0.248 |
| Hydroquinone | 0.083 | 0.268 | 1.324 | 0.405 |
| Anthranilic acid | 0.019 | 0.094 | 1.342 | 0.425 |
| Indoleacetaldehyde | 0.005 | 0.039 | 1.043 | 0.061 |
| Hexadecanedioic acid | 0.000 | 0.002 | 0.741 | -0.433 |
| Pyrophosphate | 0.007 | 0.053 | 1.279 | 0.355 |
| 2-hydroxy-2-(4-hydroxy-3-methoxyphenyl)acetic acid | 0.466 | 0.731 | 0.965 | -0.051 |
| Glu-Leu | 0.000 | 0.000 | 1.285 | 0.361 |
| 5-oxoETE | 0.035 | 0.140 | 1.436 | 0.522 |
| Ethylsalicylate | 0.844 | 0.949 | 0.973 | -0.039 |
| Octadecanamide | 0.783 | 0.926 | 1.080 | 0.112 |
| Undecanedioic acid | 0.387 | 0.660 | 0.903 | -0.147 |
| Oxaloacetic acid | 0.512 | 0.746 | 1.066 | 0.092 |
| Phenoxyacetic acid | 0.771 | 0.923 | 1.090 | 0.124 |
| 4-Hydroxybenzyl alcohol | 0.199 | 0.446 | 1.024 | 0.034 |
| Methanesulfonic acid | 0.899 | 0.970 | 1.035 | 0.050 |
| Propylparaben | 0.384 | 0.660 | 0.906 | -0.142 |
| Butylparaben | 0.195 | 0.439 | 1.243 | 0.314 |
| Methylparaben | 0.524 | 0.750 | 0.951 | -0.072 |
| 44986 | 0.008 | 0.057 | 1.615 | 0.692 |
| (±)12-HEPE | 0.000 | 0.009 | 1.971 | 0.979 |
| (±)12-HETE | 0.089 | 0.279 | 1.218 | 0.285 |
| (±)15-HEPE | 0.000 | 0.009 | 1.971 | 0.979 |
| (±)17-HDHA | 0.001 | 0.018 | 1.811 | 0.857 |
| (±)18-HEPE | 0.000 | 0.009 | 1.971 | 0.979 |
| (±)4-HDHA | 0.004 | 0.036 | 1.194 | 0.256 |
| (±)5-HEPE | 0.000 | 0.007 | 1.887 | 0.916 |
| (±)5-HETE | 0.181 | 0.415 | 1.188 | 0.248 |
| (±)9-HETE | 0.181 | 0.415 | 1.188 | 0.248 |
| 11,12-EET | 0.028 | 0.122 | 1.188 | 0.249 |
| 13-oxoODE | 0.027 | 0.116 | 1.556 | 0.638 |
| 14(S)-HDHA | 0.001 | 0.014 | 1.713 | 0.777 |
| 15-oxoETE | 0.008 | 0.058 | 1.548 | 0.630 |
| 5(S),15(S)-DiHETE | 0.160 | 0.385 | 1.381 | 0.466 |
| 5,6-DiHETrE | 0.365 | 0.644 | 1.063 | 0.088 |
| 5-HETrE | 0.013 | 0.074 | 1.374 | 0.459 |
| 9-oxoODE | 0.027 | 0.116 | 1.556 | 0.638 |
| LTB4 | 0.109 | 0.317 | 1.414 | 0.500 |
| PDX | 0.008 | 0.057 | 1.615 | 0.692 |
| Prostaglandin E2 | 0.401 | 0.666 | 1.046 | 0.065 |
| RvD5 | 0.008 | 0.057 | 1.615 | 0.692 |
| Nα-Acetyl-L-glutamine | 0.447 | 0.714 | 1.006 | 0.009 |
| FFA(14:0) | 0.549 | 0.766 | 0.987 | -0.018 |
| 4-Hydroxyhippurate | 0.099 | 0.298 | 1.341 | 0.424 |
| 3-Hydroxyglutaric acid | 0.609 | 0.808 | 1.068 | 0.095 |
| 3-(3-Hydroxyphenyl)-3-hydroxypropanoic acid | 0.532 | 0.752 | 1.037 | 0.052 |
| N-acetylornithine | 0.120 | 0.332 | 1.051 | 0.072 |
| N-Alpha-Acetyl-L-Asparagine | 0.305 | 0.582 | 0.956 | -0.065 |
| N-Amidino-L-Aspartate | 0.050 | 0.182 | 1.129 | 0.175 |
| Lumichrome | 0.128 | 0.341 | 0.940 | -0.089 |
| 3-Amino-4-Hydroxybenzoic Acid | 0.122 | 0.333 | 1.075 | 0.104 |
| 2',4'-Dihydroxyacetophenone | 0.524 | 0.750 | 0.951 | -0.072 |
| Inosine | 0.015 | 0.081 | 2.113 | 1.079 |
| L-Sepiapterin | 0.003 | 0.033 | 1.159 | 0.213 |
| Phosphoenolpyruvate | 0.961 | 0.992 | 1.000 | -0.001 |
| Uric acid | 0.410 | 0.674 | 1.242 | 0.313 |
| 3-hydroxyphenylacetic acid | 0.021 | 0.098 | 1.282 | 0.358 |
| 7-Methylguanine | 0.854 | 0.951 | 1.125 | 0.170 |
| Nicotinic Acid | 0.622 | 0.814 | 1.004 | 0.006 |
| 3-(4-Hydroxyphenyl)-Propionic Acid | 0.711 | 0.876 | 0.992 | -0.011 |
| 5,6-Dimethylbenzimidazole | 0.011 | 0.065 | 1.041 | 0.058 |
| Gly-Phe | 0.747 | 0.908 | 0.997 | -0.004 |
| 13(R)-HODE | 0.065 | 0.219 | 1.317 | 0.398 |
| p-Cresol | 0.038 | 0.152 | 1.238 | 0.308 |
| Indole-4-carboxaldehyde | 0.994 | 1.000 | 0.942 | -0.087 |
| 9(S)-HpOTrE | 0.009 | 0.062 | 0.767 | -0.383 |
| Iminodiacetic acid | 0.115 | 0.328 | 1.076 | 0.106 |
| 2-Methyl-d-erythritol 2,4-cyclodiphosphate | 0.116 | 0.328 | 1.062 | 0.086 |
| Tauroursodeoxycholic acid | 0.497 | 0.736 | 1.026 | 0.037 |
| Gly-Val | 0.063 | 0.216 | 1.285 | 0.362 |
| FFA(18:4) | 0.007 | 0.056 | 1.217 | 0.283 |
| alpha-Muricholic acid | 0.365 | 0.644 | 0.851 | -0.233 |
| 12,13-DiHOME | 0.075 | 0.247 | 0.763 | -0.391 |
| Sphingosine 1-phosphate | 0.984 | 1.000 | 0.916 | -0.127 |
| 4-acetoxyphenol | 0.163 | 0.390 | 1.146 | 0.196 |
| 2-ethyl-2-hydroxybutyric acid | 1.000 | 1.000 | 1.001 | 0.001 |
| 7-ketolithocholic acid | 0.389 | 0.660 | 1.048 | 0.068 |
| 1,6-anhydro-β-D-glucose | 0.485 | 0.736 | 1.097 | 0.134 |
| 16-Hydroxyhexadecanoic acid | 0.304 | 0.582 | 0.954 | -0.068 |
| 12-ketolithocholic acid | 0.389 | 0.660 | 1.048 | 0.068 |
| N,N′-dicyclohexylcarbodiimide | 0.287 | 0.560 | 0.962 | -0.056 |
| Gamma-Mercholic Acid | 0.365 | 0.644 | 0.851 | -0.233 |
| Apocholic acid | 0.389 | 0.660 | 1.048 | 0.068 |
| 2-hydroxyhexadecanoic acid | 0.304 | 0.582 | 0.954 | -0.068 |
| Indole-3-carboxylic acid | 0.172 | 0.405 | 1.025 | 0.036 |
| 4-Methyl-2-oxovaleric acid | 0.058 | 0.205 | 0.824 | -0.279 |
| FFA(20:4) | 0.581 | 0.787 | 1.071 | 0.099 |
| Quinolinic acid | 0.889 | 0.967 | 0.992 | -0.011 |
| p-Tolyl Sulfate | 0.642 | 0.830 | 0.821 | -0.285 |
| 5-nitrobenzimidazole | 0.438 | 0.706 | 0.996 | -0.006 |
| Lythramine | 0.117 | 0.328 | 0.920 | -0.120 |
| Acetaminophen | 0.869 | 0.958 | 0.827 | -0.274 |
| 3-Sulfocatechol | 0.070 | 0.235 | 1.309 | 0.388 |
| 6-hydroxy-3-succinylpyridine | 0.099 | 0.298 | 1.341 | 0.424 |
| 2-(4-hydroxyphenyl) propionate | 0.936 | 0.980 | 1.019 | 0.027 |
| D-Mannose 6-phosphate | 0.118 | 0.328 | 1.076 | 0.106 |
| 2-amino-4-oxovaleric acid | 0.195 | 0.439 | 1.041 | 0.058 |
| 1-O-vanillyl-β-D-glucose | 0.277 | 0.546 | 1.058 | 0.081 |
| L-2-amino-6-oximelic acid | 0.024 | 0.112 | 1.224 | 0.292 |
| 3-(pyrazol-1-yl)-L-alanine | 0.032 | 0.130 | 1.063 | 0.088 |
| 1-pyrroline-4-hydroxy-2-carboxylate | 0.901 | 0.970 | 0.998 | -0.003 |
| LPE(0:0/22:4) | 0.011 | 0.065 | 0.765 | -0.386 |
| LPE(22:4/0:0) | 0.011 | 0.065 | 0.765 | -0.386 |
| LPE(0:0/22:5) | 0.005 | 0.039 | 0.743 | -0.429 |
| LPE(22:5/0:0) | 0.005 | 0.039 | 0.743 | -0.429 |
| LPE(0:0/22:6) | 0.010 | 0.063 | 1.181 | 0.240 |
| LPE(22:6/0:0) | 0.010 | 0.063 | 1.181 | 0.240 |
| LPE(0:0/20:2) | 0.126 | 0.338 | 0.871 | -0.199 |
| LPE(20:2/0:0) | 0.126 | 0.338 | 0.871 | -0.199 |
| LPE(0:0/20:3) | 0.642 | 0.830 | 0.950 | -0.075 |
| LPE(20:3/0:0) | 0.642 | 0.830 | 0.950 | -0.075 |
| LPE(20:4/0:0) | 0.080 | 0.261 | 0.901 | -0.150 |
| LPE(0:0/20:5) | 0.025 | 0.113 | 1.205 | 0.269 |
| LPE(20:5/0:0) | 0.025 | 0.113 | 1.205 | 0.269 |
| LPE(0:0/18:0) | 0.499 | 0.736 | 1.081 | 0.112 |
| LPE(0:0/18:2) | 0.063 | 0.215 | 0.857 | -0.223 |
| LPE(0:0/16:0) | 0.532 | 0.752 | 1.039 | 0.056 |
| LPE(0:0/16:1) | 0.357 | 0.643 | 0.952 | -0.071 |
| 1-Aminocyclohexanoic acid | 0.140 | 0.359 | 1.001 | 0.001 |
| Ureidosuccinic acid | 0.300 | 0.576 | 1.035 | 0.049 |
| 2-Hydroxycaprylic acid | 0.478 | 0.736 | 0.987 | -0.019 |
| 2-hydroxyphenylacetic acid | 0.009 | 0.062 | 1.151 | 0.203 |
| 4-Hydroxy-3-methylbenzoic acid | 0.009 | 0.062 | 1.151 | 0.203 |
| 2-Hydroxy-2-Methyl Butyric acid | 0.726 | 0.887 | 1.054 | 0.076 |
| 2-Octenoic acid | 0.024 | 0.112 | 1.128 | 0.174 |
| 2-Methylglutaric Acid | 0.369 | 0.648 | 0.943 | -0.084 |
| (S)-Leucic acid | 0.140 | 0.359 | 1.028 | 0.040 |
| Glycohyodeoxycholic acid | 0.249 | 0.509 | 0.696 | -0.523 |
| N-Cinnamylglycine | 0.030 | 0.128 | 0.566 | -0.821 |
| (R)-(-)-2-Phenylpropionic Acid | 0.351 | 0.637 | 0.979 | -0.031 |
| 8-Aminooctanoic Acid | 0.096 | 0.293 | 1.012 | 0.017 |
| 4-Methoxysalicylic Acid | 0.341 | 0.630 | 1.129 | 0.175 |
| Tridecanedioic acid | 0.616 | 0.809 | 1.015 | 0.021 |
| 12-Hydroxyoctadecanoic acid | 0.232 | 0.493 | 0.946 | -0.081 |
| N-Palmitoylglycine | 0.226 | 0.486 | 0.969 | -0.046 |
| Taurolithocholic acid | 0.974 | 1.000 | 0.804 | -0.316 |
| 13(S)-HOTrE(γ) | 0.000 | 0.010 | 1.637 | 0.711 |
| 9(S)-HOTrE | 0.001 | 0.019 | 1.801 | 0.849 |
| (±)8-HETE | 0.046 | 0.175 | 1.189 | 0.250 |
| 8(S)-HETrE | 0.013 | 0.074 | 1.374 | 0.459 |
| 15(S)-HETrE | 0.013 | 0.074 | 1.374 | 0.459 |
| 9(S),12(S),13(S)-TriHOME | 0.468 | 0.733 | 1.090 | 0.125 |
| Bicyclo Prostaglandin E2 | 0.182 | 0.415 | 1.150 | 0.201 |
| 20-COOH-AA | 0.771 | 0.923 | 1.180 | 0.239 |
| Prostaglandin B2 | 0.236 | 0.495 | 1.136 | 0.184 |
| 6-trans-12-epi Leukotriene B4 | 0.109 | 0.317 | 1.414 | 0.500 |
| 6-trans Leukotriene B4 | 0.109 | 0.317 | 1.414 | 0.500 |
| 13-HDoHE | 0.001 | 0.017 | 1.864 | 0.898 |
| 10-HDoHE | 0.001 | 0.017 | 1.621 | 0.697 |
| 8-HDoHE | 0.004 | 0.038 | 1.661 | 0.732 |
| 11-HDoHE | 0.001 | 0.017 | 1.887 | 0.916 |
| 16-HDoHE | 0.002 | 0.022 | 1.739 | 0.799 |
| 20-HDoHE | 0.003 | 0.029 | 1.756 | 0.812 |
| 11-HEDE | 0.134 | 0.356 | 1.193 | 0.254 |
| 15-HEDE | 0.134 | 0.356 | 1.193 | 0.254 |
| (±)19(20)-EpDPE(A) | 0.003 | 0.033 | 1.253 | 0.325 |
| 11β-Prostaglandin E2 | 0.401 | 0.666 | 1.046 | 0.065 |
| Glu-Gln | 0.095 | 0.292 | 0.721 | -0.471 |
| Ethionamide | 0.812 | 0.937 | 0.927 | -0.109 |
| Glycerophospho-N-Arachidonoyl Ethanolamine | 0.080 | 0.261 | 0.901 | -0.150 |
| Testosterone sulfate | 0.686 | 0.861 | 0.915 | -0.129 |
| FFA(16:2) | 0.221 | 0.482 | 1.352 | 0.435 |
| (R)-(-)-Mandelic acid | 0.015 | 0.079 | 1.396 | 0.481 |
| (R)-3-Hydroxybutanoic acid | 0.002 | 0.028 | 0.580 | -0.787 |
| 2-Hydroxyhexanoic acid | 0.140 | 0.359 | 1.028 | 0.040 |
| 2'-O-methyluridine | 0.660 | 0.843 | 1.003 | 0.004 |
| 2-Phenylbutyric acid | 0.926 | 0.980 | 1.013 | 0.019 |
| 3-Hydroxycinnamic acid | 0.591 | 0.794 | 1.015 | 0.022 |
| 3-Methyluridine | 0.489 | 0.736 | 1.028 | 0.040 |
| 3-Phenoxybenzoic acid | 0.761 | 0.919 | 1.139 | 0.187 |
| 4-Ethyloctanoic acid | 0.394 | 0.663 | 0.992 | -0.011 |
| 4-Methoxyphenol | 0.478 | 0.736 | 1.061 | 0.086 |
| 5-Hydroxy-2'-deoxyuridine | 0.157 | 0.381 | 1.076 | 0.106 |
| 6-Hydroxyflavone (6-HF) | 0.032 | 0.130 | 1.607 | 0.684 |
| Acetylvaline | 0.208 | 0.457 | 1.064 | 0.089 |
| D-Galacturonic Acid | 0.005 | 0.039 | 1.204 | 0.267 |
| Dimethylmalonic acid | 0.559 | 0.770 | 1.125 | 0.169 |
| D-Tagatose | 0.499 | 0.736 | 1.013 | 0.018 |
| Val-Ala | 0.379 | 0.658 | 0.948 | -0.077 |
| Isethionic acid | 0.994 | 1.000 | 0.985 | -0.022 |
| Octadecanedioic acid | 0.000 | 0.001 | 0.734 | -0.446 |
| Sucrose 6′-monophosphate | 0.466 | 0.731 | 0.975 | -0.037 |
| Traumatic acid | 0.050 | 0.182 | 0.885 | -0.177 |
| Phosphatidylethanolamine lyso alkenyl 16:0 | 0.000 | 0.006 | 0.900 | -0.152 |
| LPA(16:0/0:0) | 0.557 | 0.770 | 0.992 | -0.011 |
| 3-Hydroxy-tetradecanoic acid | 0.017 | 0.085 | 0.835 | -0.260 |
| Acetanilide | 0.594 | 0.794 | 0.993 | -0.010 |
| FFA(22:4) | 0.616 | 0.809 | 0.931 | -0.103 |
| Cytochalasin H | 0.050 | 0.182 | 0.931 | -0.104 |
| Glu-Val | 0.000 | 0.001 | 1.227 | 0.295 |
| Isocitric acid | 0.014 | 0.078 | 1.095 | 0.131 |
| N-Acetyl-5-aminosalicylic acid | 0.002 | 0.022 | 1.847 | 0.886 |
| 2,4-Quinolinediol | 0.781 | 0.926 | 1.005 | 0.008 |
| Glu-Thr | 0.048 | 0.180 | 1.053 | 0.074 |
| Glu-Tyr | 0.007 | 0.053 | 1.157 | 0.211 |
| LPA(18:1/0:0) | 0.000 | 0.000 | 0.822 | -0.284 |
| CMPentylF | 0.065 | 0.219 | 1.135 | 0.182 |
| Barbital | 0.636 | 0.827 | 0.989 | -0.016 |
| Androsterone sulfate | 0.194 | 0.439 | 0.822 | -0.283 |
| Hydroxypiperazic acid | 0.001 | 0.015 | 0.822 | -0.282 |
| 2-Naphthalenesulfonic acid | 0.886 | 0.967 | 0.980 | -0.029 |
| 2-Deoxyribose 5'-phosphate | 0.563 | 0.772 | 1.004 | 0.006 |
| 3-(2-Naphthyl)-L-alanine | 0.495 | 0.736 | 1.004 | 0.006 |
| L-threo-3-Methylaspartate | 0.000 | 0.001 | 1.348 | 0.431 |
| FAHFA(8:0/10:0) | 0.004 | 0.038 | 1.482 | 0.567 |
| Leu-Ile | 0.850 | 0.951 | 0.978 | -0.033 |
| Docodiendioicacid | 0.954 | 0.988 | 1.039 | 0.055 |
| His-Ser | 0.633 | 0.825 | 0.822 | -0.284 |
| Phosphatidylethanolamine lyso alkenyl 18:2 | 0.700 | 0.871 | 1.050 | 0.071 |
| 2-Methylhexanoic acid | 0.522 | 0.749 | 1.067 | 0.093 |
| 3-(2-Hydroxyphenyl)propanoic acid | 0.711 | 0.876 | 0.992 | -0.011 |
| Salicyluric acid | 0.246 | 0.504 | 0.389 | -1.363 |
| 2-Octanamidoacetic acid | 0.002 | 0.027 | 0.766 | -0.384 |
| Palatinose | 0.096 | 0.293 | 1.088 | 0.122 |
| Tropine | 0.961 | 0.992 | 0.991 | -0.013 |
| M-toluene acetic acid | 0.432 | 0.701 | 0.998 | -0.003 |
| Piperic acid | 0.894 | 0.970 | 0.963 | -0.055 |
| Cholic acid | 0.365 | 0.644 | 0.851 | -0.233 |
| 7-Nitroindazole | 0.305 | 0.582 | 0.934 | -0.098 |
| 4-Methylhexanoic acid | 0.522 | 0.749 | 1.067 | 0.093 |
| 3-Methoxycatechol | 0.800 | 0.931 | 0.978 | -0.032 |
| 3,4-Dimethylbenzoic acid | 0.949 | 0.985 | 1.009 | 0.013 |
| 2,2-Dimethylpentanoic acid | 0.522 | 0.749 | 1.067 | 0.093 |
| D-Talose | 0.499 | 0.736 | 1.013 | 0.018 |
| D-Allose | 0.499 | 0.736 | 1.013 | 0.018 |
| 3,4-Dimethoxycinnamic acid | 0.793 | 0.931 | 0.823 | -0.282 |
| Naphthofluorescein | 0.663 | 0.845 | 1.021 | 0.030 |
| Zereno | 0.928 | 0.980 | 1.013 | 0.019 |
| FFA(16:1) | 0.206 | 0.454 | 1.031 | 0.044 |
| Dihydrodaidzein | 0.974 | 1.000 | 1.117 | 0.160 |
| 20-Hydroxy Prostaglandin F2α | 0.331 | 0.616 | 0.983 | -0.025 |
| Pinolenic acid | 0.031 | 0.128 | 1.237 | 0.307 |
| 15(R)-17-phenyl trinor prostaglandin F2α | 0.837 | 0.949 | 0.987 | -0.019 |
| O-1821 | 0.721 | 0.884 | 1.009 | 0.013 |
| 9,10-dihydroxystearic acid | 0.512 | 0.746 | 0.950 | -0.075 |
| (R)-3-Hydroxymyristic acid | 0.017 | 0.085 | 0.835 | -0.260 |
| 6,6'-Dihydroxy-5,5'-dimethoxybiphenyl-3,3'-dicarboxylic acid | 0.534 | 0.752 | 0.986 | -0.021 |
| 4-Oxoretinoic acid | 0.756 | 0.916 | 0.969 | -0.046 |
| N-Myristoylglycine | 0.979 | 1.000 | 0.971 | -0.043 |
| Isochodeoxycholic acid | 0.040 | 0.156 | 1.308 | 0.387 |
| Carbocyclic thromboxane A2 | 0.157 | 0.381 | 1.096 | 0.132 |
| Hydroferulic acid | 0.518 | 0.749 | 1.063 | 0.088 |
| (S)-2-Hydroxy-3-phenylpropanoic acid | 0.916 | 0.980 | 0.859 | -0.220 |
| Ethyl hydrogen malonate | 0.559 | 0.770 | 1.125 | 0.169 |
| L-Gulose | 0.540 | 0.757 | 1.025 | 0.036 |
| Deoxycholic acid | 0.716 | 0.881 | 1.224 | 0.291 |
| beta-Muricholic acid | 0.365 | 0.644 | 0.851 | -0.233 |
| Glycine deoxycholic acid | 0.512 | 0.746 | 0.726 | -0.462 |
| 3-Epideoxycholic acid | 0.070 | 0.235 | 1.386 | 0.471 |
| 5-Carboxyvanillic Acid | 0.865 | 0.956 | 0.996 | -0.006 |
| 4-Hydroxybenzoic Acid | 0.909 | 0.976 | 0.998 | -0.003 |
| Cys-Pro | 0.660 | 0.843 | 1.046 | 0.065 |
| Ser-Ala | 0.587 | 0.793 | 0.934 | -0.099 |
| Ala-Glu | 0.088 | 0.277 | 1.250 | 0.322 |
| S-Methyl-L-Cysteine-S-oxide | 0.176 | 0.412 | 0.814 | -0.297 |
| L-lyxose | 0.137 | 0.359 | 1.050 | 0.070 |
| Val-Asn | 0.416 | 0.681 | 0.891 | -0.167 |
| D-ribonate lithium salt | 0.353 | 0.640 | 1.071 | 0.099 |
| γ-Glu-Gln | 0.095 | 0.292 | 0.721 | -0.471 |
| Val-Thr | 0.136 | 0.359 | 1.099 | 0.137 |
| 2-keto-D-gluconic acid | 0.005 | 0.039 | 1.204 | 0.267 |
| Met-Asp | 0.243 | 0.503 | 1.028 | 0.039 |
| Val-Gly | 0.339 | 0.628 | 1.094 | 0.129 |
| Pyroglutamic acid | 0.884 | 0.965 | 0.982 | -0.026 |
| cyclo(gly-glu) | 0.004 | 0.035 | 1.568 | 0.649 |
| α-Hydroxyglutaric Acid (sodium salt) | 0.000 | 0.001 | 1.311 | 0.390 |
| cyclo(glu-glu) | 0.002 | 0.022 | 1.066 | 0.092 |
| Ile-Gly | 0.359 | 0.643 | 1.043 | 0.061 |
| Ile-Val | 0.896 | 0.970 | 1.012 | 0.017 |
| Asp-Leu | 0.005 | 0.043 | 1.350 | 0.433 |
| N-Acetyl-L-Glutamic Acid | 0.999 | 1.000 | 1.061 | 0.085 |
| Trp-Gly | 0.000 | 0.000 | 1.268 | 0.343 |
| 2-Hydroxy-3-Methyl Butanoic Acid | 0.778 | 0.926 | 1.056 | 0.078 |
| Salicylic acid β-D-O-glucuronic acid | 0.010 | 0.062 | 1.273 | 0.348 |
| Homovanillic Acid sulfate (sodium salt) | 0.118 | 0.328 | 1.252 | 0.325 |
| 4-Acetylaminobenzoic acid | 0.596 | 0.795 | 1.013 | 0.019 |
| Trp-Leu | 0.018 | 0.088 | 1.179 | 0.238 |
| 4-toluenesulfonic acid | 0.497 | 0.736 | 0.952 | -0.071 |
| 2,4-Dihydroxy-6-pentylbenzoic acid | 1.000 | 1.000 | 1.018 | 0.025 |
| Daidzein | 0.518 | 0.749 | 0.979 | -0.031 |
| D-Gulonic acid γ-lactone | 0.654 | 0.839 | 0.982 | -0.026 |
| L-Iditol | 0.901 | 0.970 | 1.024 | 0.034 |
| 3-Amino-5-hydroxybenzoic acid | 0.122 | 0.333 | 1.075 | 0.104 |
| 2-Methyllactic acid | 0.651 | 0.837 | 1.032 | 0.046 |
| Imidazole-4-methanol | 0.695 | 0.869 | 0.990 | -0.015 |
| (R)-2-Hydroxybutyric acid | 0.651 | 0.837 | 1.032 | 0.046 |
| 2-Methyl-3-hydroxybutyric acid | 0.045 | 0.171 | 1.086 | 0.119 |
| Pyrazine-2-carboxylic acid | 0.994 | 1.000 | 1.000 | 0.000 |
| (2s)-2-Amino-4-sulfinobutanoic acid | 0.505 | 0.743 | 0.993 | -0.011 |
| Ala-Ser | 0.994 | 1.000 | 0.907 | -0.141 |
| （2S，3R，4R，5R）-2,3,4,5,6-五羟基己醛 | 0.499 | 0.736 | 1.013 | 0.018 |
| L-rhamnonic acid | 0.934 | 0.980 | 0.976 | -0.035 |
| 3-Hydroxy-L-phenylalanine | 0.102 | 0.304 | 1.011 | 0.016 |
| Lys-Gly | 0.561 | 0.771 | 1.051 | 0.071 |
| 2-(Acetylamino)-2-deoxy-A-D-glucopyranose | 0.202 | 0.449 | 1.159 | 0.213 |
| Hyp-Thr | 0.423 | 0.692 | 1.016 | 0.023 |
| γ-Glu-Met | 0.486 | 0.736 | 1.109 | 0.150 |
| Lys-Phe | 0.020 | 0.094 | 1.069 | 0.096 |
| Met-Phe | 0.001 | 0.011 | 1.530 | 0.614 |
| Dipyrocetyl | 0.206 | 0.454 | 1.224 | 0.292 |
| 4-(Hydroxyamino)quinoline 1-oxide | 0.104 | 0.306 | 1.054 | 0.076 |
| P-Toluenesulfonamide | 0.138 | 0.359 | 1.445 | 0.532 |
| 4-Hydroxyquinoline | 0.041 | 0.160 | 1.057 | 0.080 |
| 2-Phenyl-5-benzimidazole sulfonic acid | 0.164 | 0.393 | 1.357 | 0.440 |
| 10-Hydroxystearic Acid | 0.160 | 0.385 | 0.970 | -0.043 |
| L-Glycine | 0.434 | 0.703 | 1.025 | 0.036 |
| L-Cystine | 0.476 | 0.736 | 0.903 | -0.148 |
| L-Tyrosine | 0.000 | 0.002 | 1.154 | 0.207 |
| L-Ornithine | 0.445 | 0.713 | 1.083 | 0.116 |
| L-Alanine | 0.013 | 0.074 | 1.145 | 0.195 |
| L-Histidine | 0.005 | 0.039 | 1.071 | 0.099 |
| L-Methionine | 0.656 | 0.840 | 0.952 | -0.071 |
| L-Proline | 0.008 | 0.059 | 1.175 | 0.233 |
| L-Valine | 0.012 | 0.073 | 1.156 | 0.209 |
| 5-Oxoproline | 0.704 | 0.872 | 0.950 | -0.073 |
| Betaine | 0.382 | 0.660 | 0.917 | -0.125 |
| Glyc-Pro | 0.445 | 0.713 | 0.957 | -0.063 |
| L-Cysteine | 0.377 | 0.658 | 0.994 | -0.009 |
| N6-Acetyl-L-Lysine | 0.001 | 0.018 | 1.163 | 0.217 |
| N-Acetylcysteine | 0.147 | 0.366 | 0.944 | -0.083 |
| N-Acetylputrescine | 0.400 | 0.666 | 1.016 | 0.022 |
| Serotonin | 0.737 | 0.899 | 0.986 | -0.020 |
| Trimethylamine-N-Oxide | 0.004 | 0.038 | 1.428 | 0.514 |
| Ala-Lys | 0.536 | 0.752 | 1.086 | 0.119 |
| N-Acetylhistamine | 0.534 | 0.752 | 0.932 | -0.102 |
| P-Coumaric Acid | 0.000 | 0.001 | 1.188 | 0.249 |
| 1,4-Dihydro-1-Methyl-4-Oxo-3-Pyridinecarboxamide | 0.324 | 0.607 | 1.202 | 0.266 |
| Theobromine | 0.578 | 0.785 | 1.206 | 0.270 |
| Choline | 0.122 | 0.334 | 0.955 | -0.067 |
| 1,5-Diaminopentane | 0.534 | 0.752 | 1.034 | 0.048 |
| Diethanolamine | 0.866 | 0.956 | 0.952 | -0.071 |
| Myoinositol | 0.051 | 0.182 | 0.951 | -0.072 |
| 1-Methylhistidine | 0.317 | 0.597 | 1.115 | 0.157 |
| 5,6-Dihydro-5-Methyluracil | 0.672 | 0.849 | 0.971 | -0.043 |
| 5-Methyluridine | 0.824 | 0.942 | 0.980 | -0.029 |
| Adenine | 0.011 | 0.068 | 1.141 | 0.190 |
| Cytosine | 0.683 | 0.860 | 0.919 | -0.121 |
| Purine | 0.001 | 0.018 | 1.081 | 0.112 |
| Uracil | 0.849 | 0.951 | 0.957 | -0.064 |
| 3,3',5-Triiodo-L-Thyronine | 0.726 | 0.887 | 0.999 | -0.001 |
| N-Methyltryptamine | 0.316 | 0.596 | 1.389 | 0.474 |
| D-Fructose | 0.884 | 0.965 | 1.022 | 0.031 |
| D-Mannose | 0.884 | 0.965 | 1.022 | 0.031 |
| D-Gluconic Acid | 0.842 | 0.949 | 0.974 | -0.038 |
| Orotic Acid | 0.991 | 1.000 | 1.031 | 0.044 |
| Nicotinamide | 0.243 | 0.503 | 1.149 | 0.201 |
| Riboflavin | 0.355 | 0.641 | 0.835 | -0.260 |
| Trigonelline | 0.029 | 0.122 | 1.531 | 0.614 |
| 3-Indolebutyric Acid | 0.999 | 1.000 | 1.016 | 0.022 |
| 2-Aminoethanesulfonic Acid | 0.351 | 0.637 | 0.985 | -0.021 |
| 4-Guanidinobutyric Acid | 0.116 | 0.328 | 1.104 | 0.143 |
| 5-Aminovaleric Acid | 0.851 | 0.951 | 1.048 | 0.068 |
| 6-Aminocaproic-Acid | 0.074 | 0.246 | 1.041 | 0.058 |
| 7-Methyluric Acid | 0.489 | 0.736 | 0.984 | -0.024 |
| Creatinine | 0.032 | 0.131 | 1.080 | 0.111 |
| Dl-2-Aminooctanoic Acid | 0.006 | 0.049 | 0.774 | -0.369 |
| Guanidineacetic Acid | 0.749 | 0.908 | 0.987 | -0.020 |
| L-Dihydroorotic Acid | 0.805 | 0.934 | 1.013 | 0.018 |
| L-Homoserine | 0.996 | 1.000 | 0.981 | -0.027 |
| Maleic Acid | 0.000 | 0.009 | 1.226 | 0.294 |
| LPC(0:0/14:0) | 0.000 | 0.002 | 1.402 | 0.488 |
| LPC(16:0/0:0) | 0.921 | 0.980 | 1.005 | 0.007 |
| Trans-3-Hydroxycotinine | 0.246 | 0.504 | 0.139 | -2.851 |
| L-Homoarginine | 0.014 | 0.078 | 1.215 | 0.281 |
| Pantetheine | 0.946 | 0.984 | 1.014 | 0.020 |
| D-piperidine acid | 0.704 | 0.872 | 0.950 | -0.073 |
| 5'-Deoxy-5'-(Methylthio) Adenosine | 0.006 | 0.045 | 1.188 | 0.248 |
| Sarcosine | 0.559 | 0.770 | 1.052 | 0.073 |
| Imidazoleacetic acid | 0.167 | 0.399 | 1.073 | 0.101 |
| 2-Aminoadipic Acid | 0.003 | 0.028 | 1.325 | 0.406 |
| LPC(17:0/0:0) | 0.386 | 0.660 | 1.010 | 0.015 |
| Sn-Glycero-3-Phosphocholine | 0.389 | 0.660 | 1.212 | 0.277 |
| Indole | 0.273 | 0.542 | 1.093 | 0.129 |
| LPC(15:0/0:0) | 0.001 | 0.016 | 1.140 | 0.189 |
| LPC(0:0/18:2) | 0.020 | 0.096 | 0.916 | -0.127 |
| 2-Hydroxycinnamic acid | 0.235 | 0.495 | 1.049 | 0.069 |
| Carnitine C2:0 | 0.145 | 0.364 | 0.884 | -0.178 |
| DL-Stachydrine | 0.991 | 1.000 | 1.140 | 0.189 |
| L-Norleucine | 0.004 | 0.039 | 1.057 | 0.080 |
| DL-Carnitine | 0.387 | 0.660 | 1.065 | 0.090 |
| 6-Dimethylaminopurine | 0.311 | 0.590 | 1.015 | 0.021 |
| Triethyl-phosphate | 0.401 | 0.666 | 0.951 | -0.073 |
| LPC(O-16:0/2:0) | 0.824 | 0.942 | 0.972 | -0.041 |
| 18-Hydroxycorticosterone | 0.482 | 0.736 | 1.020 | 0.028 |
| Oleamide | 0.795 | 0.931 | 0.941 | -0.088 |
| Carnitine isoC4:0 | 0.017 | 0.085 | 1.199 | 0.261 |
| 2'-Hydroxy-5'-methylacetophenone | 0.783 | 0.926 | 1.063 | 0.088 |
| Spermidine | 0.600 | 0.799 | 0.980 | -0.029 |
| N-Acetyl-L-Histidine | 0.514 | 0.747 | 1.039 | 0.056 |
| 2-Furoylglycine | 0.999 | 1.000 | 0.824 | -0.279 |
| Carnitine-2-methyl-C4 | 0.001 | 0.017 | 1.217 | 0.283 |
| Isonicotinic acid | 0.702 | 0.871 | 0.964 | -0.054 |
| Phe-Pro | 0.119 | 0.331 | 1.070 | 0.098 |
| LPE(16:1/0:0) | 0.574 | 0.784 | 0.863 | -0.212 |
| 3-Chloroaniline | 0.665 | 0.847 | 1.000 | 0.000 |
| Methylcysteine | 0.453 | 0.721 | 0.994 | -0.009 |
| DL-Leucine | 0.074 | 0.246 | 1.041 | 0.058 |
| 6-Methylnicotinamide | 0.246 | 0.504 | 1.071 | 0.098 |
| (R)-2-Hydroxy-3-phenylpropionic-acid | 0.768 | 0.922 | 0.973 | -0.039 |
| 1-Aminopropan-2-ol | 0.004 | 0.038 | 1.428 | 0.514 |
| N-Methylalanine | 0.810 | 0.937 | 1.036 | 0.052 |
| Dihydro-D-sphingosine | 0.616 | 0.809 | 1.028 | 0.040 |
| Hypaphorine | 0.370 | 0.650 | 1.004 | 0.005 |
| N-Methyl-L-Glutamate | 0.003 | 0.028 | 1.325 | 0.406 |
| Phosphocholine | 0.622 | 0.814 | 1.008 | 0.011 |
| 2,4-Dihydroxypteridine | 0.023 | 0.109 | 1.117 | 0.160 |
| Cortisol | 0.063 | 0.215 | 0.870 | -0.201 |
| L-Tryptophanamide | 0.018 | 0.088 | 1.070 | 0.097 |
| Catechol | 0.805 | 0.934 | 0.999 | -0.002 |
| Acetylcholine | 0.594 | 0.794 | 1.050 | 0.071 |
| 1-Hydroxylamino-2-phenylethane | 0.002 | 0.022 | 1.082 | 0.114 |
| β-Alanine | 0.013 | 0.074 | 1.145 | 0.195 |
| D-(+)-sucrose | 0.832 | 0.947 | 1.133 | 0.180 |
| Thr-Phe | 0.177 | 0.412 | 1.194 | 0.256 |
| Salicylaldehyde | 0.394 | 0.663 | 0.960 | -0.059 |
| Benzaldehyde | 0.001 | 0.019 | 1.093 | 0.129 |
| Biliverdin | 0.797 | 0.931 | 1.061 | 0.086 |
| N,-N-diacetyl-O-methylhydroxylamine | 0.113 | 0.325 | 0.949 | -0.075 |
| Hydroxyquinoline | 0.006 | 0.045 | 1.090 | 0.125 |
| DL-O-tyrosine | 0.001 | 0.017 | 1.044 | 0.061 |
| 7-Methylguanosine | 0.297 | 0.572 | 1.015 | 0.021 |
| 6-O-methylguanine | 0.649 | 0.837 | 0.985 | -0.022 |
| Oxypurinol | 0.002 | 0.022 | 1.396 | 0.481 |
| Carnitine C12:0 | 0.157 | 0.381 | 0.906 | -0.142 |
| Allopurinol | 0.014 | 0.078 | 2.299 | 1.201 |
| PC(12:0/12:0) | 0.462 | 0.731 | 0.994 | -0.009 |
| (R)-(-)-2-phenylglycine | 0.002 | 0.022 | 1.082 | 0.114 |
| 1-acetylindole | 0.025 | 0.113 | 1.047 | 0.066 |
| 3-Carboxypropyltrimethylammonium | 0.559 | 0.770 | 1.116 | 0.158 |
| L-Isoleucine | 0.011 | 0.068 | 1.081 | 0.113 |
| LPC(O-18:0/0:0) | 0.464 | 0.731 | 0.929 | -0.106 |
| Creatine phosphate | 0.688 | 0.863 | 0.985 | -0.021 |
| PC(O-16:0/O-2:0) | 0.464 | 0.731 | 0.929 | -0.106 |
| Butenoyl-PAF | 0.000 | 0.002 | 0.838 | -0.255 |
| N-Methyl-α-aminoisobutyric acid | 0.911 | 0.977 | 1.090 | 0.124 |
| Biotinamide | 0.126 | 0.338 | 1.131 | 0.177 |
| 8,8a-deoxy-oleane | 0.700 | 0.871 | 1.006 | 0.008 |
| N-acetylpyrrolidine | 0.709 | 0.876 | 0.964 | -0.054 |
| 1,3-Dicyclohexylurea | 0.764 | 0.921 | 1.022 | 0.032 |
| PC(O-16:0/O-1:0) | 0.781 | 0.926 | 0.995 | -0.007 |
| 4-tert-butylbenzoic-acid | 0.790 | 0.931 | 1.001 | 0.001 |
| 4-Hydroxytryptamine | 0.162 | 0.389 | 0.125 | -3.002 |
| 1-Deoxyvaleric-acid | 0.984 | 1.000 | 1.002 | 0.003 |
| 2-Mercaptobenzothiazole | 0.144 | 0.362 | 0.913 | -0.131 |
| Urobilin | 0.478 | 0.736 | 1.018 | 0.026 |
| 20,26-dihydroxyecdysone | 0.842 | 0.949 | 0.990 | -0.014 |
| Carnitine C6:0 | 0.964 | 0.993 | 0.900 | -0.152 |
| Leu-Gly | 0.105 | 0.309 | 1.078 | 0.109 |
| LPC(0:0/22:4) | 0.009 | 0.062 | 0.855 | -0.226 |
| LPC(22:4/0:0) | 0.009 | 0.062 | 0.855 | -0.226 |
| LPC(0:0/22:5) | 0.101 | 0.301 | 0.855 | -0.226 |
| LPC(22:5/0:0) | 0.398 | 0.666 | 1.060 | 0.084 |
| LPC(20:1/0:0) | 0.000 | 0.002 | 0.838 | -0.255 |
| LPC(20:2/0:0) | 0.226 | 0.486 | 0.948 | -0.078 |
| LPC(0:0/20:2) | 0.226 | 0.486 | 0.948 | -0.078 |
| LPC(0:0/20:3) | 0.362 | 0.644 | 1.138 | 0.187 |
| LPC(20:3/0:0) | 0.362 | 0.644 | 1.138 | 0.187 |
| LPC(0:0/20:4) | 0.797 | 0.931 | 1.012 | 0.017 |
| LPC(20:4/0:0) | 0.797 | 0.931 | 1.012 | 0.017 |
| LPC(18:0/0:0) | 0.824 | 0.942 | 0.972 | -0.041 |
| LPC(18:1/0:0) | 0.000 | 0.001 | 0.837 | -0.256 |
| LPC(18:2/0:0) | 0.020 | 0.096 | 0.916 | -0.127 |
| LPC(0:0/16:0) | 0.921 | 0.980 | 1.005 | 0.007 |
| LPC(16:1/0:0) | 0.576 | 0.784 | 0.986 | -0.020 |
| Mycosporine-glycine | 0.904 | 0.972 | 1.005 | 0.007 |
| Carnitine C18:0 | 0.237 | 0.495 | 0.919 | -0.122 |
| Carnitine C18:2 | 0.091 | 0.283 | 1.242 | 0.313 |
| Carnitine C16:0 | 0.800 | 0.931 | 1.008 | 0.012 |
| Carnitine C16:1 | 0.011 | 0.065 | 0.836 | -0.259 |
| Carnitine C16:2 | 0.899 | 0.970 | 1.015 | 0.022 |
| Carnitine C14-OH | 0.049 | 0.182 | 0.834 | -0.262 |
| Carnitine C14:2-OH | 0.749 | 0.908 | 0.967 | -0.049 |
| Carnitine C14:2 | 0.530 | 0.752 | 1.005 | 0.007 |
| Carnitine C12-OH | 0.064 | 0.219 | 0.908 | -0.140 |
| Carnitine C11:DC | 0.083 | 0.268 | 0.922 | -0.117 |
| Carnitine C13:1 | 0.480 | 0.736 | 1.050 | 0.070 |
| Carnitine C11:0 | 0.275 | 0.545 | 1.210 | 0.275 |
| Carnitine C11:1 | 0.946 | 0.984 | 0.968 | -0.046 |
| Carnitine C10:0 | 0.225 | 0.486 | 0.852 | -0.231 |
| Carnitine C8-OH | 0.266 | 0.538 | 0.833 | -0.263 |
| Carnitine C9:0 | 0.230 | 0.489 | 1.156 | 0.209 |
| Carnitine C8:0 | 0.624 | 0.816 | 0.860 | -0.218 |
| Carnitine C8:1 | 0.000 | 0.007 | 2.138 | 1.096 |
| Carnitine ph-C1 | 0.235 | 0.495 | 1.017 | 0.024 |
| Carnitine C4:DC | 0.766 | 0.922 | 0.967 | -0.049 |
| Carnitine C5:0 | 0.001 | 0.017 | 1.217 | 0.283 |
| Carnitine C5:1 | 0.063 | 0.215 | 1.166 | 0.221 |
| Carnitine C4:0 | 0.017 | 0.085 | 1.199 | 0.261 |
| 1,3-Diphenylguanidine | 0.693 | 0.867 | 1.011 | 0.016 |
| N'-Methyl-2-pyridone-5-carboxamide | 0.001 | 0.019 | 1.282 | 0.358 |
| 5-Methoxytryptamine | 0.202 | 0.449 | 1.029 | 0.041 |
| L-Phenylephrine | 0.594 | 0.794 | 1.022 | 0.032 |
| Theophylline | 0.587 | 0.793 | 2.702 | 1.434 |
| SDMA | 0.936 | 0.980 | 1.032 | 0.046 |
| Methylguanidine | 0.616 | 0.809 | 1.016 | 0.023 |
| N6-methyladenosine | 0.493 | 0.736 | 1.047 | 0.066 |
| 8-Azaguanine | 0.002 | 0.022 | 1.396 | 0.481 |
| Isocytosine | 0.683 | 0.860 | 0.919 | -0.121 |
| Leu-Val | 0.889 | 0.967 | 1.015 | 0.021 |
| Phe-Asn | 0.260 | 0.529 | 1.032 | 0.045 |
| Phe-Met | 0.000 | 0.003 | 1.422 | 0.508 |
| Glu-Met | 0.470 | 0.733 | 1.055 | 0.077 |
| Met-Glu | 0.403 | 0.667 | 1.067 | 0.094 |
| Phe-Val | 0.007 | 0.053 | 1.166 | 0.222 |
| Carnitine C10:1 | 0.307 | 0.584 | 1.104 | 0.142 |
| Carnitine C14:2:DC | 0.012 | 0.072 | 0.828 | -0.273 |
| Caldine | 0.278 | 0.548 | 0.938 | -0.093 |
| 1-Methyladenosine | 0.493 | 0.736 | 1.047 | 0.066 |
| PC(O-16:0/0:0) | 0.576 | 0.784 | 1.041 | 0.057 |
| LPC(O-18:1/0:0) | 0.037 | 0.149 | 0.934 | -0.098 |
| LPC(O-0:0/18:0) | 0.464 | 0.731 | 0.929 | -0.106 |
| NE,NE,NE-TRIMETHYLLYSINE | 0.000 | 0.002 | 1.149 | 0.200 |
| N,N-Dimethylarginine | 0.382 | 0.660 | 1.033 | 0.046 |
| Carnitine C3:0 | 0.091 | 0.282 | 1.145 | 0.195 |
| Phe-Glu | 0.273 | 0.542 | 1.132 | 0.179 |
| Gly-Gly-Phe | 0.093 | 0.288 | 1.075 | 0.105 |
| LPC(0:0/17:0) | 0.436 | 0.705 | 1.012 | 0.017 |
| LPC(0:0/15:0) | 0.001 | 0.016 | 1.140 | 0.189 |
| LPC(12:0/0:0) | 0.025 | 0.113 | 1.256 | 0.329 |
| LPC(O-16:1/0:0) | 0.240 | 0.498 | 0.943 | -0.084 |
| Sphingosyl-phosphocholine | 0.117 | 0.328 | 1.071 | 0.099 |
| LPC(0:0/16:1) | 0.576 | 0.784 | 0.986 | -0.020 |
| LPC(0:0/18:0) | 0.824 | 0.942 | 0.972 | -0.041 |
| LPC(0:0/18:1) | 0.000 | 0.001 | 0.837 | -0.256 |
| LPC(0:0/20:1) | 0.000 | 0.002 | 0.838 | -0.255 |
| Methyldopa | 0.180 | 0.415 | 0.874 | -0.194 |
| (E)-Guggulsterone | 0.048 | 0.180 | 1.081 | 0.112 |
| (Z)-Guggulsterone | 0.048 | 0.180 | 1.081 | 0.112 |
| 1-Methylinosine | 0.311 | 0.590 | 1.065 | 0.091 |
| 2'-O-methylcytidine | 0.273 | 0.542 | 1.236 | 0.305 |
| Acrylamide | 0.000 | 0.006 | 1.150 | 0.202 |
| Agmatine | 0.936 | 0.980 | 1.003 | 0.005 |
| Ala-Phe | 0.004 | 0.038 | 1.091 | 0.126 |
| Cyromazine | 0.344 | 0.632 | 0.911 | -0.134 |
| Cytidine 5'-diphosphate | 0.347 | 0.636 | 0.927 | -0.109 |
| L-Allothreonine | 0.996 | 1.000 | 0.981 | -0.027 |
| D-Glucosaminic acid | 0.169 | 0.400 | 0.863 | -0.213 |
| Guanidine | 0.270 | 0.542 | 0.990 | -0.014 |
| Leu-Ala | 0.651 | 0.837 | 1.045 | 0.064 |
| L-Norvaline | 0.012 | 0.073 | 1.156 | 0.209 |
| Leu-Phe | 0.667 | 0.848 | 0.839 | -0.254 |
| N4-Acetylcytidine | 0.182 | 0.415 | 1.096 | 0.132 |
| Gly-Ile | 0.046 | 0.175 | 1.289 | 0.366 |
| Palmitoylethanolamide（(PEA） | 0.016 | 0.084 | 1.124 | 0.169 |
| Sanguinarine | 0.926 | 0.980 | 1.041 | 0.058 |
| Stachydrine | 0.638 | 0.828 | 1.019 | 0.027 |
| Synephrine | 0.788 | 0.931 | 1.035 | 0.049 |
| Bicine | 0.718 | 0.883 | 0.991 | -0.013 |
| Eicosanoyl-EA | 0.287 | 0.560 | 0.907 | -0.141 |
| Gln-Phe | 0.051 | 0.182 | 1.077 | 0.107 |
| Ile-Met | 0.000 | 0.000 | 1.346 | 0.428 |
| LPE(17:1/0:0) | 0.879 | 0.964 | 0.937 | -0.094 |
| Pro-Ile | 0.001 | 0.017 | 1.446 | 0.532 |
| Ser-Leu | 0.374 | 0.653 | 1.074 | 0.103 |
| Ser-Phe | 0.220 | 0.481 | 0.997 | -0.004 |
| Tyr-Leu | 0.153 | 0.378 | 1.256 | 0.329 |
| 3-Hydroxyphenylurea | 0.386 | 0.660 | 0.971 | -0.042 |
| Caffeine | 0.879 | 0.964 | 2.861 | 1.516 |
| Carnitine C9:1-OH | 0.004 | 0.035 | 2.209 | 1.144 |
| LPE(18:2/0:0) | 0.060 | 0.211 | 0.896 | -0.159 |
| (E,Z)-2-Amino-3,14-octadecadien-1-ol | 0.795 | 0.931 | 0.941 | -0.088 |
| Phe-Trp | 0.274 | 0.543 | 0.993 | -0.009 |
| N(Alpha)-Acetyl-Epsilon-(2-Propenal)Lysine | 0.021 | 0.098 | 1.485 | 0.570 |
| Cyclo(Pro-Leu) | 0.004 | 0.035 | 1.307 | 0.387 |
| Thr-Gln | 0.267 | 0.540 | 1.365 | 0.449 |
| Cyclo(Phe-Glu) | 0.336 | 0.624 | 1.142 | 0.192 |
| Cyclo(Pro-Val) | 0.001 | 0.017 | 1.198 | 0.261 |
| Ile-Asp | 0.005 | 0.039 | 1.589 | 0.668 |
| N-MethyTrans-4-Hydroxy-Proline | 0.179 | 0.414 | 1.281 | 0.357 |
| Ser-Ile | 0.374 | 0.653 | 1.074 | 0.103 |
| 5-Aminoimidazole ribonucleotide | 0.236 | 0.495 | 1.072 | 0.100 |
| N6-(2-Hydroxyethyl)adenosine | 0.054 | 0.191 | 1.091 | 0.126 |
| 7-(alpha-D-glucosyl)-N(6)-isopentenyladenine | 0.354 | 0.640 | 1.281 | 0.357 |
| D-Proline-betaine | 0.672 | 0.849 | 0.917 | -0.125 |
| 2-Methyl-1-Pyrroline | 0.359 | 0.643 | 1.048 | 0.068 |
| Melibiose | 0.137 | 0.359 | 1.073 | 0.102 |
| N-Formylglycine | 0.449 | 0.716 | 0.980 | -0.028 |
| D-Ornithine | 0.822 | 0.942 | 1.021 | 0.030 |
| Dehydroascorbic-acid | 0.144 | 0.362 | 0.940 | -0.089 |
| Androstenediol | 0.854 | 0.951 | 1.000 | 0.000 |
| Triethylenetetramine | 0.427 | 0.695 | 0.984 | -0.023 |
| FFA(10:1) | 0.484 | 0.736 | 0.847 | -0.240 |
| 3-Aminoquinoline | 0.012 | 0.070 | 1.075 | 0.104 |
| L-Isserine | 0.144 | 0.362 | 0.933 | -0.100 |
| 1-Amino-1-cyclobutane-carboxylic-acid | 0.008 | 0.059 | 1.175 | 0.233 |
| N,N-Bis(2-hydroxyethyl)dodecanamide | 0.800 | 0.931 | 1.040 | 0.056 |
| (E)-8-Methyl-6-nonenoic-acid | 0.740 | 0.901 | 0.988 | -0.017 |
| Cork-oximate | 0.464 | 0.731 | 1.094 | 0.129 |
| 8-iso-15-keto-Prostaglandin-F2α | 0.941 | 0.983 | 1.068 | 0.095 |
| 2-Amino-3-phosphonopropionic-acid | 0.827 | 0.942 | 0.973 | -0.040 |
| Inositol 1,3,4-trisphosphate | 0.514 | 0.747 | 1.005 | 0.008 |
| S-methyl-L-thiocitrulline | 0.488 | 0.736 | 0.984 | -0.023 |
| ST-638 | 0.281 | 0.552 | 0.855 | -0.226 |
| Leu-Leu | 0.817 | 0.940 | 1.070 | 0.097 |
| Gly-Lys | 0.934 | 0.980 | 1.028 | 0.040 |
| Gly-Gln | 0.084 | 0.271 | 1.089 | 0.123 |
| Lys-Ser | 0.561 | 0.771 | 1.011 | 0.015 |
| Arg-Glu | 0.268 | 0.541 | 1.214 | 0.280 |
| Glu-His | 0.016 | 0.083 | 1.191 | 0.252 |
| Cyclocreatine | 0.532 | 0.752 | 1.041 | 0.058 |
| γ-Glu-Lys | 0.045 | 0.172 | 0.780 | -0.359 |
| (R)-(-)-1-Amino-2-propanol | 0.003 | 0.034 | 1.293 | 0.371 |
| Gly-Thr | 0.292 | 0.568 | 1.031 | 0.044 |
| Pro-Ser | 0.842 | 0.949 | 0.962 | -0.055 |
| N-Ethylglycine | 0.810 | 0.937 | 1.036 | 0.052 |
| 3-Guanidinopropionic acid | 0.493 | 0.736 | 0.985 | -0.022 |
| Glu-Ser | 0.325 | 0.609 | 1.043 | 0.061 |
| Ammeline | 0.470 | 0.733 | 0.910 | -0.136 |
| Thr-Glu | 0.024 | 0.112 | 1.221 | 0.289 |
| Glu-Cit | 0.609 | 0.808 | 0.904 | -0.146 |
| Glu-Gly | 0.141 | 0.359 | 0.874 | -0.194 |
| N-acetyl-D-Lactosamine | 0.227 | 0.488 | 1.077 | 0.107 |
| (+/-)-High-Proline | 0.613 | 0.809 | 1.073 | 0.102 |
| 3-(imidazol-4-yl)propionic-acid | 0.501 | 0.738 | 1.015 | 0.021 |
| Ser-Pro | 0.099 | 0.299 | 1.058 | 0.082 |
| N1-Acetylspermidine | 0.839 | 0.949 | 0.970 | -0.044 |
| Ser-Val | 0.379 | 0.658 | 0.966 | -0.050 |
| Ile-Gln | 0.029 | 0.122 | 1.364 | 0.448 |
| Ile-Ser | 0.050 | 0.182 | 1.201 | 0.264 |
| Tyr-Glu | 0.256 | 0.523 | 0.996 | -0.006 |
| Ile-Glu | 0.103 | 0.305 | 1.457 | 0.543 |
| Leu-Glu | 0.103 | 0.305 | 1.457 | 0.543 |
| Glu-Ile | 0.103 | 0.305 | 1.457 | 0.543 |
| Ile-Thr | 0.086 | 0.273 | 1.336 | 0.418 |
| Leu-Thr | 0.086 | 0.273 | 1.336 | 0.418 |
| 2,2'-Cyclouridine | 0.891 | 0.968 | 1.000 | 0.000 |
| 3-aminobenzamide | 0.138 | 0.359 | 1.315 | 0.395 |
| N-(2-hydroxyethyl)-3-pyridinecarboxamide | 0.043 | 0.167 | 1.037 | 0.052 |
| Phe-Ala-Ser | 0.025 | 0.113 | 1.379 | 0.464 |
| Ser-Phe-Ala | 0.025 | 0.113 | 1.379 | 0.464 |
| 1-Methylguanosine | 0.297 | 0.572 | 1.015 | 0.021 |
| Cyclo(Ala-Pro) | 0.005 | 0.039 | 1.091 | 0.126 |
| Phe-Gly | 0.926 | 0.980 | 1.037 | 0.052 |
| Val-Ile | 0.169 | 0.400 | 1.125 | 0.170 |
| Val-Leu | 0.169 | 0.400 | 1.125 | 0.170 |
| Asp-Ile | 0.005 | 0.039 | 1.589 | 0.668 |
| Leu-Met | 0.000 | 0.000 | 1.346 | 0.428 |
| cyclo(pro-pro) | 0.045 | 0.171 | 1.193 | 0.255 |
| Phe-Tyr | 0.029 | 0.124 | 1.544 | 0.626 |
| Ile-Leu | 0.817 | 0.940 | 1.070 | 0.097 |
| Phe-Ala-Leu | 0.070 | 0.236 | 0.873 | -0.195 |
| Securinine | 0.944 | 0.983 | 0.996 | -0.006 |
| Thr-Arg | 0.795 | 0.931 | 1.031 | 0.044 |
| Arg-Thr | 0.489 | 0.736 | 0.957 | -0.063 |
| Leu-Asn | 0.016 | 0.083 | 1.124 | 0.169 |
| Glu-Phe-Ala | 0.015 | 0.081 | 1.141 | 0.191 |
| 4-Amino-3-hydroxybutyric acid | 0.365 | 0.644 | 1.022 | 0.032 |
| D-Allo-Isoleucine | 0.011 | 0.068 | 1.081 | 0.113 |
| 5-Acetylamino-6-amino-3-methyluracil | 0.928 | 0.980 | 0.998 | -0.003 |
| Gln-Gly | 0.401 | 0.666 | 1.069 | 0.096 |
| Gly-Glu | 0.141 | 0.359 | 0.874 | -0.194 |
| Lys-Ala | 0.536 | 0.752 | 1.086 | 0.119 |
| Pro-Asn | 0.152 | 0.377 | 1.095 | 0.131 |
| Pro-Asp | 0.008 | 0.056 | 1.246 | 0.318 |
| Arg-Gly | 0.230 | 0.489 | 0.938 | -0.093 |
| Gly-Arg | 0.230 | 0.489 | 0.938 | -0.093 |
| Ser-Lys | 0.761 | 0.919 | 1.051 | 0.071 |
| Ser-Glu | 0.991 | 1.000 | 0.933 | -0.100 |
| Phe-Ala | 0.010 | 0.065 | 1.068 | 0.095 |
| Ile-Asn | 0.016 | 0.083 | 1.124 | 0.169 |
| Leu-Asp | 0.000 | 0.010 | 1.523 | 0.607 |
| Phe-Thr | 0.177 | 0.412 | 1.194 | 0.256 |
| Phe-Hyp | 0.482 | 0.736 | 1.014 | 0.020 |
| Glu-Phe | 0.273 | 0.542 | 1.132 | 0.179 |
| Glu-Arg | 0.026 | 0.115 | 1.241 | 0.311 |
| 2-Butyl-3-(4-hydroxybenzoyl)benzofuran | 0.000 | 0.000 | 0.638 | -0.649 |
| Quinmerac | 0.313 | 0.591 | 0.984 | -0.023 |
| Chaps | 0.394 | 0.663 | 0.749 | -0.417 |
| trans-resveratrol-3-O-sulfate | 0.526 | 0.752 | 0.972 | -0.041 |

**Table S9**. 839 Metabolites comparisions between group 4 trajectory and group 1 trajectory in males.

| Compounds | P_value | P_adjusted | FC | log2FC |
| --- | --- | --- | --- | --- |
| 3-carboxy-4-methyl-5-propyl-2-furanpropionic acid | 0.093 | 0.257 | 1.493 | 0.578 |
| 2,4-diacetamino-2,4,6-triphenoxy-D-mannopyranose | 0.002 | 0.017 | 1.737 | 0.797 |
| L-Threonine | 0.879 | 0.944 | 0.932 | -0.101 |
| L-Arginine | 0.051 | 0.172 | 1.103 | 0.142 |
| L-Aspartic Acid | 0.072 | 0.217 | 1.207 | 0.272 |
| L-Citrulline | 0.156 | 0.351 | 0.891 | -0.166 |
| L-Glutamic Acid | 0.000 | 0.000 | 1.421 | 0.507 |
| L-Phenylalanine | 0.000 | 0.001 | 1.117 | 0.160 |
| L-Serine | 0.816 | 0.910 | 0.981 | -0.027 |
| L-Tryptophan | 0.002 | 0.017 | 1.142 | 0.191 |
| (5-L-Glutamyl)-L-Amino Acid | 0.502 | 0.737 | 1.048 | 0.068 |
| Allantoin | 0.700 | 0.853 | 0.888 | -0.171 |
| Asp-Phe | 0.151 | 0.342 | 1.428 | 0.514 |
| Glutathione Oxidized | 0.668 | 0.829 | 0.945 | -0.081 |
| Hexanoyl Glycine | 0.041 | 0.144 | 0.679 | -0.559 |
| L-Asparagine Anhydrous | 0.343 | 0.587 | 0.924 | -0.114 |
| L-Glutamine | 0.000 | 0.006 | 0.804 | -0.314 |
| L-Homocitrulline | 0.574 | 0.784 | 1.043 | 0.061 |
| L-Theanine | 0.106 | 0.278 | 0.928 | -0.107 |
| N-Acetylaspartate | 0.602 | 0.793 | 0.963 | -0.054 |
| N-Acetyl-L-Leucine | 0.036 | 0.131 | 1.148 | 0.199 |
| N-Acetyl-L-Tyrosine | 0.082 | 0.240 | 0.967 | -0.048 |
| N-Acetylneuraminic Acid(SA) | 0.602 | 0.793 | 1.044 | 0.063 |
| Gly-Leu | 0.196 | 0.398 | 1.390 | 0.476 |
| N-Isovaleroylglycine | 0.258 | 0.470 | 1.275 | 0.351 |
| N-Propionylglycine | 0.000 | 0.001 | 1.168 | 0.224 |
| Nα-Acetyl-L-Arginine | 0.025 | 0.097 | 1.250 | 0.322 |
| O-Phospho-L-Serine | 0.230 | 0.436 | 0.872 | -0.197 |
| Phenylacetyl-L-Glutamine | 0.952 | 0.977 | 1.054 | 0.077 |
| Phe-Phe | 0.080 | 0.237 | 1.232 | 0.301 |
| S-(5-Adenosy)-L-Homocysteine | 0.452 | 0.698 | 1.076 | 0.105 |
| S-Sulfo-L-Cysteine | 0.181 | 0.383 | 1.081 | 0.113 |
| Trans-4-Hydroxy-L-Proline | 0.000 | 0.001 | 1.168 | 0.224 |
| γ-L-Glutamate-Cysteine | 0.402 | 0.646 | 0.993 | -0.010 |
| N-Acetyl-L-phenylalanine | 0.184 | 0.387 | 1.018 | 0.026 |
| Benzoylformic Acid | 0.543 | 0.767 | 0.934 | -0.099 |
| 3-Hydroxyanthranilic Acid | 0.062 | 0.194 | 1.205 | 0.269 |
| P–Hydroxyphenyl Acetic Acid | 0.574 | 0.784 | 1.087 | 0.120 |
| 2-Picolinic Acid | 0.175 | 0.378 | 1.014 | 0.021 |
| 4-Pyridoxic Acid | 0.028 | 0.106 | 1.219 | 0.286 |
| 6-Hydroxynicotinic Acid | 0.655 | 0.818 | 1.202 | 0.265 |
| Taurocholic acid | 0.861 | 0.929 | 1.007 | 0.010 |
| Taurochenodesoxycholic Acid | 0.689 | 0.844 | 0.895 | -0.160 |
| Glycolithocholic acid | 0.852 | 0.922 | 0.943 | -0.085 |
| Hyodeoxycholic acid | 0.315 | 0.554 | 1.028 | 0.041 |
| Glycoursodeoxycholic Acid | 0.606 | 0.795 | 0.814 | -0.297 |
| Glycochenodeoxycholic Acid | 0.660 | 0.821 | 0.710 | -0.494 |
| Chenodeoxycholic Acid | 0.956 | 0.979 | 1.140 | 0.189 |
| 4-Methylcatechol | 0.346 | 0.589 | 0.742 | -0.430 |
| 4-Hydroxy-3-methoxybenzaldehyde | 0.578 | 0.785 | 0.952 | -0.071 |
| 1,7-Dimethylxanthine | 0.215 | 0.420 | 2.883 | 1.527 |
| 1-Methylxanthine | 0.892 | 0.944 | 0.902 | -0.150 |
| Xanthine | 0.065 | 0.200 | 1.201 | 0.264 |
| 3-Methylxanthine | 0.892 | 0.944 | 0.902 | -0.150 |
| 5-Methylcytosine | 0.702 | 0.853 | 1.008 | 0.011 |
| 7-Methylxanthine | 0.892 | 0.944 | 0.902 | -0.150 |
| Guanosine | 0.435 | 0.681 | 1.325 | 0.406 |
| Hypoxanthine | 0.970 | 0.983 | 0.991 | -0.013 |
| Uridine | 0.038 | 0.136 | 1.088 | 0.122 |
| L-Thyroxine | 0.643 | 0.811 | 0.931 | -0.103 |
| Norepinephrine | 0.594 | 0.793 | 0.977 | -0.034 |
| Succinic Acid | 0.003 | 0.021 | 1.175 | 0.232 |
| Cis-Aconitic Acid | 0.651 | 0.818 | 1.077 | 0.108 |
| Melatonin | 0.842 | 0.920 | 1.000 | 0.000 |
| Tryptamine | 0.005 | 0.029 | 1.078 | 0.109 |
| D-Glucose | 0.838 | 0.918 | 0.976 | -0.035 |
| D-Trehalose | 0.715 | 0.854 | 1.028 | 0.040 |
| D-Glucose 6-Phosphate | 0.002 | 0.015 | 1.274 | 0.350 |
| Lactose | 0.715 | 0.854 | 1.028 | 0.040 |
| Lactulose | 0.715 | 0.854 | 1.028 | 0.040 |
| L-Fucose | 0.104 | 0.275 | 0.755 | -0.406 |
| Maltose | 0.715 | 0.854 | 1.028 | 0.040 |
| D-Glucoronic Acid | 0.017 | 0.075 | 1.251 | 0.323 |
| Pantothenate | 0.001 | 0.006 | 1.199 | 0.262 |
| 3-Indolepropionic Acid | 0.329 | 0.573 | 0.930 | -0.105 |
| Indole-3-Carboxaldehyde | 0.164 | 0.362 | 0.866 | -0.208 |
| Methyl Indole-3-Acetate | 0.820 | 0.911 | 1.023 | 0.033 |
| 2-Hydroxybutanoic Acid | 0.118 | 0.294 | 0.636 | -0.652 |
| 2-Hydroxyisocaproic Acid | 0.023 | 0.090 | 1.159 | 0.213 |
| 2-Methylsuccinic Acid | 0.151 | 0.342 | 1.353 | 0.437 |
| 3-Hydroxy-3-Methyl Butyric Acid | 0.399 | 0.643 | 0.945 | -0.082 |
| 3-Methylcrotonyl Glycine | 0.149 | 0.342 | 1.190 | 0.251 |
| 4-Hydroxy-2-Oxoglutaric Acid | 0.001 | 0.008 | 1.219 | 0.286 |
| Adipic Acid | 0.030 | 0.113 | 0.889 | -0.170 |
| Azelaic Acid | 0.668 | 0.829 | 0.955 | -0.066 |
| Caffeic Acid | 0.088 | 0.251 | 0.716 | -0.481 |
| Creatine | 0.092 | 0.256 | 1.366 | 0.450 |
| Dodecanedioic Aicd | 0.001 | 0.009 | 0.834 | -0.262 |
| Glutaric Acid | 0.151 | 0.342 | 1.353 | 0.437 |
| Guanidinoethyl Sulfonate | 0.567 | 0.778 | 0.988 | -0.017 |
| Hippuric Acid | 0.114 | 0.288 | 0.653 | -0.614 |
| Hydrocinnamic Acid | 0.041 | 0.144 | 0.681 | -0.554 |
| L-kynurenine | 0.002 | 0.015 | 1.115 | 0.156 |
| Kynurenic Acid | 0.000 | 0.005 | 1.269 | 0.344 |
| L-Lactic Acid | 0.506 | 0.739 | 1.122 | 0.166 |
| Malonicacid | 0.100 | 0.267 | 0.639 | -0.646 |
| Mandelic Acid | 0.574 | 0.784 | 1.087 | 0.120 |
| Methylmalonic Acid | 0.003 | 0.021 | 1.175 | 0.232 |
| Phenyllactate(Pla) | 0.182 | 0.386 | 1.155 | 0.208 |
| Pyrrole-2-Carboxylic Acid | 0.571 | 0.782 | 1.101 | 0.138 |
| Sebacate | 0.392 | 0.641 | 0.972 | -0.042 |
| Shikimic Acid | 0.018 | 0.075 | 1.469 | 0.555 |
| Subericacid | 0.377 | 0.618 | 0.978 | -0.032 |
| TXB2 | 0.924 | 0.957 | 1.105 | 0.144 |
| (±)15-HETE | 0.020 | 0.083 | 1.488 | 0.573 |
| LPG(18:1/0:0) | 0.547 | 0.767 | 0.992 | -0.012 |
| LPE(18:1/0:0) | 0.011 | 0.053 | 0.766 | -0.385 |
| LPE(18:0/0:0) | 0.015 | 0.071 | 1.293 | 0.371 |
| LPE(16:0/0:0) | 0.098 | 0.264 | 1.140 | 0.189 |
| LPE(14:0/0:0) | 0.008 | 0.042 | 1.335 | 0.417 |
| LPA(0:0/18:0) | 0.202 | 0.404 | 1.022 | 0.031 |
| LPA(0:0/16:0) | 0.059 | 0.186 | 1.052 | 0.073 |
| LipoxinA4 | 0.754 | 0.871 | 0.891 | -0.166 |
| 13-HOTrE | 0.000 | 0.002 | 2.488 | 1.315 |
| 9,10-DiHOME | 0.030 | 0.113 | 0.804 | -0.315 |
| FFA(18:3) | 0.000 | 0.001 | 1.577 | 0.657 |
| FFA(16:0) | 0.016 | 0.073 | 1.113 | 0.155 |
| FFA(18:2) | 0.033 | 0.123 | 1.129 | 0.175 |
| FFA(12:0) | 0.442 | 0.689 | 0.956 | -0.065 |
| FFA(18:1) | 0.578 | 0.785 | 1.055 | 0.078 |
| EPA | 0.000 | 0.000 | 1.800 | 0.848 |
| FFA(20:2) | 0.179 | 0.383 | 1.172 | 0.229 |
| FFA(10:0) | 0.181 | 0.383 | 0.176 | -2.509 |
| AA | 0.012 | 0.060 | 1.123 | 0.167 |
| Urocanic Acid | 0.008 | 0.043 | 1.212 | 0.277 |
| 4-Hydroxybenzaldehyde | 0.647 | 0.815 | 0.986 | -0.020 |
| Neopterin | 0.449 | 0.693 | 1.011 | 0.016 |
| Ethylmalonate | 0.194 | 0.398 | 1.238 | 0.308 |
| 2-(Formylamino)Benzoic Acid | 0.710 | 0.854 | 1.005 | 0.007 |
| Ureidoisobutyric Acid | 0.198 | 0.401 | 1.276 | 0.352 |
| Uridine 5-Monophosphate | 0.635 | 0.811 | 1.061 | 0.085 |
| N-Acetylglycine | 0.000 | 0.002 | 0.695 | -0.526 |
| 3-Hydroxyhippuric Acid | 0.906 | 0.949 | 1.080 | 0.111 |
| 2-(Dimethylamino)Guanosine | 0.184 | 0.387 | 1.117 | 0.159 |
| 5-Hydroxyhexanoic Acid | 0.023 | 0.090 | 1.159 | 0.213 |
| Β-Pseudouridine | 0.367 | 0.607 | 1.121 | 0.165 |
| N-Acetylthreonine | 0.749 | 0.871 | 0.996 | -0.006 |
| 3,4,5-Trimethoxybenzoic Acid | 0.710 | 0.854 | 0.119 | -3.067 |
| Hypoxanthine-9-β-D-Arabinofuranoside | 0.676 | 0.836 | 1.231 | 0.300 |
| D-Sedoheptuiose 7-Phosphate | 0.202 | 0.404 | 0.859 | -0.218 |
| D-Fructose 6-Phosphate-Disodium Salt | 0.002 | 0.015 | 1.274 | 0.350 |
| Aminomalonic Acid | 0.006 | 0.036 | 1.067 | 0.094 |
| 8,15-Dihete | 0.602 | 0.793 | 0.977 | -0.034 |
| N-Acetyl-L-methionine | 0.097 | 0.264 | 0.878 | -0.187 |
| Argininosuccinic acid | 0.466 | 0.707 | 1.080 | 0.110 |
| 2-Deoxyribose 1-Phosphate | 0.590 | 0.792 | 0.818 | -0.289 |
| N-Acetylglucosamine 1-Phosphate | 0.007 | 0.039 | 1.207 | 0.271 |
| Jasmonic acid | 0.019 | 0.080 | 1.511 | 0.596 |
| Indole-3-lactic acid | 0.892 | 0.944 | 0.971 | -0.043 |
| (3-Methoxy-4-hydroxyphenyl)ethylene glycol sulfate | 0.046 | 0.159 | 0.847 | -0.239 |
| Xanthosine | 0.090 | 0.254 | 1.128 | 0.174 |
| estrone 3-sulfate | 0.129 | 0.312 | 1.068 | 0.095 |
| 1-Methylguanine | 0.358 | 0.595 | 1.218 | 0.285 |
| DL-3,4-Dihydroxyphenyl glycol | 0.211 | 0.415 | 1.347 | 0.429 |
| dihydrotachysterol | 0.093 | 0.257 | 1.132 | 0.179 |
| Indoleacrylic acid | 0.491 | 0.726 | 1.066 | 0.092 |
| 2-(4-Hydroxyphenyl)ethanol | 0.626 | 0.806 | 1.022 | 0.032 |
| Hydroxyphenyllactic acid | 0.005 | 0.031 | 1.364 | 0.448 |
| Indole 3-carbinol | 0.741 | 0.863 | 0.997 | -0.004 |
| 2-Methylguanosine | 0.367 | 0.607 | 1.030 | 0.043 |
| 1,2,3-Trihydroxybenzene | 0.598 | 0.793 | 1.034 | 0.048 |
| N-lactoyl-phenylalanine | 0.000 | 0.002 | 1.443 | 0.529 |
| N-Acetyl-L-alanine | 0.396 | 0.641 | 1.030 | 0.042 |
| Cyclamic acid | 0.655 | 0.818 | 1.298 | 0.376 |
| D-Malic acid | 0.021 | 0.084 | 1.206 | 0.270 |
| Tetradecanedioic acid | 0.002 | 0.016 | 0.726 | -0.462 |
| Uridine triphosphate(UTP) | 0.163 | 0.362 | 1.312 | 0.392 |
| 6β-hydroxytestosterone | 0.121 | 0.299 | 0.968 | -0.047 |
| O-Acetyl-L-serine | 0.639 | 0.811 | 1.015 | 0.021 |
| Indoxylsulfuric acid | 0.602 | 0.793 | 1.113 | 0.155 |
| Porphobilinogen | 0.892 | 0.944 | 0.870 | -0.202 |
| Hydroquinone | 0.586 | 0.792 | 1.137 | 0.186 |
| Anthranilic acid | 0.523 | 0.746 | 1.081 | 0.112 |
| Indoleacetaldehyde | 0.000 | 0.005 | 1.077 | 0.107 |
| Hexadecanedioic acid | 0.001 | 0.009 | 0.701 | -0.512 |
| Pyrophosphate | 0.304 | 0.537 | 1.100 | 0.138 |
| 2-hydroxy-2-(4-hydroxy-3-methoxyphenyl)acetic acid | 0.168 | 0.367 | 1.183 | 0.243 |
| Glu-Leu | 0.000 | 0.001 | 1.271 | 0.346 |
| 5-oxoETE | 0.219 | 0.425 | 1.110 | 0.151 |
| Ethylsalicylate | 0.802 | 0.903 | 0.947 | -0.078 |
| Octadecanamide | 0.547 | 0.767 | 1.107 | 0.147 |
| Undecanedioic acid | 0.122 | 0.299 | 0.664 | -0.590 |
| Oxaloacetic acid | 0.299 | 0.530 | 0.959 | -0.060 |
| Phenoxyacetic acid | 0.802 | 0.903 | 0.905 | -0.144 |
| 4-Hydroxybenzyl alcohol | 0.015 | 0.070 | 1.134 | 0.182 |
| Methanesulfonic acid | 0.517 | 0.746 | 0.997 | -0.004 |
| Propylparaben | 0.906 | 0.949 | 0.977 | -0.034 |
| Butylparaben | 0.466 | 0.707 | 0.992 | -0.012 |
| Methylparaben | 0.181 | 0.383 | 1.156 | 0.209 |
| 44986 | 0.107 | 0.279 | 1.882 | 0.913 |
| (±)12-HEPE | 0.000 | 0.002 | 2.699 | 1.433 |
| (±)12-HETE | 0.058 | 0.186 | 1.488 | 0.573 |
| (±)15-HEPE | 0.000 | 0.002 | 2.699 | 1.433 |
| (±)17-HDHA | 0.001 | 0.013 | 1.995 | 0.997 |
| (±)18-HEPE | 0.000 | 0.002 | 2.699 | 1.433 |
| (±)4-HDHA | 0.003 | 0.021 | 1.242 | 0.313 |
| (±)5-HEPE | 0.000 | 0.004 | 1.974 | 0.981 |
| (±)5-HETE | 0.202 | 0.404 | 1.132 | 0.179 |
| (±)9-HETE | 0.202 | 0.404 | 1.132 | 0.179 |
| 11,12-EET | 0.018 | 0.075 | 1.515 | 0.599 |
| 13-oxoODE | 0.017 | 0.075 | 1.884 | 0.914 |
| 14(S)-HDHA | 0.001 | 0.009 | 1.848 | 0.886 |
| 15-oxoETE | 0.023 | 0.090 | 1.730 | 0.791 |
| 5(S),15(S)-DiHETE | 0.513 | 0.745 | 1.198 | 0.260 |
| 5,6-DiHETrE | 0.037 | 0.134 | 1.197 | 0.260 |
| 5-HETrE | 0.001 | 0.010 | 2.000 | 1.000 |
| 9-oxoODE | 0.017 | 0.075 | 1.884 | 0.914 |
| LTB4 | 0.459 | 0.702 | 1.286 | 0.363 |
| PDX | 0.107 | 0.279 | 1.882 | 0.913 |
| Prostaglandin E2 | 0.715 | 0.854 | 0.853 | -0.229 |
| RvD5 | 0.107 | 0.279 | 1.882 | 0.913 |
| Nα-Acetyl-L-glutamine | 0.528 | 0.750 | 1.011 | 0.016 |
| FFA(14:0) | 0.082 | 0.240 | 1.095 | 0.131 |
| 4-Hydroxyhippurate | 0.470 | 0.707 | 1.108 | 0.149 |
| 3-Hydroxyglutaric acid | 0.741 | 0.863 | 0.980 | -0.029 |
| 3-(3-Hydroxyphenyl)-3-hydroxypropanoic acid | 0.532 | 0.754 | 0.946 | -0.080 |
| N-acetylornithine | 0.017 | 0.075 | 1.113 | 0.155 |
| N-Alpha-Acetyl-L-Asparagine | 0.979 | 0.989 | 0.972 | -0.041 |
| N-Amidino-L-Aspartate | 0.059 | 0.186 | 1.139 | 0.188 |
| Lumichrome | 0.547 | 0.767 | 0.948 | -0.078 |
| 3-Amino-4-Hydroxybenzoic Acid | 0.062 | 0.194 | 1.205 | 0.269 |
| 2',4'-Dihydroxyacetophenone | 0.181 | 0.383 | 1.156 | 0.209 |
| Inosine | 0.655 | 0.818 | 1.179 | 0.238 |
| L-Sepiapterin | 0.002 | 0.016 | 1.228 | 0.296 |
| Phosphoenolpyruvate | 0.181 | 0.383 | 0.911 | -0.134 |
| Uric acid | 0.340 | 0.587 | 1.278 | 0.354 |
| 3-hydroxyphenylacetic acid | 0.402 | 0.646 | 1.093 | 0.128 |
| 7-Methylguanine | 0.358 | 0.595 | 1.218 | 0.285 |
| Nicotinic Acid | 1.000 | 1.000 | 0.999 | -0.001 |
| 3-(4-Hydroxyphenyl)-Propionic Acid | 0.852 | 0.922 | 0.990 | -0.015 |
| 5,6-Dimethylbenzimidazole | 0.022 | 0.090 | 1.043 | 0.060 |
| Gly-Phe | 0.536 | 0.758 | 1.030 | 0.043 |
| 13(R)-HODE | 0.047 | 0.160 | 1.734 | 0.794 |
| p-Cresol | 0.491 | 0.726 | 1.127 | 0.172 |
| Indole-4-carboxaldehyde | 0.164 | 0.362 | 0.866 | -0.208 |
| 9(S)-HpOTrE | 0.177 | 0.381 | 0.775 | -0.368 |
| Iminodiacetic acid | 0.045 | 0.158 | 1.139 | 0.188 |
| 2-Methyl-d-erythritol 2,4-cyclodiphosphate | 0.789 | 0.896 | 0.989 | -0.016 |
| Tauroursodeoxycholic acid | 0.643 | 0.811 | 1.007 | 0.010 |
| Gly-Val | 0.004 | 0.026 | 1.360 | 0.443 |
| FFA(18:4) | 0.000 | 0.003 | 1.815 | 0.860 |
| alpha-Muricholic acid | 0.728 | 0.854 | 1.069 | 0.096 |
| 12,13-DiHOME | 0.047 | 0.161 | 0.865 | -0.210 |
| Sphingosine 1-phosphate | 0.318 | 0.558 | 1.032 | 0.046 |
| 4-acetoxyphenol | 0.470 | 0.707 | 1.020 | 0.029 |
| 2-ethyl-2-hydroxybutyric acid | 0.567 | 0.778 | 0.930 | -0.105 |
| 7-ketolithocholic acid | 0.639 | 0.811 | 0.834 | -0.262 |
| 1,6-anhydro-β-D-glucose | 0.125 | 0.303 | 1.140 | 0.188 |
| 16-Hydroxyhexadecanoic acid | 0.352 | 0.592 | 1.068 | 0.095 |
| 12-ketolithocholic acid | 0.639 | 0.811 | 0.834 | -0.262 |
| N,N′-dicyclohexylcarbodiimide | 0.680 | 0.838 | 1.053 | 0.075 |
| Gamma-Mercholic Acid | 0.728 | 0.854 | 1.069 | 0.096 |
| Apocholic acid | 0.639 | 0.811 | 0.834 | -0.262 |
| 2-hydroxyhexadecanoic acid | 0.352 | 0.592 | 1.068 | 0.095 |
| Indole-3-carboxylic acid | 0.067 | 0.206 | 1.048 | 0.068 |
| 4-Methyl-2-oxovaleric acid | 0.212 | 0.416 | 1.147 | 0.197 |
| FFA(20:4) | 0.020 | 0.082 | 1.164 | 0.219 |
| Quinolinic acid | 0.825 | 0.913 | 1.001 | 0.001 |
| p-Tolyl Sulfate | 0.188 | 0.393 | 0.654 | -0.612 |
| 5-nitrobenzimidazole | 0.525 | 0.746 | 0.999 | -0.002 |
| Lythramine | 0.883 | 0.944 | 1.021 | 0.031 |
| Acetaminophen | 0.263 | 0.478 | 1.093 | 0.129 |
| 3-Sulfocatechol | 0.513 | 0.745 | 1.086 | 0.119 |
| 6-hydroxy-3-succinylpyridine | 0.470 | 0.707 | 1.108 | 0.149 |
| 2-(4-hydroxyphenyl) propionate | 0.163 | 0.362 | 0.551 | -0.861 |
| D-Mannose 6-phosphate | 0.002 | 0.015 | 1.274 | 0.350 |
| 2-amino-4-oxovaleric acid | 0.396 | 0.641 | 1.030 | 0.042 |
| 1-O-vanillyl-β-D-glucose | 0.299 | 0.530 | 1.057 | 0.080 |
| L-2-amino-6-oximelic acid | 0.270 | 0.489 | 1.167 | 0.223 |
| 3-(pyrazol-1-yl)-L-alanine | 0.003 | 0.020 | 1.099 | 0.137 |
| 1-pyrroline-4-hydroxy-2-carboxylate | 0.399 | 0.643 | 1.128 | 0.174 |
| LPE(0:0/22:4) | 0.320 | 0.560 | 0.959 | -0.060 |
| LPE(22:4/0:0) | 0.320 | 0.560 | 0.959 | -0.060 |
| LPE(0:0/22:5) | 0.149 | 0.342 | 0.878 | -0.187 |
| LPE(22:5/0:0) | 0.149 | 0.342 | 0.878 | -0.187 |
| LPE(0:0/22:6) | 0.003 | 0.022 | 1.249 | 0.320 |
| LPE(22:6/0:0) | 0.003 | 0.022 | 1.249 | 0.320 |
| LPE(0:0/20:2) | 0.194 | 0.398 | 0.874 | -0.194 |
| LPE(20:2/0:0) | 0.194 | 0.398 | 0.874 | -0.194 |
| LPE(0:0/20:3) | 0.100 | 0.267 | 1.113 | 0.154 |
| LPE(20:3/0:0) | 0.100 | 0.267 | 1.113 | 0.154 |
| LPE(20:4/0:0) | 0.754 | 0.871 | 0.979 | -0.031 |
| LPE(0:0/20:5) | 0.000 | 0.005 | 1.562 | 0.643 |
| LPE(20:5/0:0) | 0.000 | 0.005 | 1.562 | 0.643 |
| LPE(0:0/18:0) | 0.015 | 0.071 | 1.293 | 0.371 |
| LPE(0:0/18:2) | 0.270 | 0.489 | 0.895 | -0.161 |
| LPE(0:0/16:0) | 0.098 | 0.264 | 1.140 | 0.189 |
| LPE(0:0/16:1) | 0.793 | 0.898 | 1.030 | 0.042 |
| 1-Aminocyclohexanoic acid | 0.011 | 0.053 | 1.096 | 0.132 |
| Ureidosuccinic acid | 0.370 | 0.611 | 0.953 | -0.070 |
| 2-Hydroxycaprylic acid | 0.246 | 0.459 | 0.981 | -0.028 |
| 2-hydroxyphenylacetic acid | 0.023 | 0.091 | 1.257 | 0.330 |
| 4-Hydroxy-3-methylbenzoic acid | 0.023 | 0.091 | 1.257 | 0.330 |
| 2-Hydroxy-2-Methyl Butyric acid | 0.204 | 0.405 | 1.255 | 0.327 |
| 2-Octenoic acid | 0.881 | 0.944 | 0.975 | -0.036 |
| 2-Methylglutaric Acid | 0.053 | 0.177 | 0.910 | -0.136 |
| (S)-Leucic acid | 0.023 | 0.090 | 1.159 | 0.213 |
| Glycohyodeoxycholic acid | 0.582 | 0.789 | 0.794 | -0.332 |
| N-Cinnamylglycine | 0.016 | 0.071 | 0.483 | -1.050 |
| (R)-(-)-2-Phenylpropionic Acid | 0.041 | 0.144 | 0.681 | -0.554 |
| 8-Aminooctanoic Acid | 0.003 | 0.021 | 1.052 | 0.073 |
| 4-Methoxysalicylic Acid | 0.502 | 0.737 | 1.343 | 0.425 |
| Tridecanedioic acid | 0.138 | 0.327 | 1.026 | 0.036 |
| 12-Hydroxyoctadecanoic acid | 0.383 | 0.627 | 0.940 | -0.089 |
| N-Palmitoylglycine | 0.610 | 0.798 | 0.962 | -0.055 |
| Taurolithocholic acid | 0.184 | 0.387 | 1.099 | 0.136 |
| 13(S)-HOTrE(γ) | 0.000 | 0.001 | 2.440 | 1.287 |
| 9(S)-HOTrE | 0.000 | 0.002 | 2.329 | 1.220 |
| (±)8-HETE | 0.025 | 0.097 | 1.461 | 0.547 |
| 8(S)-HETrE | 0.001 | 0.010 | 2.000 | 1.000 |
| 15(S)-HETrE | 0.001 | 0.010 | 2.000 | 1.000 |
| 9(S),12(S),13(S)-TriHOME | 0.723 | 0.854 | 1.178 | 0.236 |
| Bicyclo Prostaglandin E2 | 0.149 | 0.342 | 1.282 | 0.358 |
| 20-COOH-AA | 0.293 | 0.523 | 1.219 | 0.286 |
| Prostaglandin B2 | 0.146 | 0.342 | 1.272 | 0.347 |
| 6-trans-12-epi Leukotriene B4 | 0.459 | 0.702 | 1.286 | 0.363 |
| 6-trans Leukotriene B4 | 0.459 | 0.702 | 1.286 | 0.363 |
| 13-HDoHE | 0.001 | 0.007 | 1.907 | 0.931 |
| 10-HDoHE | 0.001 | 0.006 | 2.140 | 1.098 |
| 8-HDoHE | 0.003 | 0.022 | 1.950 | 0.963 |
| 11-HDoHE | 0.001 | 0.013 | 2.060 | 1.043 |
| 16-HDoHE | 0.002 | 0.014 | 1.887 | 0.916 |
| 20-HDoHE | 0.002 | 0.015 | 2.114 | 1.080 |
| 11-HEDE | 0.088 | 0.251 | 1.549 | 0.632 |
| 15-HEDE | 0.088 | 0.251 | 1.549 | 0.632 |
| (±)19(20)-EpDPE(A) | 0.004 | 0.025 | 1.251 | 0.323 |
| 11β-Prostaglandin E2 | 0.715 | 0.854 | 0.853 | -0.229 |
| Glu-Gln | 0.011 | 0.053 | 0.706 | -0.503 |
| Ethionamide | 0.811 | 0.907 | 0.955 | -0.066 |
| Glycerophospho-N-Arachidonoyl Ethanolamine | 0.754 | 0.871 | 0.979 | -0.031 |
| Testosterone sulfate | 0.094 | 0.259 | 1.236 | 0.305 |
| FFA(16:2) | 0.472 | 0.707 | 1.081 | 0.112 |
| (R)-(-)-Mandelic acid | 0.405 | 0.646 | 1.296 | 0.374 |
| (R)-3-Hydroxybutanoic acid | 0.118 | 0.294 | 0.636 | -0.652 |
| 2-Hydroxyhexanoic acid | 0.023 | 0.090 | 1.159 | 0.213 |
| 2'-O-methyluridine | 0.190 | 0.394 | 0.947 | -0.079 |
| 2-Phenylbutyric acid | 0.202 | 0.404 | 1.089 | 0.123 |
| 3-Hydroxycinnamic acid | 0.221 | 0.427 | 1.010 | 0.014 |
| 3-Methyluridine | 0.241 | 0.456 | 0.927 | -0.110 |
| 3-Phenoxybenzoic acid | 0.961 | 0.981 | 0.967 | -0.049 |
| 4-Ethyloctanoic acid | 0.243 | 0.456 | 1.009 | 0.013 |
| 4-Methoxyphenol | 0.346 | 0.589 | 0.742 | -0.430 |
| 5-Hydroxy-2'-deoxyuridine | 0.405 | 0.646 | 1.095 | 0.132 |
| 6-Hydroxyflavone (6-HF) | 0.142 | 0.335 | 1.410 | 0.496 |
| Acetylvaline | 0.026 | 0.098 | 1.131 | 0.178 |
| D-Galacturonic Acid | 0.000 | 0.002 | 1.318 | 0.398 |
| Dimethylmalonic acid | 0.151 | 0.342 | 1.353 | 0.437 |
| D-Tagatose | 0.838 | 0.918 | 0.976 | -0.035 |
| Val-Ala | 0.651 | 0.818 | 0.971 | -0.043 |
| Isethionic acid | 0.767 | 0.880 | 0.997 | -0.005 |
| Octadecanedioic acid | 0.005 | 0.029 | 0.796 | -0.329 |
| Sucrose 6′-monophosphate | 0.602 | 0.793 | 1.004 | 0.005 |
| Traumatic acid | 0.622 | 0.805 | 1.005 | 0.008 |
| Phosphatidylethanolamine lyso alkenyl 16:0 | 0.005 | 0.028 | 0.880 | -0.185 |
| LPA(16:0/0:0) | 0.056 | 0.184 | 1.051 | 0.072 |
| 3-Hydroxy-tetradecanoic acid | 0.956 | 0.979 | 0.965 | -0.051 |
| Acetanilide | 0.255 | 0.470 | 0.977 | -0.033 |
| FFA(22:4) | 0.045 | 0.156 | 1.242 | 0.313 |
| Cytochalasin H | 0.058 | 0.186 | 0.922 | -0.117 |
| Glu-Val | 0.000 | 0.004 | 1.280 | 0.357 |
| Isocitric acid | 0.007 | 0.039 | 1.107 | 0.146 |
| N-Acetyl-5-aminosalicylic acid | 0.089 | 0.252 | 1.780 | 0.832 |
| 2,4-Quinolinediol | 0.525 | 0.746 | 0.990 | -0.014 |
| Glu-Thr | 0.001 | 0.008 | 1.441 | 0.527 |
| Glu-Tyr | 0.001 | 0.009 | 1.322 | 0.402 |
| LPA(18:1/0:0) | 0.000 | 0.005 | 0.827 | -0.274 |
| CMPentylF | 0.007 | 0.039 | 1.423 | 0.509 |
| Barbital | 0.676 | 0.836 | 0.999 | -0.001 |
| Androsterone sulfate | 0.852 | 0.922 | 1.026 | 0.038 |
| Hydroxypiperazic acid | 0.002 | 0.015 | 0.819 | -0.289 |
| 2-Naphthalenesulfonic acid | 0.883 | 0.944 | 1.006 | 0.009 |
| 2-Deoxyribose 5'-phosphate | 0.915 | 0.954 | 0.940 | -0.089 |
| 3-(2-Naphthyl)-L-alanine | 0.473 | 0.707 | 1.009 | 0.013 |
| L-threo-3-Methylaspartate | 0.000 | 0.000 | 1.421 | 0.507 |
| FAHFA(8:0/10:0) | 0.000 | 0.002 | 2.323 | 1.216 |
| Leu-Ile | 0.998 | 1.000 | 0.933 | -0.100 |
| Docodiendioicacid | 0.131 | 0.315 | 1.150 | 0.202 |
| His-Ser | 0.847 | 0.922 | 1.128 | 0.174 |
| Phosphatidylethanolamine lyso alkenyl 18:2 | 0.146 | 0.342 | 1.160 | 0.214 |
| 2-Methylhexanoic acid | 0.521 | 0.746 | 0.988 | -0.017 |
| 3-(2-Hydroxyphenyl)propanoic acid | 0.852 | 0.922 | 0.990 | -0.015 |
| Salicyluric acid | 0.103 | 0.273 | 3.481 | 1.800 |
| 2-Octanamidoacetic acid | 0.052 | 0.174 | 0.720 | -0.474 |
| Palatinose | 0.610 | 0.798 | 0.947 | -0.079 |
| Tropine | 0.182 | 0.386 | 1.155 | 0.208 |
| M-toluene acetic acid | 0.204 | 0.405 | 0.843 | -0.246 |
| Piperic acid | 0.820 | 0.911 | 0.999 | -0.002 |
| Cholic acid | 0.728 | 0.854 | 1.069 | 0.096 |
| 7-Nitroindazole | 0.080 | 0.237 | 0.894 | -0.162 |
| 4-Methylhexanoic acid | 0.521 | 0.746 | 0.988 | -0.017 |
| 3-Methoxycatechol | 0.415 | 0.660 | 1.426 | 0.512 |
| 3,4-Dimethylbenzoic acid | 0.861 | 0.929 | 0.906 | -0.143 |
| 2,2-Dimethylpentanoic acid | 0.521 | 0.746 | 0.988 | -0.017 |
| D-Talose | 0.838 | 0.918 | 0.976 | -0.035 |
| D-Allose | 0.838 | 0.918 | 0.976 | -0.035 |
| 3,4-Dimethoxycinnamic acid | 0.058 | 0.186 | 0.701 | -0.513 |
| Naphthofluorescein | 0.164 | 0.362 | 1.167 | 0.223 |
| Zereno | 0.563 | 0.777 | 1.037 | 0.052 |
| FFA(16:1) | 0.993 | 0.998 | 1.100 | 0.138 |
| Dihydrodaidzein | 0.525 | 0.746 | 1.325 | 0.406 |
| 20-Hydroxy Prostaglandin F2α | 0.780 | 0.890 | 1.001 | 0.001 |
| Pinolenic acid | 0.000 | 0.001 | 1.544 | 0.627 |
| 15(R)-17-phenyl trinor prostaglandin F2α | 0.445 | 0.692 | 0.941 | -0.087 |
| O-1821 | 0.525 | 0.746 | 1.111 | 0.152 |
| 9,10-dihydroxystearic acid | 0.892 | 0.944 | 0.988 | -0.017 |
| (R)-3-Hydroxymyristic acid | 0.956 | 0.979 | 0.965 | -0.051 |
| 6,6'-Dihydroxy-5,5'-dimethoxybiphenyl-3,3'-dicarboxylic acid | 0.793 | 0.898 | 0.996 | -0.006 |
| 4-Oxoretinoic acid | 0.425 | 0.673 | 1.057 | 0.080 |
| N-Myristoylglycine | 0.723 | 0.854 | 0.993 | -0.010 |
| Isochodeoxycholic acid | 0.002 | 0.016 | 1.847 | 0.885 |
| Carbocyclic thromboxane A2 | 0.104 | 0.275 | 1.345 | 0.428 |
| Hydroferulic acid | 0.449 | 0.693 | 0.771 | -0.375 |
| (S)-2-Hydroxy-3-phenylpropanoic acid | 0.221 | 0.427 | 0.541 | -0.886 |
| Ethyl hydrogen malonate | 0.151 | 0.342 | 1.353 | 0.437 |
| L-Gulose | 0.586 | 0.792 | 1.068 | 0.094 |
| Deoxycholic acid | 0.338 | 0.583 | 1.522 | 0.605 |
| beta-Muricholic acid | 0.728 | 0.854 | 1.069 | 0.096 |
| Glycine deoxycholic acid | 0.736 | 0.863 | 0.725 | -0.464 |
| 3-Epideoxycholic acid | 0.006 | 0.032 | 1.592 | 0.671 |
| 5-Carboxyvanillic Acid | 0.598 | 0.793 | 0.974 | -0.038 |
| 4-Hydroxybenzoic Acid | 0.374 | 0.615 | 1.015 | 0.021 |
| Cys-Pro | 0.506 | 0.739 | 0.994 | -0.009 |
| Ser-Ala | 0.685 | 0.842 | 1.050 | 0.070 |
| Ala-Glu | 0.392 | 0.641 | 0.998 | -0.002 |
| S-Methyl-L-Cysteine-S-oxide | 0.706 | 0.853 | 0.951 | -0.073 |
| L-lyxose | 0.491 | 0.726 | 1.015 | 0.022 |
| Val-Asn | 0.258 | 0.470 | 0.868 | -0.204 |
| D-ribonate lithium salt | 0.095 | 0.262 | 1.141 | 0.191 |
| γ-Glu-Gln | 0.011 | 0.053 | 0.706 | -0.503 |
| Val-Thr | 0.915 | 0.954 | 0.931 | -0.103 |
| 2-keto-D-gluconic acid | 0.000 | 0.002 | 1.318 | 0.398 |
| Met-Asp | 0.741 | 0.863 | 1.006 | 0.009 |
| Val-Gly | 0.058 | 0.186 | 1.316 | 0.397 |
| Pyroglutamic acid | 0.920 | 0.955 | 0.956 | -0.065 |
| cyclo(gly-glu) | 0.001 | 0.007 | 1.572 | 0.653 |
| α-Hydroxyglutaric Acid (sodium salt) | 0.000 | 0.000 | 1.334 | 0.416 |
| cyclo(glu-glu) | 0.000 | 0.002 | 1.207 | 0.271 |
| Ile-Gly | 0.618 | 0.804 | 1.080 | 0.111 |
| Ile-Val | 0.728 | 0.854 | 1.002 | 0.003 |
| Asp-Leu | 0.058 | 0.186 | 1.216 | 0.282 |
| N-Acetyl-L-Glutamic Acid | 0.622 | 0.805 | 0.997 | -0.004 |
| Trp-Gly | 0.000 | 0.002 | 1.227 | 0.295 |
| 2-Hydroxy-3-Methyl Butanoic Acid | 0.210 | 0.414 | 1.263 | 0.336 |
| Salicylic acid β-D-O-glucuronic acid | 0.251 | 0.464 | 1.104 | 0.143 |
| Homovanillic Acid sulfate (sodium salt) | 0.251 | 0.464 | 1.254 | 0.327 |
| 4-Acetylaminobenzoic acid | 0.114 | 0.288 | 0.653 | -0.614 |
| Trp-Leu | 0.019 | 0.077 | 1.185 | 0.245 |
| 4-toluenesulfonic acid | 0.151 | 0.342 | 0.822 | -0.283 |
| 2,4-Dihydroxy-6-pentylbenzoic acid | 0.031 | 0.118 | 1.160 | 0.214 |
| Daidzein | 0.811 | 0.907 | 1.011 | 0.016 |
| D-Gulonic acid γ-lactone | 0.706 | 0.853 | 0.931 | -0.103 |
| L-Iditol | 0.984 | 0.992 | 1.037 | 0.052 |
| 3-Amino-5-hydroxybenzoic acid | 0.062 | 0.194 | 1.205 | 0.269 |
| 2-Methyllactic acid | 0.016 | 0.071 | 1.357 | 0.440 |
| Imidazole-4-methanol | 0.825 | 0.913 | 1.010 | 0.014 |
| (R)-2-Hydroxybutyric acid | 0.016 | 0.071 | 1.357 | 0.440 |
| 2-Methyl-3-hydroxybutyric acid | 0.055 | 0.180 | 1.091 | 0.125 |
| Pyrazine-2-carboxylic acid | 0.135 | 0.322 | 0.960 | -0.059 |
| (2s)-2-Amino-4-sulfinobutanoic acid | 0.547 | 0.767 | 0.978 | -0.032 |
| Ala-Ser | 0.961 | 0.981 | 0.975 | -0.037 |
| （2S，3R，4R，5R）-2,3,4,5,6-五羟基己醛 | 0.838 | 0.918 | 0.976 | -0.035 |
| L-rhamnonic acid | 0.947 | 0.976 | 1.018 | 0.026 |
| 3-Hydroxy-L-phenylalanine | 0.047 | 0.161 | 1.073 | 0.102 |
| Lys-Gly | 0.825 | 0.913 | 1.020 | 0.029 |
| 2-(Acetylamino)-2-deoxy-A-D-glucopyranose | 0.970 | 0.983 | 1.092 | 0.127 |
| Hyp-Thr | 0.833 | 0.918 | 1.009 | 0.013 |
| γ-Glu-Met | 0.470 | 0.707 | 0.875 | -0.193 |
| Lys-Phe | 0.005 | 0.029 | 1.095 | 0.131 |
| Met-Phe | 0.000 | 0.004 | 1.725 | 0.786 |
| Dipyrocetyl | 0.343 | 0.587 | 1.197 | 0.259 |
| 4-(Hydroxyamino)quinoline 1-oxide | 0.355 | 0.595 | 1.052 | 0.073 |
| P-Toluenesulfonamide | 0.004 | 0.024 | 2.349 | 1.232 |
| 4-Hydroxyquinoline | 0.005 | 0.029 | 1.080 | 0.112 |
| 2-Phenyl-5-benzimidazole sulfonic acid | 0.723 | 0.854 | 0.977 | -0.033 |
| 10-Hydroxystearic Acid | 0.122 | 0.299 | 0.951 | -0.072 |
| L-Glycine | 0.011 | 0.055 | 0.861 | -0.217 |
| L-Cystine | 0.361 | 0.599 | 1.021 | 0.030 |
| L-Tyrosine | 0.000 | 0.002 | 1.211 | 0.276 |
| L-Ornithine | 0.745 | 0.867 | 1.055 | 0.078 |
| L-Alanine | 0.000 | 0.005 | 1.324 | 0.405 |
| L-Histidine | 0.014 | 0.066 | 1.084 | 0.117 |
| L-Methionine | 0.459 | 0.702 | 1.014 | 0.019 |
| L-Proline | 0.013 | 0.061 | 1.235 | 0.304 |
| L-Valine | 0.000 | 0.000 | 1.257 | 0.330 |
| 5-Oxoproline | 0.590 | 0.792 | 0.948 | -0.076 |
| Betaine | 0.217 | 0.422 | 0.874 | -0.194 |
| Glyc-Pro | 0.865 | 0.933 | 1.061 | 0.086 |
| L-Cysteine | 0.008 | 0.044 | 0.851 | -0.232 |
| N6-Acetyl-L-Lysine | 0.000 | 0.002 | 1.202 | 0.265 |
| N-Acetylcysteine | 0.225 | 0.430 | 0.930 | -0.105 |
| N-Acetylputrescine | 0.343 | 0.587 | 0.924 | -0.114 |
| Serotonin | 0.811 | 0.907 | 0.911 | -0.134 |
| Trimethylamine-N-Oxide | 0.015 | 0.070 | 1.543 | 0.626 |
| Ala-Lys | 0.767 | 0.880 | 1.118 | 0.161 |
| N-Acetylhistamine | 0.253 | 0.467 | 0.810 | -0.304 |
| P-Coumaric Acid | 0.000 | 0.000 | 1.296 | 0.374 |
| 1,4-Dihydro-1-Methyl-4-Oxo-3-Pyridinecarboxamide | 0.547 | 0.767 | 1.100 | 0.137 |
| Theobromine | 0.405 | 0.646 | 0.507 | -0.979 |
| Choline | 0.594 | 0.793 | 0.965 | -0.051 |
| 1,5-Diaminopentane | 0.924 | 0.957 | 0.978 | -0.032 |
| Diethanolamine | 0.702 | 0.853 | 1.005 | 0.008 |
| Myoinositol | 0.280 | 0.504 | 0.970 | -0.044 |
| 1-Methylhistidine | 0.901 | 0.948 | 1.072 | 0.101 |
| 5,6-Dihydro-5-Methyluracil | 0.993 | 0.998 | 0.981 | -0.027 |
| 5-Methyluridine | 0.190 | 0.394 | 0.947 | -0.079 |
| Adenine | 0.003 | 0.020 | 1.210 | 0.274 |
| Cytosine | 0.473 | 0.707 | 0.956 | -0.065 |
| Purine | 0.000 | 0.001 | 1.143 | 0.193 |
| Uracil | 0.449 | 0.693 | 1.009 | 0.013 |
| 3,3',5-Triiodo-L-Thyronine | 0.614 | 0.800 | 1.017 | 0.024 |
| N-Methyltryptamine | 0.332 | 0.576 | 0.911 | -0.134 |
| D-Fructose | 0.883 | 0.944 | 0.985 | -0.021 |
| D-Mannose | 0.883 | 0.944 | 0.985 | -0.021 |
| D-Gluconic Acid | 0.631 | 0.809 | 0.982 | -0.026 |
| Orotic Acid | 0.428 | 0.677 | 1.073 | 0.101 |
| Nicotinamide | 0.137 | 0.325 | 1.236 | 0.306 |
| Riboflavin | 0.293 | 0.523 | 1.133 | 0.181 |
| Trigonelline | 0.655 | 0.818 | 1.202 | 0.265 |
| 3-Indolebutyric Acid | 0.196 | 0.398 | 1.036 | 0.051 |
| 2-Aminoethanesulfonic Acid | 0.938 | 0.969 | 0.982 | -0.027 |
| 4-Guanidinobutyric Acid | 0.838 | 0.918 | 1.032 | 0.046 |
| 5-Aminovaleric Acid | 0.555 | 0.776 | 0.921 | -0.119 |
| 6-Aminocaproic-Acid | 0.001 | 0.006 | 1.168 | 0.224 |
| 7-Methyluric Acid | 0.470 | 0.707 | 1.047 | 0.066 |
| Creatinine | 0.396 | 0.641 | 1.048 | 0.068 |
| Dl-2-Aminooctanoic Acid | 0.018 | 0.077 | 0.707 | -0.501 |
| Guanidineacetic Acid | 0.689 | 0.844 | 1.063 | 0.089 |
| L-Dihydroorotic Acid | 0.293 | 0.523 | 0.970 | -0.044 |
| L-Homoserine | 0.622 | 0.805 | 1.027 | 0.038 |
| Maleic Acid | 0.010 | 0.051 | 1.136 | 0.184 |
| LPC(0:0/14:0) | 0.000 | 0.000 | 1.598 | 0.677 |
| LPC(16:0/0:0) | 0.008 | 0.041 | 1.079 | 0.109 |
| Trans-3-Hydroxycotinine | 0.001 | 0.010 | 0.020 | -5.668 |
| L-Homoarginine | 0.000 | 0.001 | 1.337 | 0.419 |
| Pantetheine | 0.697 | 0.853 | 0.965 | -0.052 |
| D-piperidine acid | 0.590 | 0.792 | 0.948 | -0.076 |
| 5'-Deoxy-5'-(Methylthio) Adenosine | 0.074 | 0.222 | 1.131 | 0.178 |
| Sarcosine | 0.602 | 0.793 | 0.992 | -0.011 |
| Imidazoleacetic acid | 0.123 | 0.300 | 1.063 | 0.088 |
| 2-Aminoadipic Acid | 0.001 | 0.010 | 1.344 | 0.426 |
| LPC(17:0/0:0) | 0.820 | 0.911 | 0.995 | -0.007 |
| Sn-Glycero-3-Phosphocholine | 0.147 | 0.342 | 1.243 | 0.313 |
| Indole | 0.432 | 0.678 | 1.060 | 0.085 |
| LPC(15:0/0:0) | 0.000 | 0.002 | 1.231 | 0.299 |
| LPC(0:0/18:2) | 0.243 | 0.456 | 0.961 | -0.058 |
| 2-Hydroxycinnamic acid | 0.106 | 0.278 | 1.148 | 0.199 |
| Carnitine C2:0 | 0.966 | 0.981 | 1.086 | 0.119 |
| DL-Stachydrine | 0.816 | 0.910 | 0.928 | -0.108 |
| L-Norleucine | 0.000 | 0.006 | 1.112 | 0.154 |
| DL-Carnitine | 0.049 | 0.166 | 1.174 | 0.231 |
| 6-Dimethylaminopurine | 0.147 | 0.342 | 1.060 | 0.085 |
| Triethyl-phosphate | 0.470 | 0.707 | 1.039 | 0.055 |
| LPC(O-16:0/2:0) | 0.196 | 0.398 | 1.016 | 0.022 |
| 18-Hydroxycorticosterone | 0.614 | 0.800 | 1.030 | 0.042 |
| Oleamide | 0.723 | 0.854 | 0.979 | -0.031 |
| Carnitine isoC4:0 | 0.114 | 0.288 | 1.209 | 0.274 |
| 2'-Hydroxy-5'-methylacetophenone | 0.473 | 0.707 | 1.025 | 0.035 |
| Spermidine | 0.273 | 0.493 | 1.100 | 0.138 |
| N-Acetyl-L-Histidine | 0.728 | 0.854 | 1.018 | 0.025 |
| 2-Furoylglycine | 0.092 | 0.256 | 0.561 | -0.834 |
| Carnitine-2-methyl-C4 | 0.002 | 0.015 | 1.171 | 0.227 |
| Isonicotinic acid | 0.123 | 0.300 | 0.889 | -0.169 |
| Phe-Pro | 0.001 | 0.009 | 1.236 | 0.306 |
| LPE(16:1/0:0) | 0.680 | 0.838 | 0.924 | -0.113 |
| 3-Chloroaniline | 0.906 | 0.949 | 1.004 | 0.005 |
| Methylcysteine | 0.343 | 0.587 | 1.049 | 0.068 |
| DL-Leucine | 0.001 | 0.006 | 1.168 | 0.224 |
| 6-Methylnicotinamide | 0.702 | 0.853 | 0.990 | -0.015 |
| (R)-2-Hydroxy-3-phenylpropionic-acid | 0.943 | 0.973 | 0.963 | -0.054 |
| 1-Aminopropan-2-ol | 0.015 | 0.070 | 1.543 | 0.626 |
| N-Methylalanine | 0.897 | 0.944 | 1.029 | 0.041 |
| Dihydro-D-sphingosine | 0.892 | 0.944 | 1.000 | 0.000 |
| Hypaphorine | 0.513 | 0.745 | 0.995 | -0.008 |
| N-Methyl-L-Glutamate | 0.001 | 0.010 | 1.344 | 0.426 |
| Phosphocholine | 0.676 | 0.836 | 1.003 | 0.004 |
| 2,4-Dihydroxypteridine | 0.046 | 0.159 | 1.175 | 0.232 |
| Cortisol | 0.017 | 0.075 | 0.838 | -0.255 |
| L-Tryptophanamide | 0.017 | 0.075 | 1.105 | 0.144 |
| Catechol | 0.559 | 0.777 | 0.988 | -0.018 |
| Acetylcholine | 0.459 | 0.702 | 0.947 | -0.079 |
| 1-Hydroxylamino-2-phenylethane | 0.000 | 0.002 | 1.120 | 0.164 |
| β-Alanine | 0.000 | 0.005 | 1.324 | 0.405 |
| D-(+)-sucrose | 0.495 | 0.728 | 1.033 | 0.046 |
| Thr-Phe | 0.166 | 0.364 | 1.490 | 0.575 |
| Salicylaldehyde | 0.159 | 0.357 | 0.710 | -0.493 |
| Benzaldehyde | 0.000 | 0.000 | 1.173 | 0.230 |
| Biliverdin | 0.442 | 0.689 | 1.127 | 0.172 |
| N,-N-diacetyl-O-methylhydroxylamine | 0.643 | 0.811 | 0.982 | -0.027 |
| Hydroxyquinoline | 0.000 | 0.005 | 1.140 | 0.188 |
| DL-O-tyrosine | 0.000 | 0.000 | 1.085 | 0.117 |
| 7-Methylguanosine | 0.225 | 0.430 | 1.090 | 0.124 |
| 6-O-methylguanine | 0.521 | 0.746 | 1.032 | 0.046 |
| Oxypurinol | 0.000 | 0.005 | 1.511 | 0.596 |
| Carnitine C12:0 | 0.243 | 0.456 | 0.954 | -0.068 |
| Allopurinol | 0.604 | 0.795 | 0.986 | -0.021 |
| PC(12:0/12:0) | 0.033 | 0.121 | 1.135 | 0.183 |
| (R)-(-)-2-phenylglycine | 0.000 | 0.002 | 1.120 | 0.164 |
| 1-acetylindole | 0.016 | 0.073 | 1.080 | 0.111 |
| 3-Carboxypropyltrimethylammonium | 0.432 | 0.678 | 0.967 | -0.048 |
| L-Isoleucine | 0.000 | 0.001 | 1.148 | 0.199 |
| LPC(O-18:0/0:0) | 0.109 | 0.280 | 0.848 | -0.238 |
| Creatine phosphate | 0.989 | 0.996 | 1.018 | 0.026 |
| PC(O-16:0/O-2:0) | 0.109 | 0.280 | 0.848 | -0.238 |
| Butenoyl-PAF | 0.003 | 0.022 | 0.840 | -0.251 |
| N-Methyl-α-aminoisobutyric acid | 0.079 | 0.236 | 0.863 | -0.213 |
| Biotinamide | 0.248 | 0.463 | 1.096 | 0.132 |
| 8,8a-deoxy-oleane | 0.754 | 0.871 | 1.002 | 0.003 |
| N-acetylpyrrolidine | 0.059 | 0.186 | 1.060 | 0.084 |
| 1,3-Dicyclohexylurea | 0.842 | 0.920 | 0.975 | -0.036 |
| PC(O-16:0/O-1:0) | 0.019 | 0.078 | 1.042 | 0.059 |
| 4-tert-butylbenzoic-acid | 0.278 | 0.501 | 1.137 | 0.185 |
| 4-Hydroxytryptamine | 0.003 | 0.022 | 0.009 | -6.818 |
| 1-Deoxyvaleric-acid | 0.789 | 0.896 | 1.032 | 0.046 |
| 2-Mercaptobenzothiazole | 0.010 | 0.051 | 0.735 | -0.443 |
| Urobilin | 0.049 | 0.166 | 2.178 | 1.123 |
| 20,26-dihydroxyecdysone | 0.009 | 0.047 | 1.062 | 0.087 |
| Carnitine C6:0 | 0.706 | 0.853 | 0.967 | -0.049 |
| Leu-Gly | 0.329 | 0.573 | 1.111 | 0.151 |
| LPC(0:0/22:4) | 0.210 | 0.414 | 0.881 | -0.183 |
| LPC(22:4/0:0) | 0.210 | 0.414 | 0.881 | -0.183 |
| LPC(0:0/22:5) | 0.771 | 0.881 | 0.945 | -0.082 |
| LPC(22:5/0:0) | 0.008 | 0.042 | 1.200 | 0.263 |
| LPC(20:1/0:0) | 0.003 | 0.022 | 0.840 | -0.251 |
| LPC(20:2/0:0) | 0.563 | 0.777 | 0.972 | -0.040 |
| LPC(0:0/20:2) | 0.563 | 0.777 | 0.972 | -0.040 |
| LPC(0:0/20:3) | 0.004 | 0.026 | 1.250 | 0.322 |
| LPC(20:3/0:0) | 0.004 | 0.026 | 1.250 | 0.322 |
| LPC(0:0/20:4) | 0.098 | 0.264 | 1.065 | 0.091 |
| LPC(20:4/0:0) | 0.098 | 0.264 | 1.065 | 0.091 |
| LPC(18:0/0:0) | 0.196 | 0.398 | 1.016 | 0.022 |
| LPC(18:1/0:0) | 0.000 | 0.005 | 0.777 | -0.364 |
| LPC(18:2/0:0) | 0.243 | 0.456 | 0.961 | -0.058 |
| LPC(0:0/16:0) | 0.008 | 0.041 | 1.079 | 0.109 |
| LPC(16:1/0:0) | 0.559 | 0.777 | 1.032 | 0.046 |
| Mycosporine-glycine | 0.200 | 0.404 | 0.871 | -0.199 |
| Carnitine C18:0 | 0.838 | 0.918 | 1.037 | 0.052 |
| Carnitine C18:2 | 0.022 | 0.088 | 1.223 | 0.291 |
| Carnitine C16:0 | 0.053 | 0.175 | 1.125 | 0.171 |
| Carnitine C16:1 | 0.069 | 0.210 | 0.837 | -0.256 |
| Carnitine C16:2 | 0.807 | 0.906 | 1.021 | 0.030 |
| Carnitine C14-OH | 0.784 | 0.893 | 0.988 | -0.017 |
| Carnitine C14:2-OH | 0.952 | 0.977 | 0.878 | -0.188 |
| Carnitine C14:2 | 0.771 | 0.881 | 1.026 | 0.037 |
| Carnitine C12-OH | 0.106 | 0.278 | 0.951 | -0.072 |
| Carnitine C11:DC | 0.251 | 0.464 | 1.009 | 0.013 |
| Carnitine C13:1 | 0.432 | 0.678 | 1.025 | 0.036 |
| Carnitine C11:0 | 0.338 | 0.583 | 1.215 | 0.281 |
| Carnitine C11:1 | 0.019 | 0.077 | 1.437 | 0.523 |
| Carnitine C10:0 | 0.315 | 0.554 | 0.911 | -0.135 |
| Carnitine C8-OH | 0.349 | 0.592 | 0.832 | -0.266 |
| Carnitine C9:0 | 0.058 | 0.186 | 1.382 | 0.467 |
| Carnitine C8:0 | 0.578 | 0.785 | 0.910 | -0.137 |
| Carnitine C8:1 | 0.000 | 0.005 | 2.658 | 1.410 |
| Carnitine ph-C1 | 0.296 | 0.527 | 0.580 | -0.785 |
| Carnitine C4:DC | 0.952 | 0.977 | 0.991 | -0.013 |
| Carnitine C5:0 | 0.002 | 0.015 | 1.171 | 0.227 |
| Carnitine C5:1 | 0.057 | 0.185 | 1.159 | 0.212 |
| Carnitine C4:0 | 0.114 | 0.288 | 1.209 | 0.274 |
| 1,3-Diphenylguanidine | 0.631 | 0.809 | 1.001 | 0.001 |
| N'-Methyl-2-pyridone-5-carboxamide | 0.000 | 0.001 | 1.597 | 0.675 |
| 5-Methoxytryptamine | 0.068 | 0.208 | 1.053 | 0.075 |
| L-Phenylephrine | 0.915 | 0.954 | 0.948 | -0.077 |
| Theophylline | 0.304 | 0.537 | 1.452 | 0.538 |
| SDMA | 0.435 | 0.681 | 1.073 | 0.101 |
| Methylguanidine | 0.204 | 0.405 | 1.119 | 0.163 |
| N6-methyladenosine | 0.011 | 0.053 | 1.197 | 0.259 |
| 8-Azaguanine | 0.000 | 0.005 | 1.511 | 0.596 |
| Isocytosine | 0.473 | 0.707 | 0.956 | -0.065 |
| Leu-Val | 0.594 | 0.793 | 1.191 | 0.252 |
| Phe-Asn | 0.045 | 0.156 | 1.216 | 0.283 |
| Phe-Met | 0.000 | 0.002 | 1.477 | 0.563 |
| Glu-Met | 0.689 | 0.844 | 0.891 | -0.167 |
| Met-Glu | 0.767 | 0.880 | 0.916 | -0.127 |
| Phe-Val | 0.002 | 0.016 | 1.241 | 0.311 |
| Carnitine C10:1 | 0.132 | 0.317 | 1.201 | 0.264 |
| Carnitine C14:2:DC | 0.067 | 0.206 | 0.874 | -0.195 |
| Caldine | 0.979 | 0.989 | 0.958 | -0.062 |
| 1-Methyladenosine | 0.011 | 0.053 | 1.197 | 0.259 |
| PC(O-16:0/0:0) | 1.000 | 1.000 | 0.993 | -0.009 |
| LPC(O-18:1/0:0) | 0.010 | 0.050 | 0.871 | -0.199 |
| LPC(O-0:0/18:0) | 0.109 | 0.280 | 0.848 | -0.238 |
| NE,NE,NE-TRIMETHYLLYSINE | 0.000 | 0.001 | 1.270 | 0.345 |
| N,N-Dimethylarginine | 0.018 | 0.075 | 1.125 | 0.170 |
| Carnitine C3:0 | 0.004 | 0.026 | 1.297 | 0.376 |
| Phe-Glu | 0.075 | 0.223 | 1.427 | 0.513 |
| Gly-Gly-Phe | 0.002 | 0.014 | 1.136 | 0.183 |
| LPC(0:0/17:0) | 0.517 | 0.746 | 1.013 | 0.019 |
| LPC(0:0/15:0) | 0.000 | 0.002 | 1.231 | 0.299 |
| LPC(12:0/0:0) | 0.110 | 0.282 | 1.339 | 0.421 |
| LPC(O-16:1/0:0) | 0.723 | 0.854 | 0.962 | -0.056 |
| Sphingosyl-phosphocholine | 0.087 | 0.251 | 1.163 | 0.218 |
| LPC(0:0/16:1) | 0.559 | 0.777 | 1.032 | 0.046 |
| LPC(0:0/18:0) | 0.196 | 0.398 | 1.016 | 0.022 |
| LPC(0:0/18:1) | 0.000 | 0.005 | 0.777 | -0.364 |
| LPC(0:0/20:1) | 0.003 | 0.022 | 0.840 | -0.251 |
| Methyldopa | 0.293 | 0.523 | 0.996 | -0.006 |
| (E)-Guggulsterone | 0.018 | 0.077 | 1.137 | 0.185 |
| (Z)-Guggulsterone | 0.018 | 0.077 | 1.137 | 0.185 |
| 1-Methylinosine | 0.938 | 0.969 | 0.925 | -0.113 |
| 2'-O-methylcytidine | 0.396 | 0.641 | 1.100 | 0.138 |
| Acrylamide | 0.000 | 0.001 | 1.237 | 0.307 |
| Agmatine | 0.149 | 0.342 | 0.956 | -0.065 |
| Ala-Phe | 0.003 | 0.020 | 1.130 | 0.176 |
| Cyromazine | 0.602 | 0.793 | 0.996 | -0.006 |
| Cytidine 5'-diphosphate | 0.418 | 0.664 | 0.928 | -0.107 |
| L-Allothreonine | 0.622 | 0.805 | 1.027 | 0.038 |
| D-Glucosaminic acid | 0.606 | 0.795 | 0.993 | -0.010 |
| Guanidine | 0.338 | 0.583 | 0.894 | -0.161 |
| Leu-Ala | 0.888 | 0.944 | 1.029 | 0.041 |
| L-Norvaline | 0.000 | 0.000 | 1.257 | 0.330 |
| Leu-Phe | 0.161 | 0.360 | 1.321 | 0.402 |
| N4-Acetylcytidine | 0.352 | 0.592 | 1.080 | 0.111 |
| Gly-Ile | 0.123 | 0.300 | 1.336 | 0.417 |
| Palmitoylethanolamide（(PEA） | 0.037 | 0.135 | 1.097 | 0.134 |
| Sanguinarine | 0.358 | 0.595 | 1.164 | 0.219 |
| Stachydrine | 0.798 | 0.902 | 1.091 | 0.125 |
| Synephrine | 0.477 | 0.711 | 1.000 | 0.000 |
| Bicine | 0.917 | 0.955 | 0.954 | -0.068 |
| Eicosanoyl-EA | 0.897 | 0.944 | 0.997 | -0.005 |
| Gln-Phe | 0.111 | 0.285 | 1.039 | 0.055 |
| Ile-Met | 0.000 | 0.001 | 1.382 | 0.466 |
| LPE(17:1/0:0) | 0.032 | 0.120 | 0.798 | -0.326 |
| Pro-Ile | 0.003 | 0.022 | 1.571 | 0.652 |
| Ser-Leu | 0.847 | 0.922 | 0.909 | -0.137 |
| Ser-Phe | 0.064 | 0.198 | 1.106 | 0.145 |
| Tyr-Leu | 0.525 | 0.746 | 1.169 | 0.225 |
| 3-Hydroxyphenylurea | 0.966 | 0.981 | 0.982 | -0.026 |
| Caffeine | 0.223 | 0.428 | 3.931 | 1.975 |
| Carnitine C9:1-OH | 0.013 | 0.060 | 3.301 | 1.723 |
| LPE(18:2/0:0) | 0.223 | 0.428 | 0.952 | -0.070 |
| (E,Z)-2-Amino-3,14-octadecadien-1-ol | 0.723 | 0.854 | 0.979 | -0.031 |
| Phe-Trp | 0.784 | 0.893 | 1.049 | 0.069 |
| N(Alpha)-Acetyl-Epsilon-(2-Propenal)Lysine | 0.001 | 0.006 | 1.963 | 0.973 |
| Cyclo(Pro-Leu) | 0.003 | 0.019 | 1.488 | 0.573 |
| Thr-Gln | 0.975 | 0.986 | 1.250 | 0.322 |
| Cyclo(Phe-Glu) | 0.037 | 0.135 | 1.291 | 0.368 |
| Cyclo(Pro-Val) | 0.005 | 0.029 | 1.223 | 0.291 |
| Ile-Asp | 0.002 | 0.017 | 1.737 | 0.797 |
| N-MethyTrans-4-Hydroxy-Proline | 0.217 | 0.422 | 0.763 | -0.390 |
| Ser-Ile | 0.847 | 0.922 | 0.909 | -0.137 |
| 5-Aminoimidazole ribonucleotide | 0.043 | 0.151 | 1.267 | 0.342 |
| N6-(2-Hydroxyethyl)adenosine | 0.484 | 0.719 | 1.056 | 0.079 |
| 7-(alpha-D-glucosyl)-N(6)-isopentenyladenine | 0.995 | 0.999 | 1.006 | 0.009 |
| D-Proline-betaine | 0.626 | 0.806 | 1.080 | 0.111 |
| 2-Methyl-1-Pyrroline | 0.027 | 0.104 | 1.144 | 0.195 |
| Melibiose | 0.032 | 0.119 | 1.193 | 0.254 |
| N-Formylglycine | 0.563 | 0.777 | 1.045 | 0.063 |
| D-Ornithine | 0.719 | 0.854 | 1.070 | 0.098 |
| Dehydroascorbic-acid | 0.055 | 0.180 | 0.910 | -0.136 |
| Androstenediol | 0.170 | 0.370 | 0.980 | -0.030 |
| Triethylenetetramine | 0.121 | 0.299 | 0.933 | -0.100 |
| FFA(10:1) | 0.237 | 0.448 | 1.108 | 0.148 |
| 3-Aminoquinoline | 0.001 | 0.006 | 1.146 | 0.196 |
| L-Isserine | 0.643 | 0.811 | 0.995 | -0.007 |
| 1-Amino-1-cyclobutane-carboxylic-acid | 0.013 | 0.061 | 1.235 | 0.304 |
| N,N-Bis(2-hydroxyethyl)dodecanamide | 0.639 | 0.811 | 0.999 | -0.001 |
| (E)-8-Methyl-6-nonenoic-acid | 0.090 | 0.254 | 1.199 | 0.262 |
| Cork-oximate | 0.706 | 0.853 | 1.021 | 0.030 |
| 8-iso-15-keto-Prostaglandin-F2α | 0.134 | 0.319 | 1.318 | 0.398 |
| 2-Amino-3-phosphonopropionic-acid | 0.897 | 0.944 | 0.955 | -0.066 |
| Inositol 1,3,4-trisphosphate | 0.352 | 0.592 | 1.008 | 0.012 |
| S-methyl-L-thiocitrulline | 0.171 | 0.372 | 0.851 | -0.232 |
| ST-638 | 0.223 | 0.428 | 1.125 | 0.170 |
| Leu-Leu | 0.966 | 0.981 | 0.891 | -0.166 |
| Gly-Lys | 0.741 | 0.863 | 1.021 | 0.030 |
| Gly-Gln | 0.415 | 0.660 | 1.023 | 0.033 |
| Lys-Ser | 0.495 | 0.728 | 1.040 | 0.057 |
| Arg-Glu | 0.078 | 0.234 | 1.383 | 0.467 |
| Glu-His | 0.002 | 0.016 | 1.323 | 0.404 |
| Cyclocreatine | 0.758 | 0.875 | 1.035 | 0.050 |
| γ-Glu-Lys | 0.009 | 0.048 | 0.710 | -0.493 |
| (R)-(-)-1-Amino-2-propanol | 0.009 | 0.048 | 1.357 | 0.440 |
| Gly-Thr | 0.190 | 0.394 | 1.050 | 0.070 |
| Pro-Ser | 0.590 | 0.792 | 0.985 | -0.022 |
| N-Ethylglycine | 0.897 | 0.944 | 1.029 | 0.041 |
| 3-Guanidinopropionic acid | 0.255 | 0.470 | 0.963 | -0.055 |
| Glu-Ser | 0.660 | 0.821 | 0.881 | -0.183 |
| Ammeline | 0.484 | 0.719 | 0.925 | -0.112 |
| Thr-Glu | 0.088 | 0.251 | 1.239 | 0.309 |
| Glu-Cit | 0.121 | 0.299 | 0.815 | -0.295 |
| Glu-Gly | 0.005 | 0.029 | 0.811 | -0.302 |
| N-acetyl-D-Lactosamine | 0.004 | 0.026 | 1.479 | 0.565 |
| (+/-)-High-Proline | 0.159 | 0.357 | 1.135 | 0.183 |
| 3-(imidazol-4-yl)propionic-acid | 0.915 | 0.954 | 0.998 | -0.003 |
| Ser-Pro | 0.091 | 0.256 | 1.145 | 0.195 |
| N1-Acetylspermidine | 0.175 | 0.378 | 0.923 | -0.115 |
| Ser-Val | 0.513 | 0.745 | 1.059 | 0.082 |
| Ile-Gln | 0.008 | 0.044 | 1.276 | 0.351 |
| Ile-Ser | 0.002 | 0.015 | 1.274 | 0.350 |
| Tyr-Glu | 0.802 | 0.903 | 0.935 | -0.096 |
| Ile-Glu | 0.115 | 0.289 | 1.411 | 0.497 |
| Leu-Glu | 0.115 | 0.289 | 1.411 | 0.497 |
| Glu-Ile | 0.115 | 0.289 | 1.411 | 0.497 |
| Ile-Thr | 0.258 | 0.470 | 1.287 | 0.364 |
| Leu-Thr | 0.258 | 0.470 | 1.287 | 0.364 |
| 2,2'-Cyclouridine | 0.188 | 0.393 | 1.105 | 0.144 |
| 3-aminobenzamide | 0.920 | 0.955 | 0.748 | -0.420 |
| N-(2-hydroxyethyl)-3-pyridinecarboxamide | 0.000 | 0.001 | 1.143 | 0.193 |
| Phe-Ala-Ser | 0.004 | 0.026 | 1.603 | 0.680 |
| Ser-Phe-Ala | 0.004 | 0.026 | 1.603 | 0.680 |
| 1-Methylguanosine | 0.225 | 0.430 | 1.090 | 0.124 |
| Cyclo(Ala-Pro) | 0.067 | 0.206 | 1.093 | 0.129 |
| Phe-Gly | 0.567 | 0.778 | 1.020 | 0.028 |
| Val-Ile | 0.092 | 0.256 | 1.243 | 0.314 |
| Val-Leu | 0.092 | 0.256 | 1.243 | 0.314 |
| Asp-Ile | 0.002 | 0.017 | 1.737 | 0.797 |
| Leu-Met | 0.000 | 0.001 | 1.382 | 0.466 |
| cyclo(pro-pro) | 0.089 | 0.252 | 1.172 | 0.229 |
| Phe-Tyr | 0.005 | 0.029 | 1.933 | 0.951 |
| Ile-Leu | 0.966 | 0.981 | 0.891 | -0.166 |
| Phe-Ala-Leu | 0.888 | 0.944 | 1.044 | 0.063 |
| Securinine | 0.228 | 0.433 | 0.919 | -0.122 |
| Thr-Arg | 0.134 | 0.319 | 0.908 | -0.138 |
| Arg-Thr | 0.082 | 0.240 | 0.853 | -0.229 |
| Leu-Asn | 0.002 | 0.015 | 1.152 | 0.204 |
| Glu-Phe-Ala | 0.056 | 0.184 | 1.219 | 0.286 |
| 4-Amino-3-hydroxybutyric acid | 0.445 | 0.692 | 0.984 | -0.023 |
| D-Allo-Isoleucine | 0.000 | 0.001 | 1.148 | 0.199 |
| 5-Acetylamino-6-amino-3-methyluracil | 0.349 | 0.592 | 0.966 | -0.050 |
| Gln-Gly | 0.807 | 0.906 | 1.012 | 0.017 |
| Gly-Glu | 0.005 | 0.029 | 0.811 | -0.302 |
| Lys-Ala | 0.767 | 0.880 | 1.118 | 0.161 |
| Pro-Asn | 0.405 | 0.646 | 1.004 | 0.006 |
| Pro-Asp | 0.001 | 0.007 | 1.926 | 0.946 |
| Arg-Gly | 0.358 | 0.595 | 1.082 | 0.113 |
| Gly-Arg | 0.358 | 0.595 | 1.082 | 0.113 |
| Ser-Lys | 0.563 | 0.777 | 1.054 | 0.076 |
| Ser-Glu | 0.626 | 0.806 | 0.938 | -0.093 |
| Phe-Ala | 0.014 | 0.066 | 1.123 | 0.168 |
| Ile-Asn | 0.002 | 0.015 | 1.152 | 0.204 |
| Leu-Asp | 0.039 | 0.140 | 1.270 | 0.344 |
| Phe-Thr | 0.166 | 0.364 | 1.490 | 0.575 |
| Phe-Hyp | 0.635 | 0.811 | 0.980 | -0.030 |
| Glu-Phe | 0.075 | 0.223 | 1.427 | 0.513 |
| Glu-Arg | 0.164 | 0.362 | 1.253 | 0.326 |
| 2-Butyl-3-(4-hydroxybenzoyl)benzofuran | 0.000 | 0.000 | 0.667 | -0.585 |
| Quinmerac | 0.152 | 0.345 | 0.956 | -0.065 |
| Chaps | 0.771 | 0.881 | 0.972 | -0.041 |
| trans-resveratrol-3-O-sulfate | 0.051 | 0.170 | 0.790 | -0.340 |

**Table S10.** 839 Metabolites comparisions between group 42 trajectory and group 1 trajectory in females.

| Compounds | P_value | P_adjusted | FC | log2FC |
| --- | --- | --- | --- | --- |
| 3-carboxy-4-methyl-5-propyl-2-furanpropionic acid | 0.042 | 0.210 | 1.309 | 0.388 |
| 2,4-diacetamino-2,4,6-triphenoxy-D-mannopyranose | 0.014 | 0.117 | 1.259 | 0.332 |
| L-Threonine | 0.993 | 0.999 | 1.075 | 0.105 |
| L-Arginine | 0.183 | 0.468 | 1.043 | 0.061 |
| L-Aspartic Acid | 0.042 | 0.207 | 1.066 | 0.092 |
| L-Citrulline | 0.110 | 0.365 | 0.949 | -0.076 |
| L-Glutamic Acid | 0.001 | 0.030 | 1.182 | 0.241 |
| L-Phenylalanine | 0.000 | 0.016 | 1.061 | 0.086 |
| L-Serine | 0.743 | 0.912 | 0.989 | -0.016 |
| L-Tryptophan | 0.006 | 0.077 | 1.063 | 0.088 |
| (5-L-Glutamyl)-L-Amino Acid | 0.475 | 0.723 | 0.918 | -0.123 |
| Allantoin | 0.122 | 0.389 | 1.503 | 0.588 |
| Asp-Phe | 0.531 | 0.771 | 1.024 | 0.035 |
| Glutathione Oxidized | 0.176 | 0.458 | 1.039 | 0.056 |
| Hexanoyl Glycine | 0.381 | 0.654 | 0.930 | -0.105 |
| L-Asparagine Anhydrous | 0.622 | 0.838 | 1.007 | 0.010 |
| L-Glutamine | 0.012 | 0.117 | 0.909 | -0.138 |
| L-Homocitrulline | 0.066 | 0.277 | 0.869 | -0.203 |
| L-Theanine | 0.777 | 0.925 | 0.992 | -0.012 |
| N-Acetylaspartate | 0.793 | 0.930 | 0.975 | -0.037 |
| N-Acetyl-L-Leucine | 0.074 | 0.291 | 1.146 | 0.197 |
| N-Acetyl-L-Tyrosine | 0.528 | 0.771 | 0.984 | -0.024 |
| N-Acetylneuraminic Acid(SA) | 0.965 | 0.996 | 0.958 | -0.062 |
| Gly-Leu | 0.603 | 0.836 | 0.969 | -0.045 |
| N-Isovaleroylglycine | 0.158 | 0.441 | 0.844 | -0.245 |
| N-Propionylglycine | 0.002 | 0.046 | 1.087 | 0.121 |
| Nα-Acetyl-L-Arginine | 0.063 | 0.270 | 0.901 | -0.151 |
| O-Phospho-L-Serine | 0.910 | 0.985 | 1.008 | 0.012 |
| Phenylacetyl-L-Glutamine | 0.011 | 0.116 | 0.698 | -0.519 |
| Phe-Phe | 0.652 | 0.858 | 0.929 | -0.106 |
| S-(5-Adenosy)-L-Homocysteine | 0.025 | 0.156 | 1.123 | 0.167 |
| S-Sulfo-L-Cysteine | 0.616 | 0.838 | 0.964 | -0.053 |
| Trans-4-Hydroxy-L-Proline | 0.002 | 0.046 | 1.087 | 0.121 |
| γ-L-Glutamate-Cysteine | 0.228 | 0.513 | 1.013 | 0.018 |
| N-Acetyl-L-phenylalanine | 0.187 | 0.470 | 1.100 | 0.137 |
| Benzoylformic Acid | 0.850 | 0.961 | 0.996 | -0.006 |
| 3-Hydroxyanthranilic Acid | 0.094 | 0.339 | 1.050 | 0.070 |
| P–Hydroxyphenyl Acetic Acid | 0.743 | 0.912 | 1.005 | 0.007 |
| 2-Picolinic Acid | 0.815 | 0.938 | 0.997 | -0.005 |
| 4-Pyridoxic Acid | 0.157 | 0.440 | 1.094 | 0.129 |
| 6-Hydroxynicotinic Acid | 0.469 | 0.719 | 1.118 | 0.161 |
| Taurocholic acid | 0.595 | 0.831 | 0.916 | -0.127 |
| Taurochenodesoxycholic Acid | 0.369 | 0.650 | 1.065 | 0.091 |
| Glycolithocholic acid | 0.617 | 0.838 | 1.113 | 0.154 |
| Hyodeoxycholic acid | 0.773 | 0.925 | 1.184 | 0.244 |
| Glycoursodeoxycholic Acid | 0.254 | 0.538 | 0.959 | -0.060 |
| Glycochenodeoxycholic Acid | 0.194 | 0.480 | 0.975 | -0.036 |
| Chenodeoxycholic Acid | 0.698 | 0.883 | 0.859 | -0.220 |
| 4-Methylcatechol | 0.107 | 0.365 | 0.810 | -0.304 |
| 4-Hydroxy-3-methoxybenzaldehyde | 0.028 | 0.168 | 0.923 | -0.116 |
| 1,7-Dimethylxanthine | 0.413 | 0.678 | 0.622 | -0.684 |
| 1-Methylxanthine | 0.421 | 0.684 | 0.949 | -0.076 |
| Xanthine | 0.024 | 0.156 | 1.114 | 0.156 |
| 3-Methylxanthine | 0.421 | 0.684 | 0.949 | -0.076 |
| 5-Methylcytosine | 0.260 | 0.540 | 1.097 | 0.134 |
| 7-Methylxanthine | 0.421 | 0.684 | 0.949 | -0.076 |
| Guanosine | 0.304 | 0.597 | 1.019 | 0.027 |
| Hypoxanthine | 0.163 | 0.446 | 1.082 | 0.114 |
| Uridine | 0.075 | 0.293 | 1.048 | 0.068 |
| L-Thyroxine | 0.494 | 0.737 | 0.977 | -0.033 |
| Norepinephrine | 0.893 | 0.975 | 1.015 | 0.021 |
| Succinic Acid | 0.674 | 0.866 | 1.031 | 0.044 |
| Cis-Aconitic Acid | 0.760 | 0.917 | 0.985 | -0.022 |
| Melatonin | 0.737 | 0.910 | 0.985 | -0.022 |
| Tryptamine | 0.036 | 0.193 | 1.025 | 0.036 |
| D-Glucose | 0.221 | 0.504 | 0.950 | -0.073 |
| D-Trehalose | 0.204 | 0.491 | 1.047 | 0.067 |
| D-Glucose 6-Phosphate | 0.343 | 0.627 | 0.989 | -0.016 |
| Lactose | 0.204 | 0.491 | 1.047 | 0.067 |
| Lactulose | 0.204 | 0.491 | 1.047 | 0.067 |
| L-Fucose | 0.477 | 0.723 | 1.062 | 0.087 |
| Maltose | 0.204 | 0.491 | 1.047 | 0.067 |
| D-Glucoronic Acid | 0.052 | 0.237 | 1.052 | 0.074 |
| Pantothenate | 0.000 | 0.008 | 1.196 | 0.259 |
| 3-Indolepropionic Acid | 0.730 | 0.906 | 1.048 | 0.068 |
| Indole-3-Carboxaldehyde | 0.696 | 0.883 | 0.977 | -0.033 |
| Methyl Indole-3-Acetate | 0.007 | 0.081 | 1.194 | 0.256 |
| 2-Hydroxybutanoic Acid | 0.890 | 0.975 | 1.028 | 0.039 |
| 2-Hydroxyisocaproic Acid | 0.322 | 0.605 | 0.945 | -0.081 |
| 2-Methylsuccinic Acid | 0.777 | 0.925 | 0.962 | -0.056 |
| 3-Hydroxy-3-Methyl Butyric Acid | 0.150 | 0.436 | 0.906 | -0.143 |
| 3-Methylcrotonyl Glycine | 0.035 | 0.193 | 1.193 | 0.255 |
| 4-Hydroxy-2-Oxoglutaric Acid | 0.001 | 0.033 | 1.138 | 0.187 |
| Adipic Acid | 0.346 | 0.627 | 1.018 | 0.026 |
| Azelaic Acid | 0.391 | 0.664 | 1.011 | 0.016 |
| Caffeic Acid | 0.494 | 0.737 | 0.923 | -0.115 |
| Creatine | 0.623 | 0.838 | 0.898 | -0.155 |
| Dodecanedioic Aicd | 0.189 | 0.472 | 0.983 | -0.025 |
| Glutaric Acid | 0.777 | 0.925 | 0.962 | -0.056 |
| Guanidinoethyl Sulfonate | 0.475 | 0.723 | 1.077 | 0.107 |
| Hippuric Acid | 0.395 | 0.665 | 0.941 | -0.088 |
| Hydrocinnamic Acid | 0.033 | 0.191 | 0.813 | -0.299 |
| L-kynurenine | 0.513 | 0.758 | 1.039 | 0.055 |
| Kynurenic Acid | 0.177 | 0.459 | 1.046 | 0.065 |
| L-Lactic Acid | 0.666 | 0.864 | 1.067 | 0.093 |
| Malonicacid | 0.912 | 0.986 | 1.030 | 0.043 |
| Mandelic Acid | 0.743 | 0.912 | 1.005 | 0.007 |
| Methylmalonic Acid | 0.674 | 0.866 | 1.031 | 0.044 |
| Phenyllactate(Pla) | 0.916 | 0.987 | 0.968 | -0.046 |
| Pyrrole-2-Carboxylic Acid | 0.229 | 0.513 | 1.090 | 0.124 |
| Sebacate | 0.233 | 0.518 | 1.017 | 0.024 |
| Shikimic Acid | 0.033 | 0.190 | 0.780 | -0.359 |
| Subericacid | 0.298 | 0.593 | 1.028 | 0.040 |
| TXB2 | 0.700 | 0.884 | 0.700 | -0.515 |
| (±)15-HETE | 0.096 | 0.344 | 1.122 | 0.166 |
| LPG(18:1/0:0) | 0.241 | 0.528 | 1.029 | 0.041 |
| LPE(18:1/0:0) | 0.213 | 0.503 | 0.903 | -0.148 |
| LPE(18:0/0:0) | 0.022 | 0.149 | 1.128 | 0.173 |
| LPE(16:0/0:0) | 0.020 | 0.149 | 1.114 | 0.156 |
| LPE(14:0/0:0) | 0.102 | 0.358 | 1.108 | 0.147 |
| LPA(0:0/18:0) | 0.206 | 0.493 | 1.028 | 0.040 |
| LPA(0:0/16:0) | 0.402 | 0.667 | 0.996 | -0.006 |
| LipoxinA4 | 0.319 | 0.605 | 1.060 | 0.084 |
| 13-HOTrE | 0.000 | 0.022 | 1.584 | 0.664 |
| 9,10-DiHOME | 0.062 | 0.269 | 0.818 | -0.289 |
| FFA(18:3) | 0.001 | 0.029 | 1.205 | 0.270 |
| FFA(16:0) | 0.710 | 0.892 | 1.013 | 0.018 |
| FFA(18:2) | 0.234 | 0.518 | 1.037 | 0.052 |
| FFA(12:0) | 0.163 | 0.446 | 0.950 | -0.074 |
| FFA(18:1) | 0.886 | 0.975 | 0.979 | -0.030 |
| EPA | 0.001 | 0.028 | 1.588 | 0.667 |
| FFA(20:2) | 0.559 | 0.799 | 1.072 | 0.101 |
| FFA(10:0) | 0.959 | 0.993 | 0.901 | -0.150 |
| AA | 0.184 | 0.468 | 1.042 | 0.060 |
| Urocanic Acid | 0.051 | 0.235 | 1.044 | 0.063 |
| 4-Hydroxybenzaldehyde | 0.303 | 0.597 | 0.983 | -0.024 |
| Neopterin | 0.442 | 0.703 | 1.015 | 0.022 |
| Ethylmalonate | 0.759 | 0.917 | 1.007 | 0.009 |
| 2-(Formylamino)Benzoic Acid | 0.048 | 0.227 | 0.883 | -0.180 |
| Ureidoisobutyric Acid | 0.040 | 0.206 | 1.408 | 0.494 |
| Uridine 5-Monophosphate | 0.897 | 0.977 | 1.000 | 0.000 |
| N-Acetylglycine | 0.217 | 0.504 | 0.921 | -0.118 |
| 3-Hydroxyhippuric Acid | 0.438 | 0.701 | 0.954 | -0.068 |
| 2-(Dimethylamino)Guanosine | 0.474 | 0.723 | 1.021 | 0.030 |
| 5-Hydroxyhexanoic Acid | 0.322 | 0.605 | 0.945 | -0.081 |
| Β-Pseudouridine | 0.332 | 0.615 | 0.963 | -0.055 |
| N-Acetylthreonine | 0.998 | 1.000 | 0.975 | -0.037 |
| 3,4,5-Trimethoxybenzoic Acid | 0.516 | 0.760 | 1.019 | 0.026 |
| Hypoxanthine-9-β-D-Arabinofuranoside | 0.314 | 0.605 | 1.078 | 0.108 |
| D-Sedoheptuiose 7-Phosphate | 0.250 | 0.535 | 1.004 | 0.005 |
| D-Fructose 6-Phosphate-Disodium Salt | 0.343 | 0.627 | 0.989 | -0.016 |
| Aminomalonic Acid | 0.593 | 0.831 | 0.999 | -0.002 |
| 8,15-Dihete | 0.459 | 0.713 | 1.046 | 0.065 |
| N-Acetyl-L-methionine | 0.919 | 0.987 | 1.021 | 0.030 |
| Argininosuccinic acid | 0.156 | 0.440 | 1.082 | 0.114 |
| 2-Deoxyribose 1-Phosphate | 0.637 | 0.850 | 1.021 | 0.030 |
| N-Acetylglucosamine 1-Phosphate | 0.149 | 0.436 | 1.040 | 0.057 |
| Jasmonic acid | 0.011 | 0.116 | 27.975 | 4.806 |
| Indole-3-lactic acid | 0.611 | 0.838 | 0.968 | -0.047 |
| (3-Methoxy-4-hydroxyphenyl)ethylene glycol sulfate | 0.154 | 0.436 | 0.931 | -0.104 |
| Xanthosine | 0.114 | 0.371 | 1.075 | 0.104 |
| estrone 3-sulfate | 0.693 | 0.883 | 1.014 | 0.020 |
| 1-Methylguanine | 0.478 | 0.723 | 0.984 | -0.024 |
| DL-3,4-Dihydroxyphenyl glycol | 0.443 | 0.703 | 0.991 | -0.013 |
| dihydrotachysterol | 0.403 | 0.667 | 1.088 | 0.122 |
| Indoleacrylic acid | 0.719 | 0.901 | 1.133 | 0.181 |
| 2-(4-Hydroxyphenyl)ethanol | 0.994 | 0.999 | 1.020 | 0.029 |
| Hydroxyphenyllactic acid | 0.142 | 0.432 | 1.053 | 0.075 |
| Indole 3-carbinol | 0.522 | 0.767 | 0.996 | -0.005 |
| 2-Methylguanosine | 0.929 | 0.990 | 1.028 | 0.040 |
| 1,2,3-Trihydroxybenzene | 0.456 | 0.712 | 1.110 | 0.151 |
| N-lactoyl-phenylalanine | 0.095 | 0.340 | 1.111 | 0.152 |
| N-Acetyl-L-alanine | 0.530 | 0.771 | 1.023 | 0.033 |
| Cyclamic acid | 0.815 | 0.938 | 1.116 | 0.158 |
| D-Malic acid | 0.057 | 0.255 | 1.067 | 0.094 |
| Tetradecanedioic acid | 0.102 | 0.358 | 0.949 | -0.075 |
| Uridine triphosphate(UTP) | 0.858 | 0.963 | 0.926 | -0.111 |
| 6β-hydroxytestosterone | 0.661 | 0.861 | 1.003 | 0.005 |
| O-Acetyl-L-serine | 0.137 | 0.425 | 0.964 | -0.052 |
| Indoxylsulfuric acid | 0.056 | 0.249 | 0.800 | -0.322 |
| Porphobilinogen | 0.373 | 0.650 | 1.089 | 0.123 |
| Hydroquinone | 0.983 | 0.997 | 1.029 | 0.041 |
| Anthranilic acid | 0.444 | 0.703 | 0.944 | -0.083 |
| Indoleacetaldehyde | 0.010 | 0.107 | 1.040 | 0.057 |
| Hexadecanedioic acid | 0.006 | 0.077 | 0.874 | -0.194 |
| Pyrophosphate | 0.287 | 0.578 | 1.137 | 0.185 |
| 2-hydroxy-2-(4-hydroxy-3-methoxyphenyl)acetic acid | 0.066 | 0.277 | 1.085 | 0.118 |
| Glu-Leu | 0.007 | 0.077 | 1.106 | 0.146 |
| 5-oxoETE | 0.014 | 0.120 | 1.355 | 0.438 |
| Ethylsalicylate | 0.373 | 0.650 | 1.061 | 0.085 |
| Octadecanamide | 0.330 | 0.613 | 1.055 | 0.077 |
| Undecanedioic acid | 0.111 | 0.365 | 0.820 | -0.287 |
| Oxaloacetic acid | 0.303 | 0.597 | 1.065 | 0.091 |
| Phenoxyacetic acid | 0.153 | 0.436 | 1.376 | 0.461 |
| 4-Hydroxybenzyl alcohol | 0.459 | 0.713 | 1.041 | 0.058 |
| Methanesulfonic acid | 0.949 | 0.993 | 0.986 | -0.020 |
| Propylparaben | 0.549 | 0.791 | 1.008 | 0.012 |
| Butylparaben | 0.076 | 0.297 | 0.989 | -0.016 |
| Methylparaben | 0.893 | 0.975 | 0.969 | -0.046 |
| 44986 | 0.048 | 0.225 | 1.219 | 0.285 |
| (±)12-HEPE | 0.000 | 0.008 | 1.828 | 0.870 |
| (±)12-HETE | 0.041 | 0.207 | 1.144 | 0.195 |
| (±)15-HEPE | 0.000 | 0.008 | 1.828 | 0.870 |
| (±)17-HDHA | 0.008 | 0.092 | 1.238 | 0.308 |
| (±)18-HEPE | 0.000 | 0.008 | 1.828 | 0.870 |
| (±)4-HDHA | 0.014 | 0.117 | 1.108 | 0.148 |
| (±)5-HEPE | 0.000 | 0.016 | 1.538 | 0.621 |
| (±)5-HETE | 0.139 | 0.428 | 1.102 | 0.141 |
| (±)9-HETE | 0.139 | 0.428 | 1.102 | 0.141 |
| 11,12-EET | 0.067 | 0.278 | 1.179 | 0.237 |
| 13-oxoODE | 0.060 | 0.261 | 1.228 | 0.296 |
| 14(S)-HDHA | 0.003 | 0.049 | 1.210 | 0.275 |
| 15-oxoETE | 0.014 | 0.117 | 1.303 | 0.382 |
| 5(S),15(S)-DiHETE | 0.214 | 0.503 | 1.068 | 0.094 |
| 5,6-DiHETrE | 0.161 | 0.446 | 1.001 | 0.001 |
| 5-HETrE | 0.022 | 0.149 | 1.198 | 0.261 |
| 9-oxoODE | 0.060 | 0.261 | 1.228 | 0.296 |
| LTB4 | 0.167 | 0.446 | 1.150 | 0.201 |
| PDX | 0.048 | 0.225 | 1.219 | 0.285 |
| Prostaglandin E2 | 0.259 | 0.539 | 1.140 | 0.189 |
| RvD5 | 0.048 | 0.225 | 1.219 | 0.285 |
| Nα-Acetyl-L-glutamine | 0.814 | 0.938 | 0.991 | -0.013 |
| FFA(14:0) | 0.621 | 0.838 | 1.001 | 0.001 |
| 4-Hydroxyhippurate | 0.728 | 0.905 | 1.058 | 0.081 |
| 3-Hydroxyglutaric acid | 0.869 | 0.971 | 0.971 | -0.043 |
| 3-(3-Hydroxyphenyl)-3-hydroxypropanoic acid | 0.263 | 0.544 | 0.855 | -0.226 |
| N-acetylornithine | 0.229 | 0.513 | 1.081 | 0.112 |
| N-Alpha-Acetyl-L-Asparagine | 0.247 | 0.535 | 0.977 | -0.034 |
| N-Amidino-L-Aspartate | 0.070 | 0.284 | 1.080 | 0.111 |
| Lumichrome | 0.065 | 0.274 | 1.088 | 0.121 |
| 3-Amino-4-Hydroxybenzoic Acid | 0.094 | 0.339 | 1.050 | 0.070 |
| 2',4'-Dihydroxyacetophenone | 0.893 | 0.975 | 0.969 | -0.046 |
| Inosine | 0.335 | 0.620 | 1.041 | 0.058 |
| L-Sepiapterin | 0.033 | 0.190 | 1.065 | 0.091 |
| Phosphoenolpyruvate | 1.000 | 1.000 | 1.012 | 0.017 |
| Uric acid | 0.223 | 0.504 | 1.120 | 0.163 |
| 3-hydroxyphenylacetic acid | 0.279 | 0.566 | 0.915 | -0.129 |
| 7-Methylguanine | 0.478 | 0.723 | 0.984 | -0.024 |
| Nicotinic Acid | 0.600 | 0.833 | 1.016 | 0.022 |
| 3-(4-Hydroxyphenyl)-Propionic Acid | 0.379 | 0.653 | 0.996 | -0.006 |
| 5,6-Dimethylbenzimidazole | 0.025 | 0.156 | 1.020 | 0.029 |
| Gly-Phe | 0.667 | 0.864 | 1.023 | 0.032 |
| 13(R)-HODE | 0.207 | 0.493 | 0.937 | -0.094 |
| p-Cresol | 0.446 | 0.704 | 0.877 | -0.190 |
| Indole-4-carboxaldehyde | 0.696 | 0.883 | 0.977 | -0.033 |
| 9(S)-HpOTrE | 0.441 | 0.703 | 1.027 | 0.039 |
| Iminodiacetic acid | 0.022 | 0.149 | 1.096 | 0.132 |
| 2-Methyl-d-erythritol 2,4-cyclodiphosphate | 0.793 | 0.930 | 1.017 | 0.025 |
| Tauroursodeoxycholic acid | 0.013 | 0.117 | 1.090 | 0.125 |
| Gly-Val | 0.373 | 0.650 | 1.148 | 0.200 |
| FFA(18:4) | 0.043 | 0.210 | 1.241 | 0.312 |
| alpha-Muricholic acid | 0.980 | 0.997 | 0.981 | -0.028 |
| 12,13-DiHOME | 0.078 | 0.300 | 0.871 | -0.199 |
| Sphingosine 1-phosphate | 0.829 | 0.946 | 1.011 | 0.016 |
| 4-acetoxyphenol | 0.164 | 0.446 | 0.994 | -0.009 |
| 2-ethyl-2-hydroxybutyric acid | 0.873 | 0.971 | 1.016 | 0.023 |
| 7-ketolithocholic acid | 0.394 | 0.664 | 0.727 | -0.459 |
| 1,6-anhydro-β-D-glucose | 0.018 | 0.142 | 0.867 | -0.207 |
| 16-Hydroxyhexadecanoic acid | 0.970 | 0.996 | 0.999 | -0.001 |
| 12-ketolithocholic acid | 0.394 | 0.664 | 0.727 | -0.459 |
| N,N′-dicyclohexylcarbodiimide | 0.482 | 0.726 | 0.974 | -0.038 |
| Gamma-Mercholic Acid | 0.980 | 0.997 | 0.981 | -0.028 |
| Apocholic acid | 0.394 | 0.664 | 0.727 | -0.459 |
| 2-hydroxyhexadecanoic acid | 0.970 | 0.996 | 0.999 | -0.001 |
| Indole-3-carboxylic acid | 0.819 | 0.941 | 1.002 | 0.002 |
| 4-Methyl-2-oxovaleric acid | 0.256 | 0.538 | 0.906 | -0.142 |
| FFA(20:4) | 0.190 | 0.474 | 1.042 | 0.060 |
| Quinolinic acid | 0.672 | 0.866 | 1.012 | 0.017 |
| p-Tolyl Sulfate | 0.167 | 0.446 | 0.812 | -0.300 |
| 5-nitrobenzimidazole | 0.314 | 0.605 | 0.992 | -0.011 |
| Lythramine | 0.222 | 0.504 | 1.056 | 0.078 |
| Acetaminophen | 0.036 | 0.193 | 1.358 | 0.441 |
| 3-Sulfocatechol | 1.000 | 1.000 | 1.055 | 0.077 |
| 6-hydroxy-3-succinylpyridine | 0.728 | 0.905 | 1.058 | 0.081 |
| 2-(4-hydroxyphenyl) propionate | 0.002 | 0.046 | 0.569 | -0.815 |
| D-Mannose 6-phosphate | 0.343 | 0.627 | 0.989 | -0.016 |
| 2-amino-4-oxovaleric acid | 0.530 | 0.771 | 1.023 | 0.033 |
| 1-O-vanillyl-β-D-glucose | 0.502 | 0.747 | 1.002 | 0.002 |
| L-2-amino-6-oximelic acid | 0.728 | 0.905 | 0.991 | -0.012 |
| 3-(pyrazol-1-yl)-L-alanine | 0.111 | 0.365 | 1.058 | 0.081 |
| 1-pyrroline-4-hydroxy-2-carboxylate | 0.656 | 0.859 | 1.019 | 0.027 |
| LPE(0:0/22:4) | 0.149 | 0.436 | 0.889 | -0.169 |
| LPE(22:4/0:0) | 0.149 | 0.436 | 0.889 | -0.169 |
| LPE(0:0/22:5) | 0.164 | 0.446 | 0.904 | -0.146 |
| LPE(22:5/0:0) | 0.164 | 0.446 | 0.904 | -0.146 |
| LPE(0:0/22:6) | 0.015 | 0.121 | 1.173 | 0.230 |
| LPE(22:6/0:0) | 0.015 | 0.121 | 1.173 | 0.230 |
| LPE(0:0/20:2) | 0.149 | 0.436 | 0.910 | -0.136 |
| LPE(20:2/0:0) | 0.149 | 0.436 | 0.910 | -0.136 |
| LPE(0:0/20:3) | 0.400 | 0.667 | 1.073 | 0.101 |
| LPE(20:3/0:0) | 0.400 | 0.667 | 1.073 | 0.101 |
| LPE(20:4/0:0) | 0.954 | 0.993 | 1.010 | 0.014 |
| LPE(0:0/20:5) | 0.002 | 0.046 | 1.307 | 0.387 |
| LPE(20:5/0:0) | 0.002 | 0.046 | 1.307 | 0.387 |
| LPE(0:0/18:0) | 0.022 | 0.149 | 1.128 | 0.173 |
| LPE(0:0/18:2) | 0.456 | 0.712 | 0.942 | -0.085 |
| LPE(0:0/16:0) | 0.020 | 0.149 | 1.114 | 0.156 |
| LPE(0:0/16:1) | 0.562 | 0.802 | 1.025 | 0.035 |
| 1-Aminocyclohexanoic acid | 0.041 | 0.207 | 1.083 | 0.115 |
| Ureidosuccinic acid | 0.852 | 0.961 | 0.933 | -0.101 |
| 2-Hydroxycaprylic acid | 0.074 | 0.291 | 0.833 | -0.264 |
| 2-hydroxyphenylacetic acid | 0.892 | 0.975 | 1.009 | 0.013 |
| 4-Hydroxy-3-methylbenzoic acid | 0.892 | 0.975 | 1.009 | 0.013 |
| 2-Hydroxy-2-Methyl Butyric acid | 0.454 | 0.712 | 0.920 | -0.120 |
| 2-Octenoic acid | 0.086 | 0.324 | 1.121 | 0.165 |
| 2-Methylglutaric Acid | 0.590 | 0.828 | 1.019 | 0.027 |
| (S)-Leucic acid | 0.322 | 0.605 | 0.945 | -0.081 |
| Glycohyodeoxycholic acid | 0.205 | 0.492 | 0.882 | -0.182 |
| N-Cinnamylglycine | 0.204 | 0.491 | 0.759 | -0.398 |
| (R)-(-)-2-Phenylpropionic Acid | 0.033 | 0.191 | 0.813 | -0.299 |
| 8-Aminooctanoic Acid | 0.024 | 0.156 | 1.028 | 0.040 |
| 4-Methoxysalicylic Acid | 0.930 | 0.990 | 0.868 | -0.204 |
| Tridecanedioic acid | 0.767 | 0.923 | 0.990 | -0.014 |
| 12-Hydroxyoctadecanoic acid | 0.017 | 0.136 | 0.958 | -0.062 |
| N-Palmitoylglycine | 0.703 | 0.887 | 0.990 | -0.014 |
| Taurolithocholic acid | 0.012 | 0.117 | 2.025 | 1.018 |
| 13(S)-HOTrE(γ) | 0.003 | 0.049 | 1.342 | 0.424 |
| 9(S)-HOTrE | 0.001 | 0.028 | 1.432 | 0.518 |
| (±)8-HETE | 0.088 | 0.326 | 1.225 | 0.292 |
| 8(S)-HETrE | 0.022 | 0.149 | 1.198 | 0.261 |
| 15(S)-HETrE | 0.022 | 0.149 | 1.198 | 0.261 |
| 9(S),12(S),13(S)-TriHOME | 0.449 | 0.709 | 1.020 | 0.029 |
| Bicyclo Prostaglandin E2 | 0.279 | 0.566 | 1.040 | 0.057 |
| 20-COOH-AA | 0.079 | 0.302 | 1.146 | 0.196 |
| Prostaglandin B2 | 0.246 | 0.535 | 1.002 | 0.003 |
| 6-trans-12-epi Leukotriene B4 | 0.167 | 0.446 | 1.150 | 0.201 |
| 6-trans Leukotriene B4 | 0.167 | 0.446 | 1.150 | 0.201 |
| 13-HDoHE | 0.003 | 0.049 | 1.170 | 0.226 |
| 10-HDoHE | 0.004 | 0.057 | 1.150 | 0.202 |
| 8-HDoHE | 0.009 | 0.103 | 1.134 | 0.182 |
| 11-HDoHE | 0.004 | 0.061 | 1.236 | 0.305 |
| 16-HDoHE | 0.020 | 0.149 | 1.188 | 0.248 |
| 20-HDoHE | 0.012 | 0.117 | 1.236 | 0.305 |
| 11-HEDE | 0.351 | 0.629 | 1.047 | 0.066 |
| 15-HEDE | 0.351 | 0.629 | 1.047 | 0.066 |
| (±)19(20)-EpDPE(A) | 0.010 | 0.107 | 1.130 | 0.176 |
| 11β-Prostaglandin E2 | 0.259 | 0.539 | 1.140 | 0.189 |
| Glu-Gln | 0.151 | 0.436 | 0.813 | -0.299 |
| Ethionamide | 0.383 | 0.654 | 1.040 | 0.057 |
| Glycerophospho-N-Arachidonoyl Ethanolamine | 0.954 | 0.993 | 1.010 | 0.014 |
| Testosterone sulfate | 0.140 | 0.430 | 1.266 | 0.340 |
| FFA(16:2) | 0.391 | 0.664 | 1.167 | 0.222 |
| (R)-(-)-Mandelic acid | 0.434 | 0.698 | 0.880 | -0.185 |
| (R)-3-Hydroxybutanoic acid | 0.890 | 0.975 | 1.028 | 0.039 |
| 2-Hydroxyhexanoic acid | 0.322 | 0.605 | 0.945 | -0.081 |
| 2'-O-methyluridine | 0.320 | 0.605 | 0.983 | -0.024 |
| 2-Phenylbutyric acid | 0.642 | 0.853 | 1.014 | 0.020 |
| 3-Hydroxycinnamic acid | 0.531 | 0.771 | 1.006 | 0.009 |
| 3-Methyluridine | 0.235 | 0.518 | 0.964 | -0.054 |
| 3-Phenoxybenzoic acid | 0.006 | 0.077 | 1.256 | 0.328 |
| 4-Ethyloctanoic acid | 0.788 | 0.930 | 1.003 | 0.005 |
| 4-Methoxyphenol | 0.107 | 0.365 | 0.810 | -0.304 |
| 5-Hydroxy-2'-deoxyuridine | 0.872 | 0.971 | 1.022 | 0.031 |
| 6-Hydroxyflavone (6-HF) | 0.064 | 0.272 | 1.244 | 0.315 |
| Acetylvaline | 0.149 | 0.436 | 1.034 | 0.048 |
| D-Galacturonic Acid | 0.022 | 0.149 | 1.083 | 0.116 |
| Dimethylmalonic acid | 0.777 | 0.925 | 0.962 | -0.056 |
| D-Tagatose | 0.221 | 0.504 | 0.950 | -0.073 |
| Val-Ala | 0.375 | 0.650 | 1.028 | 0.040 |
| Isethionic acid | 0.371 | 0.650 | 0.983 | -0.025 |
| Octadecanedioic acid | 0.018 | 0.142 | 0.847 | -0.239 |
| Sucrose 6′-monophosphate | 0.625 | 0.840 | 1.015 | 0.022 |
| Traumatic acid | 0.635 | 0.850 | 0.964 | -0.054 |
| Phosphatidylethanolamine lyso alkenyl 16:0 | 0.219 | 0.504 | 0.976 | -0.035 |
| LPA(16:0/0:0) | 0.173 | 0.453 | 1.022 | 0.031 |
| 3-Hydroxy-tetradecanoic acid | 0.874 | 0.971 | 0.982 | -0.027 |
| Acetanilide | 0.938 | 0.990 | 1.000 | 0.000 |
| FFA(22:4) | 0.491 | 0.737 | 0.963 | -0.054 |
| Cytochalasin H | 0.833 | 0.948 | 1.002 | 0.002 |
| Glu-Val | 0.019 | 0.145 | 1.097 | 0.134 |
| Isocitric acid | 0.200 | 0.490 | 1.049 | 0.069 |
| N-Acetyl-5-aminosalicylic acid | 0.952 | 0.993 | 0.933 | -0.101 |
| 2,4-Quinolinediol | 0.875 | 0.971 | 1.000 | 0.000 |
| Glu-Thr | 0.233 | 0.518 | 1.071 | 0.099 |
| Glu-Tyr | 0.000 | 0.021 | 1.238 | 0.309 |
| LPA(18:1/0:0) | 0.403 | 0.667 | 0.981 | -0.027 |
| CMPentylF | 0.017 | 0.134 | 1.139 | 0.188 |
| Barbital | 0.850 | 0.961 | 0.997 | -0.004 |
| Androsterone sulfate | 0.322 | 0.605 | 0.902 | -0.148 |
| Hydroxypiperazic acid | 0.002 | 0.045 | 0.877 | -0.190 |
| 2-Naphthalenesulfonic acid | 0.791 | 0.930 | 1.005 | 0.007 |
| 2-Deoxyribose 5'-phosphate | 0.687 | 0.879 | 0.977 | -0.034 |
| 3-(2-Naphthyl)-L-alanine | 0.228 | 0.513 | 0.987 | -0.019 |
| L-threo-3-Methylaspartate | 0.001 | 0.030 | 1.182 | 0.241 |
| FAHFA(8:0/10:0) | 0.022 | 0.149 | 1.670 | 0.739 |
| Leu-Ile | 0.665 | 0.864 | 0.909 | -0.138 |
| Docodiendioicacid | 0.477 | 0.723 | 1.039 | 0.055 |
| His-Ser | 0.039 | 0.205 | 1.223 | 0.290 |
| Phosphatidylethanolamine lyso alkenyl 18:2 | 0.257 | 0.538 | 1.119 | 0.162 |
| 2-Methylhexanoic acid | 0.924 | 0.987 | 0.960 | -0.059 |
| 3-(2-Hydroxyphenyl)propanoic acid | 0.379 | 0.653 | 0.996 | -0.006 |
| Salicyluric acid | 0.081 | 0.312 | 7.109 | 2.830 |
| 2-Octanamidoacetic acid | 0.216 | 0.504 | 0.943 | -0.085 |
| Palatinose | 0.963 | 0.995 | 1.058 | 0.082 |
| Tropine | 0.916 | 0.987 | 0.968 | -0.046 |
| M-toluene acetic acid | 0.105 | 0.364 | 0.783 | -0.353 |
| Piperic acid | 0.328 | 0.611 | 1.063 | 0.088 |
| Cholic acid | 0.980 | 0.997 | 0.981 | -0.028 |
| 7-Nitroindazole | 0.990 | 0.997 | 1.023 | 0.033 |
| 4-Methylhexanoic acid | 0.924 | 0.987 | 0.960 | -0.059 |
| 3-Methoxycatechol | 0.985 | 0.997 | 0.892 | -0.165 |
| 3,4-Dimethylbenzoic acid | 0.933 | 0.990 | 1.050 | 0.070 |
| 2,2-Dimethylpentanoic acid | 0.924 | 0.987 | 0.960 | -0.059 |
| D-Talose | 0.221 | 0.504 | 0.950 | -0.073 |
| D-Allose | 0.221 | 0.504 | 0.950 | -0.073 |
| 3,4-Dimethoxycinnamic acid | 0.284 | 0.575 | 1.117 | 0.160 |
| Naphthofluorescein | 0.183 | 0.468 | 1.143 | 0.193 |
| Zereno | 0.941 | 0.990 | 0.978 | -0.032 |
| FFA(16:1) | 0.652 | 0.858 | 0.971 | -0.042 |
| Dihydrodaidzein | 0.403 | 0.667 | 1.162 | 0.216 |
| 20-Hydroxy Prostaglandin F2α | 0.029 | 0.171 | 1.012 | 0.017 |
| Pinolenic acid | 0.001 | 0.028 | 1.217 | 0.284 |
| 15(R)-17-phenyl trinor prostaglandin F2α | 0.611 | 0.838 | 1.000 | 0.000 |
| O-1821 | 0.366 | 0.650 | 0.924 | -0.114 |
| 9,10-dihydroxystearic acid | 0.244 | 0.534 | 1.013 | 0.018 |
| (R)-3-Hydroxymyristic acid | 0.874 | 0.971 | 0.982 | -0.027 |
| 6,6'-Dihydroxy-5,5'-dimethoxybiphenyl-3,3'-dicarboxylic acid | 0.295 | 0.592 | 1.011 | 0.016 |
| 4-Oxoretinoic acid | 0.034 | 0.191 | 1.070 | 0.097 |
| N-Myristoylglycine | 0.749 | 0.912 | 0.980 | -0.029 |
| Isochodeoxycholic acid | 0.749 | 0.912 | 0.945 | -0.082 |
| Carbocyclic thromboxane A2 | 0.649 | 0.858 | 0.984 | -0.024 |
| Hydroferulic acid | 0.155 | 0.438 | 0.867 | -0.207 |
| (S)-2-Hydroxy-3-phenylpropanoic acid | 0.002 | 0.042 | 0.583 | -0.779 |
| Ethyl hydrogen malonate | 0.777 | 0.925 | 0.962 | -0.056 |
| L-Gulose | 0.479 | 0.723 | 0.972 | -0.042 |
| Deoxycholic acid | 0.784 | 0.930 | 1.008 | 0.012 |
| beta-Muricholic acid | 0.980 | 0.997 | 0.981 | -0.028 |
| Glycine deoxycholic acid | 0.222 | 0.504 | 1.016 | 0.023 |
| 3-Epideoxycholic acid | 0.876 | 0.971 | 1.019 | 0.028 |
| 5-Carboxyvanillic Acid | 0.872 | 0.971 | 0.997 | -0.004 |
| 4-Hydroxybenzoic Acid | 0.177 | 0.459 | 1.032 | 0.045 |
| Cys-Pro | 0.596 | 0.831 | 1.032 | 0.045 |
| Ser-Ala | 0.542 | 0.784 | 0.861 | -0.215 |
| Ala-Glu | 0.916 | 0.987 | 0.986 | -0.020 |
| S-Methyl-L-Cysteine-S-oxide | 0.183 | 0.468 | 1.071 | 0.099 |
| L-lyxose | 0.852 | 0.961 | 1.000 | 0.001 |
| Val-Asn | 0.776 | 0.925 | 0.945 | -0.081 |
| D-ribonate lithium salt | 0.119 | 0.382 | 1.077 | 0.107 |
| γ-Glu-Gln | 0.151 | 0.436 | 0.813 | -0.299 |
| Val-Thr | 0.453 | 0.712 | 1.004 | 0.005 |
| 2-keto-D-gluconic acid | 0.022 | 0.149 | 1.083 | 0.116 |
| Met-Asp | 0.206 | 0.493 | 0.986 | -0.020 |
| Val-Gly | 0.750 | 0.912 | 1.030 | 0.043 |
| Pyroglutamic acid | 0.551 | 0.792 | 1.041 | 0.058 |
| cyclo(gly-glu) | 0.003 | 0.049 | 1.353 | 0.437 |
| α-Hydroxyglutaric Acid (sodium salt) | 0.017 | 0.135 | 1.088 | 0.122 |
| cyclo(glu-glu) | 0.001 | 0.028 | 1.116 | 0.159 |
| Ile-Gly | 0.169 | 0.448 | 1.132 | 0.179 |
| Ile-Val | 0.919 | 0.987 | 0.940 | -0.090 |
| Asp-Leu | 0.004 | 0.057 | 1.175 | 0.233 |
| N-Acetyl-L-Glutamic Acid | 0.615 | 0.838 | 1.078 | 0.108 |
| Trp-Gly | 0.013 | 0.117 | 1.119 | 0.162 |
| 2-Hydroxy-3-Methyl Butanoic Acid | 0.435 | 0.698 | 0.931 | -0.103 |
| Salicylic acid β-D-O-glucuronic acid | 0.189 | 0.472 | 1.067 | 0.093 |
| Homovanillic Acid sulfate (sodium salt) | 0.286 | 0.576 | 0.926 | -0.111 |
| 4-Acetylaminobenzoic acid | 0.395 | 0.665 | 0.941 | -0.088 |
| Trp-Leu | 0.704 | 0.887 | 1.033 | 0.046 |
| 4-toluenesulfonic acid | 0.247 | 0.535 | 1.096 | 0.132 |
| 2,4-Dihydroxy-6-pentylbenzoic acid | 0.696 | 0.883 | 1.047 | 0.066 |
| Daidzein | 0.249 | 0.535 | 1.027 | 0.038 |
| D-Gulonic acid γ-lactone | 0.669 | 0.865 | 1.008 | 0.012 |
| L-Iditol | 0.308 | 0.600 | 1.020 | 0.028 |
| 3-Amino-5-hydroxybenzoic acid | 0.094 | 0.339 | 1.050 | 0.070 |
| 2-Methyllactic acid | 0.464 | 0.717 | 0.982 | -0.027 |
| Imidazole-4-methanol | 0.960 | 0.993 | 0.993 | -0.010 |
| (R)-2-Hydroxybutyric acid | 0.464 | 0.717 | 0.982 | -0.027 |
| 2-Methyl-3-hydroxybutyric acid | 0.050 | 0.234 | 1.089 | 0.123 |
| Pyrazine-2-carboxylic acid | 0.756 | 0.916 | 0.996 | -0.005 |
| (2s)-2-Amino-4-sulfinobutanoic acid | 0.910 | 0.985 | 1.013 | 0.018 |
| Ala-Ser | 0.892 | 0.975 | 0.969 | -0.046 |
| （2S，3R，4R，5R）-2,3,4,5,6-五羟基己醛 | 0.221 | 0.504 | 0.950 | -0.073 |
| L-rhamnonic acid | 0.656 | 0.859 | 0.979 | -0.030 |
| 3-Hydroxy-L-phenylalanine | 0.171 | 0.450 | 1.017 | 0.024 |
| Lys-Gly | 0.698 | 0.883 | 1.030 | 0.043 |
| 2-(Acetylamino)-2-deoxy-A-D-glucopyranose | 0.943 | 0.992 | 1.049 | 0.069 |
| Hyp-Thr | 0.716 | 0.898 | 0.999 | -0.002 |
| γ-Glu-Met | 0.268 | 0.554 | 0.906 | -0.143 |
| Lys-Phe | 0.990 | 0.997 | 0.995 | -0.007 |
| Met-Phe | 0.200 | 0.490 | 1.113 | 0.154 |
| Dipyrocetyl | 0.130 | 0.410 | 1.107 | 0.147 |
| 4-(Hydroxyamino)quinoline 1-oxide | 0.072 | 0.289 | 1.062 | 0.086 |
| P-Toluenesulfonamide | 0.272 | 0.561 | 1.144 | 0.195 |
| 4-Hydroxyquinoline | 0.319 | 0.605 | 1.009 | 0.012 |
| 2-Phenyl-5-benzimidazole sulfonic acid | 0.936 | 0.990 | 0.979 | -0.031 |
| 10-Hydroxystearic Acid | 0.099 | 0.350 | 0.967 | -0.048 |
| L-Glycine | 0.017 | 0.134 | 0.856 | -0.224 |
| L-Cystine | 0.555 | 0.795 | 0.977 | -0.034 |
| L-Tyrosine | 0.037 | 0.196 | 1.067 | 0.093 |
| L-Ornithine | 0.380 | 0.654 | 1.081 | 0.113 |
| L-Alanine | 0.796 | 0.930 | 0.986 | -0.021 |
| L-Histidine | 0.154 | 0.436 | 1.021 | 0.029 |
| L-Methionine | 0.152 | 0.436 | 0.969 | -0.046 |
| L-Proline | 0.028 | 0.170 | 1.137 | 0.186 |
| L-Valine | 0.000 | 0.008 | 1.134 | 0.181 |
| 5-Oxoproline | 0.899 | 0.977 | 1.004 | 0.006 |
| Betaine | 0.351 | 0.629 | 0.930 | -0.104 |
| Glyc-Pro | 0.768 | 0.923 | 1.027 | 0.039 |
| L-Cysteine | 0.011 | 0.116 | 0.955 | -0.066 |
| N6-Acetyl-L-Lysine | 0.040 | 0.206 | 1.055 | 0.077 |
| N-Acetylcysteine | 0.646 | 0.856 | 0.995 | -0.008 |
| N-Acetylputrescine | 0.622 | 0.838 | 1.007 | 0.010 |
| Serotonin | 0.855 | 0.961 | 1.060 | 0.084 |
| Trimethylamine-N-Oxide | 0.320 | 0.605 | 1.103 | 0.142 |
| Ala-Lys | 0.278 | 0.566 | 0.953 | -0.070 |
| N-Acetylhistamine | 0.243 | 0.533 | 1.050 | 0.070 |
| P-Coumaric Acid | 0.054 | 0.246 | 1.061 | 0.086 |
| 1,4-Dihydro-1-Methyl-4-Oxo-3-Pyridinecarboxamide | 0.345 | 0.627 | 1.051 | 0.071 |
| Theobromine | 0.852 | 0.961 | 0.882 | -0.181 |
| Choline | 0.459 | 0.713 | 1.028 | 0.040 |
| 1,5-Diaminopentane | 0.085 | 0.321 | 0.886 | -0.175 |
| Diethanolamine | 0.186 | 0.470 | 0.958 | -0.062 |
| Myoinositol | 0.291 | 0.585 | 0.992 | -0.012 |
| 1-Methylhistidine | 0.218 | 0.504 | 0.942 | -0.085 |
| 5,6-Dihydro-5-Methyluracil | 0.420 | 0.684 | 0.972 | -0.041 |
| 5-Methyluridine | 0.071 | 0.286 | 0.932 | -0.102 |
| Adenine | 0.090 | 0.333 | 1.074 | 0.103 |
| Cytosine | 0.597 | 0.831 | 1.022 | 0.032 |
| Purine | 0.000 | 0.008 | 1.104 | 0.142 |
| Uracil | 0.049 | 0.230 | 1.084 | 0.117 |
| 3,3',5-Triiodo-L-Thyronine | 0.981 | 0.997 | 0.995 | -0.007 |
| N-Methyltryptamine | 0.578 | 0.821 | 0.612 | -0.708 |
| D-Fructose | 0.435 | 0.698 | 0.984 | -0.024 |
| D-Mannose | 0.435 | 0.698 | 0.984 | -0.024 |
| D-Gluconic Acid | 0.160 | 0.443 | 1.056 | 0.079 |
| Orotic Acid | 0.671 | 0.866 | 1.008 | 0.011 |
| Nicotinamide | 0.037 | 0.198 | 1.141 | 0.191 |
| Riboflavin | 0.339 | 0.624 | 1.044 | 0.062 |
| Trigonelline | 0.469 | 0.719 | 1.118 | 0.161 |
| 3-Indolebutyric Acid | 0.368 | 0.650 | 1.012 | 0.018 |
| 2-Aminoethanesulfonic Acid | 0.515 | 0.760 | 1.011 | 0.015 |
| 4-Guanidinobutyric Acid | 0.197 | 0.485 | 0.913 | -0.131 |
| 5-Aminovaleric Acid | 0.059 | 0.261 | 0.863 | -0.213 |
| 6-Aminocaproic-Acid | 0.001 | 0.030 | 1.069 | 0.096 |
| 7-Methyluric Acid | 0.452 | 0.712 | 1.007 | 0.011 |
| Creatinine | 0.802 | 0.931 | 1.007 | 0.011 |
| Dl-2-Aminooctanoic Acid | 0.036 | 0.193 | 0.834 | -0.261 |
| Guanidineacetic Acid | 0.956 | 0.993 | 1.065 | 0.091 |
| L-Dihydroorotic Acid | 0.940 | 0.990 | 0.996 | -0.006 |
| L-Homoserine | 0.006 | 0.072 | 1.061 | 0.086 |
| Maleic Acid | 0.068 | 0.280 | 1.066 | 0.092 |
| LPC(0:0/14:0) | 0.005 | 0.067 | 1.167 | 0.223 |
| LPC(16:0/0:0) | 0.051 | 0.235 | 1.016 | 0.022 |
| Trans-3-Hydroxycotinine | 0.735 | 0.910 | 1.028 | 0.040 |
| L-Homoarginine | 0.025 | 0.157 | 1.129 | 0.175 |
| Pantetheine | 0.273 | 0.561 | 1.022 | 0.032 |
| D-piperidine acid | 0.899 | 0.977 | 1.004 | 0.006 |
| 5'-Deoxy-5'-(Methylthio) Adenosine | 0.167 | 0.446 | 1.033 | 0.047 |
| Sarcosine | 0.438 | 0.701 | 0.979 | -0.030 |
| Imidazoleacetic acid | 0.188 | 0.472 | 0.941 | -0.087 |
| 2-Aminoadipic Acid | 0.146 | 0.436 | 1.173 | 0.231 |
| LPC(17:0/0:0) | 0.327 | 0.610 | 1.016 | 0.023 |
| Sn-Glycero-3-Phosphocholine | 0.093 | 0.339 | 1.114 | 0.155 |
| Indole | 0.250 | 0.535 | 1.017 | 0.024 |
| LPC(15:0/0:0) | 0.003 | 0.049 | 1.126 | 0.171 |
| LPC(0:0/18:2) | 0.383 | 0.654 | 0.984 | -0.023 |
| 2-Hydroxycinnamic acid | 0.210 | 0.498 | 1.015 | 0.021 |
| Carnitine C2:0 | 0.803 | 0.931 | 1.040 | 0.056 |
| DL-Stachydrine | 0.904 | 0.981 | 0.978 | -0.032 |
| L-Norleucine | 0.001 | 0.034 | 1.058 | 0.081 |
| DL-Carnitine | 0.509 | 0.755 | 0.972 | -0.041 |
| 6-Dimethylaminopurine | 0.256 | 0.538 | 1.051 | 0.072 |
| Triethyl-phosphate | 0.485 | 0.729 | 1.051 | 0.072 |
| LPC(O-16:0/2:0) | 0.105 | 0.364 | 1.061 | 0.085 |
| 18-Hydroxycorticosterone | 0.035 | 0.193 | 1.060 | 0.084 |
| Oleamide | 0.791 | 0.930 | 1.100 | 0.137 |
| Carnitine isoC4:0 | 0.298 | 0.593 | 0.894 | -0.161 |
| 2'-Hydroxy-5'-methylacetophenone | 0.646 | 0.856 | 1.013 | 0.019 |
| Spermidine | 0.469 | 0.719 | 0.979 | -0.031 |
| N-Acetyl-L-Histidine | 0.398 | 0.667 | 1.055 | 0.077 |
| 2-Furoylglycine | 0.184 | 0.468 | 1.234 | 0.303 |
| Carnitine-2-methyl-C4 | 0.068 | 0.280 | 1.106 | 0.146 |
| Isonicotinic acid | 0.433 | 0.698 | 0.991 | -0.014 |
| Phe-Pro | 0.530 | 0.771 | 1.062 | 0.087 |
| LPE(16:1/0:0) | 0.496 | 0.739 | 1.108 | 0.148 |
| 3-Chloroaniline | 0.003 | 0.049 | 1.029 | 0.041 |
| Methylcysteine | 0.213 | 0.503 | 1.070 | 0.098 |
| DL-Leucine | 0.001 | 0.030 | 1.069 | 0.096 |
| 6-Methylnicotinamide | 0.990 | 0.997 | 0.976 | -0.035 |
| (R)-2-Hydroxy-3-phenylpropionic-acid | 0.366 | 0.650 | 1.042 | 0.060 |
| 1-Aminopropan-2-ol | 0.320 | 0.605 | 1.103 | 0.142 |
| N-Methylalanine | 0.855 | 0.961 | 1.025 | 0.035 |
| Dihydro-D-sphingosine | 0.045 | 0.216 | 0.909 | -0.138 |
| Hypaphorine | 0.924 | 0.987 | 1.062 | 0.087 |
| N-Methyl-L-Glutamate | 0.146 | 0.436 | 1.173 | 0.231 |
| Phosphocholine | 0.277 | 0.566 | 0.987 | -0.019 |
| 2,4-Dihydroxypteridine | 0.338 | 0.623 | 1.036 | 0.052 |
| Cortisol | 0.013 | 0.117 | 0.862 | -0.214 |
| L-Tryptophanamide | 0.028 | 0.168 | 1.037 | 0.052 |
| Catechol | 0.141 | 0.430 | 1.009 | 0.013 |
| Acetylcholine | 0.738 | 0.910 | 0.979 | -0.030 |
| 1-Hydroxylamino-2-phenylethane | 0.003 | 0.049 | 1.054 | 0.076 |
| β-Alanine | 0.796 | 0.930 | 0.986 | -0.021 |
| D-(+)-sucrose | 0.893 | 0.975 | 1.029 | 0.042 |
| Thr-Phe | 0.941 | 0.990 | 0.972 | -0.041 |
| Salicylaldehyde | 0.403 | 0.667 | 0.944 | -0.083 |
| Benzaldehyde | 0.000 | 0.016 | 1.081 | 0.113 |
| Biliverdin | 0.116 | 0.374 | 1.199 | 0.262 |
| N,-N-diacetyl-O-methylhydroxylamine | 0.795 | 0.930 | 0.982 | -0.026 |
| Hydroxyquinoline | 0.005 | 0.063 | 1.073 | 0.101 |
| DL-O-tyrosine | 0.000 | 0.008 | 1.049 | 0.070 |
| 7-Methylguanosine | 0.689 | 0.879 | 1.000 | -0.001 |
| 6-O-methylguanine | 0.108 | 0.365 | 1.040 | 0.057 |
| Oxypurinol | 0.035 | 0.193 | 1.142 | 0.191 |
| Carnitine C12:0 | 0.607 | 0.838 | 1.022 | 0.032 |
| Allopurinol | 0.071 | 0.286 | 1.271 | 0.346 |
| PC(12:0/12:0) | 0.476 | 0.723 | 1.053 | 0.075 |
| (R)-(-)-2-phenylglycine | 0.003 | 0.049 | 1.054 | 0.076 |
| 1-acetylindole | 0.001 | 0.028 | 1.064 | 0.089 |
| 3-Carboxypropyltrimethylammonium | 0.559 | 0.799 | 0.952 | -0.071 |
| L-Isoleucine | 0.001 | 0.034 | 1.070 | 0.098 |
| LPC(O-18:0/0:0) | 0.111 | 0.365 | 0.954 | -0.068 |
| Creatine phosphate | 0.136 | 0.424 | 1.065 | 0.091 |
| PC(O-16:0/O-2:0) | 0.111 | 0.365 | 0.954 | -0.068 |
| Butenoyl-PAF | 0.153 | 0.436 | 0.958 | -0.062 |
| N-Methyl-α-aminoisobutyric acid | 0.405 | 0.667 | 0.958 | -0.062 |
| Biotinamide | 0.235 | 0.518 | 1.038 | 0.054 |
| 8,8a-deoxy-oleane | 0.826 | 0.944 | 0.977 | -0.034 |
| N-acetylpyrrolidine | 0.026 | 0.159 | 1.102 | 0.141 |
| 1,3-Dicyclohexylurea | 0.349 | 0.629 | 1.063 | 0.087 |
| PC(O-16:0/O-1:0) | 0.032 | 0.187 | 1.024 | 0.035 |
| 4-tert-butylbenzoic-acid | 0.986 | 0.997 | 0.997 | -0.004 |
| 4-Hydroxytryptamine | 0.175 | 0.457 | 0.826 | -0.275 |
| 1-Deoxyvaleric-acid | 0.546 | 0.789 | 0.989 | -0.015 |
| 2-Mercaptobenzothiazole | 0.135 | 0.422 | 0.963 | -0.055 |
| Urobilin | 0.392 | 0.664 | 1.259 | 0.332 |
| 20,26-dihydroxyecdysone | 0.160 | 0.443 | 1.015 | 0.021 |
| Carnitine C6:0 | 0.960 | 0.993 | 1.031 | 0.044 |
| Leu-Gly | 0.346 | 0.627 | 1.037 | 0.053 |
| LPC(0:0/22:4) | 0.252 | 0.538 | 0.927 | -0.109 |
| LPC(22:4/0:0) | 0.252 | 0.538 | 0.927 | -0.109 |
| LPC(0:0/22:5) | 0.954 | 0.993 | 0.997 | -0.005 |
| LPC(22:5/0:0) | 0.021 | 0.149 | 1.174 | 0.231 |
| LPC(20:1/0:0) | 0.153 | 0.436 | 0.958 | -0.062 |
| LPC(20:2/0:0) | 0.827 | 0.944 | 1.017 | 0.024 |
| LPC(0:0/20:2) | 0.827 | 0.944 | 1.017 | 0.024 |
| LPC(0:0/20:3) | 0.041 | 0.207 | 1.097 | 0.134 |
| LPC(20:3/0:0) | 0.041 | 0.207 | 1.097 | 0.134 |
| LPC(0:0/20:4) | 0.077 | 0.298 | 1.071 | 0.098 |
| LPC(20:4/0:0) | 0.077 | 0.298 | 1.071 | 0.098 |
| LPC(18:0/0:0) | 0.105 | 0.364 | 1.061 | 0.085 |
| LPC(18:1/0:0) | 0.169 | 0.448 | 0.935 | -0.097 |
| LPC(18:2/0:0) | 0.383 | 0.654 | 0.984 | -0.023 |
| LPC(0:0/16:0) | 0.051 | 0.235 | 1.016 | 0.022 |
| LPC(16:1/0:0) | 0.588 | 0.828 | 1.041 | 0.059 |
| Mycosporine-glycine | 0.007 | 0.077 | 0.845 | -0.242 |
| Carnitine C18:0 | 0.031 | 0.182 | 0.928 | -0.109 |
| Carnitine C18:2 | 0.276 | 0.566 | 1.089 | 0.124 |
| Carnitine C16:0 | 0.665 | 0.864 | 1.035 | 0.050 |
| Carnitine C16:1 | 0.985 | 0.997 | 1.033 | 0.046 |
| Carnitine C16:2 | 0.357 | 0.639 | 1.107 | 0.146 |
| Carnitine C14-OH | 0.936 | 0.990 | 1.015 | 0.021 |
| Carnitine C14:2-OH | 0.326 | 0.610 | 1.063 | 0.089 |
| Carnitine C14:2 | 0.753 | 0.913 | 1.100 | 0.137 |
| Carnitine C12-OH | 0.808 | 0.935 | 0.981 | -0.028 |
| Carnitine C11:DC | 0.789 | 0.930 | 1.031 | 0.044 |
| Carnitine C13:1 | 0.021 | 0.149 | 1.171 | 0.228 |
| Carnitine C11:0 | 0.044 | 0.211 | 1.217 | 0.283 |
| Carnitine C11:1 | 0.005 | 0.063 | 1.224 | 0.292 |
| Carnitine C10:0 | 0.527 | 0.771 | 0.956 | -0.066 |
| Carnitine C8-OH | 0.648 | 0.858 | 1.013 | 0.019 |
| Carnitine C9:0 | 0.122 | 0.389 | 1.211 | 0.276 |
| Carnitine C8:0 | 0.492 | 0.737 | 0.994 | -0.009 |
| Carnitine C8:1 | 0.002 | 0.047 | 1.672 | 0.741 |
| Carnitine ph-C1 | 0.974 | 0.997 | 0.810 | -0.304 |
| Carnitine C4:DC | 0.185 | 0.469 | 0.919 | -0.121 |
| Carnitine C5:0 | 0.068 | 0.280 | 1.106 | 0.146 |
| Carnitine C5:1 | 0.144 | 0.436 | 1.102 | 0.140 |
| Carnitine C4:0 | 0.298 | 0.593 | 0.894 | -0.161 |
| 1,3-Diphenylguanidine | 0.084 | 0.319 | 0.886 | -0.174 |
| N'-Methyl-2-pyridone-5-carboxamide | 0.025 | 0.156 | 1.130 | 0.176 |
| 5-Methoxytryptamine | 0.001 | 0.029 | 1.062 | 0.086 |
| L-Phenylephrine | 0.056 | 0.249 | 0.933 | -0.100 |
| Theophylline | 0.361 | 0.645 | 1.195 | 0.258 |
| SDMA | 0.012 | 0.117 | 1.066 | 0.093 |
| Methylguanidine | 0.111 | 0.365 | 1.067 | 0.093 |
| N6-methyladenosine | 0.370 | 0.650 | 1.053 | 0.075 |
| 8-Azaguanine | 0.035 | 0.193 | 1.142 | 0.191 |
| Isocytosine | 0.597 | 0.831 | 1.022 | 0.032 |
| Leu-Val | 0.969 | 0.996 | 0.909 | -0.137 |
| Phe-Asn | 0.374 | 0.650 | 1.000 | 0.000 |
| Phe-Met | 0.024 | 0.156 | 1.153 | 0.205 |
| Glu-Met | 0.540 | 0.783 | 0.970 | -0.043 |
| Met-Glu | 0.583 | 0.826 | 0.984 | -0.024 |
| Phe-Val | 0.055 | 0.249 | 1.121 | 0.165 |
| Carnitine C10:1 | 0.463 | 0.717 | 1.095 | 0.131 |
| Carnitine C14:2:DC | 0.824 | 0.944 | 1.011 | 0.016 |
| Caldine | 0.300 | 0.596 | 0.952 | -0.071 |
| 1-Methyladenosine | 0.370 | 0.650 | 1.053 | 0.075 |
| PC(O-16:0/0:0) | 0.986 | 0.997 | 1.022 | 0.031 |
| LPC(O-18:1/0:0) | 0.839 | 0.954 | 0.970 | -0.044 |
| LPC(O-0:0/18:0) | 0.111 | 0.365 | 0.954 | -0.068 |
| NE,NE,NE-TRIMETHYLLYSINE | 0.362 | 0.645 | 1.039 | 0.055 |
| N,N-Dimethylarginine | 0.504 | 0.749 | 1.005 | 0.007 |
| Carnitine C3:0 | 0.867 | 0.971 | 1.085 | 0.117 |
| Phe-Glu | 0.659 | 0.860 | 1.041 | 0.059 |
| Gly-Gly-Phe | 0.004 | 0.056 | 1.065 | 0.091 |
| LPC(0:0/17:0) | 0.134 | 0.422 | 1.017 | 0.024 |
| LPC(0:0/15:0) | 0.003 | 0.049 | 1.126 | 0.171 |
| LPC(12:0/0:0) | 0.112 | 0.366 | 1.240 | 0.310 |
| LPC(O-16:1/0:0) | 0.424 | 0.688 | 0.979 | -0.031 |
| Sphingosyl-phosphocholine | 0.091 | 0.335 | 1.061 | 0.085 |
| LPC(0:0/16:1) | 0.588 | 0.828 | 1.041 | 0.059 |
| LPC(0:0/18:0) | 0.105 | 0.364 | 1.061 | 0.085 |
| LPC(0:0/18:1) | 0.169 | 0.448 | 0.935 | -0.097 |
| LPC(0:0/20:1) | 0.153 | 0.436 | 0.958 | -0.062 |
| Methyldopa | 0.313 | 0.605 | 0.933 | -0.100 |
| (E)-Guggulsterone | 0.013 | 0.117 | 1.052 | 0.073 |
| (Z)-Guggulsterone | 0.013 | 0.117 | 1.052 | 0.073 |
| 1-Methylinosine | 0.220 | 0.504 | 1.017 | 0.024 |
| 2'-O-methylcytidine | 0.214 | 0.503 | 0.906 | -0.142 |
| Acrylamide | 0.001 | 0.033 | 1.121 | 0.164 |
| Agmatine | 0.085 | 0.321 | 1.049 | 0.069 |
| Ala-Phe | 0.073 | 0.291 | 1.020 | 0.028 |
| Cyromazine | 0.092 | 0.339 | 1.082 | 0.114 |
| Cytidine 5'-diphosphate | 0.890 | 0.975 | 1.020 | 0.029 |
| L-Allothreonine | 0.006 | 0.072 | 1.061 | 0.086 |
| D-Glucosaminic acid | 0.061 | 0.264 | 1.141 | 0.191 |
| Guanidine | 0.248 | 0.535 | 1.094 | 0.130 |
| Leu-Ala | 0.752 | 0.913 | 0.994 | -0.009 |
| L-Norvaline | 0.000 | 0.008 | 1.134 | 0.181 |
| Leu-Phe | 0.584 | 0.826 | 0.931 | -0.103 |
| N4-Acetylcytidine | 0.171 | 0.450 | 0.876 | -0.192 |
| Gly-Ile | 0.604 | 0.836 | 1.048 | 0.068 |
| Palmitoylethanolamide（(PEA） | 0.567 | 0.807 | 0.983 | -0.024 |
| Sanguinarine | 0.856 | 0.961 | 1.027 | 0.038 |
| Stachydrine | 0.815 | 0.938 | 0.926 | -0.111 |
| Synephrine | 0.585 | 0.826 | 0.997 | -0.004 |
| Bicine | 0.257 | 0.538 | 1.017 | 0.024 |
| Eicosanoyl-EA | 0.106 | 0.364 | 0.913 | -0.131 |
| Gln-Phe | 0.124 | 0.394 | 1.091 | 0.125 |
| Ile-Met | 0.025 | 0.156 | 1.123 | 0.167 |
| LPE(17:1/0:0) | 0.192 | 0.477 | 1.066 | 0.093 |
| Pro-Ile | 0.000 | 0.025 | 1.203 | 0.266 |
| Ser-Leu | 0.749 | 0.912 | 0.926 | -0.111 |
| Ser-Phe | 0.425 | 0.688 | 1.070 | 0.098 |
| Tyr-Leu | 0.117 | 0.379 | 0.854 | -0.228 |
| 3-Hydroxyphenylurea | 0.249 | 0.535 | 1.022 | 0.032 |
| Caffeine | 0.968 | 0.996 | 0.865 | -0.210 |
| Carnitine C9:1-OH | 0.007 | 0.077 | 1.750 | 0.808 |
| LPE(18:2/0:0) | 0.466 | 0.719 | 0.892 | -0.165 |
| (E,Z)-2-Amino-3,14-octadecadien-1-ol | 0.791 | 0.930 | 1.100 | 0.137 |
| Phe-Trp | 0.325 | 0.609 | 0.964 | -0.053 |
| N(Alpha)-Acetyl-Epsilon-(2-Propenal)Lysine | 0.019 | 0.145 | 1.166 | 0.221 |
| Cyclo(Pro-Leu) | 0.001 | 0.028 | 1.226 | 0.293 |
| Thr-Gln | 0.007 | 0.077 | 0.679 | -0.559 |
| Cyclo(Phe-Glu) | 0.314 | 0.605 | 1.132 | 0.179 |
| Cyclo(Pro-Val) | 0.110 | 0.365 | 1.061 | 0.086 |
| Ile-Asp | 0.014 | 0.117 | 1.259 | 0.332 |
| N-MethyTrans-4-Hydroxy-Proline | 0.892 | 0.975 | 1.095 | 0.131 |
| Ser-Ile | 0.749 | 0.912 | 0.926 | -0.111 |
| 5-Aminoimidazole ribonucleotide | 0.149 | 0.436 | 1.083 | 0.115 |
| N6-(2-Hydroxyethyl)adenosine | 0.305 | 0.599 | 1.017 | 0.024 |
| 7-(alpha-D-glucosyl)-N(6)-isopentenyladenine | 0.035 | 0.193 | 1.199 | 0.261 |
| D-Proline-betaine | 0.621 | 0.838 | 1.303 | 0.382 |
| 2-Methyl-1-Pyrroline | 0.014 | 0.119 | 1.067 | 0.093 |
| Melibiose | 0.183 | 0.468 | 1.083 | 0.115 |
| N-Formylglycine | 0.622 | 0.838 | 1.040 | 0.057 |
| D-Ornithine | 0.013 | 0.117 | 1.120 | 0.164 |
| Dehydroascorbic-acid | 0.374 | 0.650 | 0.964 | -0.053 |
| Androstenediol | 0.749 | 0.912 | 1.006 | 0.008 |
| Triethylenetetramine | 0.616 | 0.838 | 1.000 | -0.001 |
| FFA(10:1) | 0.707 | 0.889 | 0.943 | -0.084 |
| 3-Aminoquinoline | 0.002 | 0.045 | 1.057 | 0.080 |
| L-Isserine | 0.040 | 0.206 | 1.052 | 0.074 |
| 1-Amino-1-cyclobutane-carboxylic-acid | 0.028 | 0.170 | 1.137 | 0.186 |
| N,N-Bis(2-hydroxyethyl)dodecanamide | 0.024 | 0.156 | 0.926 | -0.110 |
| (E)-8-Methyl-6-nonenoic-acid | 0.794 | 0.930 | 0.980 | -0.030 |
| Cork-oximate | 0.778 | 0.925 | 1.068 | 0.095 |
| 8-iso-15-keto-Prostaglandin-F2α | 0.722 | 0.902 | 0.945 | -0.081 |
| 2-Amino-3-phosphonopropionic-acid | 0.256 | 0.538 | 0.936 | -0.095 |
| Inositol 1,3,4-trisphosphate | 0.979 | 0.997 | 0.999 | -0.001 |
| S-methyl-L-thiocitrulline | 0.443 | 0.703 | 0.991 | -0.013 |
| ST-638 | 0.589 | 0.828 | 0.825 | -0.277 |
| Leu-Leu | 0.623 | 0.838 | 0.766 | -0.384 |
| Gly-Lys | 0.946 | 0.993 | 0.970 | -0.044 |
| Gly-Gln | 0.345 | 0.627 | 0.971 | -0.042 |
| Lys-Ser | 0.111 | 0.365 | 0.969 | -0.045 |
| Arg-Glu | 0.999 | 1.000 | 0.994 | -0.009 |
| Glu-His | 0.068 | 0.280 | 1.112 | 0.153 |
| Cyclocreatine | 0.684 | 0.878 | 1.022 | 0.031 |
| γ-Glu-Lys | 0.122 | 0.389 | 0.788 | -0.344 |
| (R)-(-)-1-Amino-2-propanol | 0.607 | 0.838 | 0.990 | -0.015 |
| Gly-Thr | 0.960 | 0.993 | 0.998 | -0.003 |
| Pro-Ser | 0.958 | 0.993 | 1.003 | 0.004 |
| N-Ethylglycine | 0.855 | 0.961 | 1.025 | 0.035 |
| 3-Guanidinopropionic acid | 0.095 | 0.340 | 1.049 | 0.068 |
| Glu-Ser | 0.768 | 0.923 | 0.984 | -0.023 |
| Ammeline | 0.194 | 0.480 | 0.978 | -0.033 |
| Thr-Glu | 0.162 | 0.446 | 1.078 | 0.109 |
| Glu-Cit | 0.404 | 0.667 | 0.972 | -0.040 |
| Glu-Gly | 0.801 | 0.931 | 0.985 | -0.022 |
| N-acetyl-D-Lactosamine | 0.511 | 0.757 | 1.060 | 0.084 |
| (+/-)-High-Proline | 0.043 | 0.210 | 1.252 | 0.324 |
| 3-(imidazol-4-yl)propionic-acid | 0.619 | 0.838 | 0.986 | -0.020 |
| Ser-Pro | 0.254 | 0.538 | 0.954 | -0.067 |
| N1-Acetylspermidine | 0.410 | 0.674 | 1.019 | 0.028 |
| Ser-Val | 0.637 | 0.850 | 0.969 | -0.045 |
| Ile-Gln | 0.307 | 0.600 | 1.082 | 0.113 |
| Ile-Ser | 0.547 | 0.789 | 1.074 | 0.103 |
| Tyr-Glu | 0.724 | 0.904 | 1.013 | 0.018 |
| Ile-Glu | 0.004 | 0.059 | 1.303 | 0.381 |
| Leu-Glu | 0.004 | 0.059 | 1.303 | 0.381 |
| Glu-Ile | 0.004 | 0.059 | 1.303 | 0.381 |
| Ile-Thr | 0.940 | 0.990 | 1.043 | 0.061 |
| Leu-Thr | 0.940 | 0.990 | 1.043 | 0.061 |
| 2,2'-Cyclouridine | 0.223 | 0.504 | 1.048 | 0.068 |
| 3-aminobenzamide | 0.064 | 0.272 | 1.405 | 0.491 |
| N-(2-hydroxyethyl)-3-pyridinecarboxamide | 0.000 | 0.021 | 1.078 | 0.109 |
| Phe-Ala-Ser | 0.006 | 0.072 | 1.276 | 0.351 |
| Ser-Phe-Ala | 0.006 | 0.072 | 1.276 | 0.351 |
| 1-Methylguanosine | 0.689 | 0.879 | 1.000 | -0.001 |
| Cyclo(Ala-Pro) | 0.322 | 0.605 | 1.032 | 0.045 |
| Phe-Gly | 0.413 | 0.678 | 1.027 | 0.038 |
| Val-Ile | 0.638 | 0.850 | 1.092 | 0.126 |
| Val-Leu | 0.638 | 0.850 | 1.092 | 0.126 |
| Asp-Ile | 0.014 | 0.117 | 1.259 | 0.332 |
| Leu-Met | 0.025 | 0.156 | 1.123 | 0.167 |
| cyclo(pro-pro) | 0.099 | 0.350 | 1.144 | 0.194 |
| Phe-Tyr | 0.085 | 0.321 | 1.138 | 0.186 |
| Ile-Leu | 0.623 | 0.838 | 0.766 | -0.384 |
| Phe-Ala-Leu | 0.308 | 0.600 | 0.960 | -0.059 |
| Securinine | 0.799 | 0.931 | 0.977 | -0.034 |
| Thr-Arg | 0.621 | 0.838 | 1.009 | 0.014 |
| Arg-Thr | 0.564 | 0.803 | 0.960 | -0.059 |
| Leu-Asn | 0.013 | 0.117 | 1.082 | 0.113 |
| Glu-Phe-Ala | 0.153 | 0.436 | 1.115 | 0.157 |
| 4-Amino-3-hydroxybutyric acid | 0.230 | 0.514 | 1.123 | 0.168 |
| D-Allo-Isoleucine | 0.001 | 0.034 | 1.070 | 0.098 |
| 5-Acetylamino-6-amino-3-methyluracil | 0.803 | 0.931 | 1.002 | 0.003 |
| Gln-Gly | 0.653 | 0.858 | 1.020 | 0.029 |
| Gly-Glu | 0.801 | 0.931 | 0.985 | -0.022 |
| Lys-Ala | 0.278 | 0.566 | 0.953 | -0.070 |
| Pro-Asn | 0.035 | 0.193 | 1.148 | 0.199 |
| Pro-Asp | 0.004 | 0.057 | 1.361 | 0.444 |
| Arg-Gly | 0.738 | 0.910 | 0.974 | -0.038 |
| Gly-Arg | 0.738 | 0.910 | 0.974 | -0.038 |
| Ser-Lys | 0.654 | 0.859 | 1.001 | 0.002 |
| Ser-Glu | 0.982 | 0.997 | 0.962 | -0.056 |
| Phe-Ala | 0.000 | 0.021 | 1.057 | 0.081 |
| Ile-Asn | 0.013 | 0.117 | 1.082 | 0.113 |
| Leu-Asp | 0.041 | 0.207 | 1.206 | 0.270 |
| Phe-Thr | 0.941 | 0.990 | 0.972 | -0.041 |
| Phe-Hyp | 0.824 | 0.944 | 1.015 | 0.022 |
| Glu-Phe | 0.659 | 0.860 | 1.041 | 0.059 |
| Glu-Arg | 0.947 | 0.993 | 1.046 | 0.065 |
| 2-Butyl-3-(4-hydroxybenzoyl)benzofuran | 0.002 | 0.042 | 0.835 | -0.260 |
| Quinmerac | 0.348 | 0.629 | 1.029 | 0.041 |
| Chaps | 0.317 | 0.605 | 0.879 | -0.186 |
| trans-resveratrol-3-O-sulfate | 0.301 | 0.596 | 0.972 | -0.040 |

**Table S11.** Associations between characteristic microbiota and differential metabolites in group 3 and group 4 of males.

| Group | Bacteria_genus | Metabolites | Correlation | P_value | Significant |
| --- | --- | --- | --- | --- | --- |
| group 3 | CHKCI002 | (±)12-HEPE | -0.196 | 0.004 | yes |
| group 3 | CHKCI002 | (±)15-HEPE | -0.196 | 0.004 | yes |
| group 3 | CHKCI002 | (±)17-HDHA | -0.148 | 0.036 | yes |
| group 3 | CHKCI002 | (±)18-HEPE | -0.196 | 0.004 | yes |
| group 3 | CHKCI002 | (±)5-HEPE | -0.171 | 0.011 | yes |
| group 3 | CHKCI002 | (R)-3-Hydroxybutanoic acid | 0.024 | 0.816 | no |
| group 3 | CHKCI002 | 1-Aminopropan-2-ol | 0.039 | 0.711 | no |
| group 3 | CHKCI002 | 10-HDoHE | -0.150 | 0.035 | yes |
| group 3 | CHKCI002 | 11-HDoHE | -0.139 | 0.052 | no |
| group 3 | CHKCI002 | 13-HDoHE | -0.157 | 0.025 | yes |
| group 3 | CHKCI002 | 13-HOTrE | -0.181 | 0.008 | yes |
| group 3 | CHKCI002 | 13(S)-HOTrE(γ) | -0.202 | 0.004 | yes |
| group 3 | CHKCI002 | 14(S)-HDHA | -0.173 | 0.010 | yes |
| group 3 | CHKCI002 | 16-HDoHE | -0.141 | 0.048 | yes |
| group 3 | CHKCI002 | 2-Butyl-3-(4-hydroxybenzoyl)benzofuran | 0.135 | 0.059 | no |
| group 3 | CHKCI002 | 2-Hydroxybutanoic Acid | 0.024 | 0.816 | no |
| group 3 | CHKCI002 | 2,4-diacetamino-2,4,6-triphenoxy-D-mannopyranose | -0.011 | 0.894 | no |
| group 3 | CHKCI002 | 20-HDoHE | -0.152 | 0.032 | yes |
| group 3 | CHKCI002 | 8-HDoHE | -0.144 | 0.043 | yes |
| group 3 | CHKCI002 | 9(S)-HOTrE | -0.199 | 0.004 | yes |
| group 3 | CHKCI002 | Asp-Ile | -0.011 | 0.894 | no |
| group 3 | CHKCI002 | Carnitine C8:1 | -0.109 | 0.132 | no |
| group 3 | CHKCI002 | Carnitine C9:1-OH | -0.076 | 0.332 | no |
| group 3 | CHKCI002 | cyclo(gly-glu) | -0.106 | 0.146 | no |
| group 3 | CHKCI002 | FAHFA(8:0/10:0) | -0.138 | 0.053 | no |
| group 3 | CHKCI002 | Ile-Asp | -0.011 | 0.894 | no |
| group 3 | CHKCI002 | L-Fucose | 0.011 | 0.894 | no |
| group 3 | CHKCI002 | Leu-Asp | -0.077 | 0.326 | no |
| group 3 | CHKCI002 | Malonicacid | 0.023 | 0.816 | no |
| group 3 | CHKCI002 | Met-Phe | -0.058 | 0.502 | no |
| group 3 | CHKCI002 | N-Acetyl-5-aminosalicylic acid | -0.062 | 0.469 | no |
| group 3 | CHKCI002 | N-Acetylglycine | 0.029 | 0.812 | no |
| group 3 | CHKCI002 | N-lactoyl-phenylalanine | -0.051 | 0.585 | no |
| group 3 | CHKCI002 | Phe-Met | -0.081 | 0.299 | no |
| group 3 | CHKCI002 | Pro-Ile | -0.114 | 0.110 | no |
| group 3 | CHKCI002 | Trimethylamine-N-Oxide | 0.039 | 0.711 | no |
| group 3 | Clostridium_sensu_stricto_1 | (±)12-HEPE | -0.020 | 0.823 | no |
| group 3 | Clostridium_sensu_stricto_1 | (±)15-HEPE | -0.020 | 0.823 | no |
| group 3 | Clostridium_sensu_stricto_1 | (±)17-HDHA | -0.011 | 0.894 | no |
| group 3 | Clostridium_sensu_stricto_1 | (±)18-HEPE | -0.020 | 0.823 | no |
| group 3 | Clostridium_sensu_stricto_1 | (±)5-HEPE | 0.009 | 0.907 | no |
| group 3 | Clostridium_sensu_stricto_1 | (R)-3-Hydroxybutanoic acid | 0.060 | 0.479 | no |
| group 3 | Clostridium_sensu_stricto_1 | 1-Aminopropan-2-ol | -0.117 | 0.104 | no |
| group 3 | Clostridium_sensu_stricto_1 | 10-HDoHE | -0.022 | 0.823 | no |
| group 3 | Clostridium_sensu_stricto_1 | 11-HDoHE | -0.027 | 0.813 | no |
| group 3 | Clostridium_sensu_stricto_1 | 13-HDoHE | -0.024 | 0.816 | no |
| group 3 | Clostridium_sensu_stricto_1 | 13-HOTrE | -0.064 | 0.448 | no |
| group 3 | Clostridium_sensu_stricto_1 | 13(S)-HOTrE(γ) | -0.070 | 0.385 | no |
| group 3 | Clostridium_sensu_stricto_1 | 14(S)-HDHA | -0.028 | 0.812 | no |
| group 3 | Clostridium_sensu_stricto_1 | 16-HDoHE | -0.005 | 0.947 | no |
| group 3 | Clostridium_sensu_stricto_1 | 2-Butyl-3-(4-hydroxybenzoyl)benzofuran | 0.098 | 0.180 | no |
| group 3 | Clostridium_sensu_stricto_1 | 2-Hydroxybutanoic Acid | 0.060 | 0.479 | no |
| group 3 | Clostridium_sensu_stricto_1 | 2,4-diacetamino-2,4,6-triphenoxy-D-mannopyranose | -0.028 | 0.812 | no |
| group 3 | Clostridium_sensu_stricto_1 | 20-HDoHE | -0.005 | 0.947 | no |
| group 3 | Clostridium_sensu_stricto_1 | 8-HDoHE | -0.009 | 0.907 | no |
| group 3 | Clostridium_sensu_stricto_1 | 9(S)-HOTrE | -0.074 | 0.355 | no |
| group 3 | Clostridium_sensu_stricto_1 | Asp-Ile | -0.028 | 0.812 | no |
| group 3 | Clostridium_sensu_stricto_1 | Carnitine C8:1 | -0.104 | 0.151 | no |
| group 3 | Clostridium_sensu_stricto_1 | Carnitine C9:1-OH | -0.113 | 0.117 | no |
| group 3 | Clostridium_sensu_stricto_1 | cyclo(gly-glu) | 0.002 | 0.969 | no |
| group 3 | Clostridium_sensu_stricto_1 | FAHFA(8:0/10:0) | -0.172 | 0.010 | yes |
| group 3 | Clostridium_sensu_stricto_1 | Ile-Asp | -0.028 | 0.812 | no |
| group 3 | Clostridium_sensu_stricto_1 | L-Fucose | 0.047 | 0.624 | no |
| group 3 | Clostridium_sensu_stricto_1 | Leu-Asp | 0.013 | 0.889 | no |
| group 3 | Clostridium_sensu_stricto_1 | Malonicacid | 0.060 | 0.480 | no |
| group 3 | Clostridium_sensu_stricto_1 | Met-Phe | 0.019 | 0.829 | no |
| group 3 | Clostridium_sensu_stricto_1 | N-Acetyl-5-aminosalicylic acid | -0.046 | 0.638 | no |
| group 3 | Clostridium_sensu_stricto_1 | N-Acetylglycine | 0.162 | 0.019 | yes |
| group 3 | Clostridium_sensu_stricto_1 | N-lactoyl-phenylalanine | -0.153 | 0.031 | yes |
| group 3 | Clostridium_sensu_stricto_1 | Phe-Met | 0.014 | 0.882 | no |
| group 3 | Clostridium_sensu_stricto_1 | Pro-Ile | -0.039 | 0.711 | no |
| group 3 | Clostridium_sensu_stricto_1 | Trimethylamine-N-Oxide | -0.117 | 0.104 | no |
| group 3 | f__Oscillospiraceae | (±)12-HEPE | 0.002 | 0.969 | no |
| group 3 | f__Oscillospiraceae | (±)15-HEPE | 0.002 | 0.969 | no |
| group 3 | f__Oscillospiraceae | (±)17-HDHA | 0.030 | 0.812 | no |
| group 3 | f__Oscillospiraceae | (±)18-HEPE | 0.002 | 0.969 | no |
| group 3 | f__Oscillospiraceae | (±)5-HEPE | 0.021 | 0.823 | no |
| group 3 | f__Oscillospiraceae | (R)-3-Hydroxybutanoic acid | 0.018 | 0.833 | no |
| group 3 | f__Oscillospiraceae | 1-Aminopropan-2-ol | 0.100 | 0.176 | no |
| group 3 | f__Oscillospiraceae | 10-HDoHE | 0.018 | 0.833 | no |
| group 3 | f__Oscillospiraceae | 11-HDoHE | 0.021 | 0.823 | no |
| group 3 | f__Oscillospiraceae | 13-HDoHE | 0.024 | 0.816 | no |
| group 3 | f__Oscillospiraceae | 13-HOTrE | 0.046 | 0.638 | no |
| group 3 | f__Oscillospiraceae | 13(S)-HOTrE(γ) | 0.007 | 0.936 | no |
| group 3 | f__Oscillospiraceae | 14(S)-HDHA | 0.017 | 0.840 | no |
| group 3 | f__Oscillospiraceae | 16-HDoHE | 0.029 | 0.812 | no |
| group 3 | f__Oscillospiraceae | 2-Butyl-3-(4-hydroxybenzoyl)benzofuran | 0.093 | 0.219 | no |
| group 3 | f__Oscillospiraceae | 2-Hydroxybutanoic Acid | 0.018 | 0.833 | no |
| group 3 | f__Oscillospiraceae | 2,4-diacetamino-2,4,6-triphenoxy-D-mannopyranose | -0.012 | 0.894 | no |
| group 3 | f__Oscillospiraceae | 20-HDoHE | 0.028 | 0.812 | no |
| group 3 | f__Oscillospiraceae | 8-HDoHE | 0.024 | 0.816 | no |
| group 3 | f__Oscillospiraceae | 9(S)-HOTrE | 0.037 | 0.734 | no |
| group 3 | f__Oscillospiraceae | Asp-Ile | -0.012 | 0.894 | no |
| group 3 | f__Oscillospiraceae | Carnitine C8:1 | 0.019 | 0.826 | no |
| group 3 | f__Oscillospiraceae | Carnitine C9:1-OH | 0.062 | 0.471 | no |
| group 3 | f__Oscillospiraceae | cyclo(gly-glu) | -0.119 | 0.101 | no |
| group 3 | f__Oscillospiraceae | FAHFA(8:0/10:0) | 0.046 | 0.638 | no |
| group 3 | f__Oscillospiraceae | Ile-Asp | -0.012 | 0.894 | no |
| group 3 | f__Oscillospiraceae | L-Fucose | 0.024 | 0.816 | no |
| group 3 | f__Oscillospiraceae | Leu-Asp | -0.039 | 0.711 | no |
| group 3 | f__Oscillospiraceae | Malonicacid | 0.018 | 0.834 | no |
| group 3 | f__Oscillospiraceae | Met-Phe | -0.052 | 0.579 | no |
| group 3 | f__Oscillospiraceae | N-Acetyl-5-aminosalicylic acid | 0.025 | 0.816 | no |
| group 3 | f__Oscillospiraceae | N-Acetylglycine | 0.056 | 0.532 | no |
| group 3 | f__Oscillospiraceae | N-lactoyl-phenylalanine | -0.039 | 0.711 | no |
| group 3 | f__Oscillospiraceae | Phe-Met | -0.077 | 0.326 | no |
| group 3 | f__Oscillospiraceae | Pro-Ile | 0.029 | 0.812 | no |
| group 3 | f__Oscillospiraceae | Trimethylamine-N-Oxide | 0.100 | 0.176 | no |
| group 3 | f__Ruminococcaceae | (±)12-HEPE | -0.003 | 0.969 | no |
| group 3 | f__Ruminococcaceae | (±)15-HEPE | -0.003 | 0.969 | no |
| group 3 | f__Ruminococcaceae | (±)17-HDHA | 0.058 | 0.498 | no |
| group 3 | f__Ruminococcaceae | (±)18-HEPE | -0.003 | 0.969 | no |
| group 3 | f__Ruminococcaceae | (±)5-HEPE | -0.005 | 0.948 | no |
| group 3 | f__Ruminococcaceae | (R)-3-Hydroxybutanoic acid | 0.025 | 0.816 | no |
| group 3 | f__Ruminococcaceae | 1-Aminopropan-2-ol | 0.099 | 0.179 | no |
| group 3 | f__Ruminococcaceae | 10-HDoHE | 0.041 | 0.690 | no |
| group 3 | f__Ruminococcaceae | 11-HDoHE | 0.052 | 0.579 | no |
| group 3 | f__Ruminococcaceae | 13-HDoHE | 0.046 | 0.636 | no |
| group 3 | f__Ruminococcaceae | 13-HOTrE | 0.003 | 0.969 | no |
| group 3 | f__Ruminococcaceae | 13(S)-HOTrE(γ) | 0.015 | 0.871 | no |
| group 3 | f__Ruminococcaceae | 14(S)-HDHA | 0.028 | 0.812 | no |
| group 3 | f__Ruminococcaceae | 16-HDoHE | 0.057 | 0.513 | no |
| group 3 | f__Ruminococcaceae | 2-Butyl-3-(4-hydroxybenzoyl)benzofuran | 0.088 | 0.234 | no |
| group 3 | f__Ruminococcaceae | 2-Hydroxybutanoic Acid | 0.025 | 0.816 | no |
| group 3 | f__Ruminococcaceae | 2,4-diacetamino-2,4,6-triphenoxy-D-mannopyranose | -0.024 | 0.816 | no |
| group 3 | f__Ruminococcaceae | 20-HDoHE | 0.051 | 0.585 | no |
| group 3 | f__Ruminococcaceae | 8-HDoHE | 0.053 | 0.569 | no |
| group 3 | f__Ruminococcaceae | 9(S)-HOTrE | -0.013 | 0.889 | no |
| group 3 | f__Ruminococcaceae | Asp-Ile | -0.024 | 0.816 | no |
| group 3 | f__Ruminococcaceae | Carnitine C8:1 | -0.052 | 0.579 | no |
| group 3 | f__Ruminococcaceae | Carnitine C9:1-OH | -0.039 | 0.711 | no |
| group 3 | f__Ruminococcaceae | cyclo(gly-glu) | -0.100 | 0.176 | no |
| group 3 | f__Ruminococcaceae | FAHFA(8:0/10:0) | -0.110 | 0.131 | no |
| group 3 | f__Ruminococcaceae | Ile-Asp | -0.024 | 0.816 | no |
| group 3 | f__Ruminococcaceae | L-Fucose | 0.025 | 0.816 | no |
| group 3 | f__Ruminococcaceae | Leu-Asp | 0.024 | 0.816 | no |
| group 3 | f__Ruminococcaceae | Malonicacid | 0.024 | 0.816 | no |
| group 3 | f__Ruminococcaceae | Met-Phe | -0.016 | 0.850 | no |
| group 3 | f__Ruminococcaceae | N-Acetyl-5-aminosalicylic acid | 0.020 | 0.823 | no |
| group 3 | f__Ruminococcaceae | N-Acetylglycine | 0.053 | 0.574 | no |
| group 3 | f__Ruminococcaceae | N-lactoyl-phenylalanine | -0.132 | 0.065 | no |
| group 3 | f__Ruminococcaceae | Phe-Met | -0.020 | 0.823 | no |
| group 3 | f__Ruminococcaceae | Pro-Ile | -0.109 | 0.132 | no |
| group 3 | f__Ruminococcaceae | Trimethylamine-N-Oxide | 0.099 | 0.179 | no |
| group 3 | Lachnospiraceae_NK4A136_group | (±)12-HEPE | -0.029 | 0.812 | no |
| group 3 | Lachnospiraceae_NK4A136_group | (±)15-HEPE | -0.029 | 0.812 | no |
| group 3 | Lachnospiraceae_NK4A136_group | (±)17-HDHA | -0.033 | 0.810 | no |
| group 3 | Lachnospiraceae_NK4A136_group | (±)18-HEPE | -0.029 | 0.812 | no |
| group 3 | Lachnospiraceae_NK4A136_group | (±)5-HEPE | -0.034 | 0.787 | no |
| group 3 | Lachnospiraceae_NK4A136_group | (R)-3-Hydroxybutanoic acid | 0.090 | 0.224 | no |
| group 3 | Lachnospiraceae_NK4A136_group | 1-Aminopropan-2-ol | 0.108 | 0.133 | no |
| group 3 | Lachnospiraceae_NK4A136_group | 10-HDoHE | -0.029 | 0.812 | no |
| group 3 | Lachnospiraceae_NK4A136_group | 11-HDoHE | -0.025 | 0.816 | no |
| group 3 | Lachnospiraceae_NK4A136_group | 13-HDoHE | -0.025 | 0.816 | no |
| group 3 | Lachnospiraceae_NK4A136_group | 13-HOTrE | 0.010 | 0.896 | no |
| group 3 | Lachnospiraceae_NK4A136_group | 13(S)-HOTrE(γ) | -0.074 | 0.355 | no |
| group 3 | Lachnospiraceae_NK4A136_group | 14(S)-HDHA | -0.023 | 0.817 | no |
| group 3 | Lachnospiraceae_NK4A136_group | 16-HDoHE | -0.030 | 0.812 | no |
| group 3 | Lachnospiraceae_NK4A136_group | 2-Butyl-3-(4-hydroxybenzoyl)benzofuran | 0.096 | 0.193 | no |
| group 3 | Lachnospiraceae_NK4A136_group | 2-Hydroxybutanoic Acid | 0.090 | 0.224 | no |
| group 3 | Lachnospiraceae_NK4A136_group | 2,4-diacetamino-2,4,6-triphenoxy-D-mannopyranose | -0.070 | 0.380 | no |
| group 3 | Lachnospiraceae_NK4A136_group | 20-HDoHE | -0.031 | 0.812 | no |
| group 3 | Lachnospiraceae_NK4A136_group | 8-HDoHE | -0.017 | 0.838 | no |
| group 3 | Lachnospiraceae_NK4A136_group | 9(S)-HOTrE | -0.018 | 0.834 | no |
| group 3 | Lachnospiraceae_NK4A136_group | Asp-Ile | -0.070 | 0.380 | no |
| group 3 | Lachnospiraceae_NK4A136_group | Carnitine C8:1 | -0.019 | 0.829 | no |
| group 3 | Lachnospiraceae_NK4A136_group | Carnitine C9:1-OH | 0.001 | 0.987 | no |
| group 3 | Lachnospiraceae_NK4A136_group | cyclo(gly-glu) | -0.180 | 0.008 | yes |
| group 3 | Lachnospiraceae_NK4A136_group | FAHFA(8:0/10:0) | -0.092 | 0.219 | no |
| group 3 | Lachnospiraceae_NK4A136_group | Ile-Asp | -0.070 | 0.380 | no |
| group 3 | Lachnospiraceae_NK4A136_group | L-Fucose | 0.089 | 0.230 | no |
| group 3 | Lachnospiraceae_NK4A136_group | Leu-Asp | -0.041 | 0.699 | no |
| group 3 | Lachnospiraceae_NK4A136_group | Malonicacid | 0.090 | 0.224 | no |
| group 3 | Lachnospiraceae_NK4A136_group | Met-Phe | -0.046 | 0.638 | no |
| group 3 | Lachnospiraceae_NK4A136_group | N-Acetyl-5-aminosalicylic acid | -0.051 | 0.585 | no |
| group 3 | Lachnospiraceae_NK4A136_group | N-Acetylglycine | 0.052 | 0.579 | no |
| group 3 | Lachnospiraceae_NK4A136_group | N-lactoyl-phenylalanine | -0.154 | 0.031 | yes |
| group 3 | Lachnospiraceae_NK4A136_group | Phe-Met | -0.081 | 0.293 | no |
| group 3 | Lachnospiraceae_NK4A136_group | Pro-Ile | -0.051 | 0.585 | no |
| group 3 | Lachnospiraceae_NK4A136_group | Trimethylamine-N-Oxide | 0.108 | 0.133 | no |
| group 3 | Sellimonas | (±)12-HEPE | -0.124 | 0.082 | no |
| group 3 | Sellimonas | (±)15-HEPE | -0.124 | 0.082 | no |
| group 3 | Sellimonas | (±)17-HDHA | -0.072 | 0.372 | no |
| group 3 | Sellimonas | (±)18-HEPE | -0.124 | 0.082 | no |
| group 3 | Sellimonas | (±)5-HEPE | -0.115 | 0.108 | no |
| group 3 | Sellimonas | (R)-3-Hydroxybutanoic acid | 0.090 | 0.224 | no |
| group 3 | Sellimonas | 1-Aminopropan-2-ol | 0.092 | 0.219 | no |
| group 3 | Sellimonas | 10-HDoHE | -0.061 | 0.479 | no |
| group 3 | Sellimonas | 11-HDoHE | -0.061 | 0.479 | no |
| group 3 | Sellimonas | 13-HDoHE | -0.070 | 0.380 | no |
| group 3 | Sellimonas | 13-HOTrE | -0.098 | 0.180 | no |
| group 3 | Sellimonas | 13(S)-HOTrE(γ) | -0.071 | 0.380 | no |
| group 3 | Sellimonas | 14(S)-HDHA | -0.099 | 0.179 | no |
| group 3 | Sellimonas | 16-HDoHE | -0.070 | 0.380 | no |
| group 3 | Sellimonas | 2-Butyl-3-(4-hydroxybenzoyl)benzofuran | 0.103 | 0.156 | no |
| group 3 | Sellimonas | 2-Hydroxybutanoic Acid | 0.090 | 0.224 | no |
| group 3 | Sellimonas | 2,4-diacetamino-2,4,6-triphenoxy-D-mannopyranose | -0.045 | 0.642 | no |
| group 3 | Sellimonas | 20-HDoHE | -0.068 | 0.400 | no |
| group 3 | Sellimonas | 8-HDoHE | -0.060 | 0.479 | no |
| group 3 | Sellimonas | 9(S)-HOTrE | -0.098 | 0.181 | no |
| group 3 | Sellimonas | Asp-Ile | -0.045 | 0.642 | no |
| group 3 | Sellimonas | Carnitine C8:1 | -0.133 | 0.062 | no |
| group 3 | Sellimonas | Carnitine C9:1-OH | -0.117 | 0.104 | no |
| group 3 | Sellimonas | cyclo(gly-glu) | -0.088 | 0.234 | no |
| group 3 | Sellimonas | FAHFA(8:0/10:0) | -0.148 | 0.036 | yes |
| group 3 | Sellimonas | Ile-Asp | -0.045 | 0.642 | no |
| group 3 | Sellimonas | L-Fucose | 0.078 | 0.312 | no |
| group 3 | Sellimonas | Leu-Asp | -0.069 | 0.389 | no |
| group 3 | Sellimonas | Malonicacid | 0.090 | 0.224 | no |
| group 3 | Sellimonas | Met-Phe | -0.010 | 0.896 | no |
| group 3 | Sellimonas | N-Acetyl-5-aminosalicylic acid | -0.036 | 0.760 | no |
| group 3 | Sellimonas | N-Acetylglycine | 0.069 | 0.389 | no |
| group 3 | Sellimonas | N-lactoyl-phenylalanine | -0.106 | 0.146 | no |
| group 3 | Sellimonas | Phe-Met | -0.067 | 0.400 | no |
| group 3 | Sellimonas | Pro-Ile | -0.008 | 0.911 | no |
| group 3 | Sellimonas | Trimethylamine-N-Oxide | 0.092 | 0.219 | no |
| group 3 | Terrisporobacter | (±)12-HEPE | -0.115 | 0.108 | no |
| group 3 | Terrisporobacter | (±)15-HEPE | -0.115 | 0.108 | no |
| group 3 | Terrisporobacter | (±)17-HDHA | -0.134 | 0.059 | no |
| group 3 | Terrisporobacter | (±)18-HEPE | -0.115 | 0.108 | no |
| group 3 | Terrisporobacter | (±)5-HEPE | -0.108 | 0.136 | no |
| group 3 | Terrisporobacter | (R)-3-Hydroxybutanoic acid | -0.023 | 0.817 | no |
| group 3 | Terrisporobacter | 1-Aminopropan-2-ol | -0.178 | 0.008 | yes |
| group 3 | Terrisporobacter | 10-HDoHE | -0.123 | 0.084 | no |
| group 3 | Terrisporobacter | 11-HDoHE | -0.142 | 0.047 | yes |
| group 3 | Terrisporobacter | 13-HDoHE | -0.131 | 0.065 | no |
| group 3 | Terrisporobacter | 13-HOTrE | -0.176 | 0.009 | yes |
| group 3 | Terrisporobacter | 13(S)-HOTrE(γ) | -0.123 | 0.085 | no |
| group 3 | Terrisporobacter | 14(S)-HDHA | -0.130 | 0.068 | no |
| group 3 | Terrisporobacter | 16-HDoHE | -0.128 | 0.072 | no |
| group 3 | Terrisporobacter | 2-Butyl-3-(4-hydroxybenzoyl)benzofuran | 0.112 | 0.118 | no |
| group 3 | Terrisporobacter | 2-Hydroxybutanoic Acid | -0.023 | 0.817 | no |
| group 3 | Terrisporobacter | 2,4-diacetamino-2,4,6-triphenoxy-D-mannopyranose | -0.030 | 0.812 | no |
| group 3 | Terrisporobacter | 20-HDoHE | -0.128 | 0.072 | no |
| group 3 | Terrisporobacter | 8-HDoHE | -0.119 | 0.101 | no |
| group 3 | Terrisporobacter | 9(S)-HOTrE | -0.177 | 0.009 | yes |
| group 3 | Terrisporobacter | Asp-Ile | -0.030 | 0.812 | no |
| group 3 | Terrisporobacter | Carnitine C8:1 | -0.137 | 0.054 | no |
| group 3 | Terrisporobacter | Carnitine C9:1-OH | -0.118 | 0.104 | no |
| group 3 | Terrisporobacter | cyclo(gly-glu) | -0.044 | 0.656 | no |
| group 3 | Terrisporobacter | FAHFA(8:0/10:0) | -0.182 | 0.008 | yes |
| group 3 | Terrisporobacter | Ile-Asp | -0.030 | 0.812 | no |
| group 3 | Terrisporobacter | L-Fucose | -0.049 | 0.597 | no |
| group 3 | Terrisporobacter | Leu-Asp | -0.062 | 0.469 | no |
| group 3 | Terrisporobacter | Malonicacid | -0.021 | 0.823 | no |
| group 3 | Terrisporobacter | Met-Phe | -0.028 | 0.812 | no |
| group 3 | Terrisporobacter | N-Acetyl-5-aminosalicylic acid | -0.145 | 0.042 | yes |
| group 3 | Terrisporobacter | N-Acetylglycine | 0.105 | 0.146 | no |
| group 3 | Terrisporobacter | N-lactoyl-phenylalanine | -0.147 | 0.036 | yes |
| group 3 | Terrisporobacter | Phe-Met | -0.037 | 0.734 | no |
| group 3 | Terrisporobacter | Pro-Ile | -0.071 | 0.380 | no |
| group 3 | Terrisporobacter | Trimethylamine-N-Oxide | -0.178 | 0.008 | yes |
| group 3 | Turicibacter | (±)12-HEPE | -0.049 | 0.597 | no |
| group 3 | Turicibacter | (±)15-HEPE | -0.049 | 0.597 | no |
| group 3 | Turicibacter | (±)17-HDHA | -0.085 | 0.261 | no |
| group 3 | Turicibacter | (±)18-HEPE | -0.049 | 0.597 | no |
| group 3 | Turicibacter | (±)5-HEPE | -0.045 | 0.642 | no |
| group 3 | Turicibacter | (R)-3-Hydroxybutanoic acid | -0.022 | 0.823 | no |
| group 3 | Turicibacter | 1-Aminopropan-2-ol | -0.241 | 0.000 | yes |
| group 3 | Turicibacter | 10-HDoHE | -0.084 | 0.265 | no |
| group 3 | Turicibacter | 11-HDoHE | -0.086 | 0.248 | no |
| group 3 | Turicibacter | 13-HDoHE | -0.090 | 0.224 | no |
| group 3 | Turicibacter | 13-HOTrE | -0.136 | 0.059 | no |
| group 3 | Turicibacter | 13(S)-HOTrE(γ) | -0.060 | 0.480 | no |
| group 3 | Turicibacter | 14(S)-HDHA | -0.080 | 0.305 | no |
| group 3 | Turicibacter | 16-HDoHE | -0.080 | 0.304 | no |
| group 3 | Turicibacter | 2-Butyl-3-(4-hydroxybenzoyl)benzofuran | 0.087 | 0.244 | no |
| group 3 | Turicibacter | 2-Hydroxybutanoic Acid | -0.022 | 0.823 | no |
| group 3 | Turicibacter | 2,4-diacetamino-2,4,6-triphenoxy-D-mannopyranose | -0.028 | 0.812 | no |
| group 3 | Turicibacter | 20-HDoHE | -0.079 | 0.312 | no |
| group 3 | Turicibacter | 8-HDoHE | -0.075 | 0.339 | no |
| group 3 | Turicibacter | 9(S)-HOTrE | -0.127 | 0.075 | no |
| group 3 | Turicibacter | Asp-Ile | -0.028 | 0.812 | no |
| group 3 | Turicibacter | Carnitine C8:1 | -0.131 | 0.065 | no |
| group 3 | Turicibacter | Carnitine C9:1-OH | -0.127 | 0.075 | no |
| group 3 | Turicibacter | cyclo(gly-glu) | -0.009 | 0.907 | no |
| group 3 | Turicibacter | FAHFA(8:0/10:0) | -0.179 | 0.008 | yes |
| group 3 | Turicibacter | Ile-Asp | -0.028 | 0.812 | no |
| group 3 | Turicibacter | L-Fucose | -0.040 | 0.711 | no |
| group 3 | Turicibacter | Leu-Asp | -0.020 | 0.823 | no |
| group 3 | Turicibacter | Malonicacid | -0.021 | 0.823 | no |
| group 3 | Turicibacter | Met-Phe | 0.022 | 0.822 | no |
| group 3 | Turicibacter | N-Acetyl-5-aminosalicylic acid | -0.068 | 0.398 | no |
| group 3 | Turicibacter | N-Acetylglycine | 0.090 | 0.224 | no |
| group 3 | Turicibacter | N-lactoyl-phenylalanine | -0.092 | 0.219 | no |
| group 3 | Turicibacter | Phe-Met | -0.011 | 0.896 | no |
| group 3 | Turicibacter | Pro-Ile | 0.009 | 0.907 | no |
| group 3 | Turicibacter | Trimethylamine-N-Oxide | -0.241 | 0.000 | yes |
| group 4 | CHKCI002 | (±)12-HEPE | -0.196 | 0.003 | yes |
| group 4 | CHKCI002 | (±)15-HEPE | -0.196 | 0.003 | yes |
| group 4 | CHKCI002 | (±)17-HDHA | -0.148 | 0.031 | yes |
| group 4 | CHKCI002 | (±)18-HEPE | -0.196 | 0.003 | yes |
| group 4 | CHKCI002 | (±)5-HEPE | -0.171 | 0.010 | yes |
| group 4 | CHKCI002 | 10-HDoHE | -0.150 | 0.030 | yes |
| group 4 | CHKCI002 | 11-HDoHE | -0.139 | 0.047 | yes |
| group 4 | CHKCI002 | 13-HDoHE | -0.157 | 0.021 | yes |
| group 4 | CHKCI002 | 13-HOTrE | -0.181 | 0.007 | yes |
| group 4 | CHKCI002 | 13(S)-HOTrE(γ) | -0.202 | 0.003 | yes |
| group 4 | CHKCI002 | 14(S)-HDHA | -0.173 | 0.010 | yes |
| group 4 | CHKCI002 | 15(S)-HETrE | -0.120 | 0.090 | no |
| group 4 | CHKCI002 | 16-HDoHE | -0.141 | 0.043 | yes |
| group 4 | CHKCI002 | 2-Butyl-3-(4-hydroxybenzoyl)benzofuran | 0.135 | 0.054 | no |
| group 4 | CHKCI002 | 2,4-diacetamino-2,4,6-triphenoxy-D-mannopyranose | -0.011 | 0.902 | no |
| group 4 | CHKCI002 | 20-HDoHE | -0.152 | 0.028 | yes |
| group 4 | CHKCI002 | 3-Epideoxycholic acid | -0.109 | 0.132 | no |
| group 4 | CHKCI002 | 4-Hydroxytryptamine | -0.031 | 0.725 | no |
| group 4 | CHKCI002 | 5-HETrE | -0.120 | 0.090 | no |
| group 4 | CHKCI002 | 8-Azaguanine | 0.017 | 0.833 | no |
| group 4 | CHKCI002 | 8-HDoHE | -0.144 | 0.038 | yes |
| group 4 | CHKCI002 | 8(S)-HETrE | -0.120 | 0.090 | no |
| group 4 | CHKCI002 | 9(S)-HOTrE | -0.199 | 0.003 | yes |
| group 4 | CHKCI002 | Asp-Ile | -0.011 | 0.902 | no |
| group 4 | CHKCI002 | Carnitine C8:1 | -0.109 | 0.132 | no |
| group 4 | CHKCI002 | CMPentylF | -0.040 | 0.630 | no |
| group 4 | CHKCI002 | cyclo(gly-glu) | -0.106 | 0.148 | no |
| group 4 | CHKCI002 | Cyclo(Pro-Leu) | -0.093 | 0.215 | no |
| group 4 | CHKCI002 | EPA | -0.197 | 0.003 | yes |
| group 4 | CHKCI002 | FAHFA(8:0/10:0) | -0.138 | 0.047 | yes |
| group 4 | CHKCI002 | FFA(18:3) | -0.199 | 0.003 | yes |
| group 4 | CHKCI002 | FFA(18:4) | -0.186 | 0.005 | yes |
| group 4 | CHKCI002 | Glu-Thr | -0.044 | 0.584 | no |
| group 4 | CHKCI002 | Hexadecanedioic acid | 0.095 | 0.200 | no |
| group 4 | CHKCI002 | Ile-Asp | -0.011 | 0.902 | no |
| group 4 | CHKCI002 | Isochodeoxycholic acid | -0.147 | 0.033 | yes |
| group 4 | CHKCI002 | L-Glutamic Acid | -0.108 | 0.136 | no |
| group 4 | CHKCI002 | L-threo-3-Methylaspartate | -0.108 | 0.136 | no |
| group 4 | CHKCI002 | LPC(0:0/14:0) | -0.102 | 0.165 | no |
| group 4 | CHKCI002 | LPE(0:0/20:5) | -0.124 | 0.078 | no |
| group 4 | CHKCI002 | LPE(20:5/0:0) | -0.124 | 0.078 | no |
| group 4 | CHKCI002 | Met-Phe | -0.058 | 0.473 | no |
| group 4 | CHKCI002 | N'-Methyl-2-pyridone-5-carboxamide | 0.006 | 0.941 | no |
| group 4 | CHKCI002 | N-acetyl-D-Lactosamine | 0.043 | 0.611 | no |
| group 4 | CHKCI002 | N-Acetylglycine | 0.029 | 0.725 | no |
| group 4 | CHKCI002 | N-lactoyl-phenylalanine | -0.051 | 0.534 | no |
| group 4 | CHKCI002 | N(Alpha)-Acetyl-Epsilon-(2-Propenal)Lysine | -0.137 | 0.051 | no |
| group 4 | CHKCI002 | Oxypurinol | 0.017 | 0.833 | no |
| group 4 | CHKCI002 | P-Toluenesulfonamide | -0.049 | 0.542 | no |
| group 4 | CHKCI002 | Phe-Ala-Ser | -0.078 | 0.298 | no |
| group 4 | CHKCI002 | Phe-Met | -0.081 | 0.281 | no |
| group 4 | CHKCI002 | Phe-Tyr | -0.085 | 0.256 | no |
| group 4 | CHKCI002 | Pinolenic acid | -0.190 | 0.004 | yes |
| group 4 | CHKCI002 | Pro-Asp | -0.119 | 0.093 | no |
| group 4 | CHKCI002 | Pro-Ile | -0.114 | 0.111 | no |
| group 4 | CHKCI002 | Ser-Phe-Ala | -0.078 | 0.298 | no |
| group 4 | CHKCI002 | Trans-3-Hydroxycotinine | -0.005 | 0.941 | no |
| group 4 | Clostridium_sensu_stricto_1 | (±)12-HEPE | -0.020 | 0.812 | no |
| group 4 | Clostridium_sensu_stricto_1 | (±)15-HEPE | -0.020 | 0.812 | no |
| group 4 | Clostridium_sensu_stricto_1 | (±)17-HDHA | -0.011 | 0.902 | no |
| group 4 | Clostridium_sensu_stricto_1 | (±)18-HEPE | -0.020 | 0.812 | no |
| group 4 | Clostridium_sensu_stricto_1 | (±)5-HEPE | 0.009 | 0.920 | no |
| group 4 | Clostridium_sensu_stricto_1 | 10-HDoHE | -0.022 | 0.791 | no |
| group 4 | Clostridium_sensu_stricto_1 | 11-HDoHE | -0.027 | 0.731 | no |
| group 4 | Clostridium_sensu_stricto_1 | 13-HDoHE | -0.024 | 0.770 | no |
| group 4 | Clostridium_sensu_stricto_1 | 13-HOTrE | -0.064 | 0.428 | no |
| group 4 | Clostridium_sensu_stricto_1 | 13(S)-HOTrE(γ) | -0.070 | 0.369 | no |
| group 4 | Clostridium_sensu_stricto_1 | 14(S)-HDHA | -0.028 | 0.727 | no |
| group 4 | Clostridium_sensu_stricto_1 | 15(S)-HETrE | -0.034 | 0.706 | no |
| group 4 | Clostridium_sensu_stricto_1 | 16-HDoHE | -0.005 | 0.941 | no |
| group 4 | Clostridium_sensu_stricto_1 | 2-Butyl-3-(4-hydroxybenzoyl)benzofuran | 0.098 | 0.184 | no |
| group 4 | Clostridium_sensu_stricto_1 | 2,4-diacetamino-2,4,6-triphenoxy-D-mannopyranose | -0.028 | 0.725 | no |
| group 4 | Clostridium_sensu_stricto_1 | 20-HDoHE | -0.005 | 0.941 | no |
| group 4 | Clostridium_sensu_stricto_1 | 3-Epideoxycholic acid | -0.075 | 0.321 | no |
| group 4 | Clostridium_sensu_stricto_1 | 4-Hydroxytryptamine | 0.032 | 0.725 | no |
| group 4 | Clostridium_sensu_stricto_1 | 5-HETrE | -0.034 | 0.706 | no |
| group 4 | Clostridium_sensu_stricto_1 | 8-Azaguanine | -0.083 | 0.265 | no |
| group 4 | Clostridium_sensu_stricto_1 | 8-HDoHE | -0.009 | 0.920 | no |
| group 4 | Clostridium_sensu_stricto_1 | 8(S)-HETrE | -0.034 | 0.706 | no |
| group 4 | Clostridium_sensu_stricto_1 | 9(S)-HOTrE | -0.074 | 0.327 | no |
| group 4 | Clostridium_sensu_stricto_1 | Asp-Ile | -0.028 | 0.725 | no |
| group 4 | Clostridium_sensu_stricto_1 | Carnitine C8:1 | -0.104 | 0.155 | no |
| group 4 | Clostridium_sensu_stricto_1 | CMPentylF | -0.057 | 0.473 | no |
| group 4 | Clostridium_sensu_stricto_1 | cyclo(gly-glu) | 0.002 | 0.970 | no |
| group 4 | Clostridium_sensu_stricto_1 | Cyclo(Pro-Leu) | -0.032 | 0.719 | no |
| group 4 | Clostridium_sensu_stricto_1 | EPA | -0.074 | 0.327 | no |
| group 4 | Clostridium_sensu_stricto_1 | FAHFA(8:0/10:0) | -0.172 | 0.010 | yes |
| group 4 | Clostridium_sensu_stricto_1 | FFA(18:3) | -0.133 | 0.055 | no |
| group 4 | Clostridium_sensu_stricto_1 | FFA(18:4) | -0.098 | 0.184 | no |
| group 4 | Clostridium_sensu_stricto_1 | Glu-Thr | -0.036 | 0.689 | no |
| group 4 | Clostridium_sensu_stricto_1 | Hexadecanedioic acid | 0.064 | 0.428 | no |
| group 4 | Clostridium_sensu_stricto_1 | Ile-Asp | -0.028 | 0.725 | no |
| group 4 | Clostridium_sensu_stricto_1 | Isochodeoxycholic acid | -0.077 | 0.302 | no |
| group 4 | Clostridium_sensu_stricto_1 | L-Glutamic Acid | -0.029 | 0.725 | no |
| group 4 | Clostridium_sensu_stricto_1 | L-threo-3-Methylaspartate | -0.029 | 0.725 | no |
| group 4 | Clostridium_sensu_stricto_1 | LPC(0:0/14:0) | -0.159 | 0.020 | yes |
| group 4 | Clostridium_sensu_stricto_1 | LPE(0:0/20:5) | -0.030 | 0.725 | no |
| group 4 | Clostridium_sensu_stricto_1 | LPE(20:5/0:0) | -0.030 | 0.725 | no |
| group 4 | Clostridium_sensu_stricto_1 | Met-Phe | 0.019 | 0.820 | no |
| group 4 | Clostridium_sensu_stricto_1 | N'-Methyl-2-pyridone-5-carboxamide | -0.073 | 0.333 | no |
| group 4 | Clostridium_sensu_stricto_1 | N-acetyl-D-Lactosamine | -0.058 | 0.473 | no |
| group 4 | Clostridium_sensu_stricto_1 | N-Acetylglycine | 0.162 | 0.018 | yes |
| group 4 | Clostridium_sensu_stricto_1 | N-lactoyl-phenylalanine | -0.153 | 0.027 | yes |
| group 4 | Clostridium_sensu_stricto_1 | N(Alpha)-Acetyl-Epsilon-(2-Propenal)Lysine | -0.018 | 0.833 | no |
| group 4 | Clostridium_sensu_stricto_1 | Oxypurinol | -0.083 | 0.265 | no |
| group 4 | Clostridium_sensu_stricto_1 | P-Toluenesulfonamide | -0.012 | 0.899 | no |
| group 4 | Clostridium_sensu_stricto_1 | Phe-Ala-Ser | 0.062 | 0.447 | no |
| group 4 | Clostridium_sensu_stricto_1 | Phe-Met | 0.014 | 0.879 | no |
| group 4 | Clostridium_sensu_stricto_1 | Phe-Tyr | 0.000 | 0.998 | no |
| group 4 | Clostridium_sensu_stricto_1 | Pinolenic acid | -0.126 | 0.073 | no |
| group 4 | Clostridium_sensu_stricto_1 | Pro-Asp | -0.093 | 0.215 | no |
| group 4 | Clostridium_sensu_stricto_1 | Pro-Ile | -0.039 | 0.641 | no |
| group 4 | Clostridium_sensu_stricto_1 | Ser-Phe-Ala | 0.062 | 0.447 | no |
| group 4 | Clostridium_sensu_stricto_1 | Trans-3-Hydroxycotinine | 0.099 | 0.183 | no |
| group 4 | Romboutsia | (±)12-HEPE | -0.057 | 0.473 | no |
| group 4 | Romboutsia | (±)15-HEPE | -0.057 | 0.473 | no |
| group 4 | Romboutsia | (±)17-HDHA | -0.050 | 0.540 | no |
| group 4 | Romboutsia | (±)18-HEPE | -0.057 | 0.473 | no |
| group 4 | Romboutsia | (±)5-HEPE | -0.065 | 0.418 | no |
| group 4 | Romboutsia | 10-HDoHE | -0.045 | 0.579 | no |
| group 4 | Romboutsia | 11-HDoHE | -0.051 | 0.534 | no |
| group 4 | Romboutsia | 13-HDoHE | -0.046 | 0.573 | no |
| group 4 | Romboutsia | 13-HOTrE | -0.101 | 0.171 | no |
| group 4 | Romboutsia | 13(S)-HOTrE(γ) | -0.082 | 0.265 | no |
| group 4 | Romboutsia | 14(S)-HDHA | -0.041 | 0.622 | no |
| group 4 | Romboutsia | 15(S)-HETrE | -0.061 | 0.448 | no |
| group 4 | Romboutsia | 16-HDoHE | -0.056 | 0.479 | no |
| group 4 | Romboutsia | 2-Butyl-3-(4-hydroxybenzoyl)benzofuran | 0.026 | 0.756 | no |
| group 4 | Romboutsia | 2,4-diacetamino-2,4,6-triphenoxy-D-mannopyranose | -0.029 | 0.725 | no |
| group 4 | Romboutsia | 20-HDoHE | -0.047 | 0.559 | no |
| group 4 | Romboutsia | 3-Epideoxycholic acid | -0.025 | 0.761 | no |
| group 4 | Romboutsia | 4-Hydroxytryptamine | 0.098 | 0.184 | no |
| group 4 | Romboutsia | 5-HETrE | -0.061 | 0.448 | no |
| group 4 | Romboutsia | 8-Azaguanine | -0.055 | 0.479 | no |
| group 4 | Romboutsia | 8-HDoHE | -0.038 | 0.662 | no |
| group 4 | Romboutsia | 8(S)-HETrE | -0.061 | 0.448 | no |
| group 4 | Romboutsia | 9(S)-HOTrE | -0.090 | 0.231 | no |
| group 4 | Romboutsia | Asp-Ile | -0.029 | 0.725 | no |
| group 4 | Romboutsia | Carnitine C8:1 | -0.090 | 0.231 | no |
| group 4 | Romboutsia | CMPentylF | -0.050 | 0.540 | no |
| group 4 | Romboutsia | cyclo(gly-glu) | -0.019 | 0.820 | no |
| group 4 | Romboutsia | Cyclo(Pro-Leu) | -0.004 | 0.963 | no |
| group 4 | Romboutsia | EPA | -0.088 | 0.243 | no |
| group 4 | Romboutsia | FAHFA(8:0/10:0) | -0.066 | 0.415 | no |
| group 4 | Romboutsia | FFA(18:3) | -0.118 | 0.095 | no |
| group 4 | Romboutsia | FFA(18:4) | -0.094 | 0.211 | no |
| group 4 | Romboutsia | Glu-Thr | -0.102 | 0.163 | no |
| group 4 | Romboutsia | Hexadecanedioic acid | 0.017 | 0.839 | no |
| group 4 | Romboutsia | Ile-Asp | -0.029 | 0.725 | no |
| group 4 | Romboutsia | Isochodeoxycholic acid | -0.003 | 0.970 | no |
| group 4 | Romboutsia | L-Glutamic Acid | 0.008 | 0.925 | no |
| group 4 | Romboutsia | L-threo-3-Methylaspartate | 0.008 | 0.925 | no |
| group 4 | Romboutsia | LPC(0:0/14:0) | -0.088 | 0.241 | no |
| group 4 | Romboutsia | LPE(0:0/20:5) | -0.058 | 0.473 | no |
| group 4 | Romboutsia | LPE(20:5/0:0) | -0.058 | 0.473 | no |
| group 4 | Romboutsia | Met-Phe | -0.032 | 0.719 | no |
| group 4 | Romboutsia | N'-Methyl-2-pyridone-5-carboxamide | -0.033 | 0.712 | no |
| group 4 | Romboutsia | N-acetyl-D-Lactosamine | -0.020 | 0.812 | no |
| group 4 | Romboutsia | N-Acetylglycine | 0.100 | 0.179 | no |
| group 4 | Romboutsia | N-lactoyl-phenylalanine | -0.070 | 0.369 | no |
| group 4 | Romboutsia | N(Alpha)-Acetyl-Epsilon-(2-Propenal)Lysine | -0.051 | 0.534 | no |
| group 4 | Romboutsia | Oxypurinol | -0.055 | 0.479 | no |
| group 4 | Romboutsia | P-Toluenesulfonamide | -0.084 | 0.257 | no |
| group 4 | Romboutsia | Phe-Ala-Ser | 0.006 | 0.941 | no |
| group 4 | Romboutsia | Phe-Met | -0.041 | 0.628 | no |
| group 4 | Romboutsia | Phe-Tyr | -0.020 | 0.812 | no |
| group 4 | Romboutsia | Pinolenic acid | -0.111 | 0.123 | no |
| group 4 | Romboutsia | Pro-Asp | -0.064 | 0.428 | no |
| group 4 | Romboutsia | Pro-Ile | -0.003 | 0.970 | no |
| group 4 | Romboutsia | Ser-Phe-Ala | 0.006 | 0.941 | no |
| group 4 | Romboutsia | Trans-3-Hydroxycotinine | 0.120 | 0.091 | no |
| group 4 | Turicibacter | (±)12-HEPE | -0.049 | 0.540 | no |
| group 4 | Turicibacter | (±)15-HEPE | -0.049 | 0.540 | no |
| group 4 | Turicibacter | (±)17-HDHA | -0.085 | 0.257 | no |
| group 4 | Turicibacter | (±)18-HEPE | -0.049 | 0.540 | no |
| group 4 | Turicibacter | (±)5-HEPE | -0.045 | 0.584 | no |
| group 4 | Turicibacter | 10-HDoHE | -0.084 | 0.259 | no |
| group 4 | Turicibacter | 11-HDoHE | -0.086 | 0.252 | no |
| group 4 | Turicibacter | 13-HDoHE | -0.090 | 0.231 | no |
| group 4 | Turicibacter | 13-HOTrE | -0.136 | 0.052 | no |
| group 4 | Turicibacter | 13(S)-HOTrE(γ) | -0.060 | 0.465 | no |
| group 4 | Turicibacter | 14(S)-HDHA | -0.080 | 0.286 | no |
| group 4 | Turicibacter | 15(S)-HETrE | -0.057 | 0.473 | no |
| group 4 | Turicibacter | 16-HDoHE | -0.080 | 0.285 | no |
| group 4 | Turicibacter | 2-Butyl-3-(4-hydroxybenzoyl)benzofuran | 0.087 | 0.249 | no |
| group 4 | Turicibacter | 2,4-diacetamino-2,4,6-triphenoxy-D-mannopyranose | -0.028 | 0.725 | no |
| group 4 | Turicibacter | 20-HDoHE | -0.079 | 0.292 | no |
| group 4 | Turicibacter | 3-Epideoxycholic acid | -0.088 | 0.241 | no |
| group 4 | Turicibacter | 4-Hydroxytryptamine | 0.115 | 0.109 | no |
| group 4 | Turicibacter | 5-HETrE | -0.057 | 0.473 | no |
| group 4 | Turicibacter | 8-Azaguanine | -0.133 | 0.055 | no |
| group 4 | Turicibacter | 8-HDoHE | -0.075 | 0.318 | no |
| group 4 | Turicibacter | 8(S)-HETrE | -0.057 | 0.473 | no |
| group 4 | Turicibacter | 9(S)-HOTrE | -0.127 | 0.073 | no |
| group 4 | Turicibacter | Asp-Ile | -0.028 | 0.725 | no |
| group 4 | Turicibacter | Carnitine C8:1 | -0.131 | 0.059 | no |
| group 4 | Turicibacter | CMPentylF | -0.048 | 0.548 | no |
| group 4 | Turicibacter | cyclo(gly-glu) | -0.009 | 0.920 | no |
| group 4 | Turicibacter | Cyclo(Pro-Leu) | 0.036 | 0.690 | no |
| group 4 | Turicibacter | EPA | -0.082 | 0.265 | no |
| group 4 | Turicibacter | FAHFA(8:0/10:0) | -0.179 | 0.007 | yes |
| group 4 | Turicibacter | FFA(18:3) | -0.178 | 0.007 | yes |
| group 4 | Turicibacter | FFA(18:4) | -0.117 | 0.097 | no |
| group 4 | Turicibacter | Glu-Thr | -0.054 | 0.493 | no |
| group 4 | Turicibacter | Hexadecanedioic acid | 0.048 | 0.548 | no |
| group 4 | Turicibacter | Ile-Asp | -0.028 | 0.725 | no |
| group 4 | Turicibacter | Isochodeoxycholic acid | -0.073 | 0.333 | no |
| group 4 | Turicibacter | L-Glutamic Acid | -0.024 | 0.768 | no |
| group 4 | Turicibacter | L-threo-3-Methylaspartate | -0.024 | 0.768 | no |
| group 4 | Turicibacter | LPC(0:0/14:0) | -0.103 | 0.163 | no |
| group 4 | Turicibacter | LPE(0:0/20:5) | -0.042 | 0.611 | no |
| group 4 | Turicibacter | LPE(20:5/0:0) | -0.042 | 0.611 | no |
| group 4 | Turicibacter | Met-Phe | 0.022 | 0.791 | no |
| group 4 | Turicibacter | N'-Methyl-2-pyridone-5-carboxamide | -0.112 | 0.123 | no |
| group 4 | Turicibacter | N-acetyl-D-Lactosamine | -0.068 | 0.391 | no |
| group 4 | Turicibacter | N-Acetylglycine | 0.090 | 0.231 | no |
| group 4 | Turicibacter | N-lactoyl-phenylalanine | -0.092 | 0.218 | no |
| group 4 | Turicibacter | N(Alpha)-Acetyl-Epsilon-(2-Propenal)Lysine | -0.002 | 0.971 | no |
| group 4 | Turicibacter | Oxypurinol | -0.133 | 0.055 | no |
| group 4 | Turicibacter | P-Toluenesulfonamide | -0.085 | 0.257 | no |
| group 4 | Turicibacter | Phe-Ala-Ser | 0.034 | 0.706 | no |
| group 4 | Turicibacter | Phe-Met | -0.011 | 0.906 | no |
| group 4 | Turicibacter | Phe-Tyr | -0.014 | 0.879 | no |
| group 4 | Turicibacter | Pinolenic acid | -0.159 | 0.020 | yes |
| group 4 | Turicibacter | Pro-Asp | -0.054 | 0.500 | no |
| group 4 | Turicibacter | Pro-Ile | 0.009 | 0.920 | no |
| group 4 | Turicibacter | Ser-Phe-Ala | 0.034 | 0.706 | no |
| group 4 | Turicibacter | Trans-3-Hydroxycotinine | 0.148 | 0.031 | yes |

**Table S12.** Associations between characteristic microbiota and differential metabolites in group 2 of females.

| Bacteria_genus | Metabolites | Correlation | P_value | Significant |
| --- | --- | --- | --- | --- |
| [Eubacterium]_brachy_group | (±)12-HEPE | -0.087 | 0.055 | no |
| [Eubacterium]_brachy_group | (±)15-HEPE | -0.087 | 0.055 | no |
| [Eubacterium]_brachy_group | (±)18-HEPE | -0.087 | 0.055 | no |
| [Eubacterium]_brachy_group | (±)5-HEPE | -0.097 | 0.041 | yes |
| [Eubacterium]_brachy_group | (S)-2-Hydroxy-3-phenylpropanoic acid | 0.016 | 0.709 | no |
| [Eubacterium]_brachy_group | 13-HOTrE | -0.073 | 0.095 | no |
| [Eubacterium]_brachy_group | 2-(4-hydroxyphenyl) propionate | 0.020 | 0.677 | no |
| [Eubacterium]_brachy_group | 9(S)-HOTrE | -0.074 | 0.095 | no |
| [Eubacterium]_brachy_group | Carnitine C8:1 | -0.121 | 0.010 | yes |
| [Eubacterium]_brachy_group | EPA | -0.132 | 0.010 | yes |
| Parabacteroides | (±)12-HEPE | -0.124 | 0.010 | yes |
| Parabacteroides | (±)15-HEPE | -0.124 | 0.010 | yes |
| Parabacteroides | (±)18-HEPE | -0.124 | 0.010 | yes |
| Parabacteroides | (±)5-HEPE | -0.095 | 0.042 | yes |
| Parabacteroides | (S)-2-Hydroxy-3-phenylpropanoic acid | 0.123 | 0.010 | yes |
| Parabacteroides | 13-HOTrE | -0.116 | 0.013 | yes |
| Parabacteroides | 2-(4-hydroxyphenyl) propionate | 0.128 | 0.010 | yes |
| Parabacteroides | 9(S)-HOTrE | -0.128 | 0.010 | yes |
| Parabacteroides | Carnitine C8:1 | -0.077 | 0.089 | no |
| Parabacteroides | EPA | -0.125 | 0.010 | yes |
